# Recoverable Fluorination Accelerates Ring‐Opening Copolymerisation and Enables Post‐Polymerisation‐Modification of Polyesters

**DOI:** 10.1002/anie.202515104

**Published:** 2025-10-20

**Authors:** Christoph Fornacon‐Wood, Luca Steiner, Chengzhang Xu, Beate Paulus, Alex J. Plajer

**Affiliations:** ^1^ Makromolekulare Chemie Universität Bayreuth Universitätsstraße 30 Bayreuth 95447 Germany; ^2^ Institut für Chemie und Biochemie Freie Universität Berlin Arnimallee 22 14195 Berlin Germany; ^3^ Bayrisches Polymer Institut (BPI) Universität Bayreuth Universitätsstraße 30 95447 Bayreuth Germany

**Keywords:** Fluorinated polymers, polyesters, ring‐opening copolymerisation

## Abstract

Fluorination of polymers is a powerful strategy to enhance chemical or material properties yet integrating these benefits into degradable polymers remains underexplored. Here, we report a new class of fluorinated polyesters synthesized via ring‐opening copolymerisation of pentafluoro styrene oxide with phthalic anhydride. The pendant C_6_F_5_ groups accelerate catalysis through fluorine‐specific π‐stacking interactions and improve obtained molecular weights compared to the non‐fluorinated variant giving access to high weight materials (*M*_n,max._ > 100 kg mol^−1^) with thermal and mechanical properties competitive with commodity plastics. These C_6_F_5_ groups then act as reactive handles in the material for efficient post‐polymerisation modification (PPM) in solution, allowing fine‐tuning of thermal, mechanical, optical, and solubility properties. PPM can even be performed on material surfaces, films and fibres can be selectively modified without dissolution. Lastly, degradation enables quantitative recovery of fluorine centres as sodium fluoride, offering a sustainable end‐of‐life option for the incorporated fluorine. Our work demonstrates how targeted fluorination of degradable polyesters can simultaneously enhance catalysis and unlock advanced material functionality.

## Graphical abstract

A fluorinated polyester is introduced via ring‐opening copolymerisation of pentafluorostyrene oxide and phthalic anhydride. Pendant C_6_F_5_ groups accelerate catalysis through fluorine‐specific interactions, enabling access to high‐molecular‐weight materials. These groups serve as reactive handles for efficient post‐polymerisation and surface modification, allowing property tuning while preserving degradability and enabling fluoride recovery.

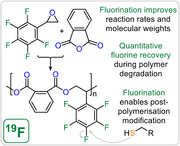

## Introduction

Polyesters are appealing degradable polymers due to the susceptibility of their ester linkages to cleavage making them useful for the chemical recycling of polymer waste into monomers or other useful starting materials.^[^ ^1^, ^2^, ^3^, ^4^, ^5^, ^6^, ^7^, ^8^, ^9^, ^10^, ^11^, ^12^, ^13^, ^14^ ^]^ Unfortunately though, in comparison to polymers with carbon‐based backbones, structural versatility, and synthetic strategies as well as properties of polyesters lag behind. Synthetic limitation affects both the range of polymer sequences accessible through synthesis and the potential for post‐polymerization modification (PPM). PPM, in particular, is a promising strategy for introducing structural complexity into polymers, as it allows the installation of functional groups after synthesis.^[^ ^15^, ^16^, ^17^, ^18^, ^19^, ^20^, ^21^, ^22^ ^]^ This approach circumvents the need for these groups to be compatible with the polymerization methodology, which can be particularly problematic for polyesters often synthesised via anionic ring‐opening polymerisation. One of the most established PPM methodologies to achieve this for oxygenated polymers is thiol‐ene click chemistry, although the required double bonds can on occasion lead to unwanted crosslinking during processing.^[^ ^23^, ^24^, ^25^, ^26^ ^]^ In the area of more traditional polymers with non‐degradable carbon–carbon backbones, improving polymer properties and enabling PPM can be achieved by fluorination.^[^ ^17^, ^18^, ^27^, ^28^, ^29^, ^30^ ^]^ Additionally, during synthesis and catalysis, fluorine‐specific interactions with electron deficient fluorine centres can lead to beneficial performances.^[^ ^31^, ^32^, ^33^, ^34^, ^35^ ^]^ However, fluorinated polymers have come under scrutiny for their lack of appreciable degradability and the absence of end‐of‐life recyclability options. Moving to polymers with intrinsically degradable backbones, such as polyesters, could present a solution offering a more direct access to end‐of‐life degradability and recyclability that are highly sought after for fluorinated materials.^[^ ^36^, ^37^, ^38^, ^39^, ^40^, ^41^, ^42^ ^]^ Despite this, fluorination of polyesters has received little attention perhaps due to difficulties with respect to their synthesis. Here ring‐opening copolymerisation (ROCOP) of strained heterocycles such as epoxides with anhydrides or heteroallenes offers access to polymer structures, including polyesters, which are hard to obtain otherwise.^[^ ^43^, ^44^, ^45^, ^46^, ^47^, ^48^, ^49^, ^50^, ^51^, ^52^, ^53^, ^54^, ^55^, ^56^, ^57^, ^58^ ^]^ In answer to these questions, we here report a new degradable fluorinated polyester with competitive properties to commodity materials featuring pendant C_6_F_5_ groups that accelerate catalysis and enable PPM of the bulk as well as the surface of processed objects. Upon degradation fluorine can be recovered in a once again useful form.

## Results and Discussion

**Figure 1:**
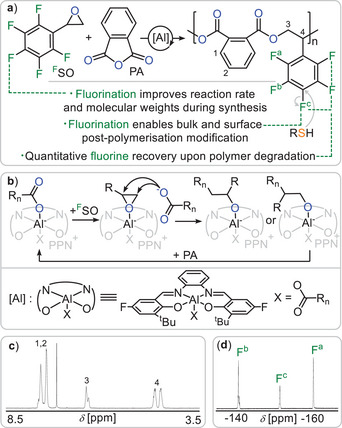
a) Outline of the current work; only head‐to‐tail regioisomer of repeat unit shown for simplicity. b) Propagation mechanism. c) ^1^H and d) ^19^F NMR spectrum (CDCl_3_) of copolymer corresponding to Table 1 entry #1. [Al] = ^F^SalphenAl(III)Cl/PPNCl with PPN = Ph_3_P = N^+^ = PPh_3_.

Starting our study, we investigated the ROCOP of pentafluoro styrene oxide (^F^SO) with phthalic anhydride (PA) (Figure 1) employing a previously optimised bicomponent ROCOP catalyst comprising of a ^F^SalphenAl(III)Cl/PPNCl catalyst pair (PPN = Ph_3_PNPPh_3_) in which Salphen is a bisphenoxy imine ligand with a phenylene backbone and *para*‐fluorinated salicyl imine.^[^ ^59^ ^]^ PA/^F^SO ROCOP at an initial loading of 1 ^F^SalphenAlCl: 1 PPNCl: 500 ^F^SO: 500 PA at 80 °C for 3 h indeed resulted in a highly viscous mixture from which the poly(PA‐*co*‐^F^SO) polymer can be isolated via precipitation (see Table 1 run #1). Multinuclear 1D and 2D NMR analysis shows selective formation of a strictly alternating polyester with pendant C_6_F_5_ groups (see Supporting Information Section S3) formed by alternating insertion of ^F^SO and PA (see Figure 1a,b). The ^1^H NMR (see Figure 1c) exhibits arylic resonances around 7.6 ppm, a tertiary CH resonance at 6.5 ppm and a secondary CH_2_ resonance at 4.7 ppm in a 4:1:2 integrative ratio.

**Table 1:** ROCOPs investigated in this report.

Run	PA:^F^SO	t (h)	Conv. (%)^a)^	*M_n, GPC_* (kg mol^−1^) (*Đ*)^b)^
#1	500:500	3	95	42.7 (1.37)
#2	50:50	0.25	>99	5.8 (1.12)
#3	100:100	0.5	95	9.3 (1.16)
#4	250:250	1	94	23.3 (1.39)
#5	2000:2000	12	92	107.3 (1.66)
#6^§)^	500:500	15	>99	10.0 (1.33)

*T* = 80 °C, equivalents of monomers relative to 1 eq. ^F^SalphenAlCl and 1 eq. PPNCl: 500 eq. epoxide: 500 eq. PA.

^a)^ Relative integral in the normalised ^1^H NMR spectrum of resonances from polymer versus unconsumed monomer.

^b)^ Apparent number averaged molecular weight determined by GPC (gel permeation chromatography) measurements conducted in THF, using narrow polystyrene standards to calibrate the instrument.

^§)^ SO was employed in place of ^F^SO.

The CH resonance correlates to a quaternary ester carbonyl resonance showing three distinct resonances at 165.7, 166.1, and 166.5 ppm (see Figure S6). These correlate to head‐to‐head, head‐to‐tail, and tail‐to‐tail diads in a 1:4.5:1 ratio by integration of the ^13^C NMR signals (see Figure S6) meaning that ^F^SO monomers are both opened at the CH_2_ tail and the CH head position.^[^ ^60^, ^61^ ^]^ However, no resonances of erroneous ether links from ^F^SO homopropagation can be observed. Accordingly, mass spectrometry (see Figure S21) of a low molecular weight sample shows series of peaks separated by 358 a.u. (i.e., the weight of PA+^F^SO). However, the circumstance that multiple series are observed indicates co‐occurring chain‐transfer and transesterification alongside propagation.^[^ ^62^, ^63^ ^]^ Accordingly, gel permeation chromatography (GPC) in THF relative to a polystyrene standard shows a multimodal distribution with apparent number averaged molecular weight *M*_n,GPC_ = 42.7 kg mol^−1^ (*Đ* = 1.37). This is somewhat lower than the theoretically expected molecular weights (*M*_n,theo _= 89.5 kg mol^−1^), which can be attributed to chain‐transfer reactions with protic impurities in the monomers, as commonly observed in ROCOP.^[^ ^43^ ^]^ Accordingly deliberate addition of diols to the initial polymerisation mixture leads to a drastic reduction of the obtained molecular weights (see Figure S20).

Furthermore, MALDI‐TOF mass spectrometry reveals that, in addition to the α‐Cl, ω‐OH terminated chains formed via initial ring‐opening of the epoxide by the chlorides of ^F^SalphenAl(III)Cl and PPNCl, cyclic polymers also form. These arise from transesterification reactions in which alkoxide intermediates dissociate from the catalyst and react with ester linkages instead of anhydride monomers. Transesterification likewise causes deviations between theoretical and experimentally obtained molecular weights and leads to broader molecular weight distributions. We confirmed this by performing a ROCOP with excess epoxide, which results in the accumulation of alkoxides after anhydride consumption and a corresponding broadening of the molecular weight distribution (see Figure S19).

Adjusting the catalyst to monomer loading lets us control the obtained molecular weights from 5.8 to 107.3 kg mol^−1^ (*Đ* = 1.12–1.66) (see Table 1 run #1). Such high maximum molecular weights are unusual given that non‐fluorinated styrene oxide (SO) is a notoriously problematic epoxide in ROCOP typically only yielding low molecular weight materials with *M*_n,GPC_ < 15 kg mol^−1^.^[^ ^64^, ^65^, ^66^, ^67^, ^68^, ^69^ ^]^ Accordingly employing SO in place of ^F^SO under otherwise identical conditions (compare Table 1 run #1 to #6) results in less than a quarter of the apparent molecular weight. On the one hand the apparent weight increase fluorination leads to could be a consequence of fewer protic impurities in ^F^SO compared to SO which cause less chain‐transfer side reactions that decrease weights in the fluorinated case. On the other hand, there might also be mechanistic differences due to fluorination regarding the propagating species.

**Figure 2:**
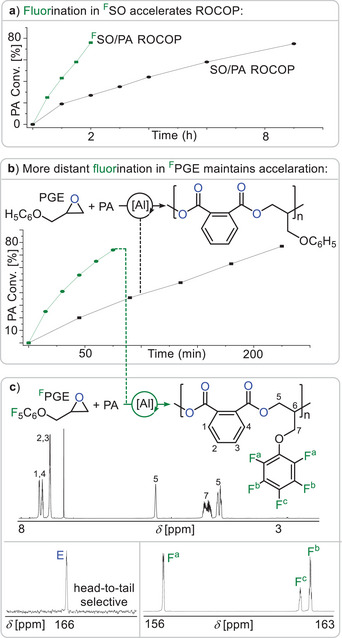
a) Comparison of PA Conversion versus time for PA/^F^SO and PA/SO ROCOP and b) for PA/^F^PGE and PA/PGE ROCOP; all at 1 eq. ^F^SalphenAlCl and 1 eq. PPNCl: 500 eq. epoxide: 500 eq. PA. c) ^1^H and ^19^F NMR (CDCl_3_) of PA/^F^PGE copolymer as well as zoom into the ^13^C NMR confirming head‐to‐tail selectivity.

As can be seen in Figure 2a employing SO in place of ^F^SO under otherwise identical conditions results in an at least threefold decrease of the reaction rate following the polymerisation progress by ^1^H aliquot analysis. In order to exclude that rate enhancement is only due to the electron withdrawing nature of fluorination close to the site of epoxide ring opening, we compared the PA ROCOP of fluorinated phenyl glycidyl ether (^F^PGE) to phenyl glycidyl ether (PGE) (see Figure 2b). Here the aromatic ring is not in close electronic communication to the electrophilic reaction site of epoxide ring‐opening. Nevertheless, we observed a maintenance of rate enhancement upon fluorination meaning that ^F^PGE polymerises three times as fast as PGE. An alternating copolymer in perfect head‐to‐tail selectivity forms with *M*_n,GPC_ = 41.7 kg mol^−1^ (*Đ =* 1.18). Notably the unfluorinated PGE again yields reduced molecular weights under otherwise analogous conditions (*M*_n,GPC_ = 17.1 kg mol^−1^, *Đ =* 1.73). Importantly in order to form a head‐to‐tail selective structure, ring opening of the epoxide must selectively occur at the CH_2_‐tail position of ^F^PGE which is most distant from the C_6_F_5_ group. Finally, we compared the ROCOP kinetics of PA with 1,2‐epoxypropan (propylene oxide) versus PA with trifluoro‐1,2‐epoxypropan to study the effects of fluorination in absence of a π‐system on the epoxide (see Figure S65). However, a much more modest acceleration by a factor of ca. 1.4 was observed for the fluorinated case. Taken together, our observations indicate that the rate enhancement cannot be merely due to favouring epoxide ring opening by rendering the tail‐position of the epoxide more electrophilic.

In order to shed light on the performance benefits that fluorination brings about we turned to density functional theory (wB97M‐V/def2‐TZVPP//PBEh‐3c//COSMO‐RS(styrene oxide)) using ORCA version 5.0 (see computational details in Section S11).^[^ ^70^, ^71^, ^72^, ^73^, ^74^ ^]^ We considered a previously established mode of action by the catalyst in which hexacoordinate aluminate centres form with two chain‐ends (one initiated by the chloride coligand of the precatalyst and the other one by the chloride of PPNCl).^[^ ^75^, ^76^ ^]^ Propagation occurs at one side of the catalyst plane, while the other is coordinated by acetate, as a model for the carboxylate resting state of the propagating chain end.

For computational efficiency ^F^SO ROCOP was investigated and the pendant polymer chain was simplified by a methyl group and in all optimised geometries. Section S11 discusses the full propagation cycle for the fluorinated and non‐fluorinated case which are both viable from a thermodynamic perspective in agreement with prior reports. Yet in order to understand rate enhancement we analysed the respective transition states of PA and ^(F)^SO insertion. For these we considered ring‐opening at the epoxide CH_2_‐tail position which minimises the effects electrophilicity enhancement of the epoxide upon fluorination. Furthermore, attack at the CH_2_‐tail position is the mode of propagation for ^F^PGE, for which fluorination likewise accelerates polymerisation.

**Figure 3:**
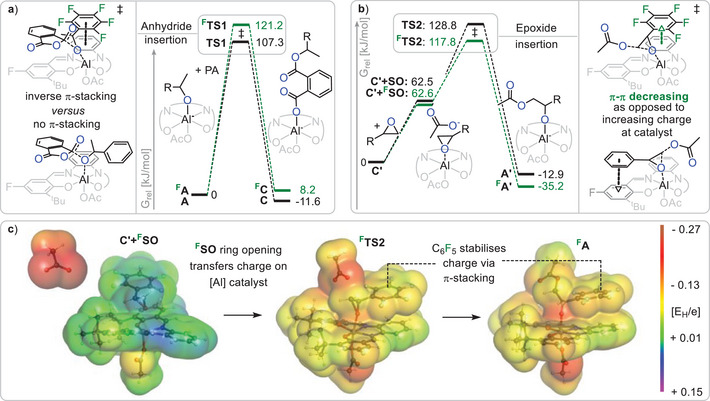
Density functional theory calculations on the wB97M‐V/def2‐TZVPP//PBEh‐3c//COSMO‐RS(styrene oxide) level of theory investigating the effect of epoxide fluorination on a) anhydride and b) epoxide insertion. c) Electrostatic potentials mapped onto an isosurface corresponding to an electron density of 0.002 e/$a_{0}^{3}$.

Considering PA insertion (see Figure 3a) starting from the alkoxide intermediate **^F^A** (stemming from ^F^SO ring opening) or **A** (stemming from SO ring opening) yielding the carboxylate **^F^C** and **C**, the most favourable transition states **^F^TS1** and **TS1** that were identified involve a parallel orientation of the PA to the ligand plane with the inserting alkoxide remaining coordinated at the aluminate centre. In the fluorinated case **^F^TS1** a parallel orientation of the C_6_F_5_ plane with the ligand plane can be observed resulting in π‐stacking primarily with the phenylene backbone of the catalyst. In contrast to the energetically more facile non‐fluorinated case **TS1** the C_6_H_5_ substituents twists away from parallel orientation so that no π‐stacking is observed altogether.

Next, we turned our attention to the epoxide ring‐opening step. Starting from **C’**, in which both carboxylates have been simplified as acetates, substitution of one carboxylate by an epoxide has to occur prior to ring opening giving **C’+^F^SO** and **C’+SO** which are very similar in energy (see Figure 3(b)). Afterward epoxide insertion in the fluorinated case **^F^TS2** (*G* ^‡^ = 117.8 kJ mol^−1^ versus **C’+^F^SO**) is easier than in the non‐fluorinated case **TS2** (*G* ^‡^ = 128.8 kJ mol^−1^ versus **C’+SO**). Hence considering all insertion barriers which have to be overcome, DFT confirms that ROCOP is faster in the fluorinated case. Interestingly during epoxide ring‐opening, π‐stacking between the epoxide substituent is observed in both cases. While the C_6_F_5_ substituent in **^F^TS2** interacts with the phenylene backbone, the C_6_H_5_ substituent in **TS2** interaction with the aromatic system of the salcylimine. Importantly epoxide ring opening by the carboxylate in **TS2** and **^F^TS2** triggers a charge redistribution through the ring‐opened styrene oxide to the aluminium atom, resulting in full charge delocalization within the complex. Figure 3c exemplarily shows the electrostatic potential maps of the fluorinated pathway. As charge redistribution occurs, **^F^TS2** displays stronger electrostatic interactions between negatively charged fluorine atoms and slightly positive hydrogen atoms than what is observed for **TS2**. This leads to an emerging anion –π interaction in the π‐stacking stabilising the accumulating charge on the catalyst during epoxide ring opening and thereby increasing the reaction rate. In contrast for **TS1** no charge accumulation at the catalyst occurs so that this effect does not lead to stabilisation of the transition state.

However, these subtle energetic differences are at the limit of what the computational method can reliably determine. In addition, the simplifications of the polymer chain growing from the catalyst limit the comparability of epoxide versus anhydride insertion steps and also preclude the elucidation of potential stabilising π‐stacking interactions between the chain end and the growing polymer chain. A more complete picture would also require assessment of interactions with the PPN countercation as well as explicitly modelled interactions with residual monomers in the surrounding medium. Nevertheless, our experimental and computational results consistently identify fluorine‐specific π‐stacking as an active contributor to the reaction kinetics. Such interactions could also influence the extent of transesterification occurring alongside propagation, as favourable π interactions limit dissociation of the chain ends from the catalyst that leads to side reactions. This might explain the differences in molecular weights observed for PA/^F^SO and PA/SO ROCOP. Hence, future strategies should focus on strengthening these interactions—e.g., by employing π‐electron‐rich ligand scaffolds—in addition to pursuing multifunctional catalysis and investigating monomer purity to maximise molecular weights.

**Figure 4:**
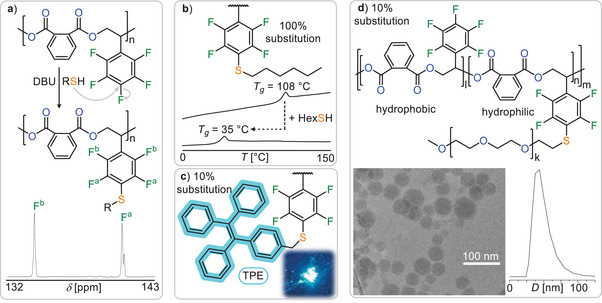
a) Post polymerisation modification (PPM) via nucleophilic aromatic substitution with ^19^F NMR spectrum after complete PPM with hexanethiol. b) Comparison of the differential scanning calorimetry (DSC) heating curve before and after PPM with hexanethiol. c) Picture of powdered polymer under UV light after PPM with blue emissive fluorophore tetraphenylethylene (TPE). d) PPM with PEG‐SH enables self‐assembly into micellar aggregates in water as seen by Cryo‐TEM and DLS.

Having accessed and understood PA/^F^SO ROCOP we turned to using the C_6_F_5_ substituent in PPM which was previously utilised for polymers with carbon–carbon backbones but remains to be realised for polyesters.^[^ ^30^, ^77^, ^78^ ^]^ Hence we reacted poly(PA‐*co*‐^F^SO) with hexylthiol and diazabicycloundecene (DBU) in DMF in order to achieve replacement of the para fluorine‐atom with a hexyl‐thioether chain. Indeed, reaction monitoring by ^19^F NMR spectroscopy reveals substitution in under a minute so that the initially three fluorine resonances at ‐140.6, ‐152.0, and ‐161.2 ppm in a 2:1:2 integrative ratio for the initial ─C_6_F_5_ groups transform into two resonances at ‐133.8 and ‐141.2 ppm in a 1:1 ratio corresponding to a ─C_6_F_4_SR group (see Figure 4a). ^1^H‐^13^C HSQC NMR spectroscopy confirms installation of the hexyl chain at the aromatic core as the S bonded CH_2_ group at 2.9 ppm correlates to an aromatic resonance at 115 ppm of the sulfur bonded aromatic carbon. GPC shows a ca. 90% maintenance of the apparent molecular weight confirming that the polyester backbone remains mostly intact during PPM alongside minimal basic degradation. PPM with hexane thiol drastically modulates the thermal properties shifting the glass transition temperature from *T*_g_ = 108.3 to *T*_g_ = 35.5 °C (see Figure 4b). PPM with 0.5 equiv. of hexane thiol per equivalent of repeat unit resulted in a material with an intermediate *T*_g_ = 66.4 °C (see Figure S77) highlighting how the degree of functionalisation can be used to tune thermal properties.

Relatedly we installed a fluorinated chain on the polymer, relevant for later surface modification (see below) introducing semi‐crystallinity with a broad *T*_m_ = 93.5 °C due to crystallisation of the fluorinated pendant chains (see Figure S85) as observed previously for other acrylate polymers.^[^ ^79^ ^]^ Moving to other functions we installed aggregation induced emission (AIE) fluorophors onto our polyesters. ^[^ ^80^ ^]^ Reacting tetraphenylethene (TPE) bearing a CH_2_SH group with poly(PA‐*co*‐^F^SO) resulted in bright blue luminescent polymer after PPM (Figure 4c). In this case functionalisation of ca. 10% of the reaction sites occurred for TPE‐CH_2_SH under identical PPM conditions employed for hexane thiol, quantified by the relative integrals of the ^19^F NMR signals corresponding to the functionalised ─C_6_F_4_SCH_2_TPE group and the remaining ─C_6_F_5_ group. We attribute this to the steric hindrance of the thiol which however was sufficient to impart luminescence. Installing hydrophilic PEG chains (*M*_n_ = 5 kg mol^−1^) alters the solubility properties of the material. While the parent poly(PA‐*co*‐^F^SO) is highly hydrophobic, attaching PEG chains at ca. 10% of the C_6_F_5_ groups renders the material sufficiently amphiphilic for self‐assembly in water. Nanoprecipitation into water of a 1 mg mL^−1^ THF solution after PPM with mPEG‐SH results in the formation of a stable colloidal solution that by eye exhibits a Tyndall effect. Dynamic light scattering (DLS) confirms the formation of nanoaggregates with an average diameter of 72 nm and a dispersity of 0.2. Cryo‐transmission electron microscopy (Cryo‐TEM) reveals the formation of multi‐layer core shell micelles (Figure 4d and Figures S105 and S106) with diameters ranging from 20 to 90 nm.

**Figure 5:**
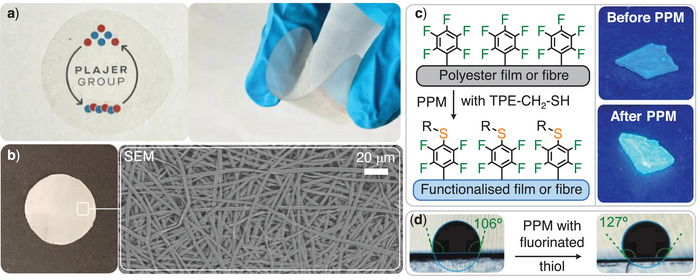
a) Photographs of compression moulded film. b) Photographs of electrospun nonwoven applied as microplastics filter and scanning electron microscopy image of the nonwoven c) Polymerisation surface modification with picture of polymer film under UV‐light before and after surface modification with TPE‐CH_2_SH fluorophore. d) Static water contact angle on surface covered with fibres before and after PPM with fluorinated thiol CF_3_(CF_2_)_5_CH_2_CH_2_SH.

Concerning properties, poly(PA‐*co*‐^F^SO) is an amorphous thermoplastic with a *T*_g_ of 108 °C due to its atactic nature. The regioselectivity has little effect on this and a polymer with a different head‐to‐head:head‐to‐tail:tail‐to‐tail ratio (prepared at higher polymerisation temperature) shows an identical *T*_g_ (see Figure S12). The wide processing window of >230 °C (*T*_d,5%_ = 340 °C) enabling compression moulding into clear, colourless, self‐standing and flexible films (*σ*_b_  = 38.67 MPa, *E*_y_ = 2.74 GPa, *ε*_b _= 1.5%, see Figure 5a and Figure S26) which feature perfect mechanical recyclability. The material possesses good solubility and hence processability from common organic solvents enabling processing by electrospinning (see Section S1). The high molecular weight material (Table 1 run #5) can be processed into polyester fibres with a homogenous thickness of between 5–10 µm (see Figure S25). This allows for the fabrication of self‐standing electrospun nonwovens (see Figure 3b) with pore sizes between 50–350 µm which renders these useful as filters for microplastics with neat quantitative efficiency. Both thermal and mechanical properties of the poly(PA‐*co*‐^F^SO), as well as filtration properties of the nonwoven fibre mat, are competitive with commodity polystyrene underlining the utility of the material.

However, as opposed to commodity polystyrene, objects comprising poly(PA‐*co*‐^F^SO) can be functionalised and degraded. Hence we investigated whether PPM can also achieve modification of the material in media in which the polymer is not soluble thereby selectively modifying the material surface. This would allow to minimise the amount of reactants required to modulate properties. To investigate this, we subjected films of poly(PA‐*co*‐^F^SO) to PPM in methanol in which the reactants for modification are soluble, but the polymer is not. Reaction of a compression moulded poly(PA‐*co*‐^F^SO) film submerged in methanol containing TPE‐CH_2_SH and DBU indeed resulted in the installation of the fluorophor on the material surface as visible under UV light (see Figure 5c). This observation was corroborated by X‐ray photoelectron spectroscopy (see Figure S97), which detected signals corresponding to the sulphur centres introduced during functionalisation. Scanning electron microscopy combined with energy‐dispersive X‐ray spectroscopy (SEM‐EDX) confirmed functionalisation across the entire surface, with an increased degree of functionalisation observed at film imperfections, presumably arising from their comparatively higher surface area generated during processing. Importantly we performed control experiments without DBU or without a thiol functionality on the TPE fluorophore and did not observe modification of the material.

**Figure 6:**
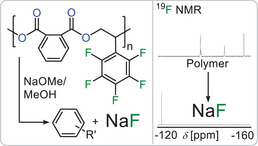
Fluoride recovery upon basic methanolysis with ^19^F NMR spectroscopy before (in CDCl_3_) and after (in D_2_O) degradation.

Surface modification is particularly attractive for electrospun polymer fibres which feature a high surface area. In this context reactive fibres are sought‐after targets in the fields of gas and air filtration, advanced textiles, microsensors, tissue engineering, and many more.^[^ ^81^ ^]^ Demonstrating the reactive nature of the fibre surface, we submerged electrospun fibres in a methanolic solution of DBU and TPE‐CH_2_SH at room temperature and indeed obtained blue, emissive fibres (see Figure S96). Yet not only the emission colour but also the surface properties of the fibres can be enhanced. Attaching a fluorinated chain CF_3_(CF_2_)_5_CH_2_CH_2_SH to the fibre surface significantly increases the hydrophobicity of a fibre covered surface. The static water contact angle increases from 106° to 127° after PPM (see Figure 6d) thereby improving hydrophobicity into the useful regime for water‐repellent coatings and self‐cleaning surfaces. Importantly ^19^F NMR spectroscopy of the dissolved fibres after PPM shows a spectrum near identical to unfunctional polyester confirming that minimal PPM on the fibre surface is necessary to achieve drastic property changes.

The intrinsic advantage of polyesters compared to polymers with carbon–carbon backbones is their in‐built opportunity for degradation and chemical recycling via cleavage of the ester bonds. Fluorinated materials have the added sustainability challenge that the fluorine centre within them stem from mined Fluorspar, a finite resource. Upon addition of sulfuric acid, Fluorspar is transformed into hydrogen fluoride HF which is then used for the synthesis of a wide range of fluorinated materials. Limited societal recycling strategies exist to recover the fluorine into a useful form, although without chemical recycling, fluorine will ultimately become scarce and expensive. In this context we recently found that the methanolysis of polyesters featuring arene bound fluorine centres allows for recovery of fluorine as degradation products comprising sodium fluoride and defluorinated organics via nucleophilic aromatic substitution. Reaction of these degradation products with sulfuric acid results in the formation of HF, the starting material of all fluorochemicals.^[^ ^82^ ^]^ Hence we subjected a Poly(PA‐*co*‐^F^SO) film to basic methanolysis employing a 5 wt% sodium methoxide solution in methanol at 110 °C for 6 h producing a finely dispersed white precipitate. ^19^F NMR analysis of the precipitate in D_2_O which can be easily isolated by centrifugation reveals clean transformation of the initially unsymmetric polymer ^19^F spectrum into a single resonance at ‐122 ppm which can be identified as sodium fluoride (see Figure 6). No fluoride resonances could be identified in the degradation products soluble in organic solvents (see Figure S108) indicating full nucleophilic aromatic substitution of the C_6_F_5_ ring and quantitative recovery of the aryl bound fluorine as NaF. Acidification of the mixture with H_2_SO_4_, in analogy of how HF is produced industrially, leads to production of hydrogen fluoride which instantaneously reacts with the glass vessel to form tetrafluoroborate as seen by ^19^F NMR (see Figure S111) Hence poly(PA‐*co*‐^F^SO) allows for chemical recycling of the bound fluorine which due to the simplicity of the reagents involved might also be suitable for upscaling. This is particularly attractive as, e.g., surface modification substitutes few fluorine centres leaving the majority to be recovered which could be confirmed in a separate experiment degrading functionalised fibres.

## Conclusion

In conclusion we have demonstrated fluorination to accelerate rates and improve molecular weights in the ring‐opening copolymerization of phthalic anhydride and pentafluoro styrene oxide. The resulting pendant C_6_F_5_ groups in the material serve as versatile handles for post‐polymerization and surface modification. These enable tuning of thermal, optical, and solubility properties and allow the fabrication of reactive electrospun fibres. Crucially, the fluorinated polymers retain the degradability of their polyester backbone, and chemical degradation facilitates the recovery of fluoride as a recyclable resource. This work highlights the potential of fluorinated degradable polymers to unite functional versatility with sustainability.

## Author Contributions

C.F.‐W. synthesised and characterised all polymer materials, recorded kinetics, performed PPM and degradation experiments which A.J.P. supervised. L.S. performed DFT calculations which B.P. supervised. C.X. performed electrospinning and filtration experiments. All authors wrote their respective parts of the supporting information. A.J.P. conceived and managed the project and furthermore wrote the manuscript which all authors jointly proof‐read.

## Conflict of Interests

The authors declare no conflict of interest.

## Supporting information



Supporting Information

## Data Availability

The data supporting this article have been included as part of the ESI.

## References

[1] S. T. R. Velasquez , Q. Hu , J. Kramm , V. C. Santin , C. Völker , F. R. Wurm , Angew. Chem. Int. Ed. 2025, 64, e202423406, 10.1002/anie.202423406. 40126932

[2] G. W. Coates , Y. D. Y. L. Getzler , Nat. Rev. Mater. 2020, 5, 501–516, 10.1038/s41578-020-0190-4.

[3] M. Häußler , M. Eck , D. Rothauer , S. Mecking , Nature 2021, 590, 423–427. 33597754 10.1038/s41586-020-03149-9

[4] C. V. Aarsen , A. Liguori , R. Mattsson , M. H. Sipponen , M. Hakkarainen , Chem. Rev. 2024, 124, 8473–8515, 10.1021/acs.chemrev.4c00032. 38936815 PMC11240263

[5] Z. Guo , H. Zhang , H. Chen , M. Zhang , X. Tang , M. Wang , D. Ma , Angew. Chem. Int. Ed. 2025, 137, e202418157, 10.1002/ange.202418157. 39491320

[6] W. Zhao , Z. Guo , J. He , Y. Zhang , Angew. Chem. Int. Ed. 2025, 137, e202420688, 10.1002/ange.202420688. 39719037

[7] Y. Hu , Y. Gu , Y. Dong , Y. Wang , J. Xu , Y. Han , C. Zhang , Y. Xie , Angew. Chem. Int. Ed. 2025, 137, e202502923, 10.1002/ange.202502923. 39953637

[8] H. Fan , C. Hu , M. Niu , Q. Zhang , B. Li , X. Pang , X. Chen , J. Am. Chem. Soc. 2025, 147, 9836–9843, 10.1021/jacs.5c00044. 40037633

[9] Y. Liu , P. Yan , X. Li , Q. Li , S. Li , H. Han , M. Chu , J. Fu , M. Cao , P. Xu , Q. Zhang , L. He , J. Chen , Adv. Mater. 2025, 37, 2412740, 10.1002/adma.202412740. 39748634

[10] C. Shi , E. C. Quinn , W. T. Diment , E. Y.‐X. Chen , Chem. Rev. 2024, 124, 4393–4478, 10.1021/acs.chemrev.3c00848. 38518259

[11] R. M. Rapagnani , R. J. Dunscomb , A. A. Fresh , I. A. Tonks , Nat. Chem. 2022, 14, 877–883, 10.1038/s41557-022-00969-2. 35760958

[12] G. X. De Hoe , M. T. Zumstein , B. J. Tiegs , J. P. Brutman , K. McNeill , M. Sander , G. W. Coates , M. A. Hillmyer , J. Am. Chem. Soc. 2018, 140, 963–973, 10.1021/jacs.7b10173. 29337538

[13] F. M. Haque , J. S. A. Ishibashi , C. A. L. Lidston , H. Shao , F. S. Bates , A. B. Chang , G. W. Coates , C. J. Cramer , P. J. Dauenhauer , W. R. Dichtel , C. J. Ellison , E. A. Gormong , L. S. Hamachi , T. R. Hoye , M. Jin , J. A. Kalow , H. J. Kim , G. Kumar , C. J. LaSalle , S. Liffland , B. M. Lipinski , Y. Pang , R. Parveen , X. Peng , Y. Popowski , E. A. Prebihalo , Y. Reddi , T. M. Reineke , D. T. Sheppard , J. L. Swartz , et al, Chem. Rev. 2022, 122, 6322–6373, 10.1021/acs.chemrev.1c00173. 35133803

[14] F. Ren , J. Xian , Z. Jia , Z. Chen , H. Fu , R. Wang , W.‐D. Chu , X. Pan , J. Wu , Angew. Chem. Int. Ed. 2023, 135, e202306759, 10.1002/ange.202306759. 37710396

[15] M. A. Gauthier , M. I. Gibson , H.‐A. Klok , Angew. Chem. Int. Ed. 2009, 48, 48–58, 10.1002/anie.200801951. 19040233

[16] S. R. Gitter , W. P. Teh , X. Yang , A. F. Dohoda , F. E. Michael , A. J. Boydston , Angew. Chem. Int. Ed. 2023, 62, e202303174, 10.1002/anie.202303174. 36943770

[17] K. A. Günay , P. Theato , H.‐A. Klok , J. Polym. Sci. : Polym. Chem. 2013, 51, 1–28.

[18] J. De Breuck , M. Streiber , M. Ringle , D. Schröder , N. Herzog , U. S. Schubert , S. Zechel , A. Traeger , M. N. Leiske , ACS Polym. Au 2024, 4, 222–234. 38882030 10.1021/acspolymersau.3c00048 PMC11177303

[19] A. P. Grimm , M. Plank , A. Stihl , C. W. Schmitt , D. Voll , F. H. Schacher , J. Lahann , P. Théato , Angew. Chem. Int. Ed. 2024, 63, e202411010, 10.1002/anie.202411010. 38895894

[20] J. F. R. Van Guyse , Y. Bernhard , A. Podevyn , R. Hoogenboom , Angew. Chem. Int. Ed. 2023, 135, e202303841, 10.1002/ange.202303841. 37335931

[21] C. Czysch , C. Medina‐Montano , Z. Zhong , A. Fuchs , J. Stickdorn , P. Winterwerber , S. Schmitt , K. Deswarte , M. Raabe , M. Scherger , F. Combes , J. De Vrieze , S. Kasmi , N. N. Sandners , S. Lienenklaus , K. Koynov , H.‐J. Räder , B. N. Lambrecht , S. A. David , M. Bros , H. Schild , S. Grabbe , B. G. De Geest , L. Nuhn , Adv. Funct. Mater. 2022, 32, 2203490, 10.1002/adfm.202203490.

[22] A. G. Heck , J. Stickdorn , L. J. Rosenberger , M. Scherger , J. Woller , K. Eigen , M. Bros , S. Grabbe , L. Nuhn , J. Am. Chem. Soc. 2023, 145, 27424–27436, 10.1021/jacs.3c08511. 38054646

[23] C. E. Hoyle , C. N. Bowman , Angew. Chem. Int. Ed. 2010, 49, 1540–1573, 10.1002/anie.200903924. 20166107

[24] O. Hauenstein , S. Agarwal , A. Greiner , Nat. Commun. 2016, 7, 11862, 10.1038/ncomms11862. 27302694 PMC4912624

[25] M. Concilio , G. S. Sulley , F. Vidal , S. Brown , C. K. Williams , J. Am. Chem. Soc. 2025, 147, 6492–6502, 10.1021/jacs.4c14032. 39949303 PMC11869290

[26] M. J. Sanford , N. J. V. Zee , G. W. Coates , Chem. Sci. 2017, 9, 134–142, 10.1039/C7SC03643D. 29629081 PMC5868299

[27] B. Ameduri , S. Fomin , Fascinating Fluoropolymers and Their Applications, Elsevier, 2020, https://www.sciencedirect.com/book/9780128218730/fascinating‐fluoropolymers‐and‐their‐applications.

[28] B. Ameduri , Chem. ‐ Eur. J. 2018, 24, 18830–18841, 10.1002/chem.201802708. 30011096

[29] Z. He , S. Li , R. Zeng , Y. Lin , Y. Zhang , Z. Hao , S. Zhang , F. Liu , Z. Tang , H. Zhong , Adv. Mater. 2024, 36, 2404824, 10.1002/adma.202404824. 38733312

[30] G. Delaittre , L. Barner , Polym. Chem. 2018, 9, 2679–2684, 10.1039/C8PY00287H.

[31] Y. Zeng , Q. Quan , P. Wen , Z. Zhang , M. Chen , Angew. Chem. Int. Ed. 2022, 61, e202215628, 10.1002/anie.202215628. 36329621

[32] K. Chen , X. Guo , M. Chen , Angew. Chem. Int. Ed. 2023, 62, e202310636, 10.1002/anie.202310636. 37581580

[33] M. Baur , F. Lin , T. O. Morgen , L. Odenwald , S. Mecking , Science 2021, 374, 604–607, 10.1126/science.abi8183. 34709904

[34] K. Livingstone , K. Siebold , S. Meyer , V. Martín‐Heras , C. G. Daniliuc , R. Gilmour , ACS Catal. 2022, 12, 14507–14516, 10.1021/acscatal.2c04511. 36504915 PMC9724094

[35] Y.‐J. Yu , M. Schäfer , C. G. Daniliuc , R. Gilmour , Angew. Chem. Int. Ed. 2023, 62, e202214906, 10.1002/anie.202214906. PMC10107283 36345795

[36] D. Li , S. Ning , L. Yu , F. Jiang , D. Zhao , S. Zhang , M. Liao , Q. Meng , Q. Fang , H. Kang , L. Li , Adv. Mater. 2025, 37, 2501622, 10.1002/adma.202501622. 40200789

[37] L. Yang , Z. Chen , C. A. Goult , T. Schlatzer , R. S. Paton , V. Gouverneur , Nature 2025, 640, 100–106, 10.1038/s41586-025-08698-5. 40140572 PMC11964924

[38] J. Gao , Z. Liu , Z. Chen , D. Rao , S. Che , C. Gu , Y. Men , J. Huang , J. Liu , Nat. Water 2023, 1, 381–390, 10.1038/s44221-023-00046-z.

[39] X. Liu , A. Sau , A. R. Green , M. V. Popescu , N. F. Pompetti , Y. Li , Y. Zhao , R. S. Paton , N. H. Damrauer , G. M. Miyake , Nature 2025, 637, 601–607, 10.1038/s41586-024-08327-7. 39566550 PMC12823158

[40] B. Trang , Y. Li , X.‐S. Xue , M. Ateia , K. N. Houk , W. R. Dichtel , Science 2022, 377, 839–845, 10.1126/science.abm8868. 35981038

[41] S. Huo , P. Zhao , Z. Shi , M. Zou , X. Yang , E. Warszawik , M. Loznik , R. Göstl , A. Herrmann , Nat. Chem. 2021, 13, 131–139, 10.1038/s41557-020-00624-8. 33514936

[42] B. Améduri , H. Hori , Chem. Soc. Rev. 2023, 52, 4208–4247. 37285132 10.1039/d2cs00763k

[43] A. J. Plajer , C. K. Williams , Angew. Chem. Int. Ed. 2022, 61, e202104495, 10.1002/anie.202104495. PMC9298364 34015162

[44] N. Jannsen , K. C. Poon , A. Craze , C. Gao , C. K. Williams , Angew. Chem. Int. Ed. 2025, 64, e202505070, 10.1002/anie.202505070. PMC12124439 40152906

[45] F. Vidal , S. Smith , C. K. Williams , J. Am. Chem. Soc. 2023, 145, 13888–13900, 10.1021/jacs.3c03261. 37311063 PMC10311538

[46] X. Lu , X. Zhang , C. Zhang , X. Zhang , Adv. Sci. 2024, 11, 2306072, 10.1002/advs.202306072.

[47] X. Zhang , X. Feng , W. Guo , C. Zhang , X. Zhang , Nat. Commun. 2024, 15, 8536, 10.1038/s41467-024-52852-y. 39358344 PMC11447067

[48] X. Zhang , W. Guo , C. Zhang , X. Zhang , Nat. Commun. 2023, 14, 5423, 10.1038/s41467-023-41136-6. 37669954 PMC10480228

[49] S. M. Severson , B.‐H. Ren , M. Cayzer , I. Keresztes , M. L. Johnson , X.‐B. Lu , G. W. Coates , J. Am. Chem. Soc. 2025, 147, 801–810, 10.1021/jacs.4c13550. 39694538

[50] X.‐Y. Fu , T.‐J. Yue , X.‐H. Guo , X.‐B. Lu , W.‐M. Ren , Nat. Commun. 2025, 16, 2154, 10.1038/s41467-025-57449-7. 40038273 PMC11880438

[51] J. Li , Y. Liu , W.‐M. Ren , X.‐B. Lu , J. Am. Chem. Soc. 2016, 138, 11493–11496, 10.1021/jacs.6b07520. 27562940

[52] Z.‐Q. Wan , W.‐M. Ren , S. Yang , M.‐R. Li , G.‐G. Gu , X.‐B. Lu , Angew. Chem. Int. Ed. 2019, 58, 17636–17640, 10.1002/anie.201910369. 31595601

[53] R. Xie , Y.‐Y. Zhang , G.‐W. Yang , X.‐F. Zhu , B. Li , G.‐P. Wu , Angew. Chem. Int. Ed. 2021, 133, 19402–19410, 10.1002/ange.202104981.

[54] R. Xie , Y. Wang , S. Li , B. Li , J. Xu , J. Liu , Y. He , G.‐W. Yang , G.‐P. Wu , Angew. Chem. Int. Ed. 2024, 136, e202404207, 10.1002/ange.202404207. 38647637

[55] Z. Xie , Z. Yang , C. Hu , F.‐Q. Bai , N. Li , Z. Wang , S. Ku , X. Pang , X. Chen , X. Wang , J. Am. Chem. Soc. 2025, 147, 12115–12126, 10.1021/jacs.5c00426. 40143535

[56] B. R. Manjunatha , K. S. Marcus , R. M. Gomila , A. Frontera , A. J. Plajer , Green Chem. 2025, 27, 3494–3502, 10.1039/D4GC05665E.

[57] Y. Ma , X. You , J. Zhang , X. Wang , X. Kou , S. Liu , R. Zhong , Z. Li , Angew. Chem. Int. Ed. 2023, 62, e202303315, 10.1002/anie.202303315. 37073925

[58] B. R. Manjunatha , C. Gallizioli , C. Fornacon‐Wood , J. Stephan , M. R. Stühler , A. J. Plajer , Angew. Chem. Int. Ed. 2025, 64, e202507243, 10.1002/anie.202507243. PMC12184296 40323177

[59] N. J. Van Zee , M. J. Sanford , G. W. Coates , J. Am. Chem. Soc. 2016, 138, 2755–2761, 10.1021/jacs.5b12888. 26883176

[60] N. D. Harrold , Y. Li , M. H. Chisholm , Macromolecules 2013, 46, 692–698, 10.1021/ma302492p.

[61] G.‐P. Wu , S.‐H. Wei , X.‐B. Lu , W.‐M. Ren , D. J. Darensbourg , Macromolecules 2010, 43, 9202–9204, 10.1021/ma1021456.

[62] D. J. Darensbourg , Green Chem. 2019, 21, 2214–2223, 10.1039/C9GC00620F.

[63] J. Xu , P. Zhang , Y. Yuan , N. Hadjichristidis , Angew. Chem. Int. Ed. 2023, 14, e202218891. 10.1002/anie.202218891 36734167

[64] S. Zhao , C.‐K. Xu , Y. Gong , G.‐W. Yang , G.‐P. Wu , Eur. Polym. J. 232, 113912, 10.1016/j.eurpolymj.2025.113912.

[65] S. Abbina , V. K. Chidara , G. Du , ChemCatChem 2017, 9, 1343–1348, 10.1002/cctc.201601679.

[66] E. H. Nejad , A. Paoniasari , C. E. Koning , R. Duchateau , Polym. Chem. 2012, 3, 1308, 10.1039/c2py20026k.

[67] H. Zhang , S. Hu , J. Zhao , G. Zhang , Eur. Polym. J. 2017, 95, 693–701, 10.1016/j.eurpolymj.2017.06.005.

[68] C. Gallizioli , D. Battke , H. Schlaad , P. Deglmann , A. J. Plajer , Angew. Chem. Int. Ed. 2024, 63, e202319810, 10.1002/anie.202319810. 38421100

[69] C. Gallizioli , P. Deglmann , A. J. Plajer , Angew. Chem. Int. Ed. 2025, 64, e202501337. 10.1002/anie.202501337 PMC12281093 40208780

[70] A. Klamt , V. Jonas , T. Bürger , J. C. W. Lohrenz , J. Phys. Chem. A 1998, 102, 5074–5085, 10.1021/jp980017s.

[71] F. Neese , WIREs 2022, 12, e1606.

[72] S. Grimme , J. G. Brandenburg , C. Bannwarth , A. Hansen , J. Chem. Phys. 2015, 143, 054107, 10.1063/1.4927476. 26254642

[73] F. Weigend , R. Ahlrichs , Phys. Chem. Chem. Phys. 2005, 7, 3297, 10.1039/b508541a. 16240044

[74] N. Mardirossian , M. Head‐Gordon , J. Chem. Phys. 2016, 144, 214110, 10.1063/1.4952647. 27276948

[75] M. E. Fieser , M. J. Sanford , L. A. Mitchell , C. R. Dunbar , M. Mandal , N. J. Van Zee , D. M. Urness , C. J. Cramer , G. W. Coates , W. B. Tolman , J. Am. Chem. Soc. 2017, 139, 15222–15231, 10.1021/jacs.7b09079. 28984455

[76] B. A. Abel , C. A. L. Lidston , G. W. Coates , J. Am. Chem. Soc. 2019, 141, 12760–12769, 10.1021/jacs.9b05570. 31380637

[77] G. Topcu , D. R. Arenas , S. Huband , T. McNally , C. R. Becer , J. Mater. Chem. C 2022, 10, 9356–9363.

[78] B. Mekonnen , D. Flahaut , A. Khoukh , L. Perrier , C. Miqueu , A. Bousquet , J. Allouche , D. Grégoire , Chem. Mater. 2025, 37, 266–277, 10.1021/acs.chemmater.4c02441.

[79] Y. Shibasaki , K. Chiba , J. Therm. Anal. 1997, 49, 115–121.

[80] Z. Zhao , H. Zhang , J. W. Y. Lam , B. Z. Tang , Angew. Chem. Int. Ed. 2020, 59, 9888–9907, 10.1002/anie.201916729. 32048428

[81] R. Merckx , V. Dhaware , M. N. Leiske , K. De Clerck , R. Hoogenboom , Chem. Mater. 2024, 36, 9189–9206, 10.1021/acs.chemmater.4c00277.

[82] C. Fornacon‐Wood , M. R. Stühler , A. Millanvois , L. Steiner , C. Weimann , D. Silbernagl , H. Sturm , B. Paulus , A. J. Plajer , Chem. Commun. 2024, 60, 7479–7482, 10.1039/D4CC02513J. 38939919

