# Supplementary material for: Recoverable Fluorination Accelerates Ring‐Opening Copolymerisation and Enables Post‐Polymerisation‐Modification of Polyesters
Source: Angew Chem Int Ed Engl. 2025 Oct 20;64(51):e202515104. doi: 10.1002/anie.202515104 (PMC12707361; doi:10.1002/anie.202515104)
Supplement: Supplementary file 1 — Supporting Information [file ANIE-64-e202515104-s001.pdf]

Supplementary Information

Table of contents

Section S1: Supplementary methods……….…………………………………….3

Section S2: Polymerisation data……………..……………………………………7

Section S3: ^{F}SO/PA copolymerisation……….……………………………………8

Section S4: ^{F}PhGE/PA copolymerisation……………………..…………………21

Section S5: SO/PA copolymerisation…………………………………………..….26

Section S6: PhGE/PA copolymerisation………………………………………….30

Section S7: FSO/PA copolymerisation……………………………………………..35

Section S7: Kinetics…………………………………………………………...………40

Section S8: Post-polymerisation modification……………………………………45

Section S10: Polymer degradation and fluoride recovery…………………………67

Section S11: Computational details…………………………………………………70

Section S12: References…………………………………………………………….132

Section S1: Supplementary methods

FSalphenAl(III)Cl catalyst was synthesised according to literature.[1] Bis(triphenylphosphine)iminium chloride (PPNCl) was recrystallized from dry dichloromethane (DCM) and dried in vacuum prior to use. Phthalic anhydride (PA) was purified according to literature.[2] Epoxides were synthesised according to the literature or obtained commercially and dried over calcium hydride and sodium hydride before distillation and storage in an argon filled glovebox.[3] Thiols used for post-functionalisation were either synthesised according to literature or obtained commercially.[4] All other reagents were used as received if not stated otherwise. Nuclear Magnetic Resonance (NMR) spectra were recorded by using a Jeol JNMECA 400II, Bruker Advance 300 and 500 MHz spectrometer. ^{1}H and ^{13}C chemical shifts are referenced to the residual proton resonance of the deuterated solvents. ^{19}F chemical shifts are referenced to $CFCl_3$ at 0 ppm. Thermogravimetric Analysis (TGA) data was measured using a Mettler Toledo STARe System ''TGA/DSC 3+''. Differential scanning calorimetry (DSC) was measured on a Mettler Toledo ''STARe System DSC 3+'' at a heating rate of 10.0 K/min. The molecular weight and polydispersity of the polymers were determined by a Waters 515 Gel permeation chromatography (GPC) instrument equipped with two linear PLgel columns (Mixed-C) following guard column and a differential refractive index detector using tetrahydrofuran as the eluent at a flow rate of 1.0 mL/min at 30 °C and a series of narrow polystyrene standards for the calibration of the columns. Each polymer sample was dissolved in HPLC-grade THF (5 mg/mL) and filtered through a 0.20 µm porous filter frit prior to analysis. MALDI-TOF spectra was recorded with a MALDI-TOF Spectrometer Bruker Autoflex Max.

General polymerisation protocol: The catalyst, cocatalyst and monomers were added to an oven dried vial equipped with an oven dried stirrer bar and sealed with a melamine cap containing a Teflon inlay inside an argon filled glovebox. The vial was brought outside the glovebox and placed in a pre-heated aluminium block at the specified temperature for the specified time. At the specified end point of the reaction, the polymerisation mixture was cooled down to room temperature and an aliquot was removed and analysed by ^{1}H and ^{19}F NMR for the determination of the conversion. The mixture was solubilized with ca. 5 ml of DCM and then added to 50 mL of MeOH causing the precipitation of the polymer which was isolated by centrifugation. This was repeated twice and the polymer was then dried in a vacuum oven set to 50 °C for 2 h.

Polymer films were made using a Weber Hot press at 140°C with 2 metric tonnes of pressure for 30 min, after that the plates were left to cool slowly while keeping the pressure to ensure flat films and avoid any bending or cracking due to inhomogeneous cooling.

Tensile tests were performed on an Instron 5565 universal tester using a 1 kN load cell and pneumatic clamps. The thickness of the specimens was measured with a digital micrometre (Mitutoyo 293–831, digimatic MDC Lite) in the range of 600 to 650 µm. The E-Moduli of all specimens were investigated at a strain rate of 0.2 mm min^{-1} and calculated between 0.1 % and 0.3 % strain. To ensure a better evaluation of the properties, the measure was conducted on 5 dog bones cut from the same film.

XPS was recorded using a PHI 5000 Versaprobe III instrument. SEM-EDX was recorded using a Zeiss Leo 1530 equipped with a UltraDry-EDX-Detector.

For electrospinning of the polymer, 1.20 g of FSO/PA copolymer (table S2, entry 2) (26 wt.%) was dissolved in a solvent mixture containing 2.90 g chloroform ($CHCl_3$, 62.9 wt.%) and 0.51 g dimethylformamide (DMF, 11.1 wt.%). This solution was filled in a plastic syringe (Injekt, 2 mL, B. Braun) assembled with a blunt-ended stainless steel needle (Sterican, Ø = 0.8 mm, B. Braun). A custom-made electrospinning device was used for the electrospinning of nonwovens.[5] The following conditions were applied for electrospinning: voltage 20 kV, flow

rate 2 mL/h, and the distance between needle and collector 15 cm with relative humidity ~40 % and temperature ~22 °C. The electrospun nonwoven was collected on a flat collector covered by aluminum foil connected to −2 kV negative voltage.

Filtration Experiments - Nonwoven commercial polystyrene (average Mw = 192000, Sigma Aldrich, Germany) was prepared by dissolving 1.10 g polystyrene in 3.90 g DMF. Electrospinning was performed under the following conditions: applied voltage 20 kV, flow rate 1.5 mL/h, and the distance between needle and collector 15 cm.

The water filtration was done using a round shape nonwoven (polymer table S2, entry 2). The pore size distribution of the electrospun nonwoven was determined by statistical analysis of 100 pores using ImageJ. A circular nonwoven with a diameter of 25 mm was cut from the electrospun mat using scissors. The filter was then fixed in a Sartorius filtration apparatus (Sartorius AG), providing an effective filtration area of 3.14 cm^2. A total of 40 mL of polystyrene aqueous suspension (100 µg/mL, particle size ranging from 200 to 500 nm) was used as the feed solution. The suspension was loaded in a 50 mL-syringe (B. Braun, Switzerland), which was fixed on a LA100 syringe pump (Landgraf Laborsysteme). The flow rate was set to 1 mL/min using the syringe pump. The transmembrane pressure was recorded every minute using a digital manometer (digi-04). Membrane permeability was calculated using the following equation:

$$Permeability\ \left(L\cdot m^{-2}\cdot h^{-1}\cdot bar^{-1}\right) = \frac{V}{A\times\Delta t\times\Delta p}$$

Where V is the volume of filtrate, A is the filtration area, Δt is the filtration time, and Δp is the transmembrane pressure. The filtrate was collected every 5 min to calculate the filtration efficiency. The filtration efficiency was determined by measuring the residual polystyrene particle concentration using UV-Vis (Agilent 8453 diode-array spectrophotometer). The same experiment was conducted using an electrospun polystyrene nonwoven as a benchmark.

The filtration performance of electrospun polymer high nonwoven (ES-^{F}SO/PA) was based on its ability to remove polystyrene particles from suspension. The water filtration test was performed using a round, self-standing ES- ^{F}SO/PA (Figure S1), with an electrospun polystyrene nonwoven (ES-PS) used as benchmark. The average filtration efficiency of ES-^{F}SO/PA (80.4%) was lower than that of ES-PS (98.9%). The higher efficiency of PS-ES can be attributed to its smaller pore size (Figure S3a and S3b) and speculatively to stronger interactions between the polystyrene particles and the ES-PS matrix, resulting in better particle retention. However, the filtration efficiency of ES-^{F}SO/PA increased over time, reaching 99.6% at 40 minutes (Figure S4a). Meanwhile, the filtration efficiency of ES-PS showed a slight increase, ultimately reaching 100% by the end of the test. The increase in filtration efficiency can be attributed to the accumulation of polystyrene particles between the fibres, thereby clogging the pores.

Notably, the permeability of ES-^{F}SO/PA is significantly higher than that of ES-PS (Figure S3b), which is likely due to the relatively large pore size of ES-^{F}SO/PA. The large cavities between the fibres allow more fluid to pass through. This behaviour indicates depth filtration of ES-^{F}SO/PA, which is considered to be more efficient because it prevents surface clogging and increases the effective filtration area and capacity. (https://doi.org/10.1002/bit.28957).

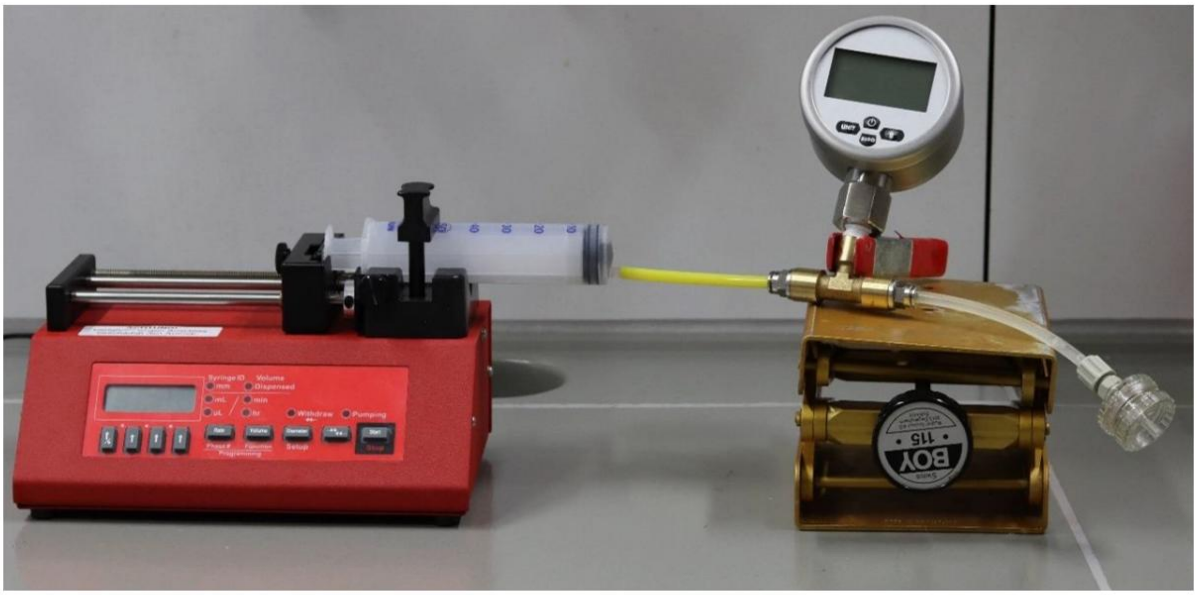

Figure S1 - Measurement setup for the aqueous filtration of PS microparticles from water with parallel measurement of the pressure drop. Figure adapted from ref. [6] under CC BY 3.0.

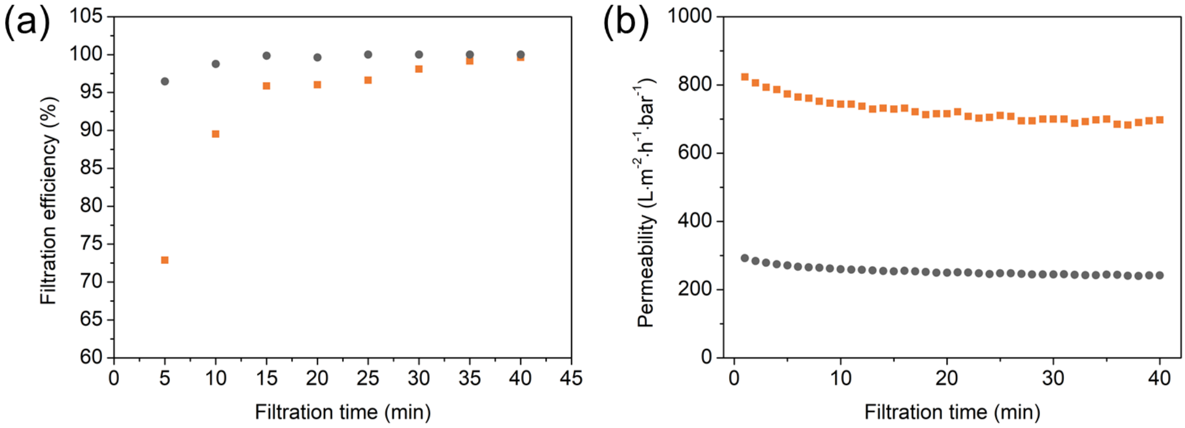

Figure S2 - (a) Filtration efficiency and (b) permeability over time for polystyrene particles removal using ES-^{F}SO/PA (orange squares) and ES-PS (grey dots).

For post-polymerisation modification the polymer (100 mg) and the thiol (1 mol. equiv.) were dissolved in DMF. 1,8-Diazabicyclo[5.4.0]undec-7-ene (DBU) (1 mol. equiv.) was added and directly after addition the mixture was crashed out into 50 mL of MeOH causing precipitation of the post-functionalised polymer which was isolated by centrifugation and dried in a vacuum oven set to 50 °C for 2 h.

Cryto-TEM images were taken using a JEOL JEM 2200FS with in-column Filter.

For surface functionalisation the polymer film/fibre was placed in a methanol solution containing the thiol and DBU (1 equiv.) for 30 mins. This was then taken out and washed with fresh methanol before being dried in a vacuum oven set to 50 °C for 2 h.

For methanolysis a 200 mg pressed film of the polymer material was added to a 5 wt.% NaOMe in methanol solution and stirred at 110 °C overnight. The product was diluted with DCM and separated from the solid by centrifugation. The solid material was used as is for further analysis.

Section S2: Polymerisation Data

Table S1 – Polymerisation Data

Entry[a]	Monomer Combination	Time (h)	% Conversion[b]	TOF (h^{-1})[c]	$M_{n\ GPC}$ ($Đ$)[d]	$M_{n\ theo}$
1	^{F}SO/PA	3	95	158	42.7 (1.20)	89.5
2	^{F}PhGE/PA	1	>99	500	41.7 (1.18)	97.6
3	SO/PA	15	>99	33	10.0 (1.33)	67.6
4	PhGE/PA	4	>99	125	17.1 (1.73)	75.1
5	^{F}PO/PA	2	92	230	23.1 (1.11)	59.8

[a]ROCOP conditions: 1 eq. Cat.:1 eq. PPNCl: 500 eq. epoxide: 500 eq. anhydride at 80 °C, [b]% Conversion calculated by comparing anhydride monomer and polymer signals in ^{1}H and ^{19}F NMR, [c]TOF measured by TON/time where TON is number of moles of monomer consumed per mole of catalyst, [d]determined by gel permeation chromatography (GPC) in THF using narrow dispersity polystyrene standards to calibrate; $Đ = M_w/M_n$.

Table S2 – Loading Data

Entry[a]	Loading[b]	Time (h)	% Conversion[c]	TOF (h^{-1})[d]	$M_{n\ GPC}$ (Đ)[e]	$M_{n\ theo}$
1	1:1:500:500	3	95	158	42.7 (1.20)	89.5
2	1:1:2000:2000	12	92	153	107.3 (1.66)	346.7
3	1:1:250:250	1	94	235	23.3 (1.39)	44.2
4	1:1:100:100	0.5	95	190	9.34 (1.16)	17.9
5	1:1:50:50	0.25	>99	200	5.8 (1.12)	9.42

[a]ROCOP conditions at 80 °C, [b]Loading corresponds to molar ratio of Cat.: PPNCl: ^{F}SO: PA, [c]% Conversion calculated by comparing anhydride monomer and polymer signals in ^{1}H and ^{19}F NMR, [d]TOF measured by TON/time where TON is number of moles of monomer consumed per mole of catalyst, [e]determined by gel permeation chromatography (GPC) in THF using narrow dispersity polystyrene standards to calibrate; $Đ = M_w/M_n$.

Section S3: ^{F}SO/PA copolymerisation

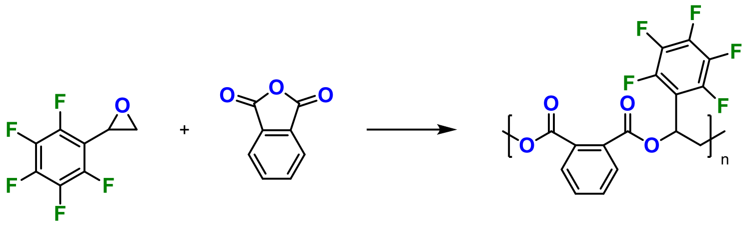

Figure S3 – Copolymerisation of ^{F}SO and PA.

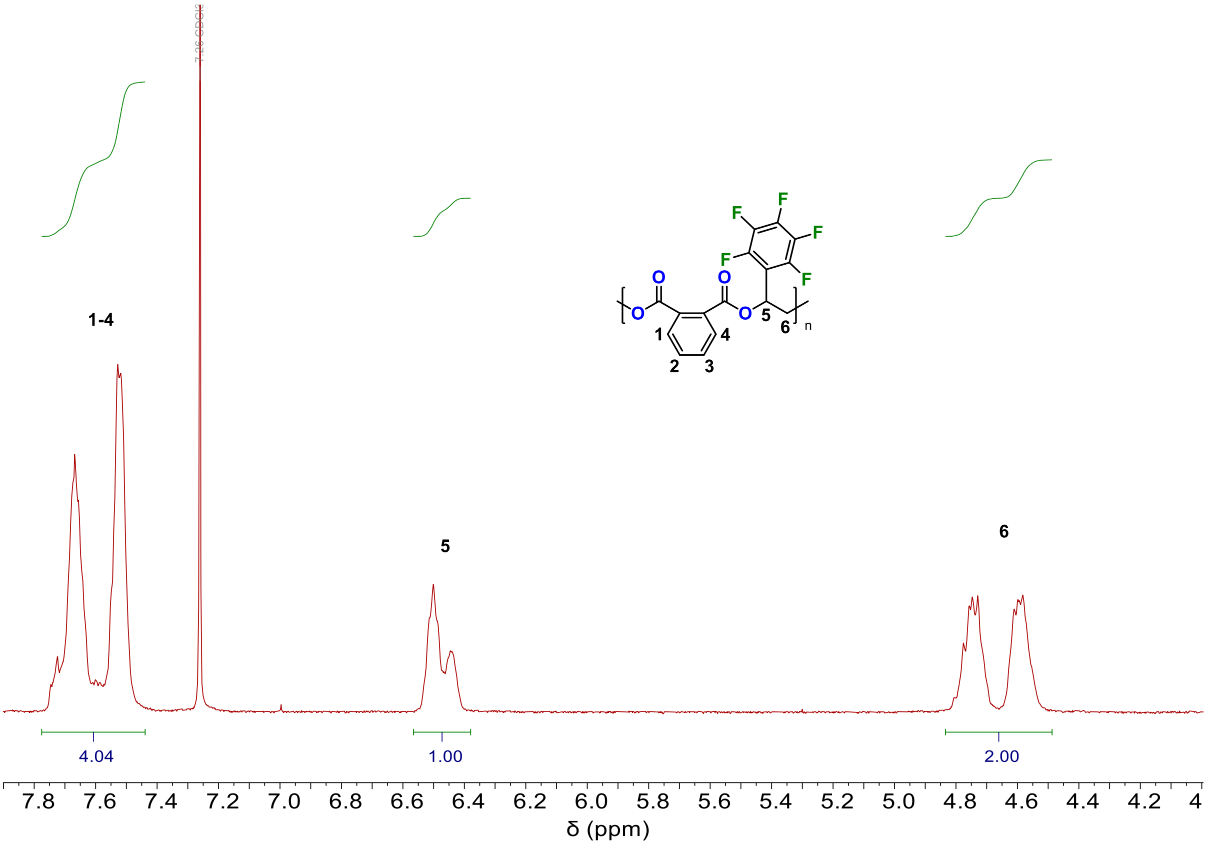

Figure S4 – ^{1}H NMR spectrum (500 MHz, $CDCl_3$) of ^{F}SO/PA copolymerisation corresponding to Table S1 entry 1.

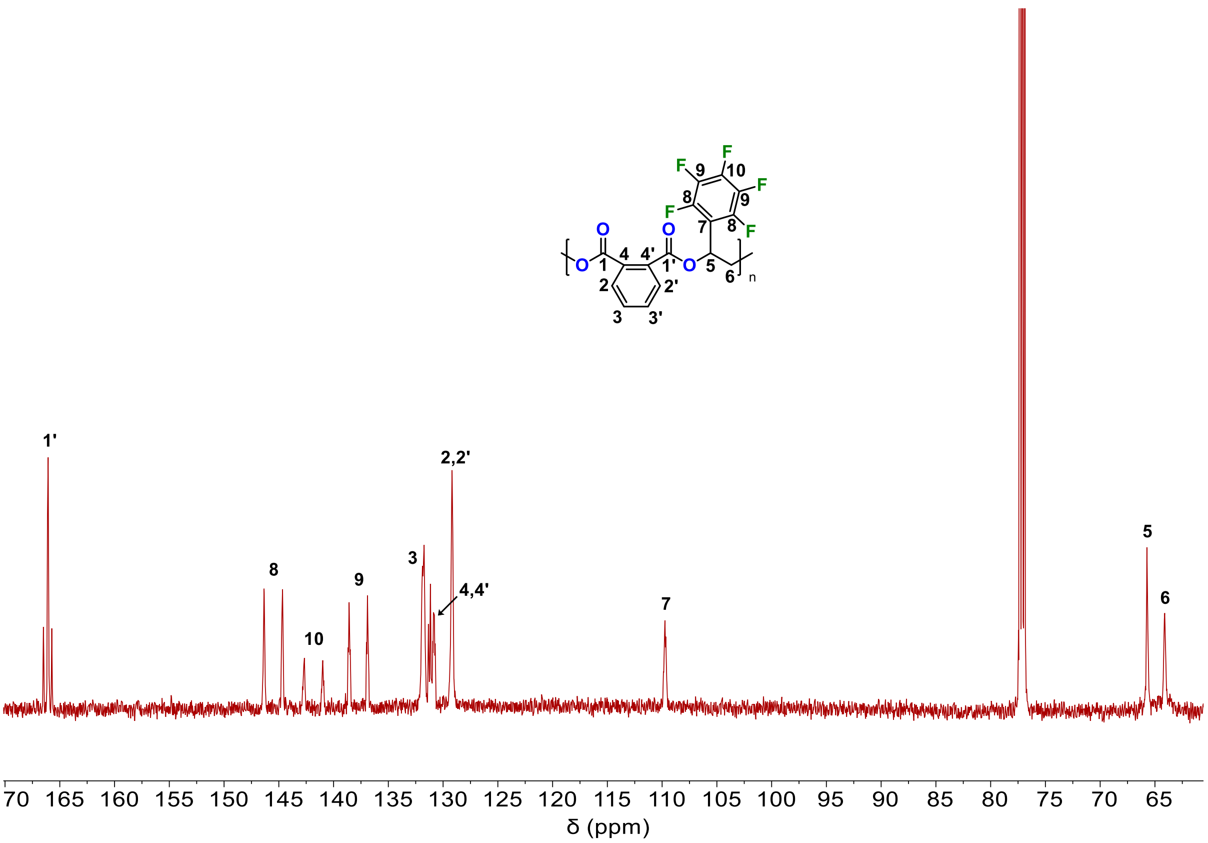

Figure S5 - ^{13}C NMR spectrum (126 MHz, CDCl$_3$) of ^{F}SO/PA copolymer corresponding to Table S1 entry 1.

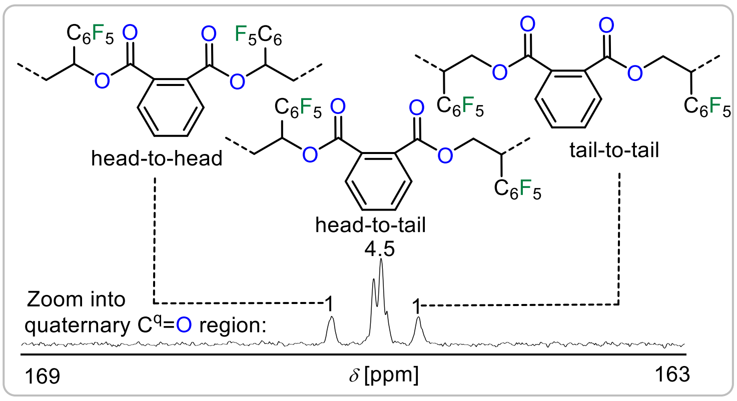

Figure S6 – Zoom into quaternary C=O region showing head-to-head, head-to-tail, and tail-to-tail polymer linkage at 80 °C.[7a]

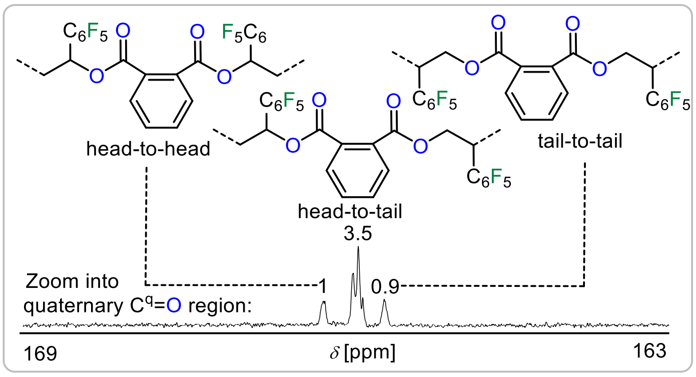

Figure S7 - Zoom into quaternary C=O region showing head-to-head, head-to-tail, and tail-to-tail polymer linkage at 120 °C.[7]

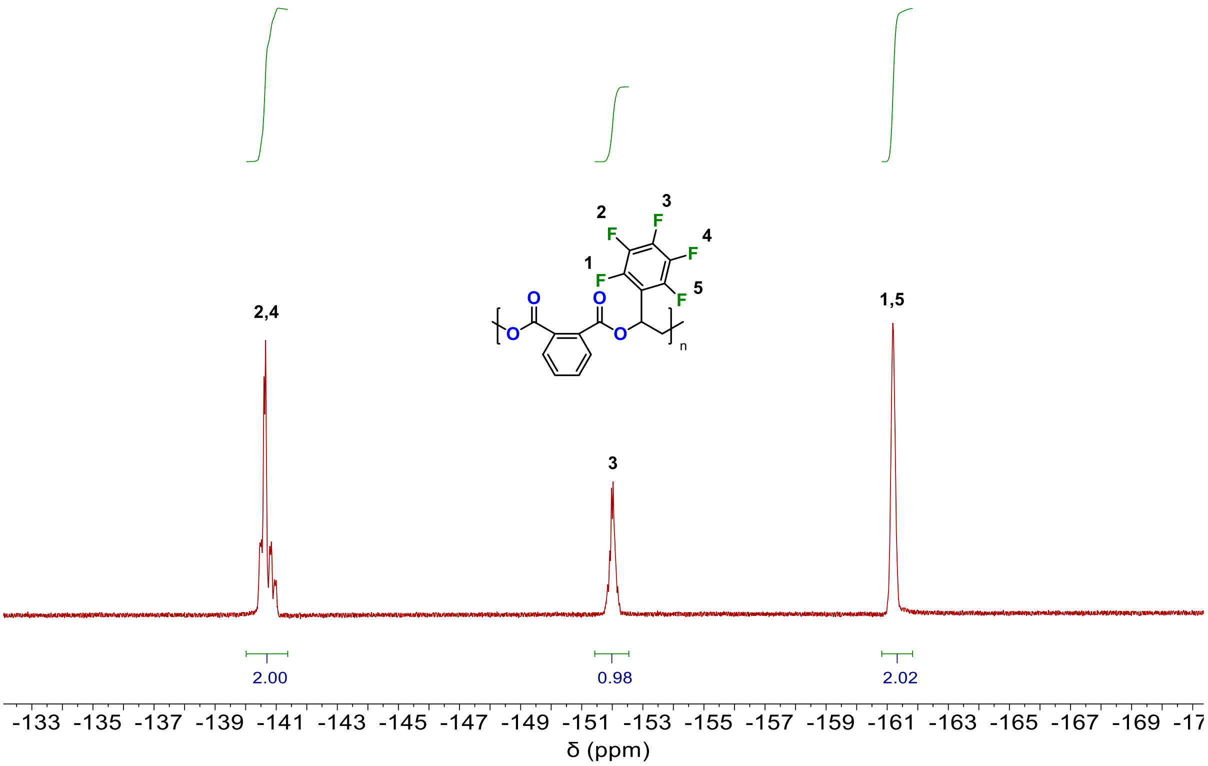

Figure S8 – ^{19}F NMR spectrum (376 MHz, $CDCl_3$) of ^{F}SO/PA copolymer corresponding to Table S1 entry 1.

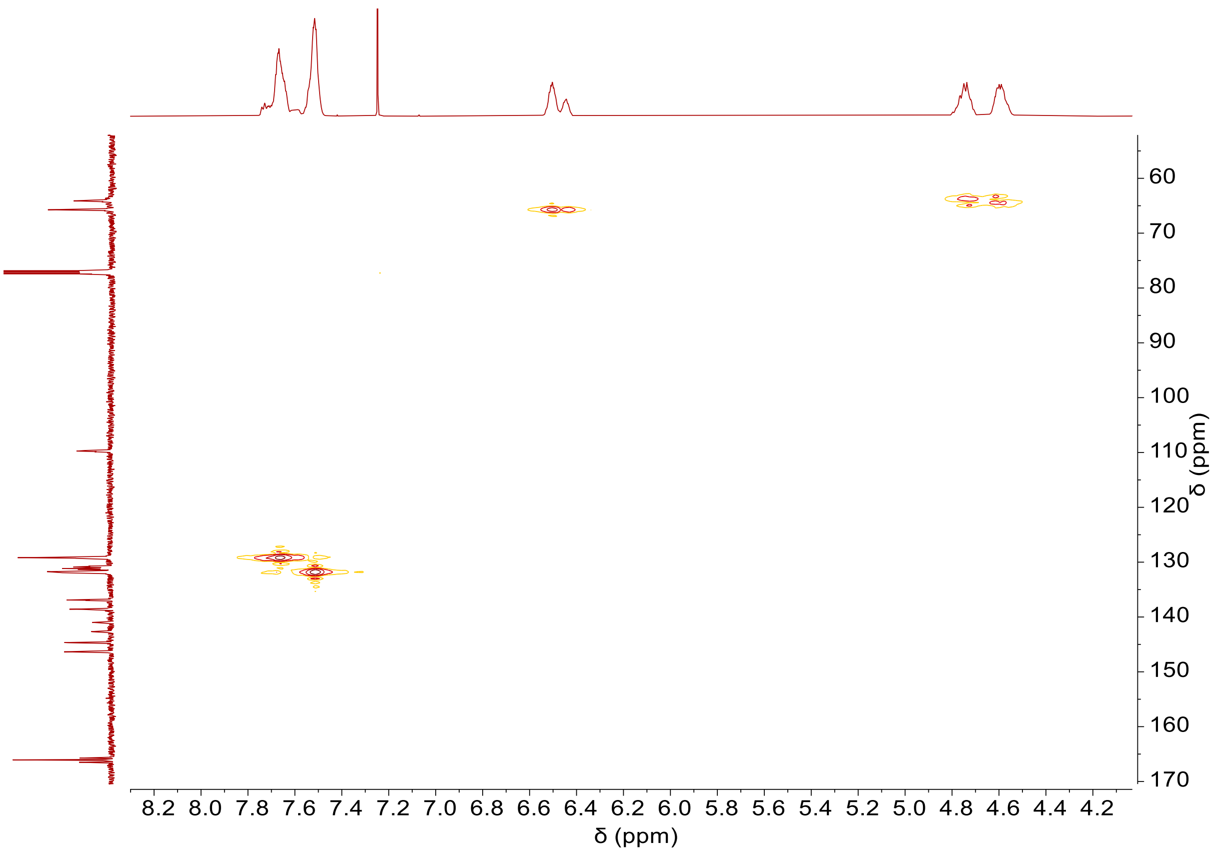

Figure S9 - ${}^{1}H$-${}^{13}C$ HSQC NMR spectrum ($CDCl_3$) spectrum of the ${}^{F}SO$/PA copolymer corresponding to Table S1 entry 1.

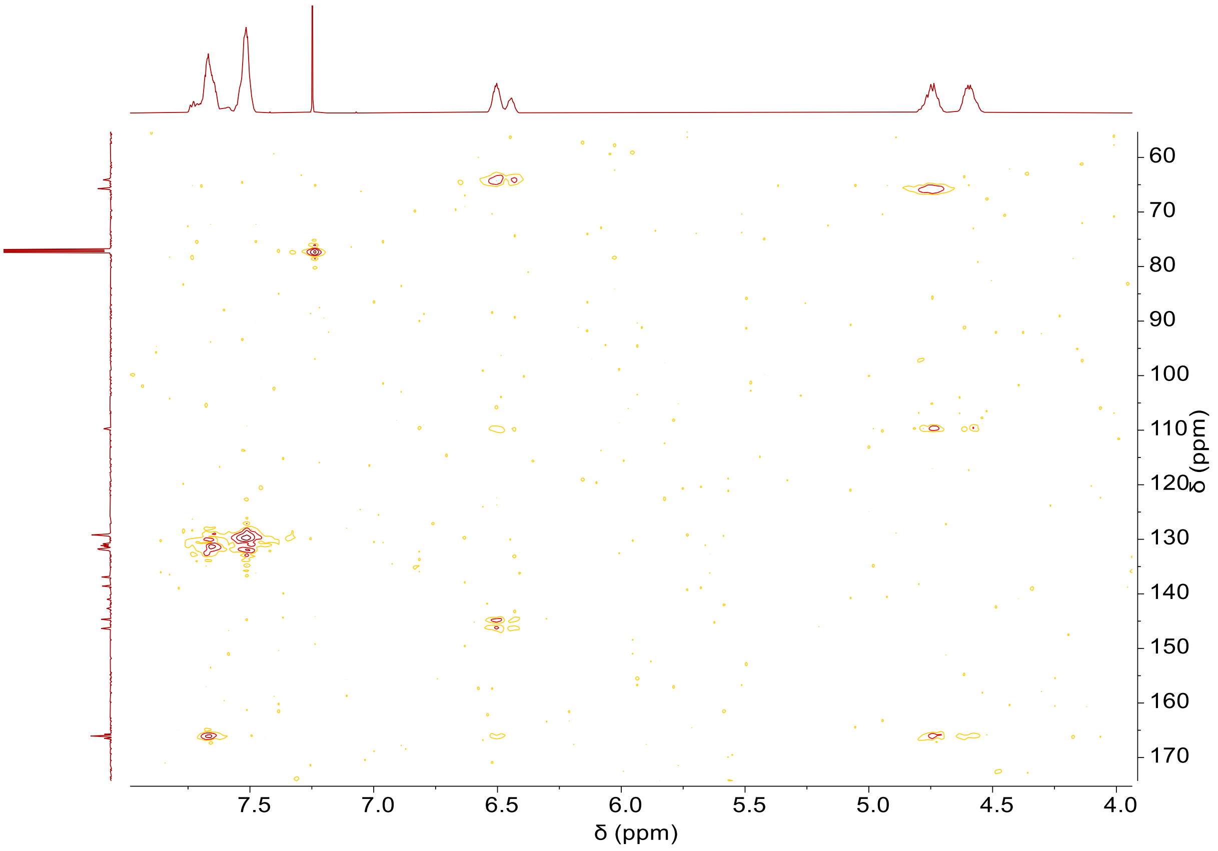

Figure S10 - ${}^{1}H$-${}^{13}C$ HMBC NMR spectrum ($CDCl_3$) spectrum of the ${}^{F}SO$/PA copolymer corresponding to Table S1 entry 1.

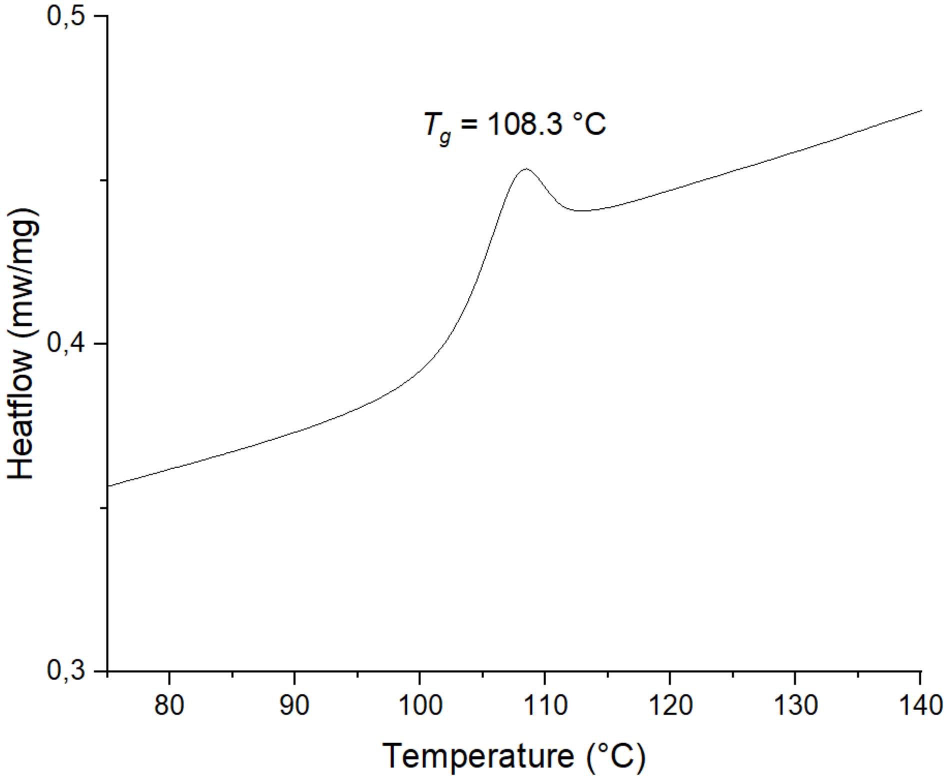

Figure S11 - DSC data from the second heating cycle of [F]SO/PA copolymer corresponding to Table S1 entry 1.

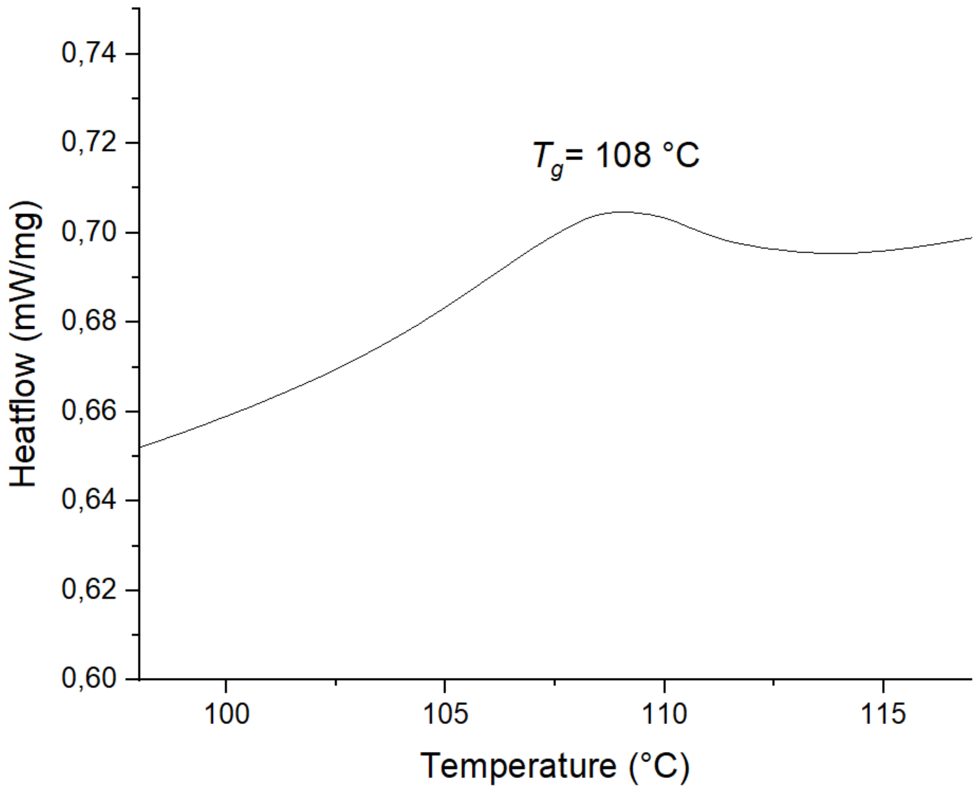

Figure S12 - DSC data from the second heating cycle corresponding to [F]SO/PA copolymer at 120 °C.

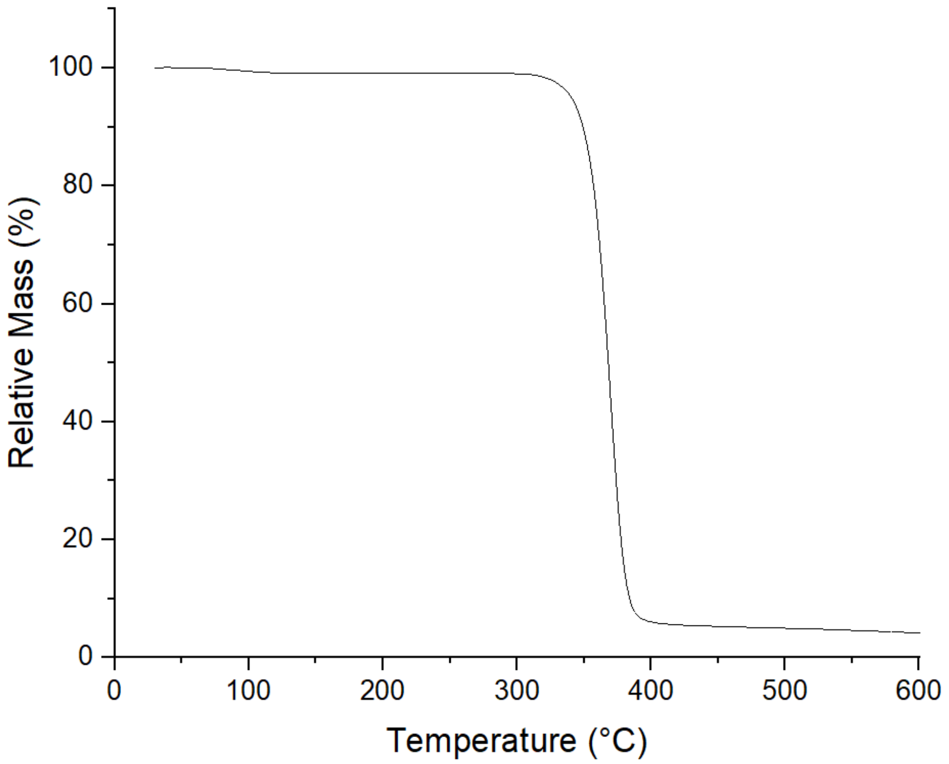

Figure S13 - TGA data of copolymer corresponding to ^{F}SO/PA copolymer corresponding to Table S1 entry 1. $T_{d,5\%}$ = 340.6 ºC.

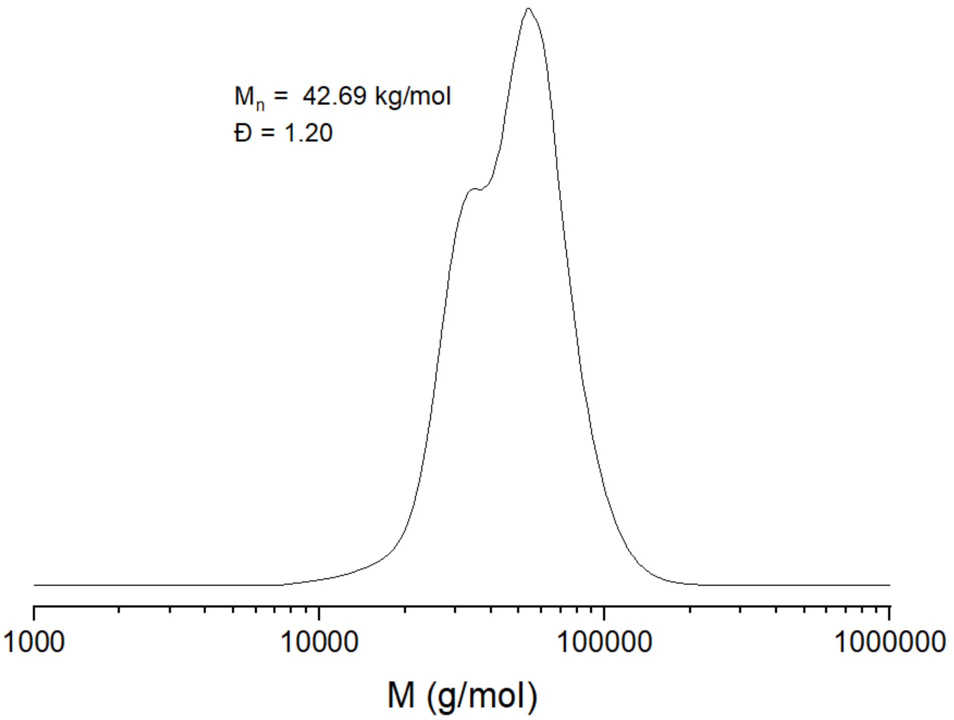

Figure S14 - GPC trace of ^{F}SO/PA copolymer corresponding to Table S2 entry 1.

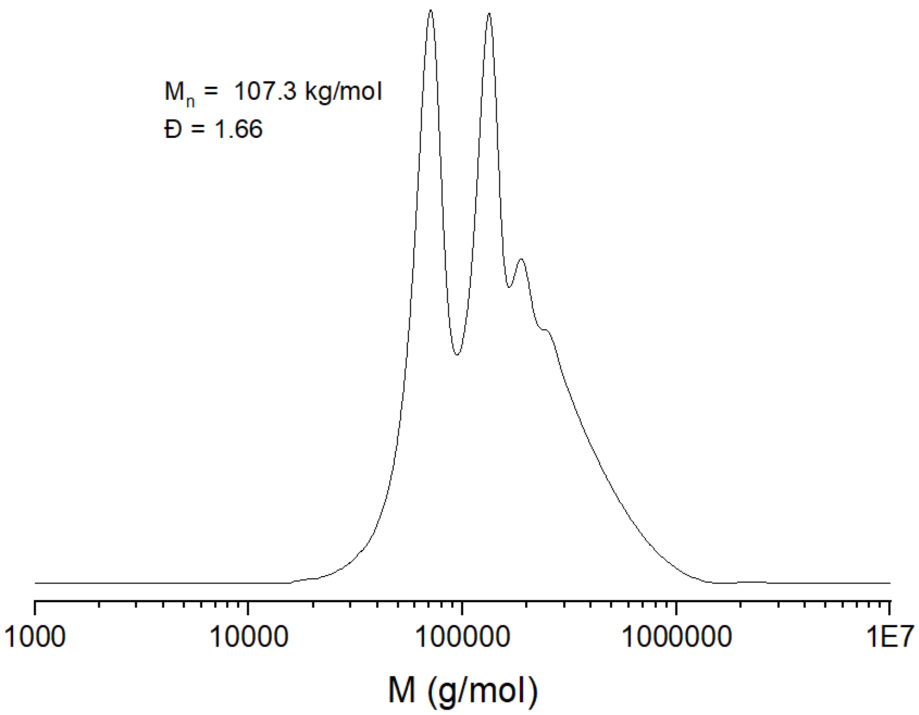

Figure S15 - GPC trace of ^{F}SO/PA copolymer corresponding to Table S2 entry 2.

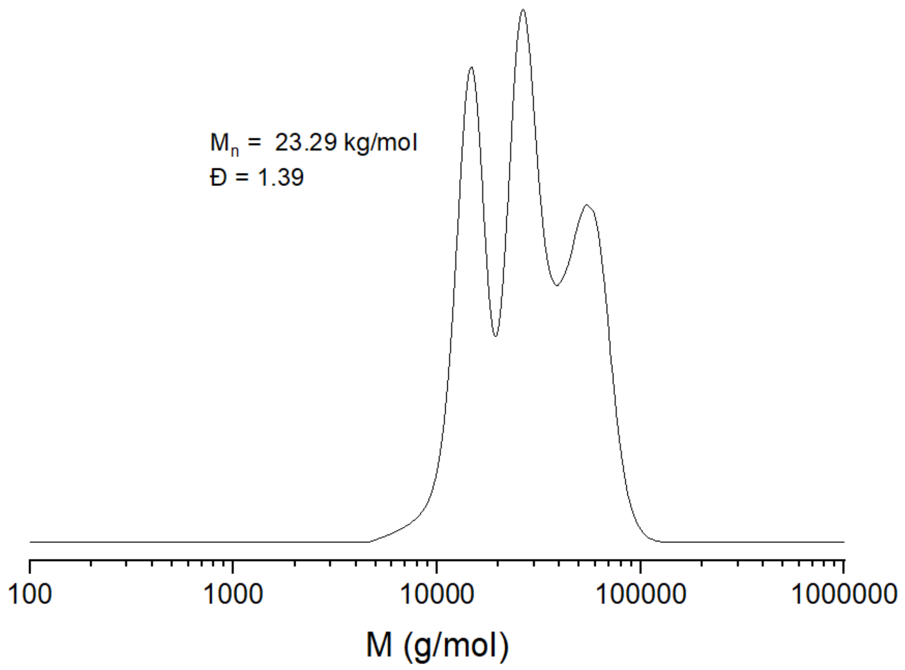

Figure S16 - GPC trace of ^{F}SO/PA copolymer corresponding to Table S2 entry 3.

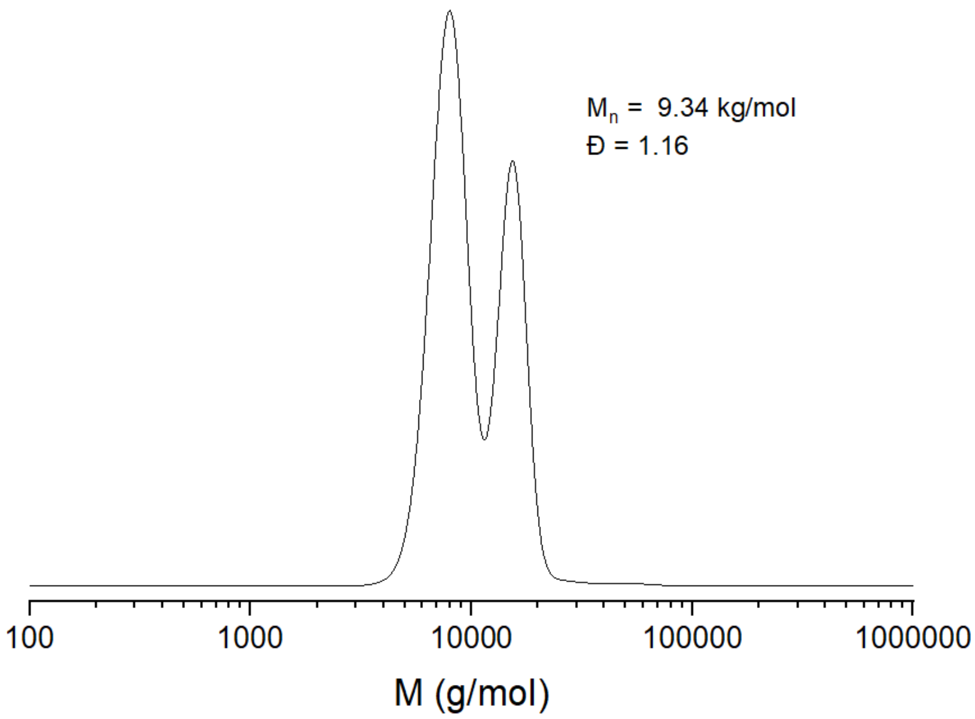

Figure S17 - GPC trace of ^{F}SO/PA copolymer corresponding to Table S2 entry 4.

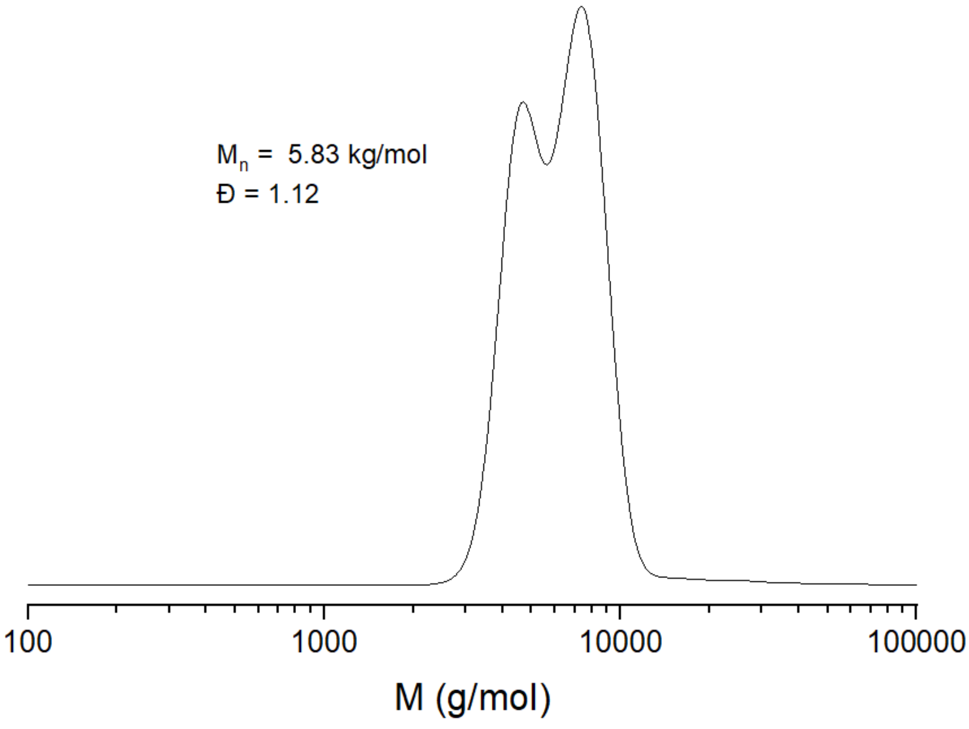

Figure S18 - GPC trace of ^{F}SO/PA copolymer corresponding to Table S2 entry 5.

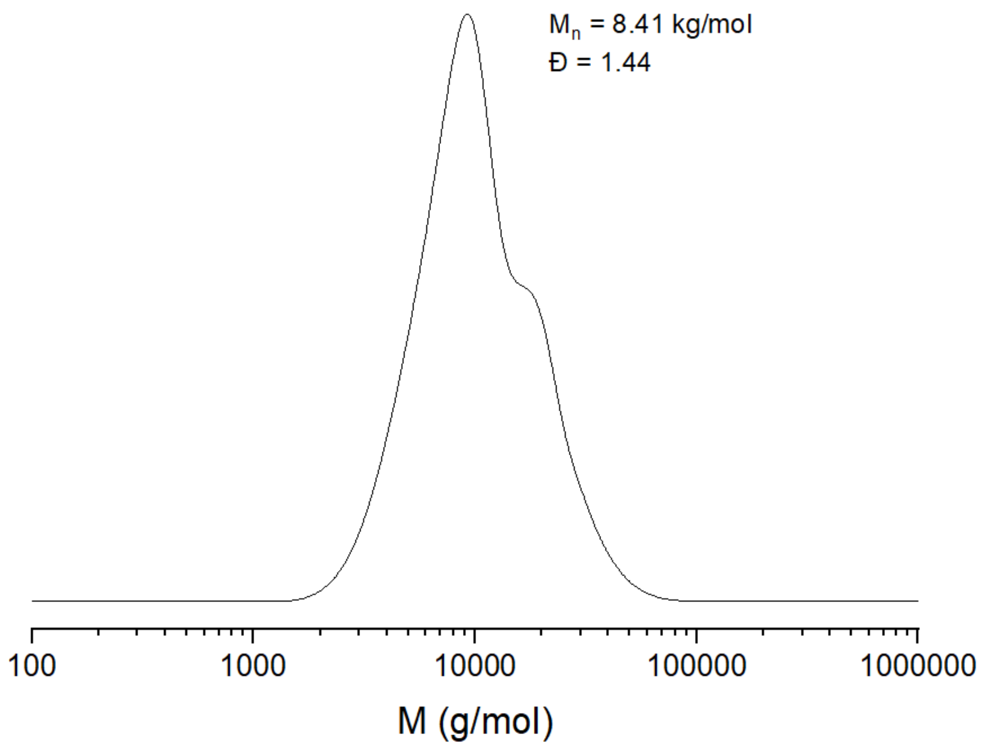

Figure S19 – GPC trace corresponding to 1 Cat.:1 Cocat: 100 PA: 500 ^{F}SO at 80 °C.

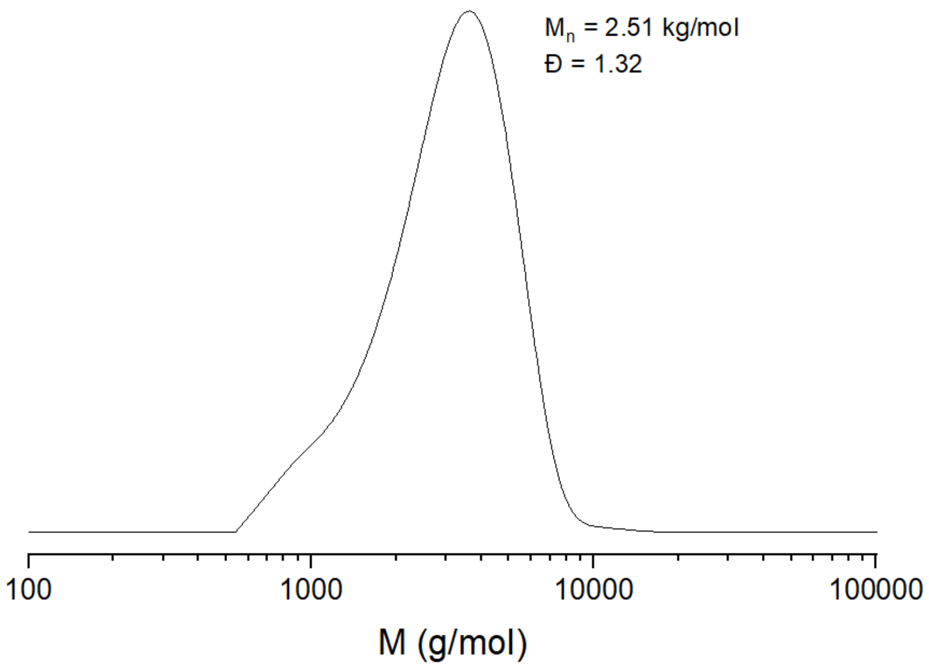

Figure S20 – GPC trace corresponding to 1 Cat.:1 Cocat: 20 BDM: 100 PA: 500 ^{F}SO at 80 °C.

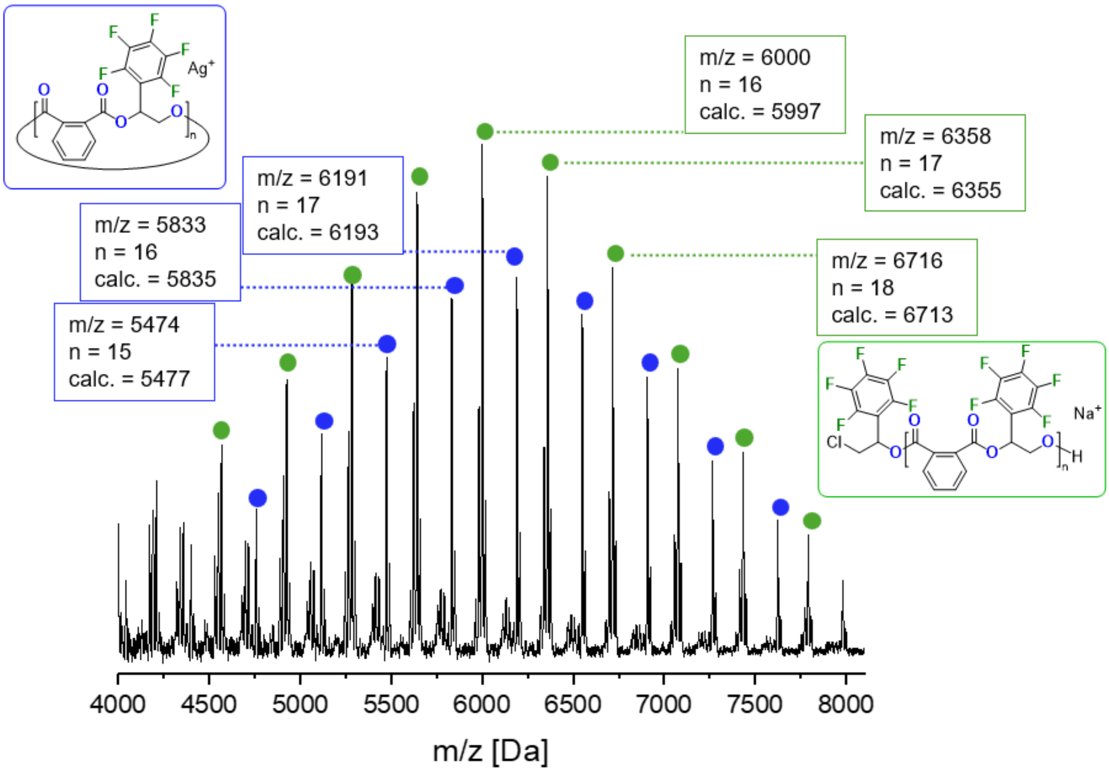

Figure S21 – MALDI-TOF of ^{F}SO/PA copolymer corresponding to table S2 entry 4.

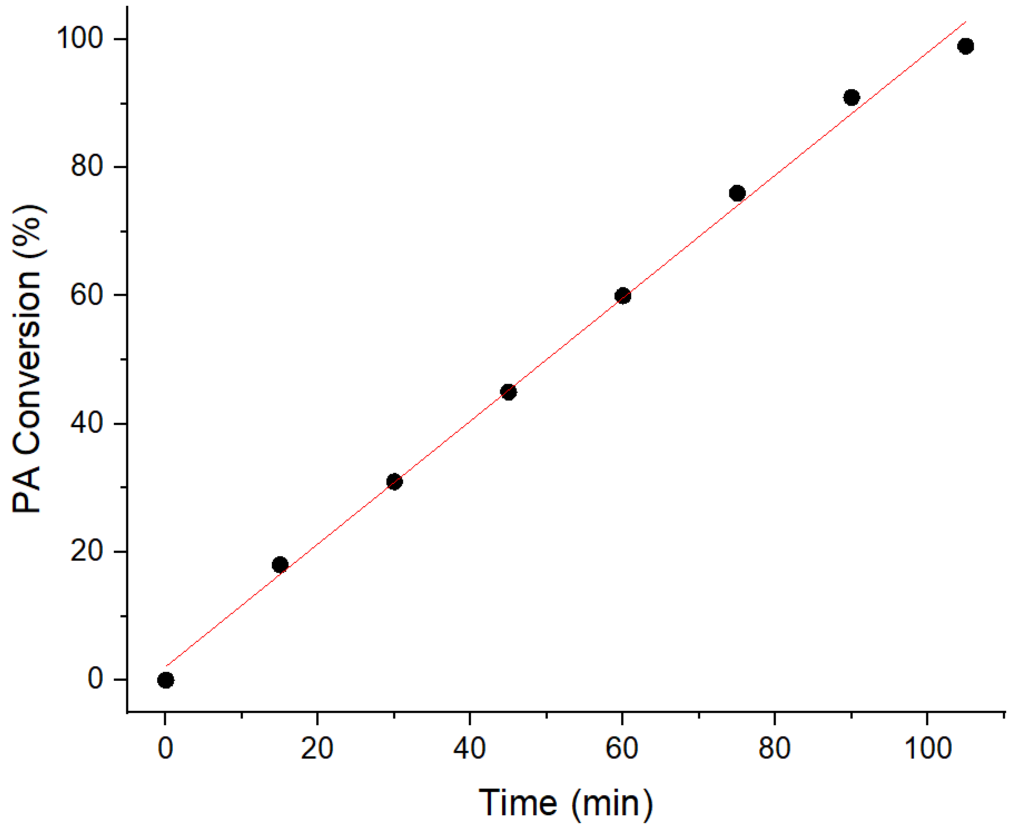

Figure S22 – 1:1:500:100 Cat.:Cocat.:^{F}SO:PA at 50 °C showing linear PA conversion vs time.

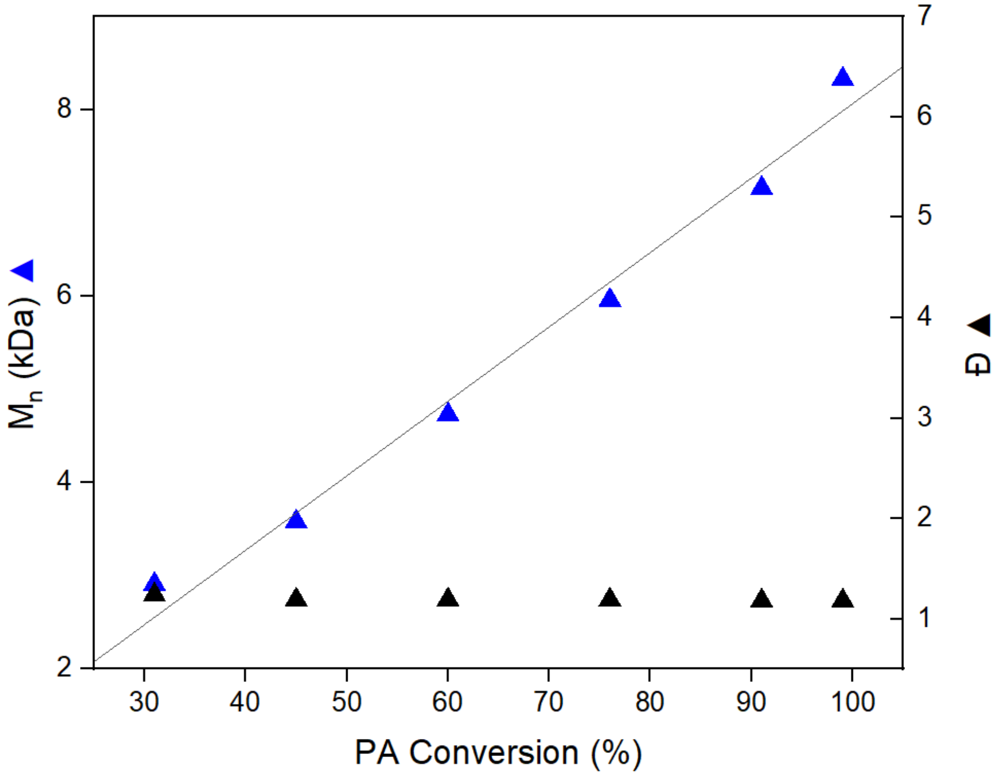

Figure S23 - 1:1:500:100 Cat.:Cocat.:^{F}SO:PA at 50 °C showing linear PA conversion vs molecular weight (M_n) and polydispersion (*Đ*).

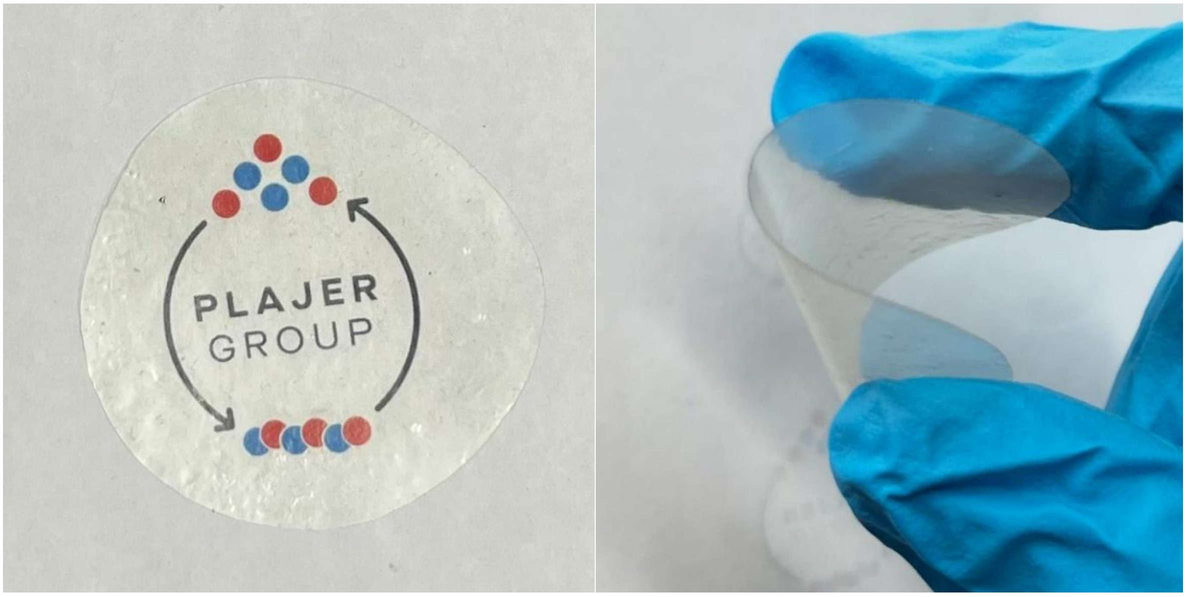

Figure S24 – ^{F}SO/PA copolymer (Table S2 entry 2) after compression moulding of material at 140 °C under 2 tonnes of pressure for 2 hours.

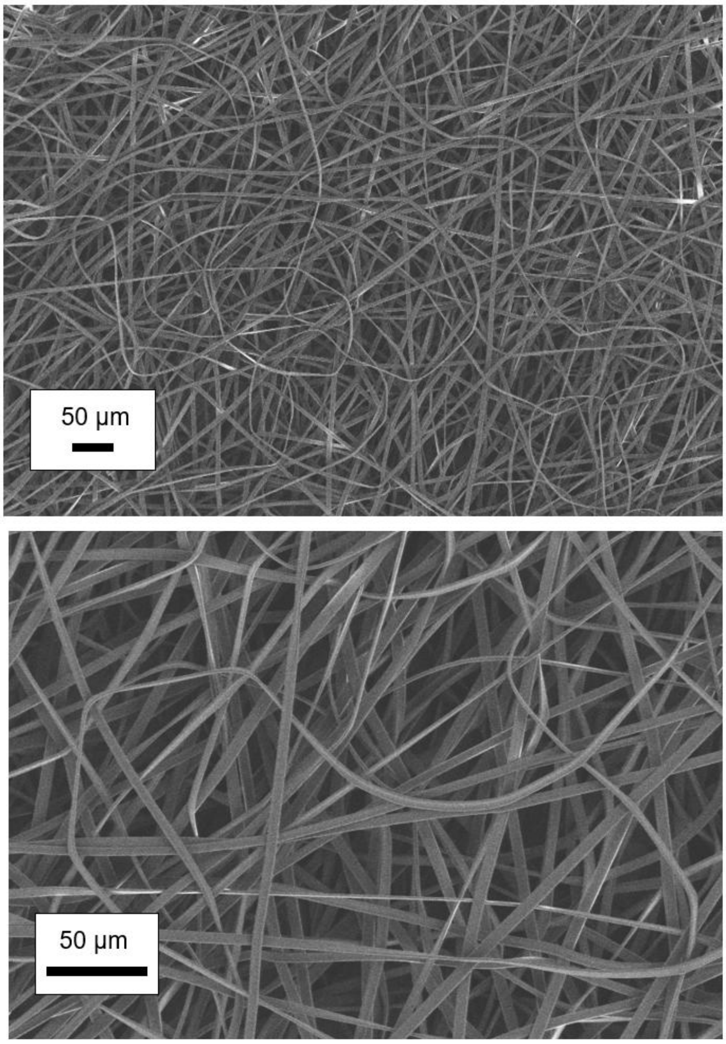

Figure S25 – Scanning Electron Microscopy (SEM) images of electro spun fibres

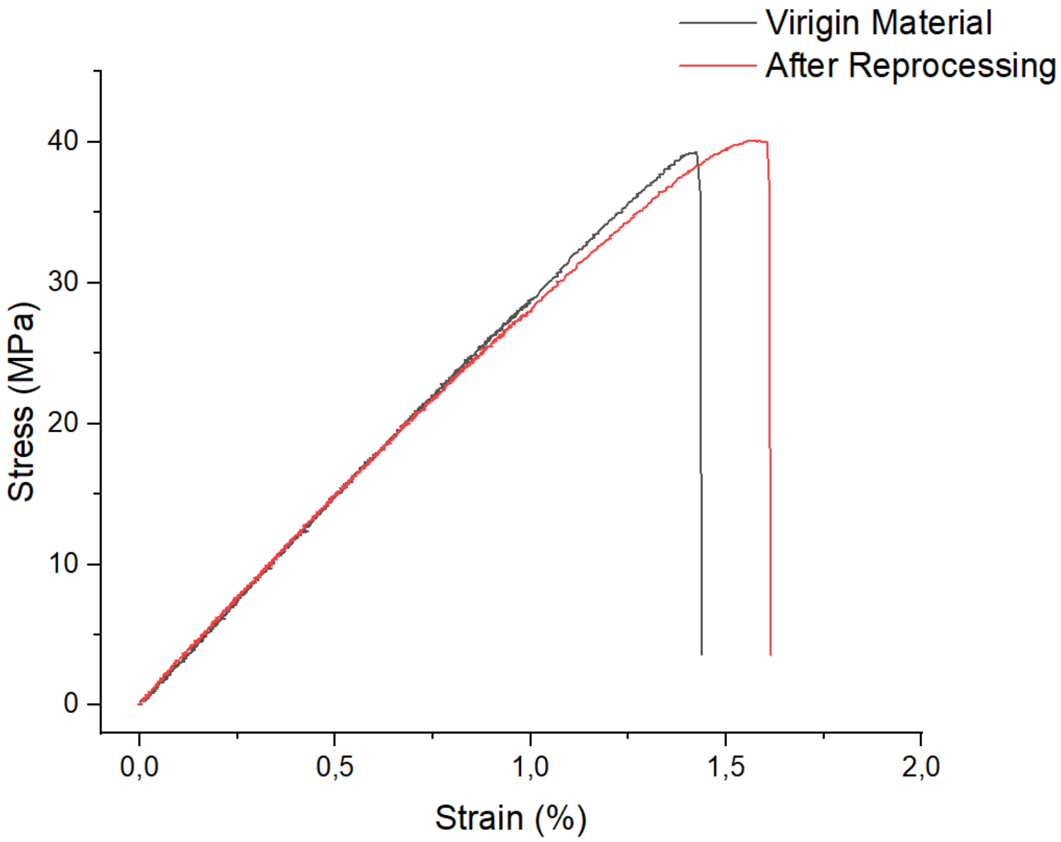

Figure S26 – Stress/Strain curve of ^{F}SO/PA copolymer (Table S2 entry 2) compression moulded film .

Table S3 – Tensile Test Data

	E_{Y}^{a} [GPa]	σ^{b} [MPa]	ε^{c} [%]
Virgin Material	2.7417 ± 0.271	38.67 ± 3.35	1.56 ± 0.16
After Reprocessing	2.8879 ± 0.140	38.79 ± 3.08	1.64 ± 0.10

[a] - Young‘s modulus, determined from the strain/stress gradient. [b] - Ultimate tensile strength. [c] - Tensile strain at break. Results from the tensile test strength of dog bones cut from ^{F}SO/PA copolymer (Table S2 entry 2). Average values and associated errors are calculated from 5 samples.

Section S4: ^{F}PhGE/PA copolymerisation

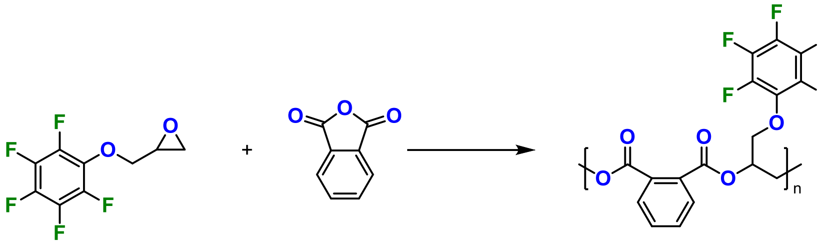

Figure S27 - Copolymerisation of ^{F}PhGE and PA.

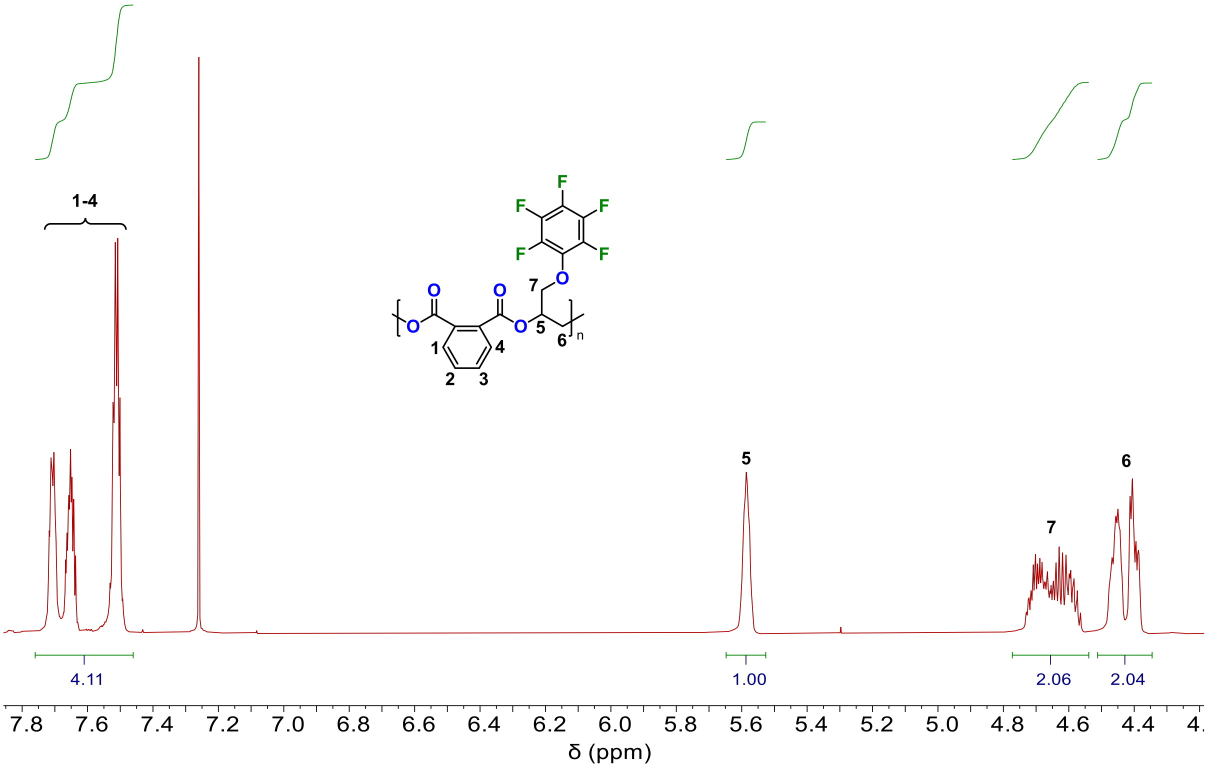

Figure S28 - ^{1}H NMR spectrum (500 MHz, $CDCl_3$) of ^{F}PhGE/PA copolymerisation corresponding to Table S1 entry 2.

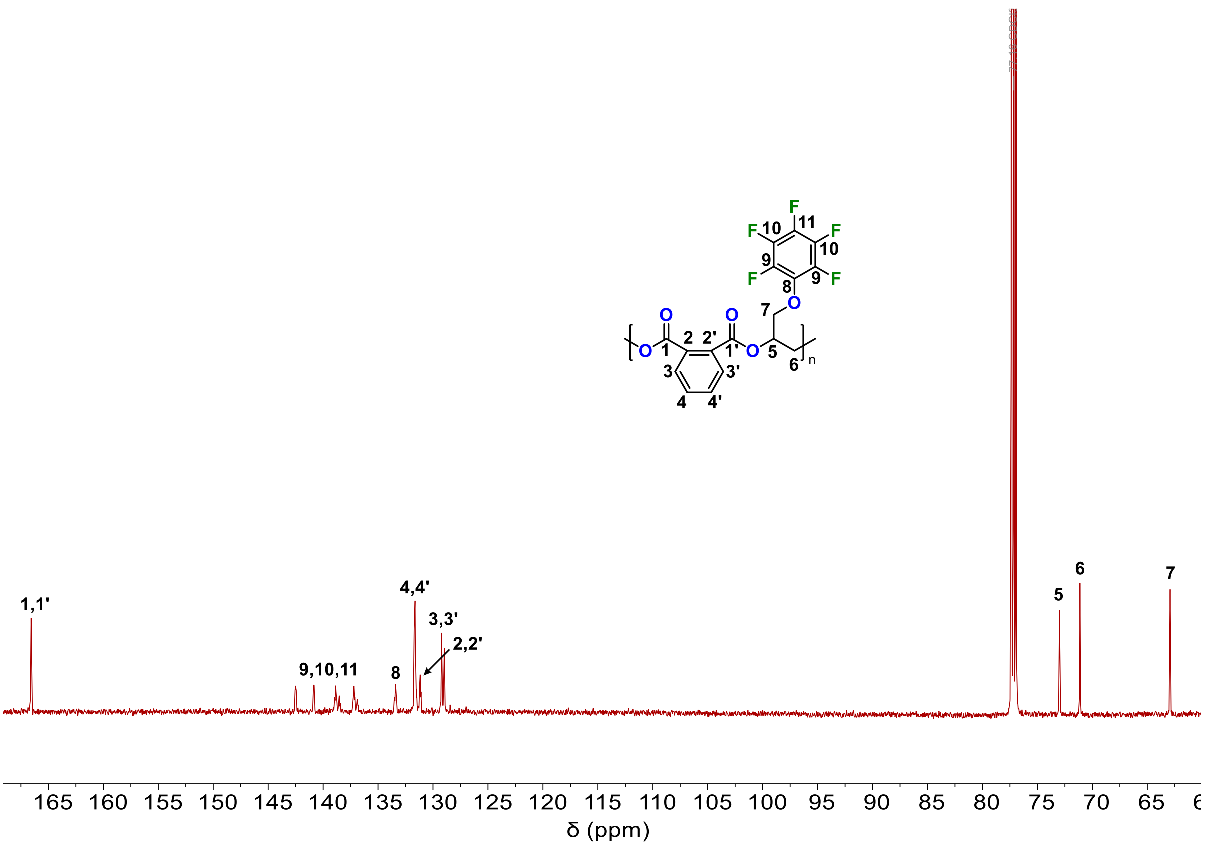

Figure S29 - ^{13}C NMR spectrum (126 MHz, $CDCl_3$) of ^{F}PhGE/PA copolymer corresponding to Table S1 entry 2.

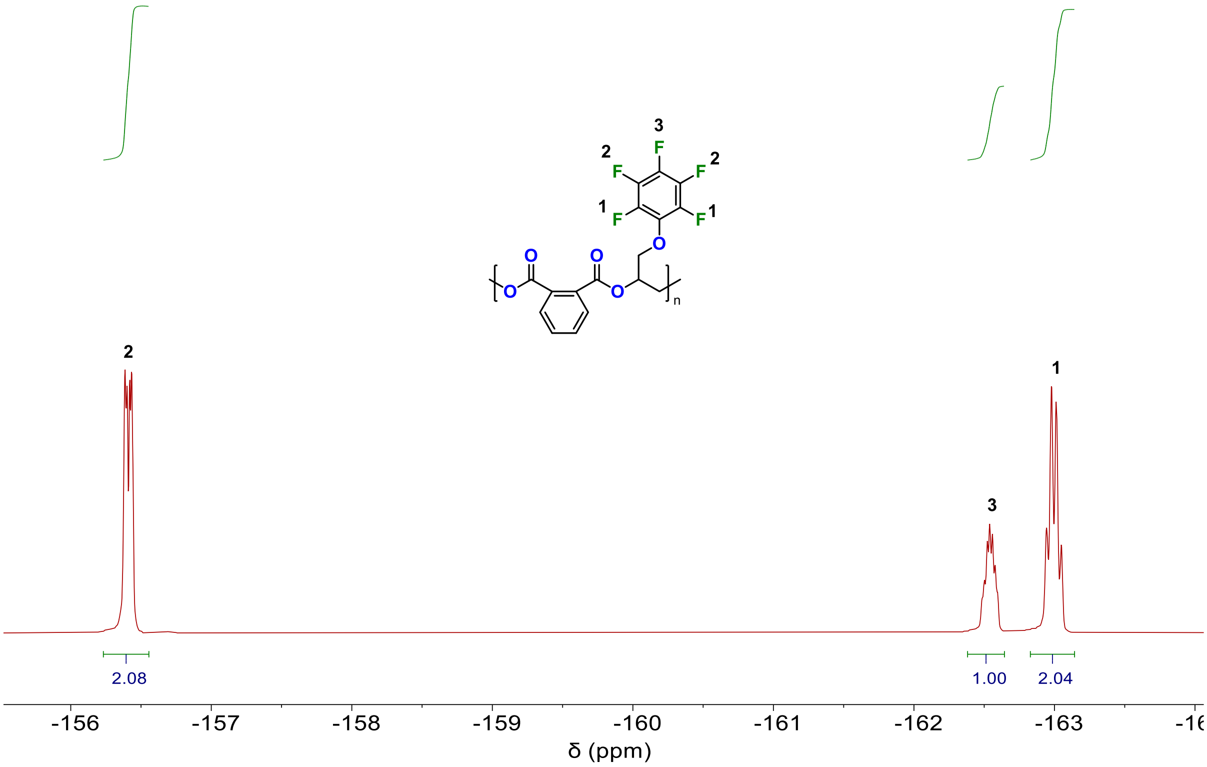

Figure S30 - ^{19}F NMR spectrum (376 MHz, $CDCl_3$) of ^{F}PhGE/PA copolymer corresponding to Table S1 entry 2.

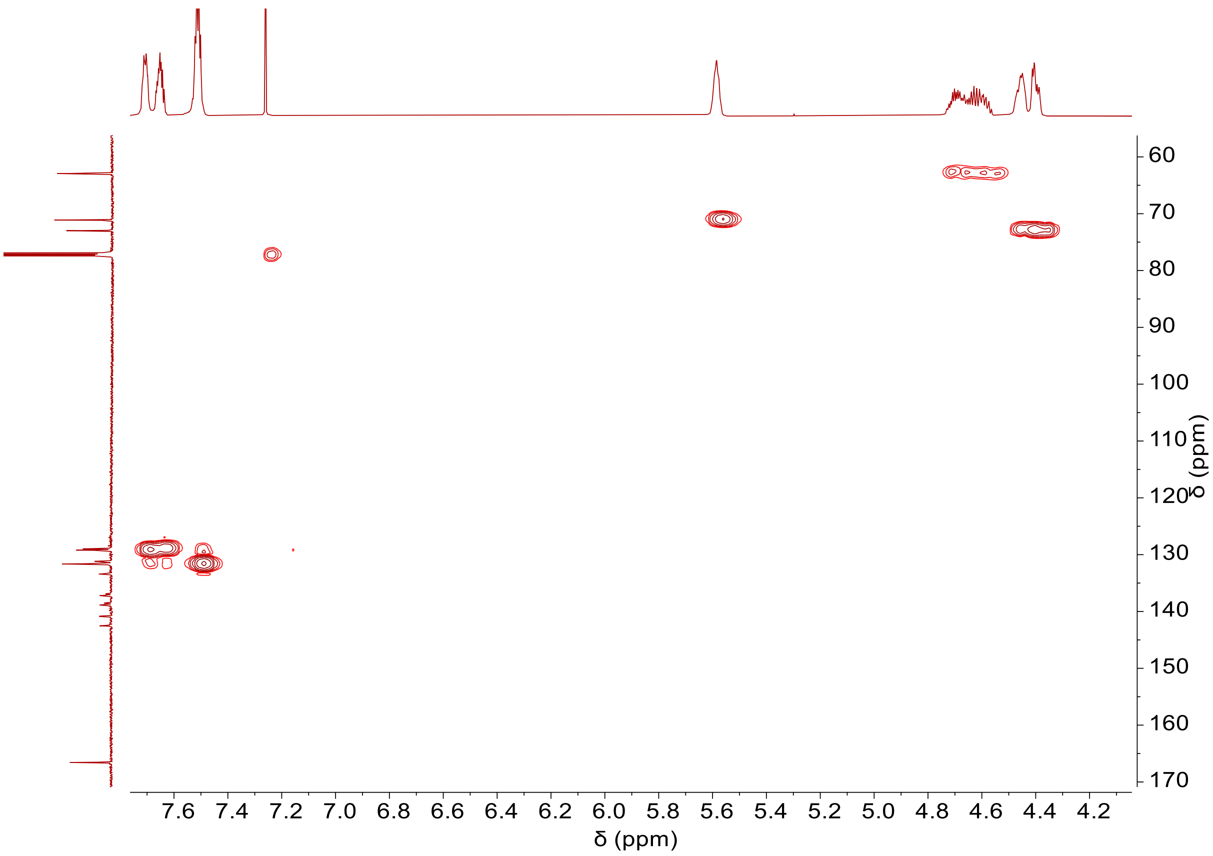

Figure S31 - ^{1}H-^{13}C HSQC NMR spectrum ($CDCl_3$) spectrum of the ^{F}PhGE/PA copolymer corresponding to Table S1 entry 2.

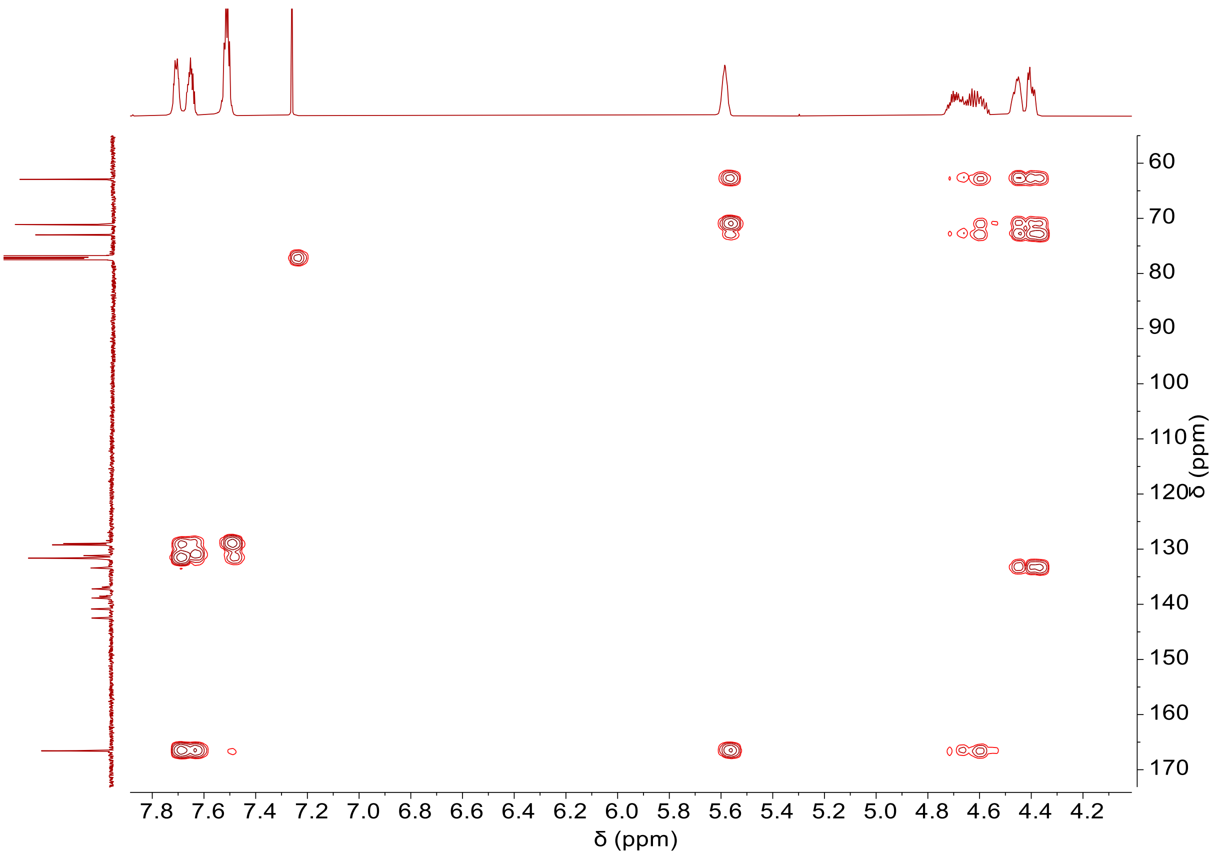

Figure S32 - ^{1}H-^{13}C HMBC NMR spectrum ($CDCl_3$) spectrum of the ^{F}PhGE/PA copolymer corresponding to Table S1 entry 2.

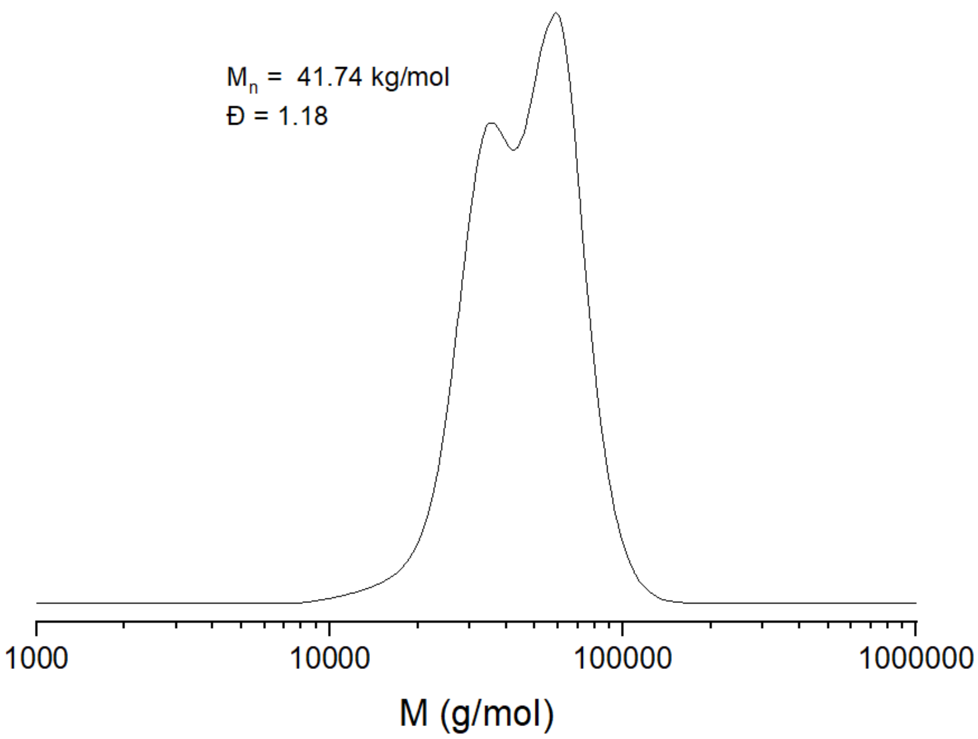

Figure S33 – GPC trace of ^{F}PhGE/PA copolymer corresponding to Table S1 entry 2.

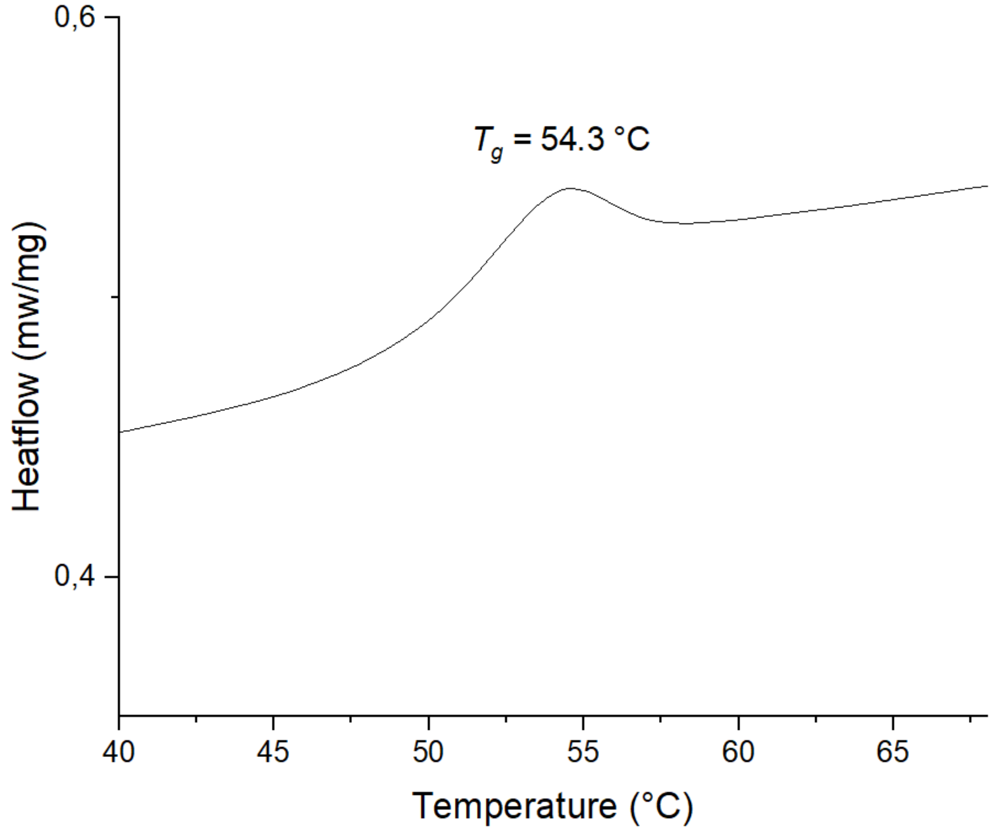

Figure S34 - DSC data from the second heating cycle of ^{F}PhGE/PA copolymer corresponding to Table S1 entry 2.

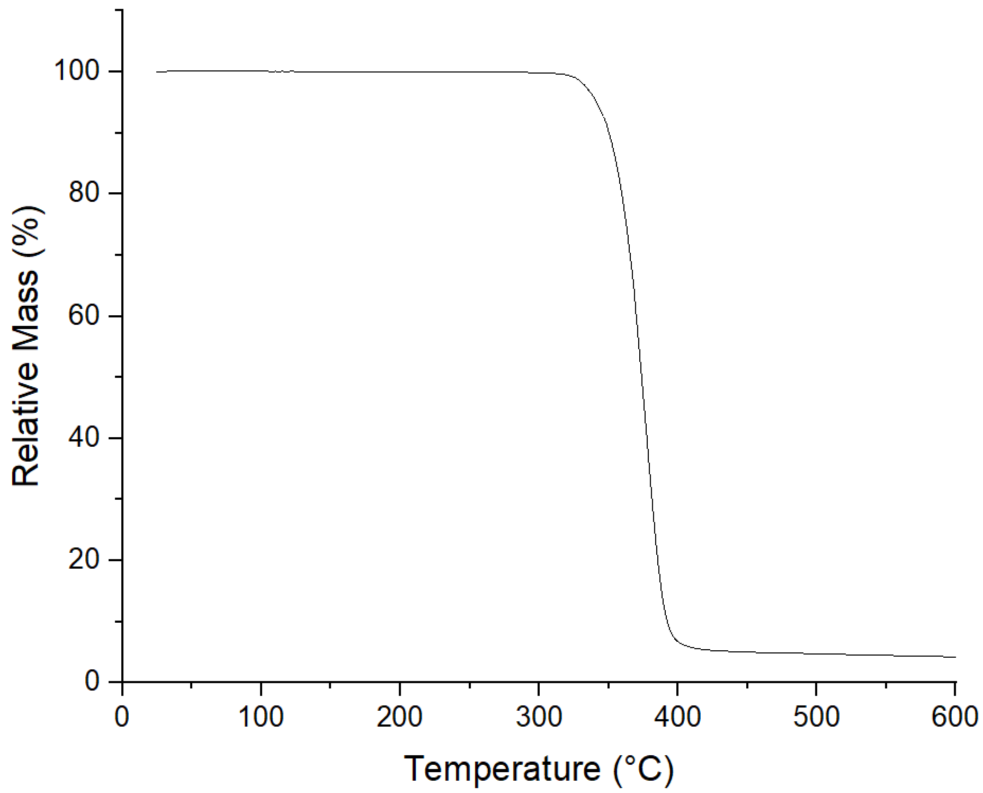

Figure S35 - TGA data of copolymer of ^{F}PhGE/PA copolymer corresponding to Table S1 entry 2. $T_{d,5\%}$ = 341.5 ºC.

Section S5: SO/PA copolymerisation

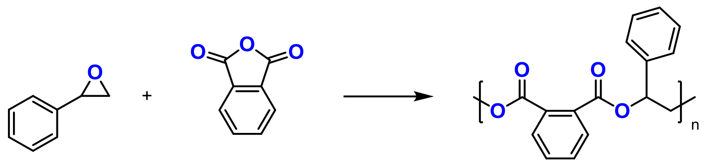

Figure S36 - Copolymerisation of SO and PA.

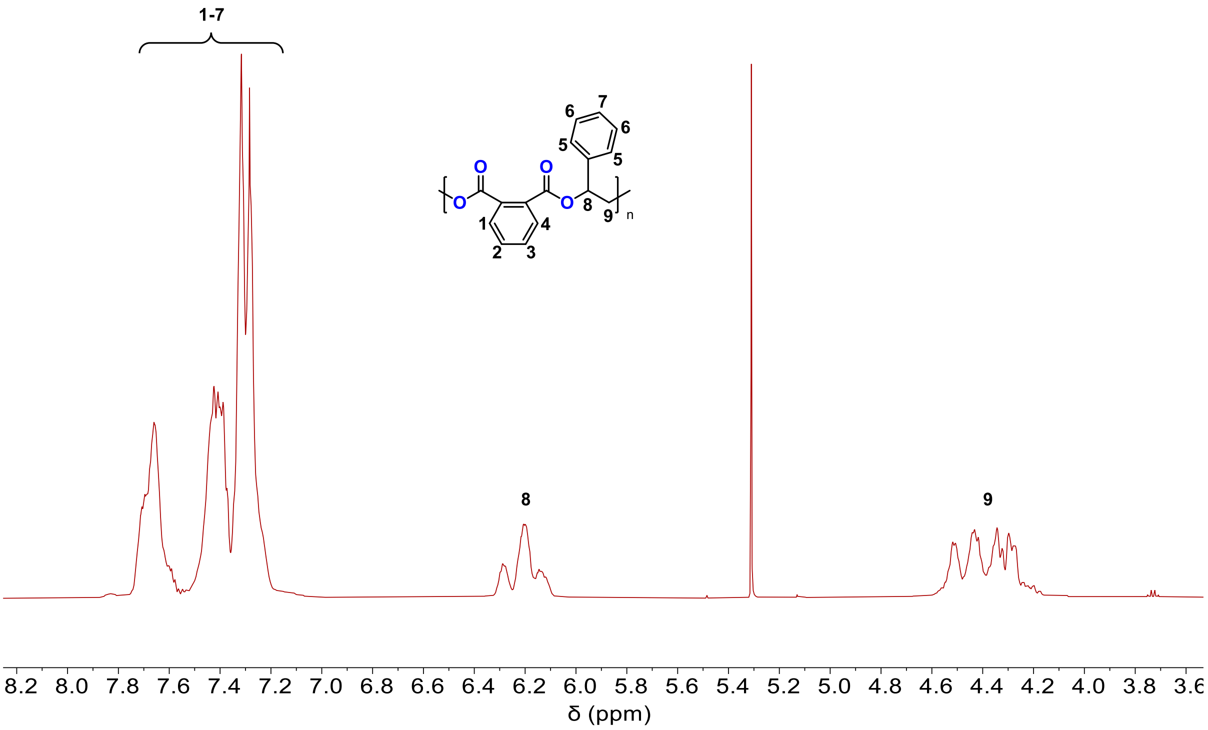

Figure S37 - ^{1}H NMR spectrum (500 MHz, $CDCl_3$) of SO/PA copolymerisation corresponding to Table S1 entry 3.

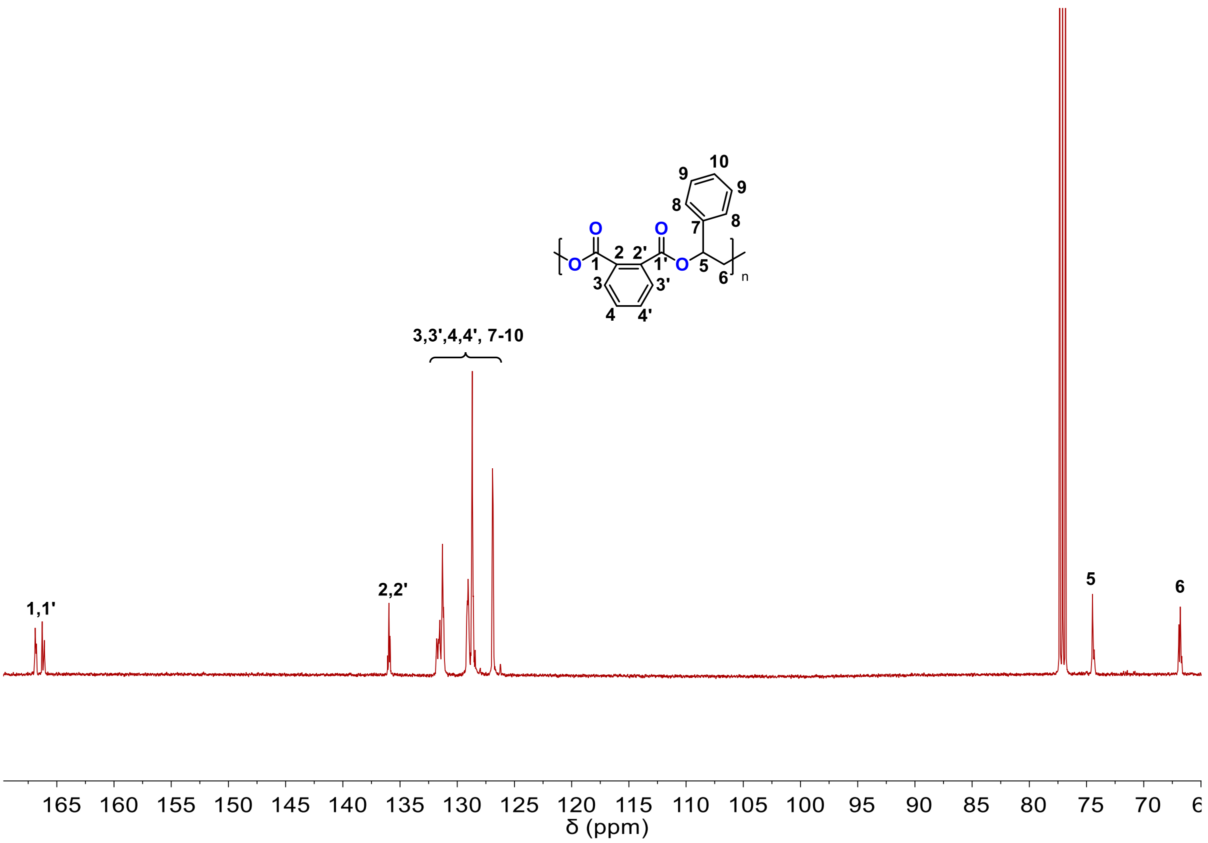

Figure S38 - ^{13}C NMR spectrum (126 MHz, $CDCl_3$) of SO/PA copolymer corresponding to Table S1 entry 3.

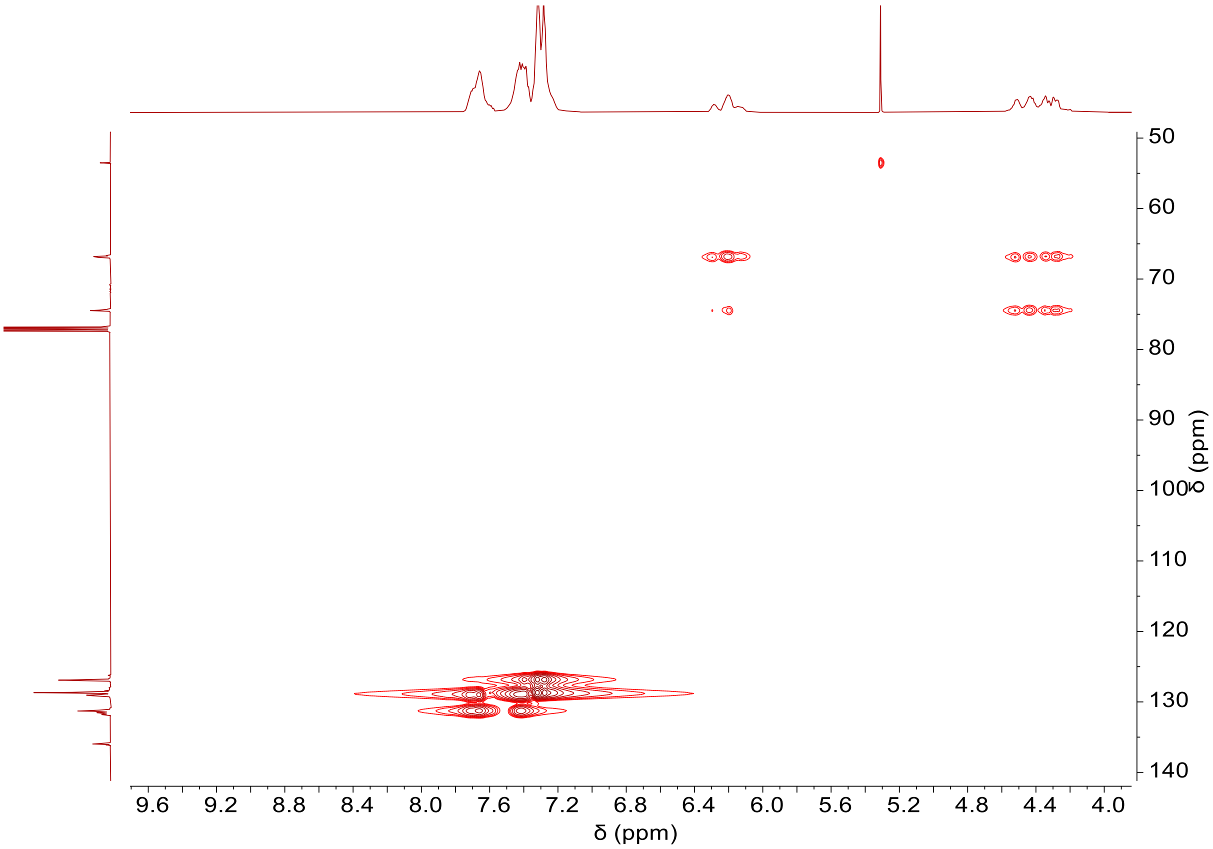

Figure S39 - ^{1}H-^{13}C HSQC NMR spectrum ($CDCl_3$) spectrum of the SO/PA copolymer corresponding to Table S1 entry 3.

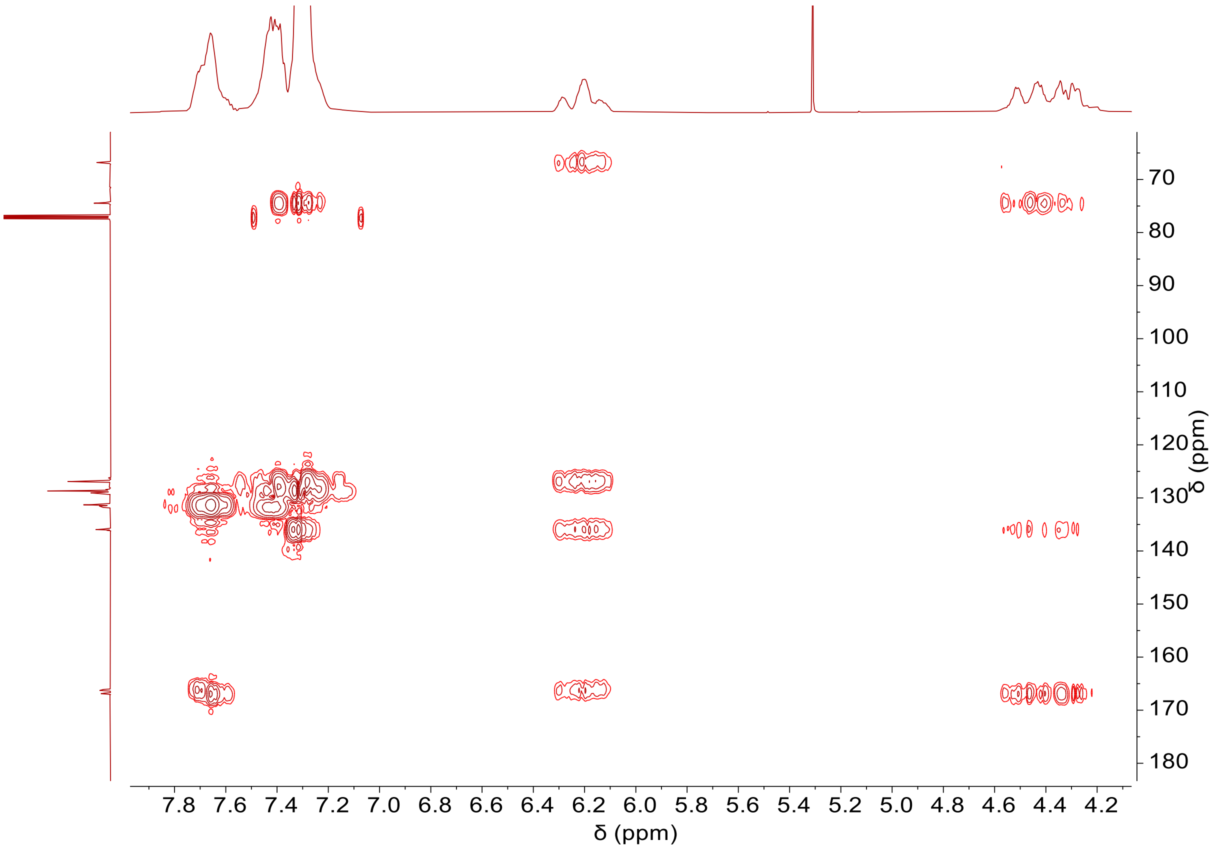

Figure S40 - ^1H-^{13}C HMBC NMR spectrum ($CDCl_3$) spectrum of the SO/PA copolymer corresponding to Table S1 entry 3.

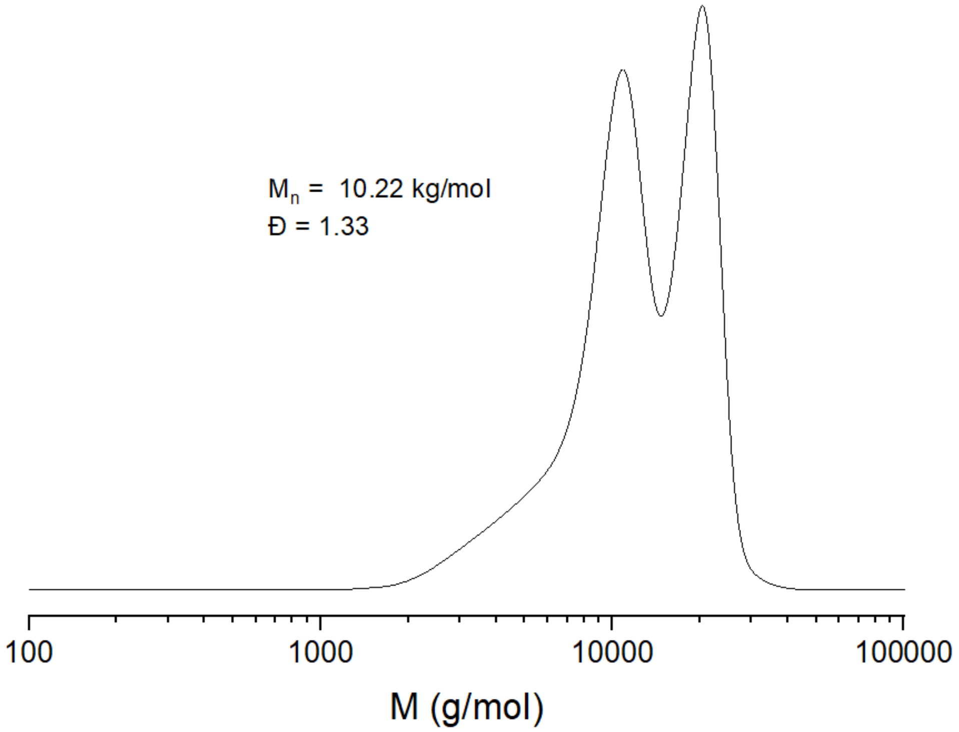

Figure S41 – GPC trace of PA/PO copolymer corresponding to Table S1 entry 3.

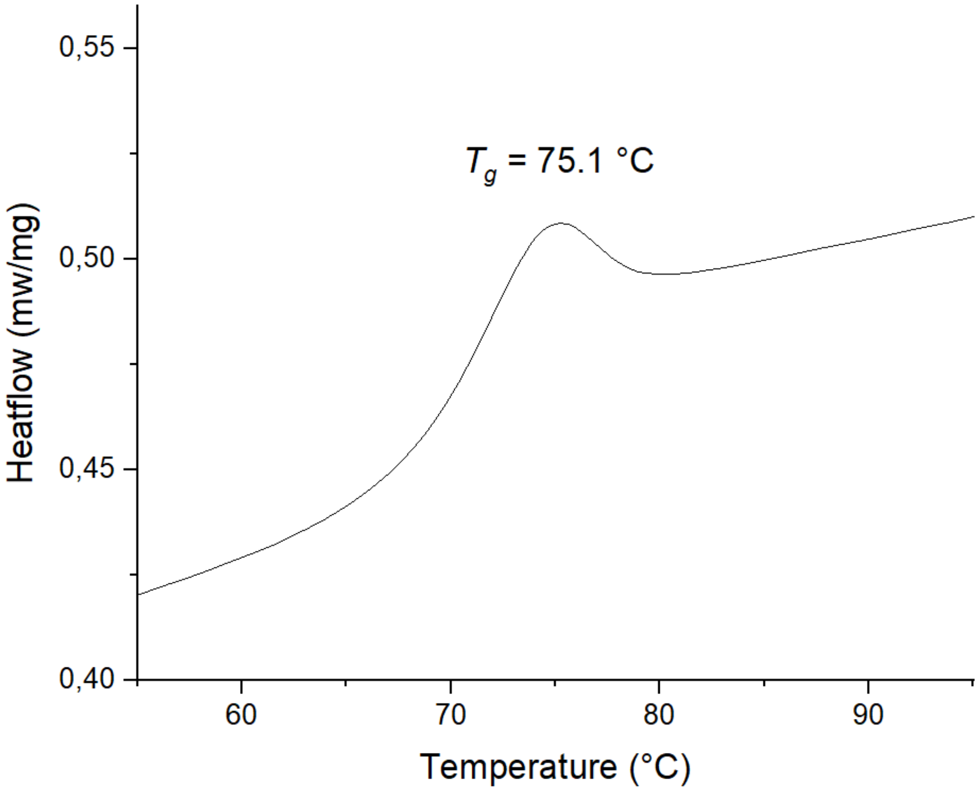

Figure S42 - DSC data from the second heating cycle of SO/PA copolymer corresponding to Table S1 entry 3.

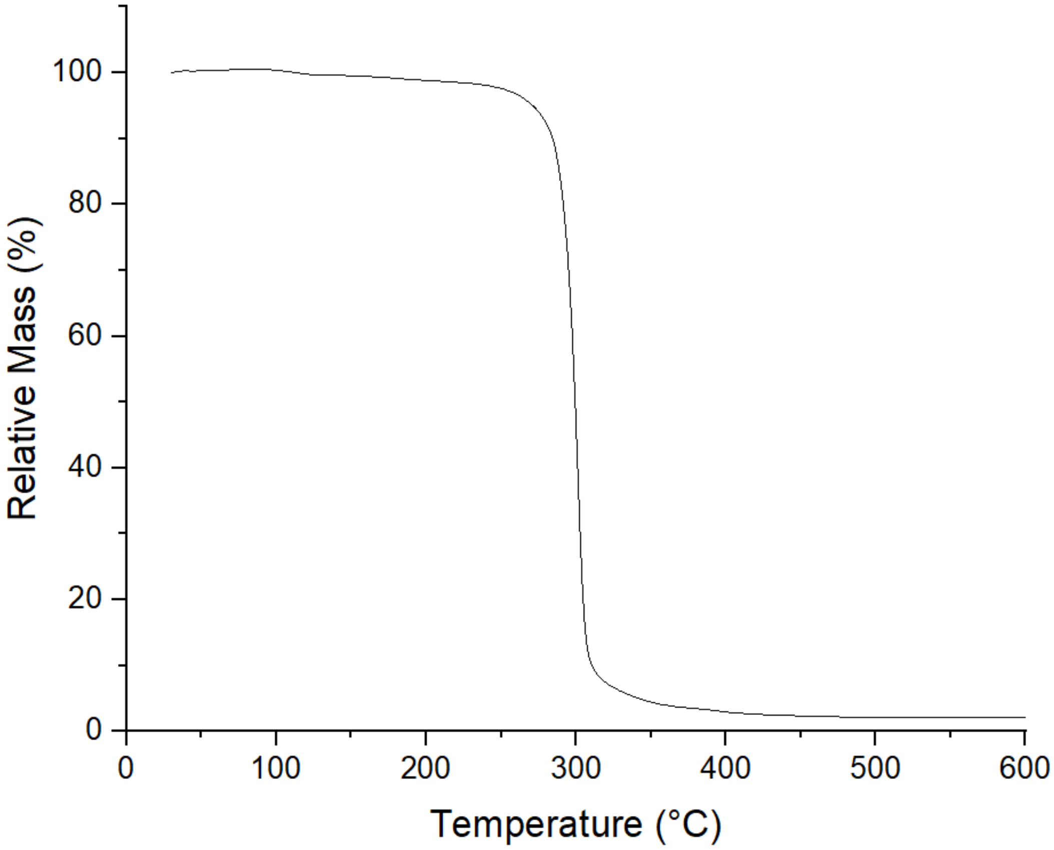

Figure S43 - TGA data of copolymer of PO/PA copolymer corresponding to Table S1 entry 3. $T_{d,5\%}$ = 280.7 ºC.

Section S6: PhGE/PA copolymerisation

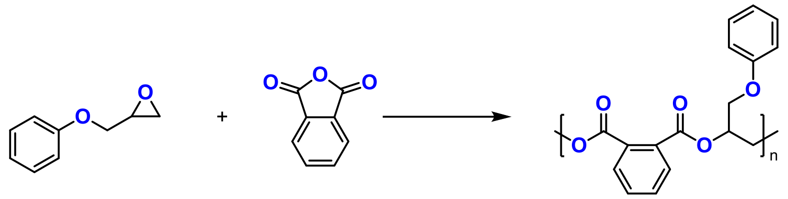

Figure S44 – Copolymerisation of PhGE and PA.

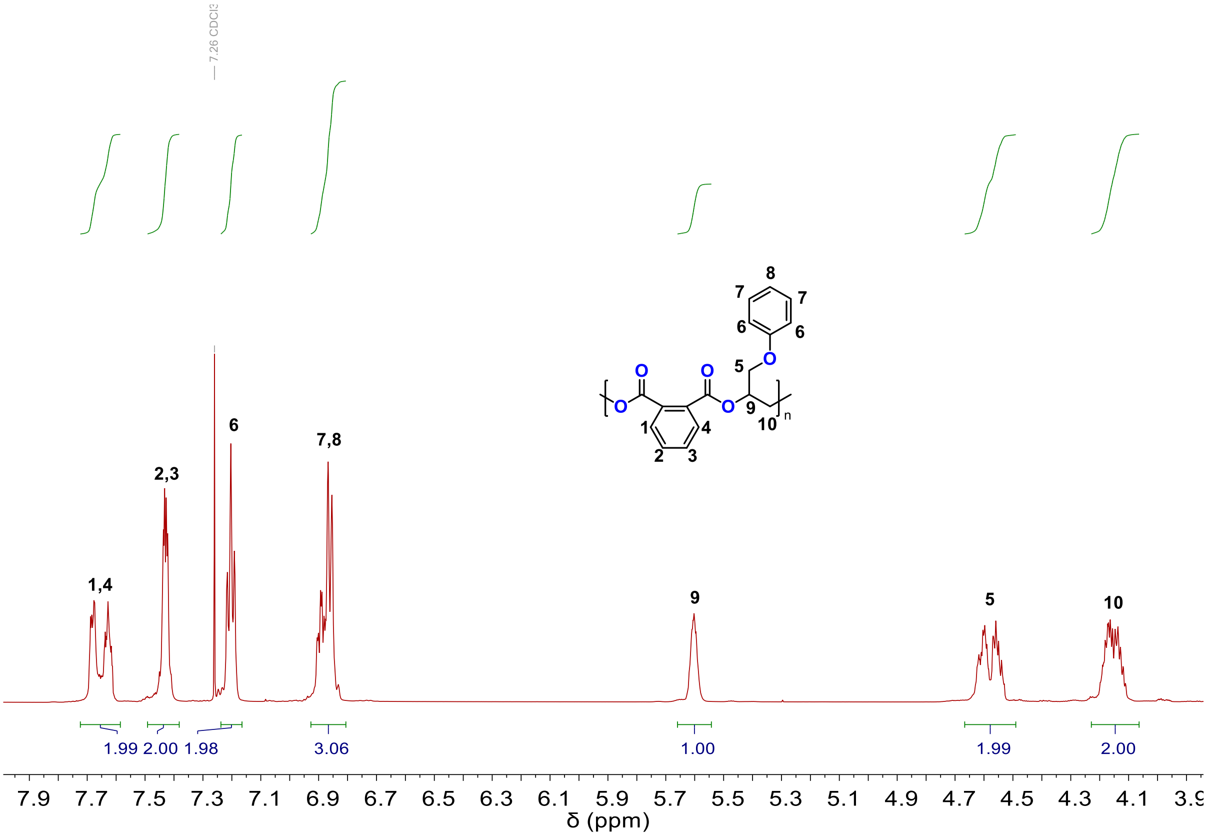

Figure S45 - ^{1}H NMR spectrum (500 MHz, $CDCl_3$) of PhGE/PA copolymerisation corresponding to Table S1 entry 4.

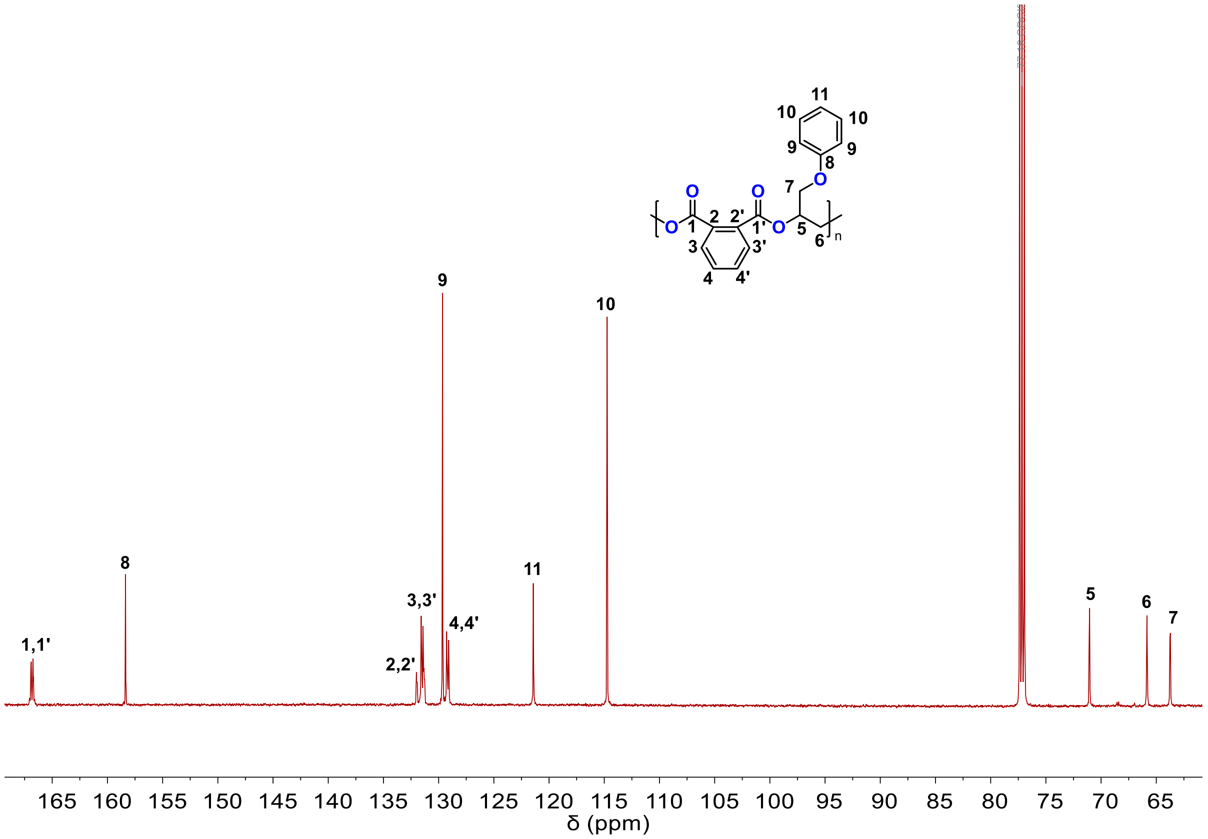

Figure S46 - ^{13}C NMR spectrum (126 MHz, $CDCl_3$) of PhGE/PA copolymer corresponding to Table S1 entry 4.

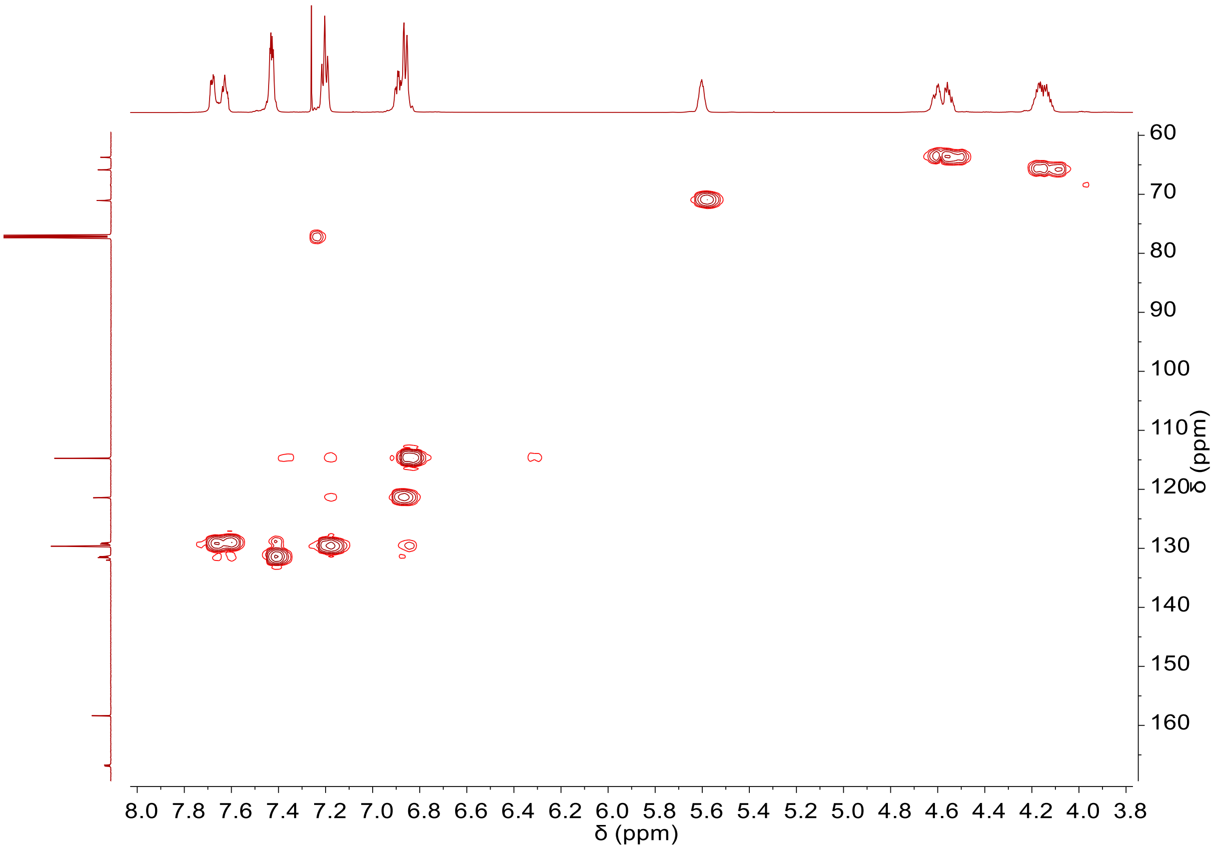

Figure S47 - ^{1}H-^{13}C HSQC NMR spectrum ($CDCl_3$) spectrum of the PhGE/PA copolymer corresponding to Table S1 entry 4.

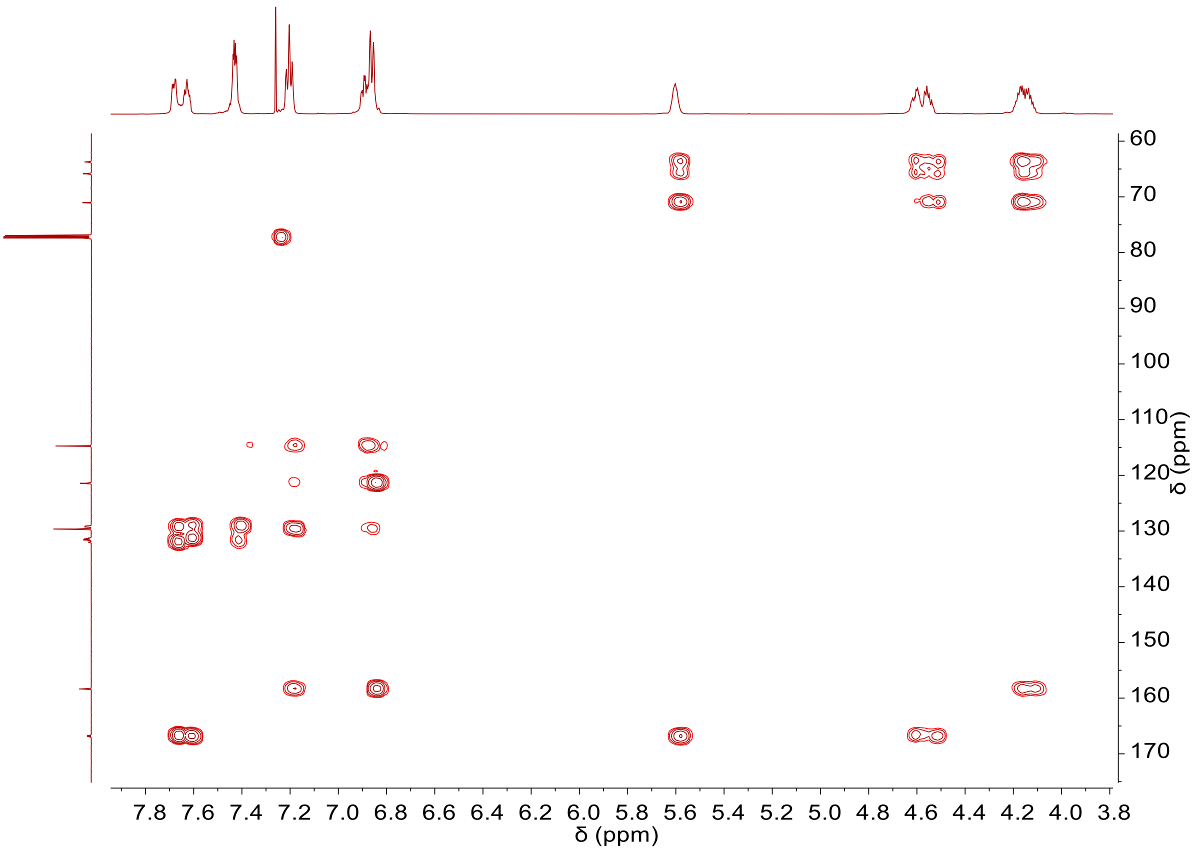

Figure S48- ^{1}H-^{13}C HMBC NMR spectrum ($CDCl_3$) spectrum of the PhGE/PA copolymer corresponding to Table S1 entry 4.

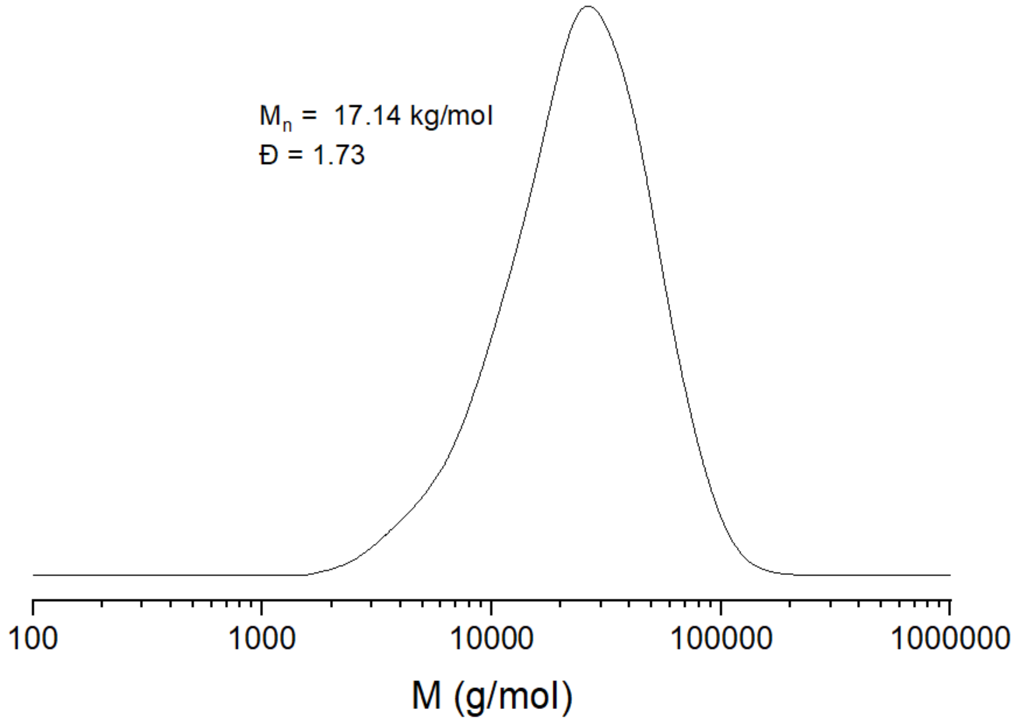

Figure S49 – GPC trace of PhGE/PA copolymer corresponding to Table S1 entry 4.

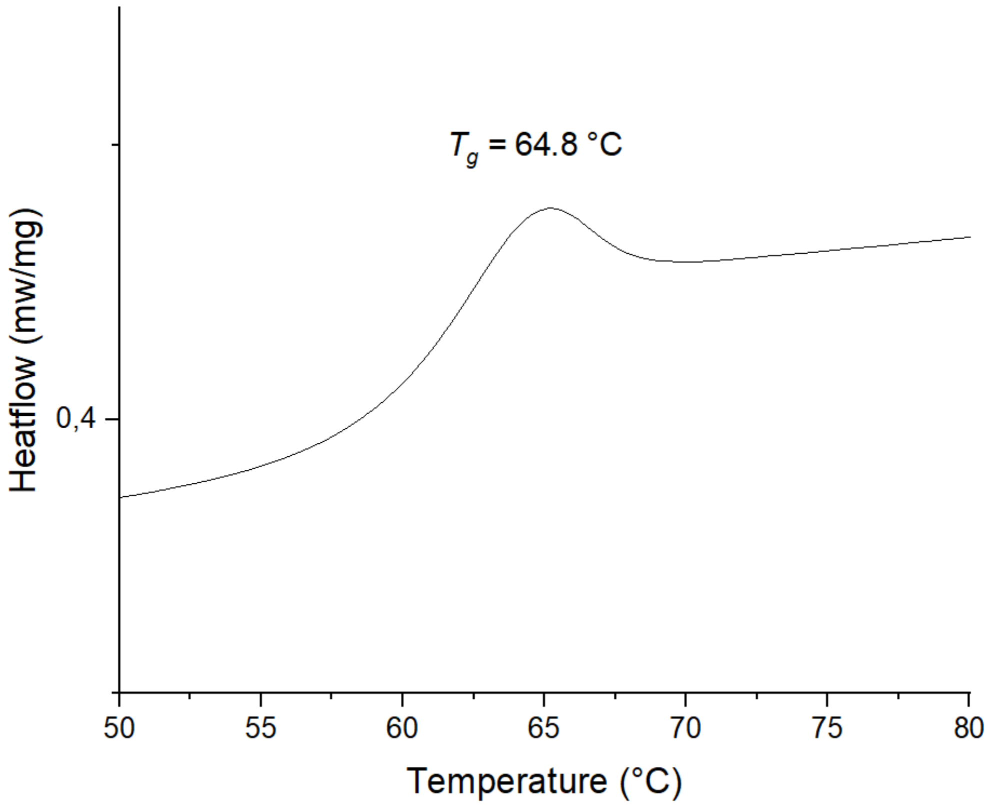

Figure S50 - DSC data from the second heating cycle of PhGE/PA copolymer corresponding to Table S1 entry 4.

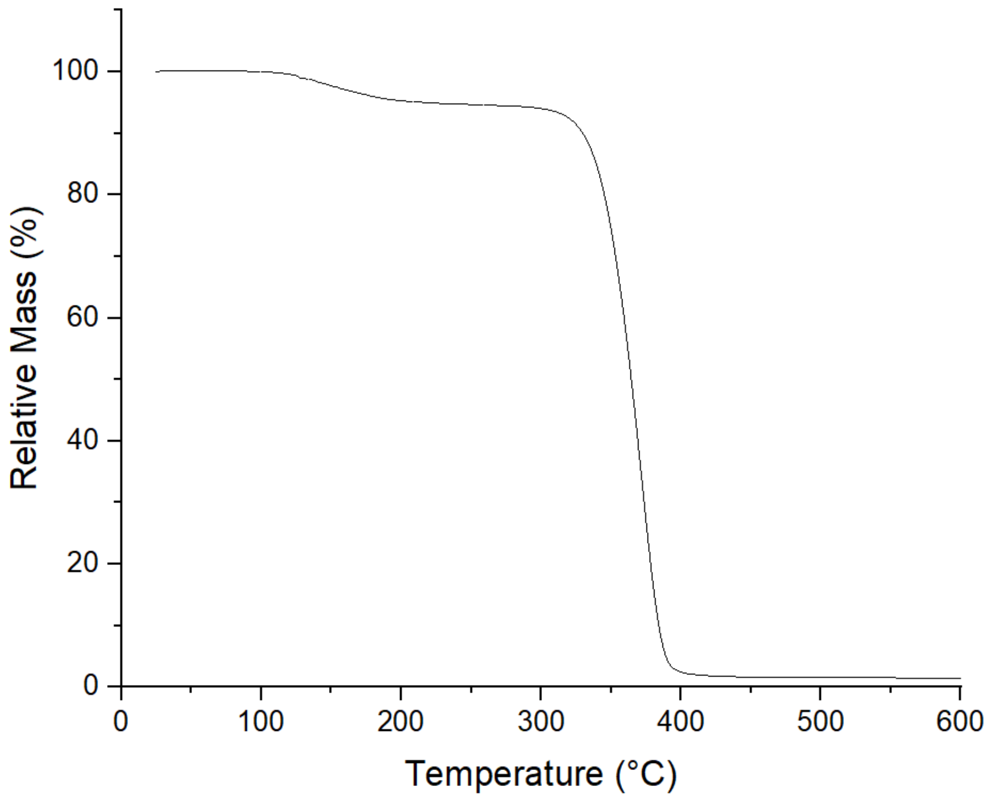

Figure S51 - TGA data of copolymer of PhGE/PA copolymer corresponding to Table S1 entry 4. $T_{d,5\%}$ = 224.1 ºC.

Section S7: ^{F}PO/PA copolymerisation

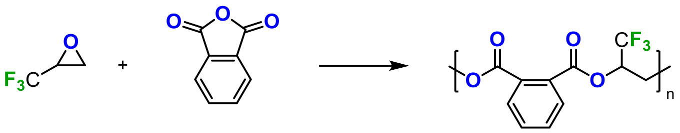

Figure S52 – Copolymerisation of ^{F}PO and PA

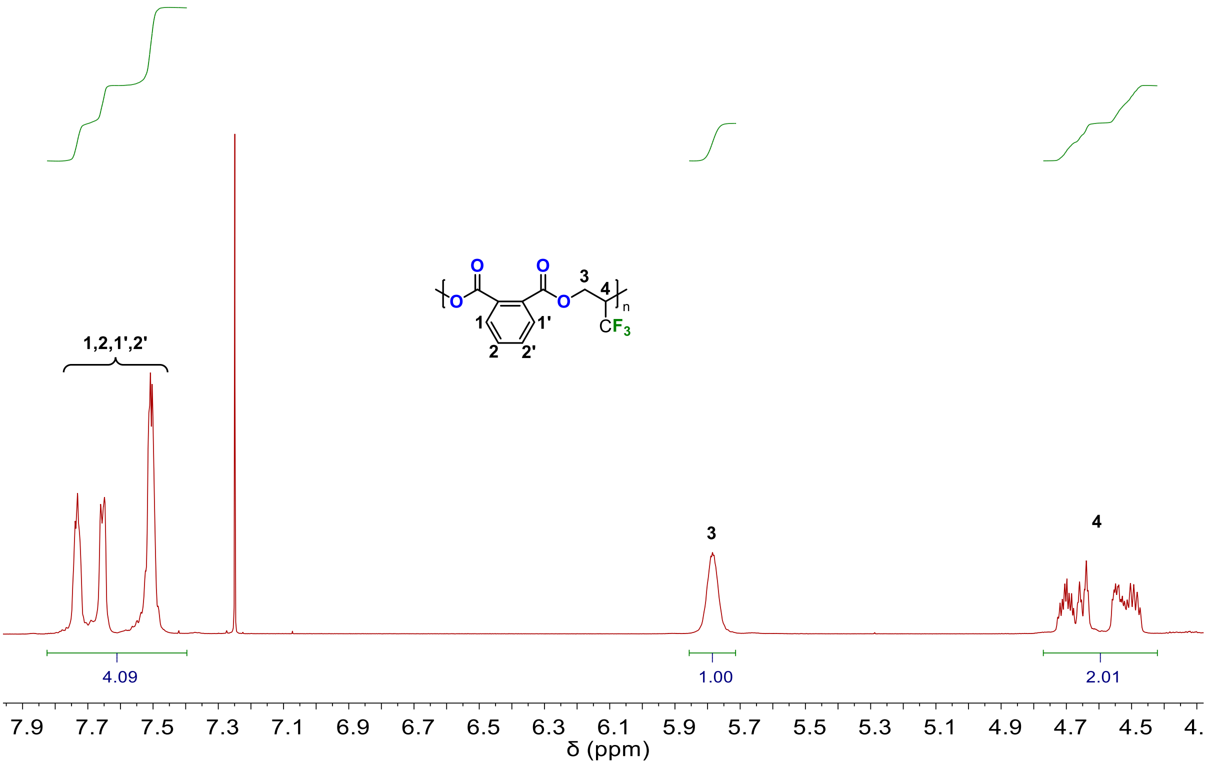

Figure S53 - ^{1}H NMR spectrum (500 MHz, $CDCl_3$) of ^{F}PO/PA copolymerisation corresponding to Table S1 entry 5.

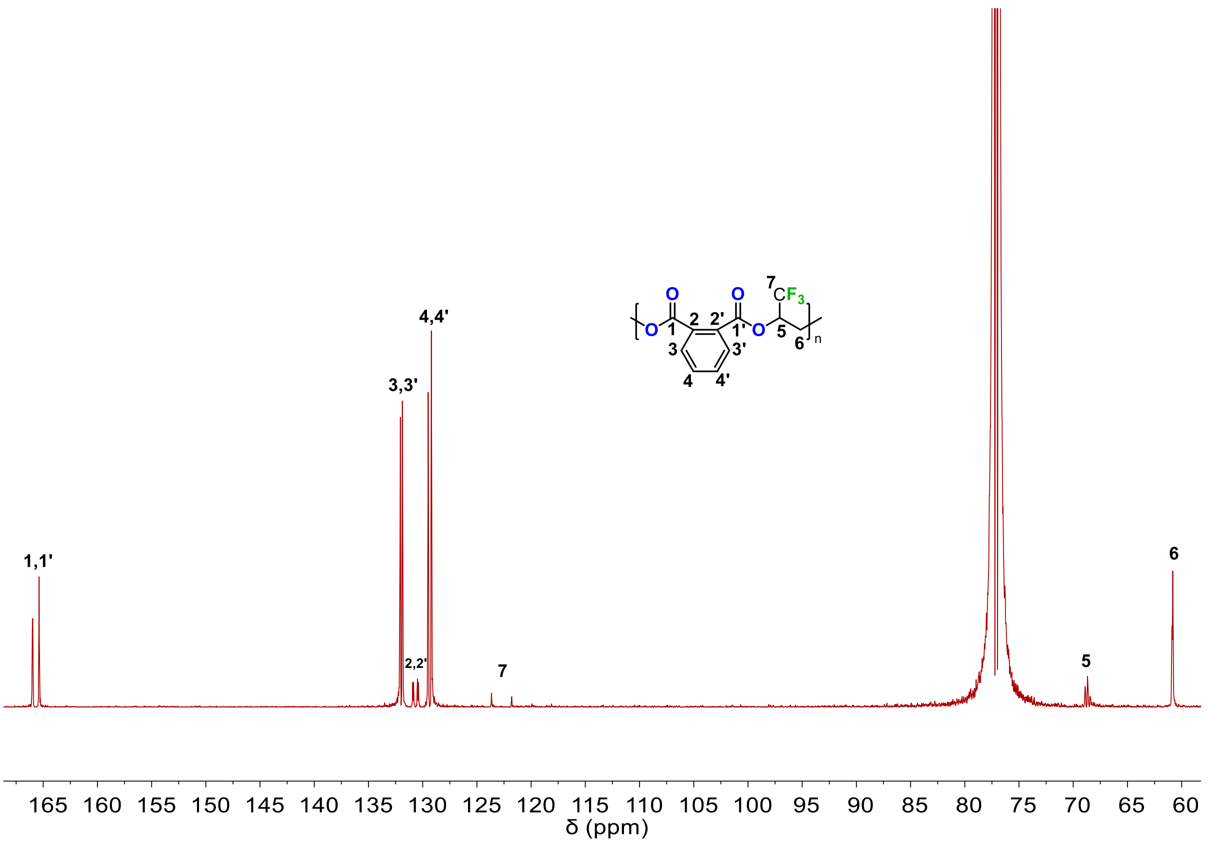

Figure S54 - ^{13}C NMR spectrum (126 MHz, $CDCl_3$) of ^{F}PO/PA copolymer corresponding to Table S1 entry 5.

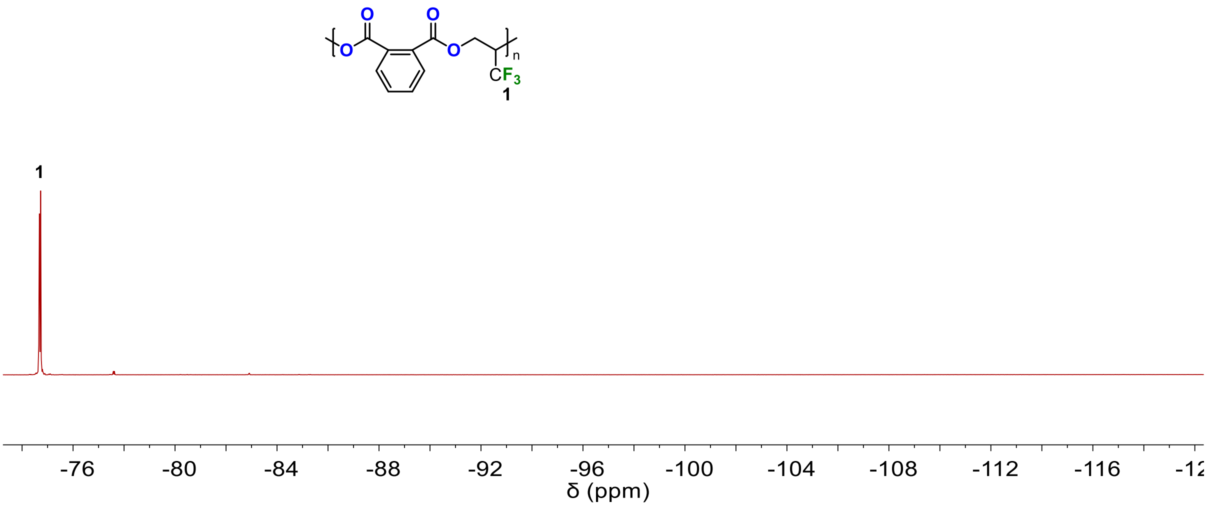

Figure S55 - ^{19}F NMR spectrum (376 MHz, $CDCl_3$) of ^{F}PO/PA copolymer corresponding to Table S1 entry 5.

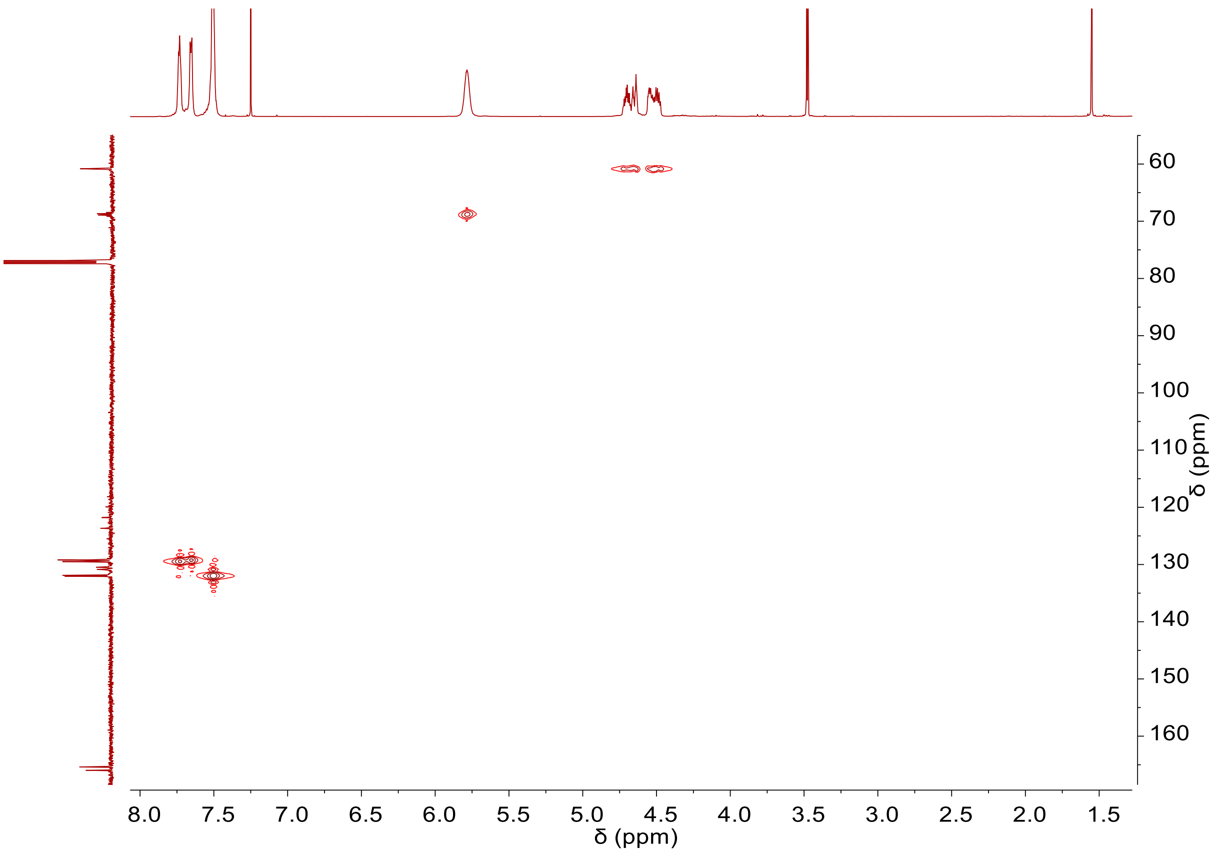

Figure S56 - ^{1}H-^{13}C HSQC NMR spectrum ($CDCl_3$) spectrum of the ^{F}PO/PA copolymer corresponding to Table S1 entry 5.

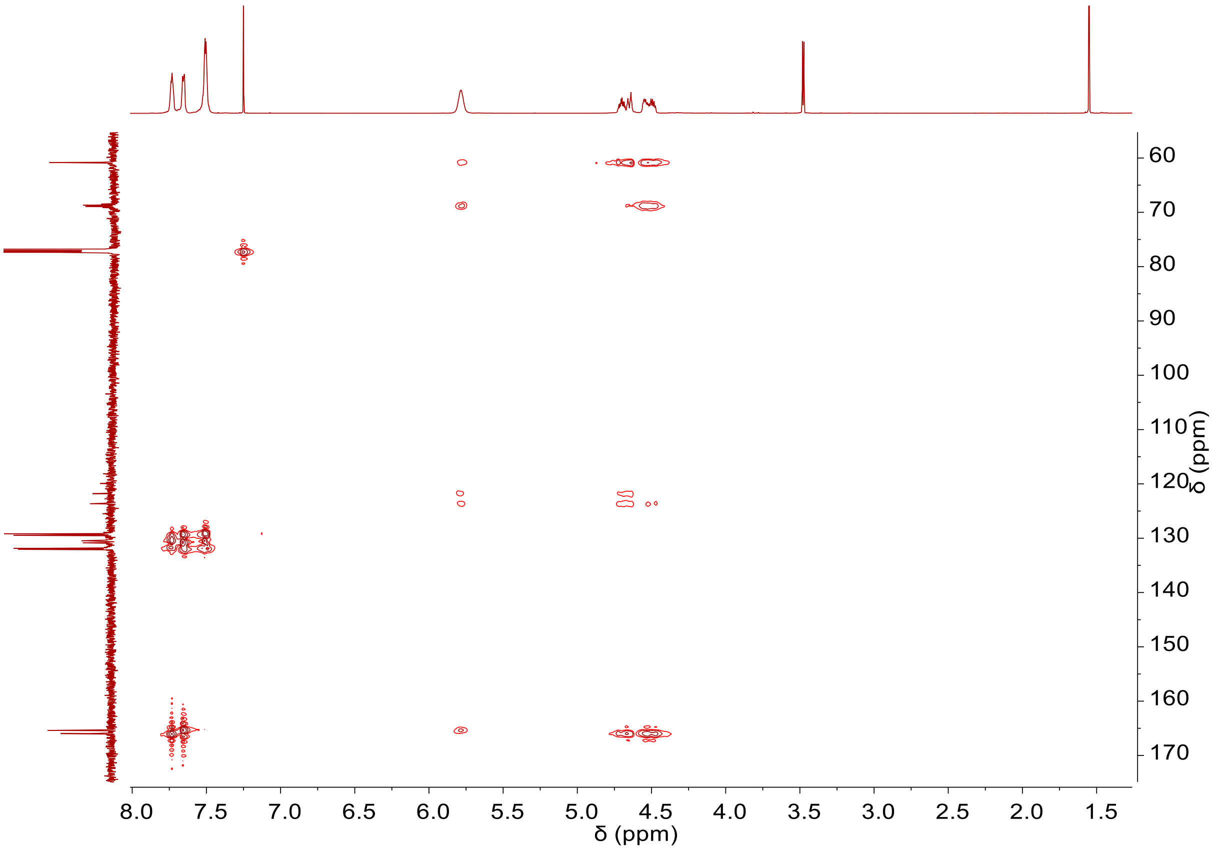

Figure S57 - ^{1}H-^{13}C HMBC NMR spectrum ($CDCl_3$) spectrum of the ^{F}PO/PA copolymer corresponding to Table S1 entry 5.

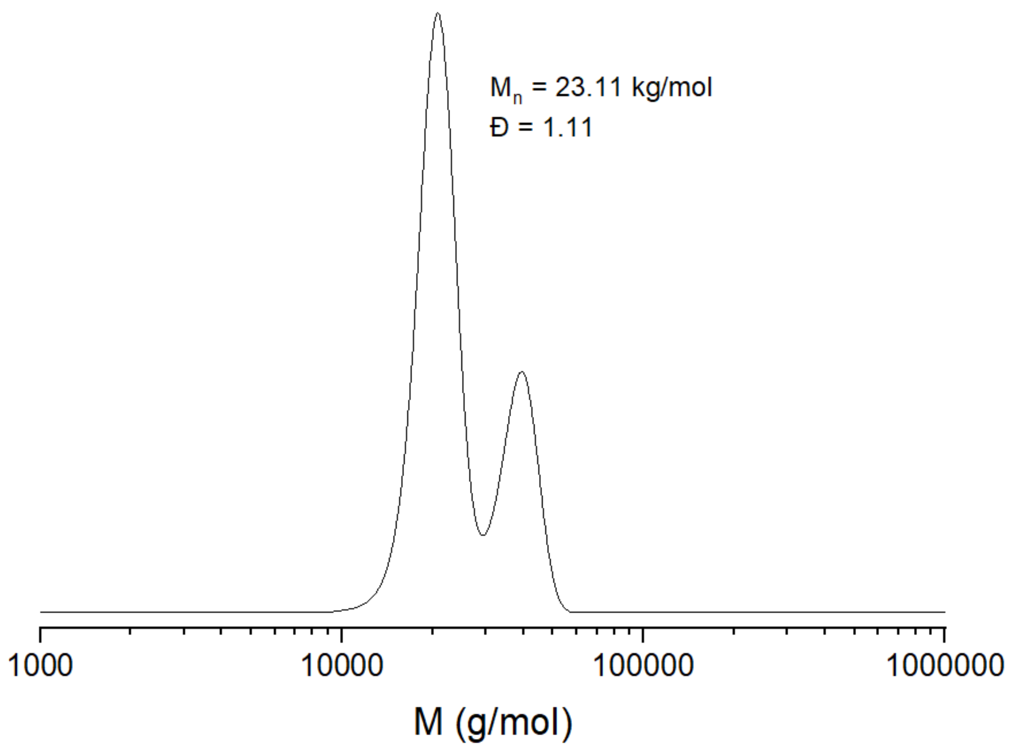

Figure S58 - GPC trace of ^{F}PO/PA copolymer corresponding to Table S1 entry 5.

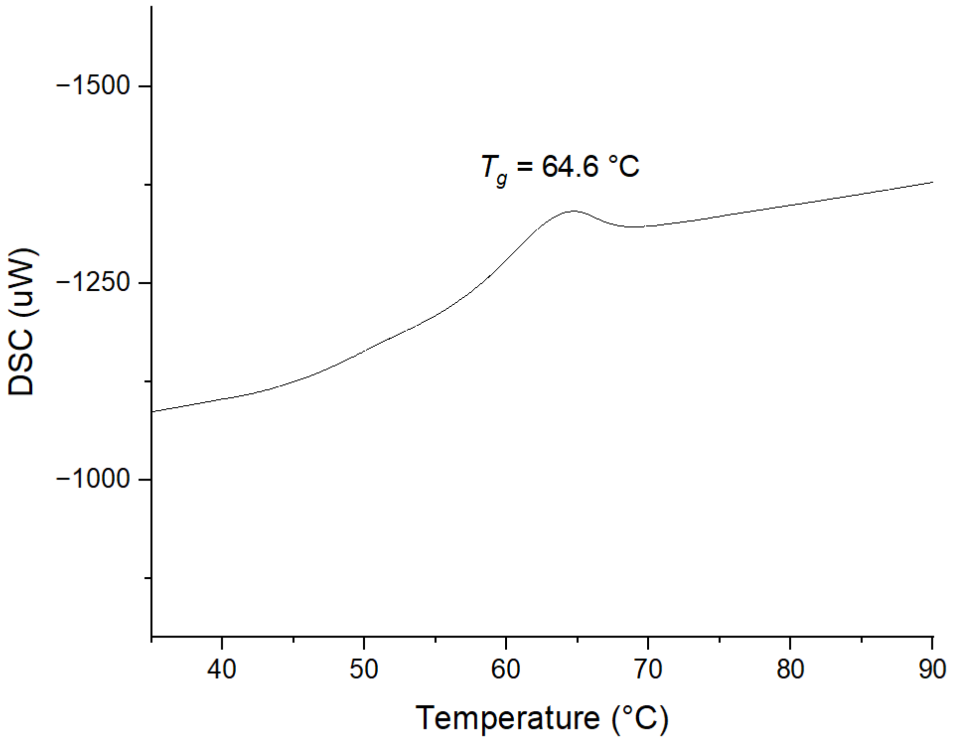

Figure S59 - DSC data from the second heating cycle of ^{F}PO/PA copolymer corresponding to Table S1 entry 5.

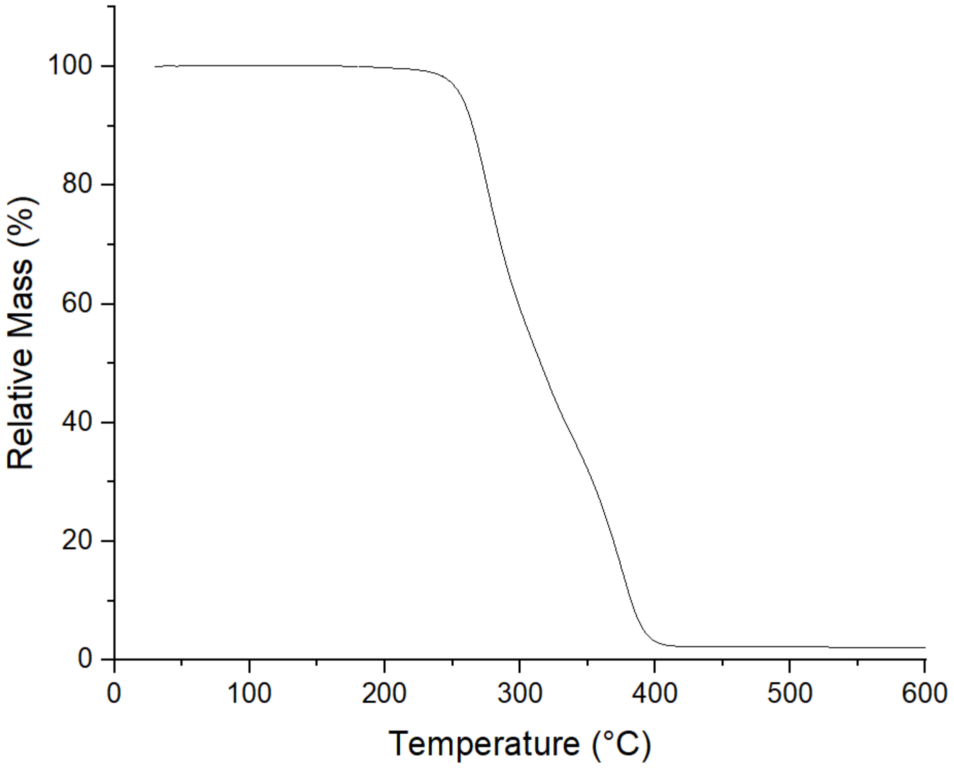

Figure S60 - TGA data of copolymer of [F]PO/PA copolymer $T_{d,5\%}$ = 257.0 ºC.

Section S8: Kinetics

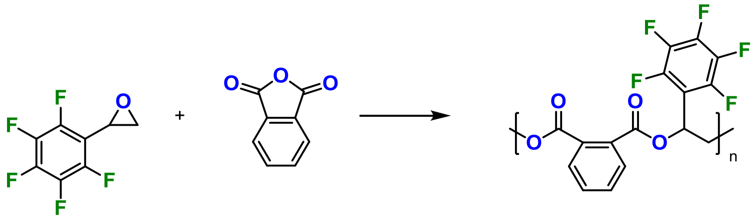

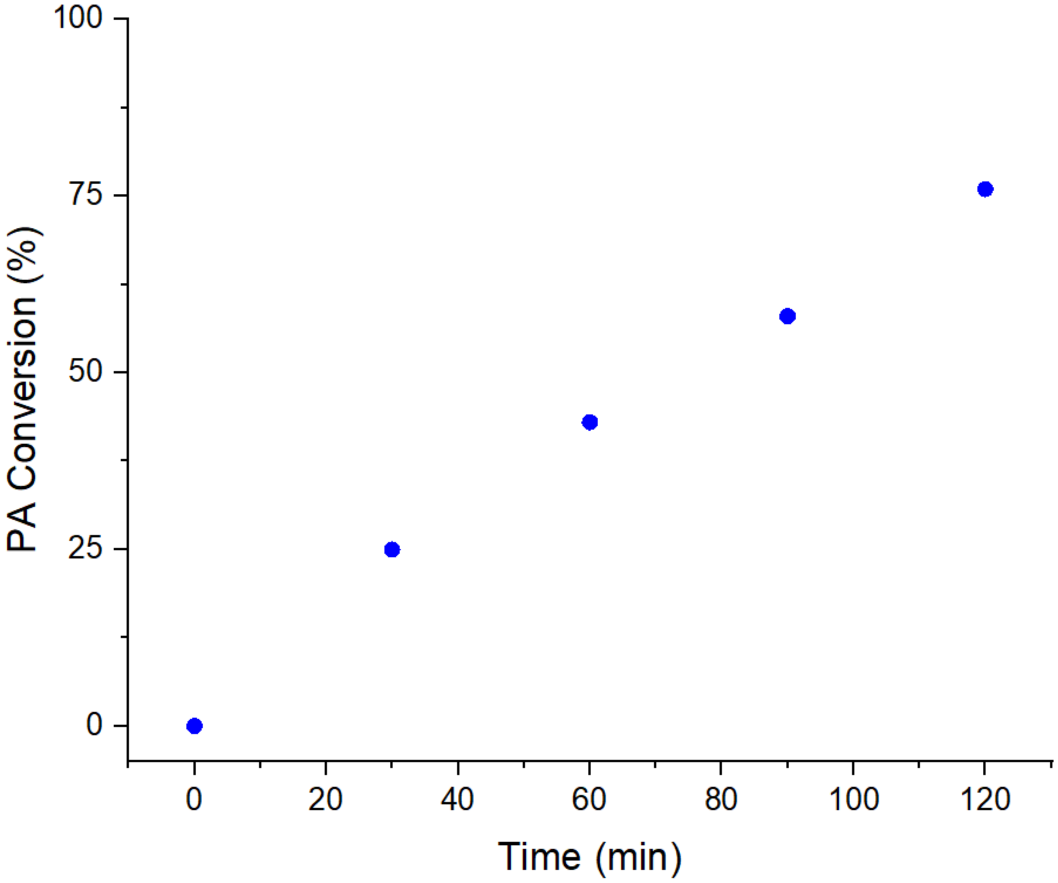

Figure S61 – Kinetic data for the ^{F}SO/PA copolymerisation 1 Cat.: 1 Cocat.: 500 ^{F}SO: 500 PA at 80 °C.

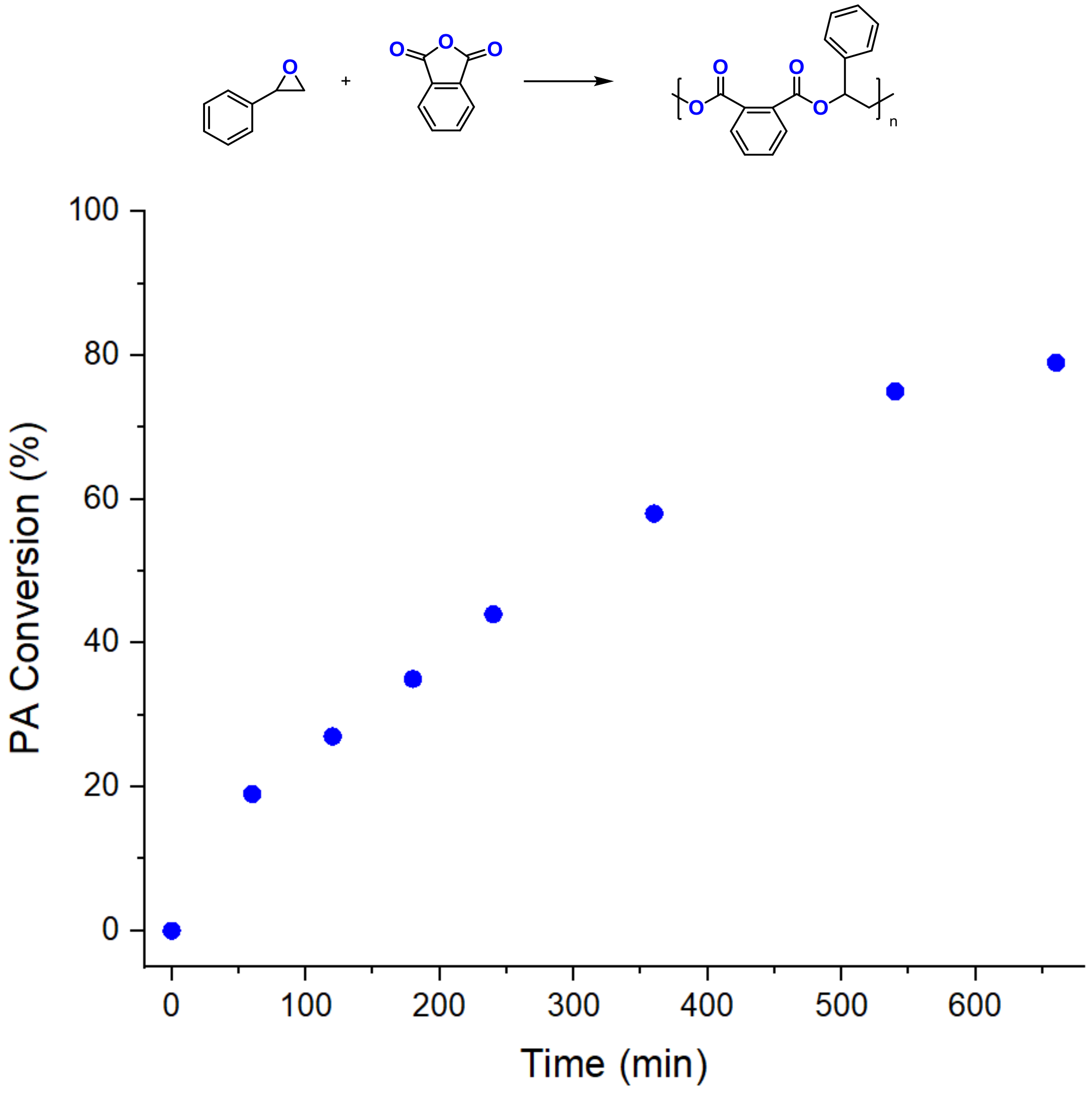

Figure S62 - Kinetic data for the SO/PA copolymerisation 1 Cat.: 1 Cocat.: 500 SO: 500 PA at 80 °C.

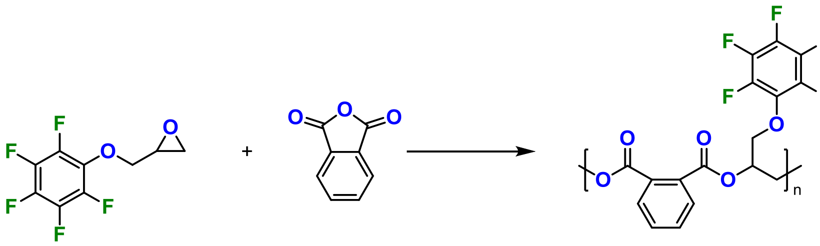

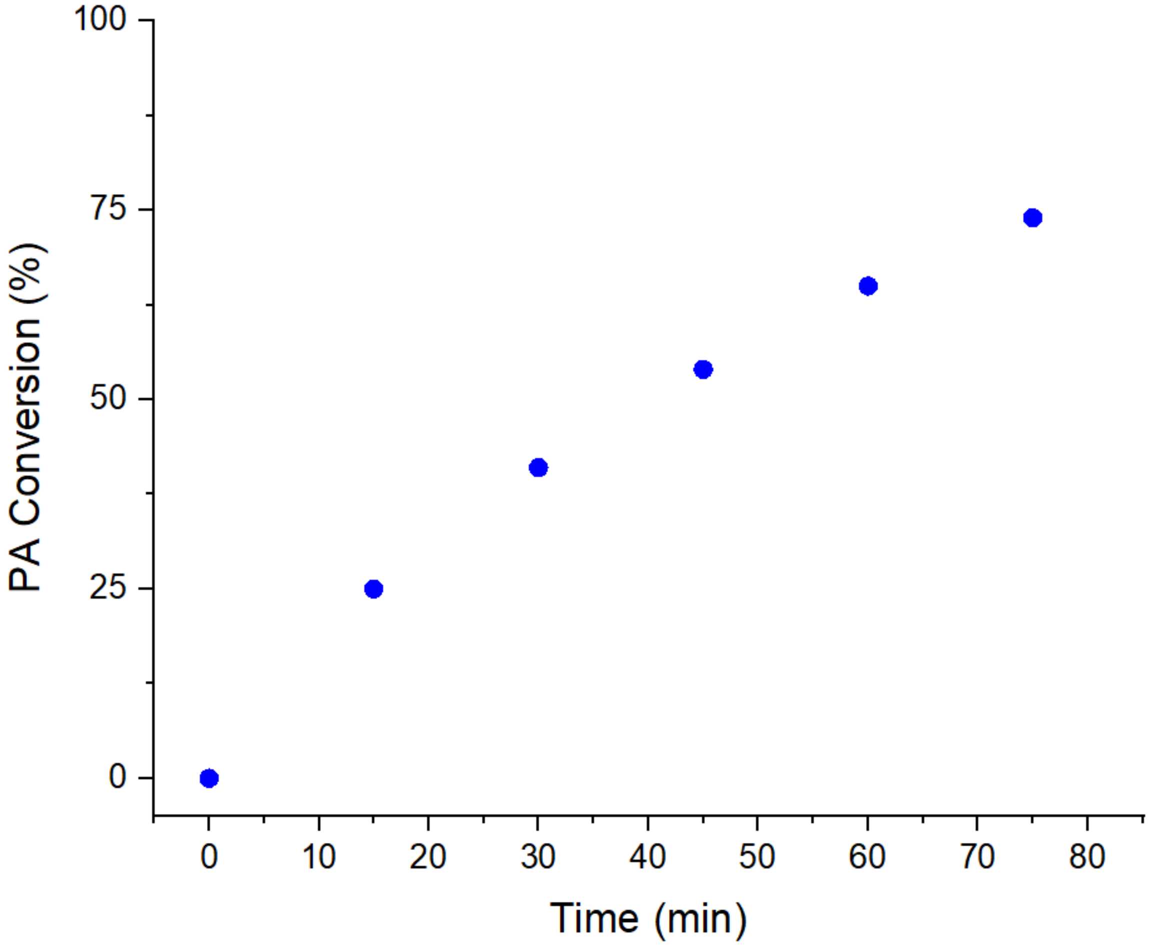

Figure S63 - Kinetic data for the ^{F}PhGE/PA copolymerisation 1 Cat.: 1 Cocat.: 500 ^{F}PhGE: 500 PA at 80 °C.

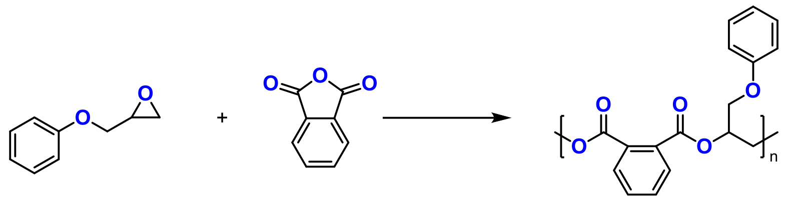

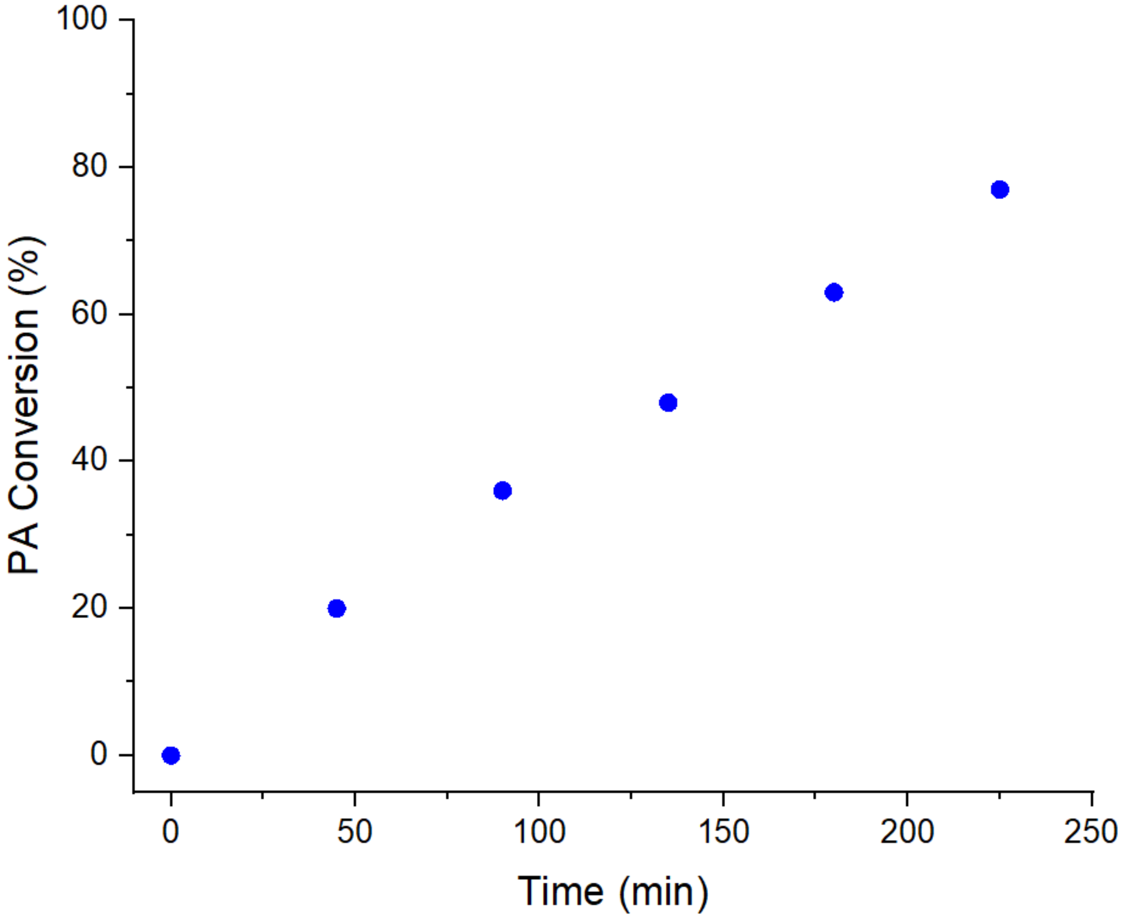

Figure S64 - Kinetic data for the PhGE/PA copolymerisation 1 Cat.: 1 Cocat.: 500 PhGE: 500 PA at 80 °C.

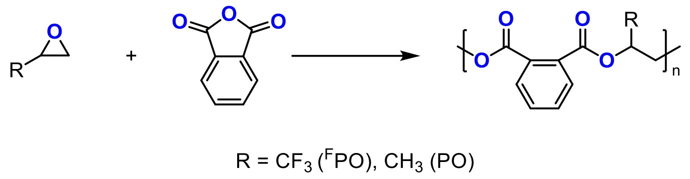

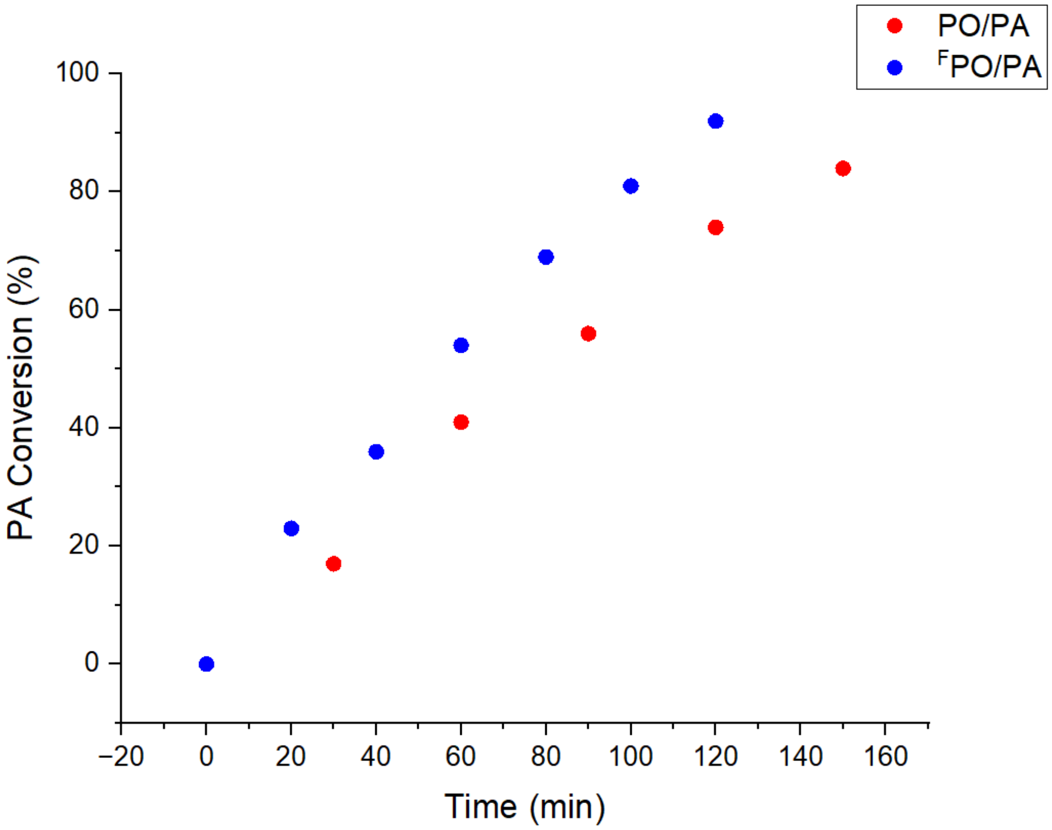

Figure S65 - Kinetic data for the (F)PO/PA copolymerisation 1 Cat.: 1 Cocat.: 500 (F)PO: 500 PA at 80 °C. Kinetic data for PO/PA taken from our previously published work.[7b]

Section S9: Post-polymerisation Modification

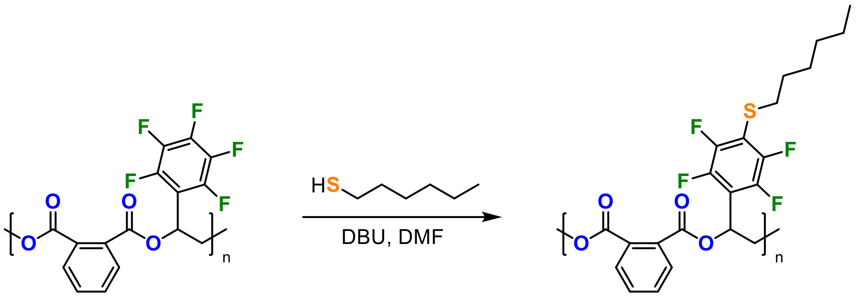

Figure S66 – Postfunctionalisation of ^{F}PO/PA copolymer with 1-hexanethiol.

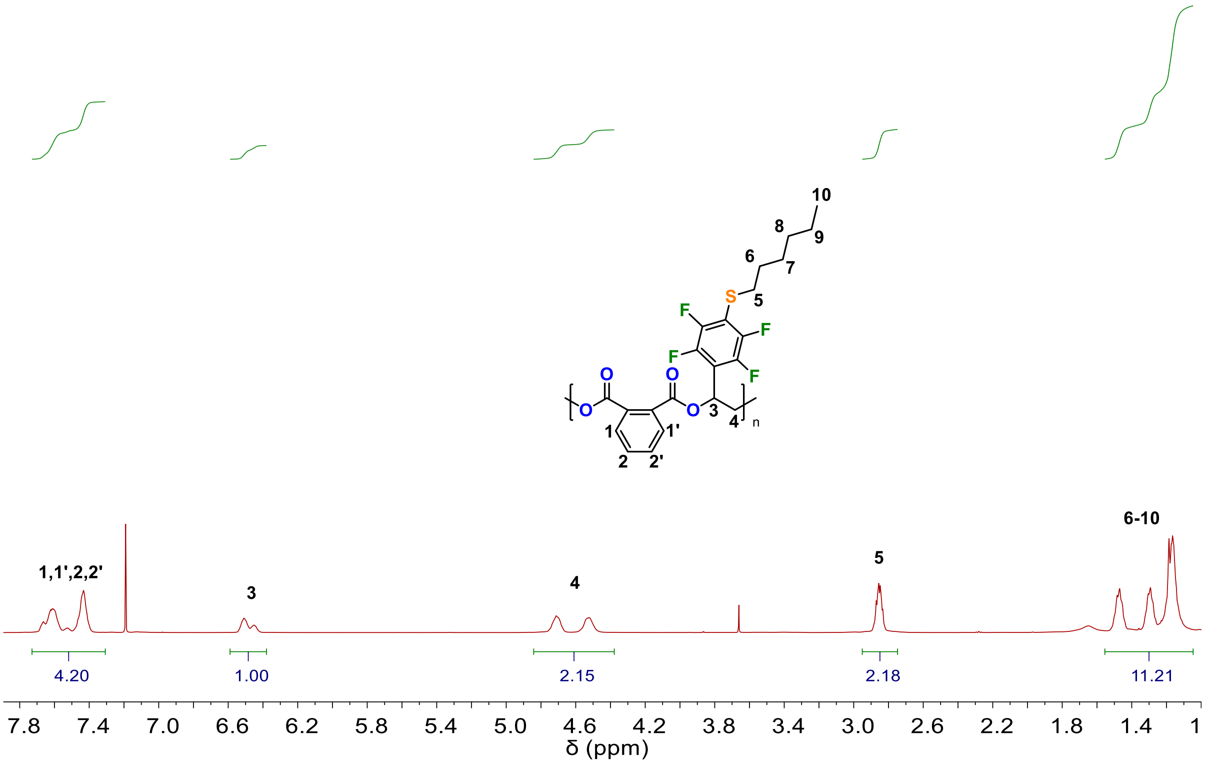

Figure S67 - ^{1}H NMR (500 MHz, $CDCl_3$) of ^{F}PO/PA copolymer after post-functionalisation with 1-hexanethiol.

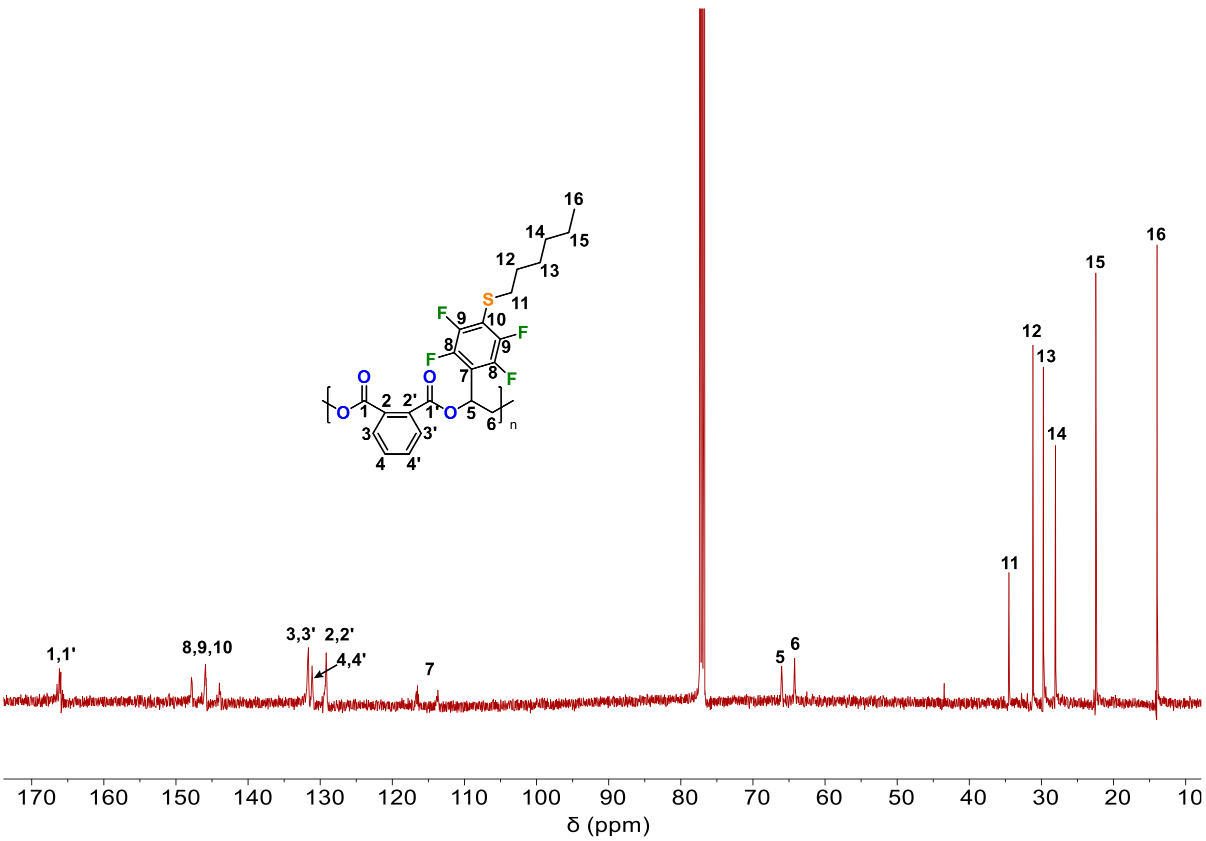

Figure S68 - ^{13}C NMR spectrum (126 MHz, $CDCl_3$) of ^{F}PO/PA copolymerisation after post-functionalisation with 1-hexanethiol.

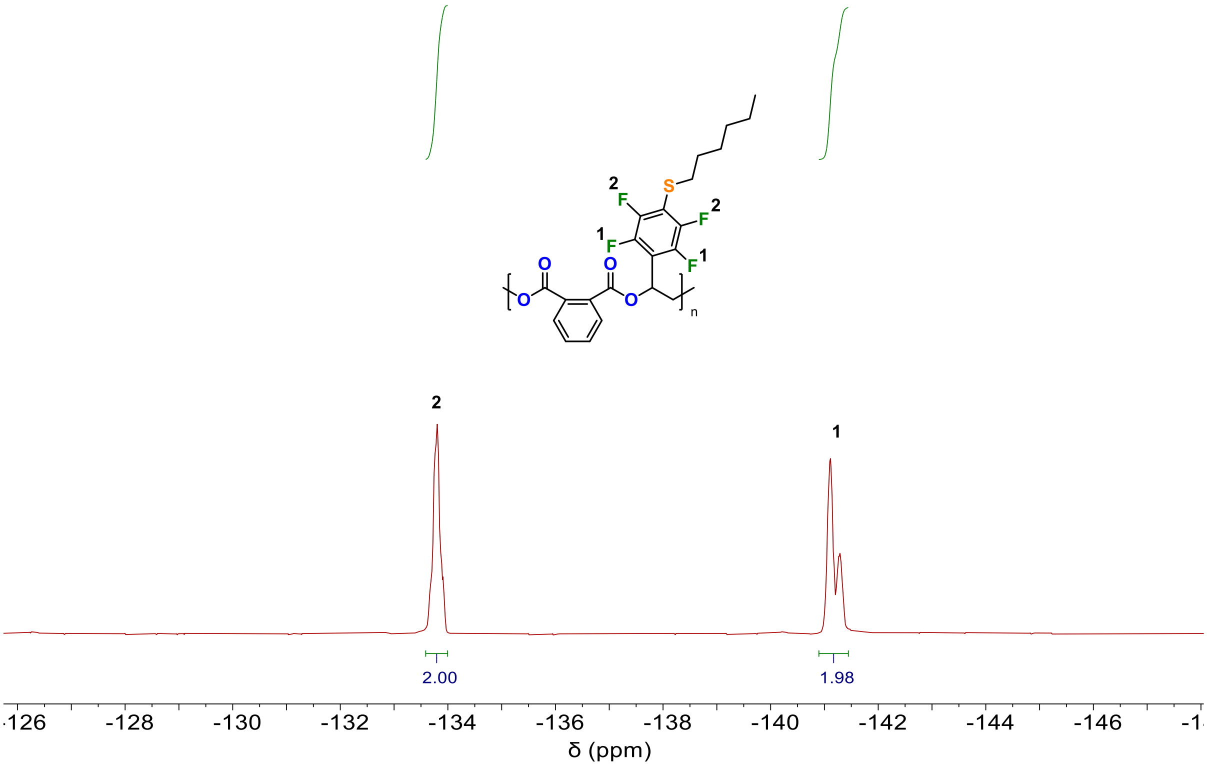

Figure S69 - ^{19}F NMR spectrum (376 MHz, $CDCl_3$) of ^{F}PO/PA copolymerisation after post-functionalisation with 1-hexanethiol.

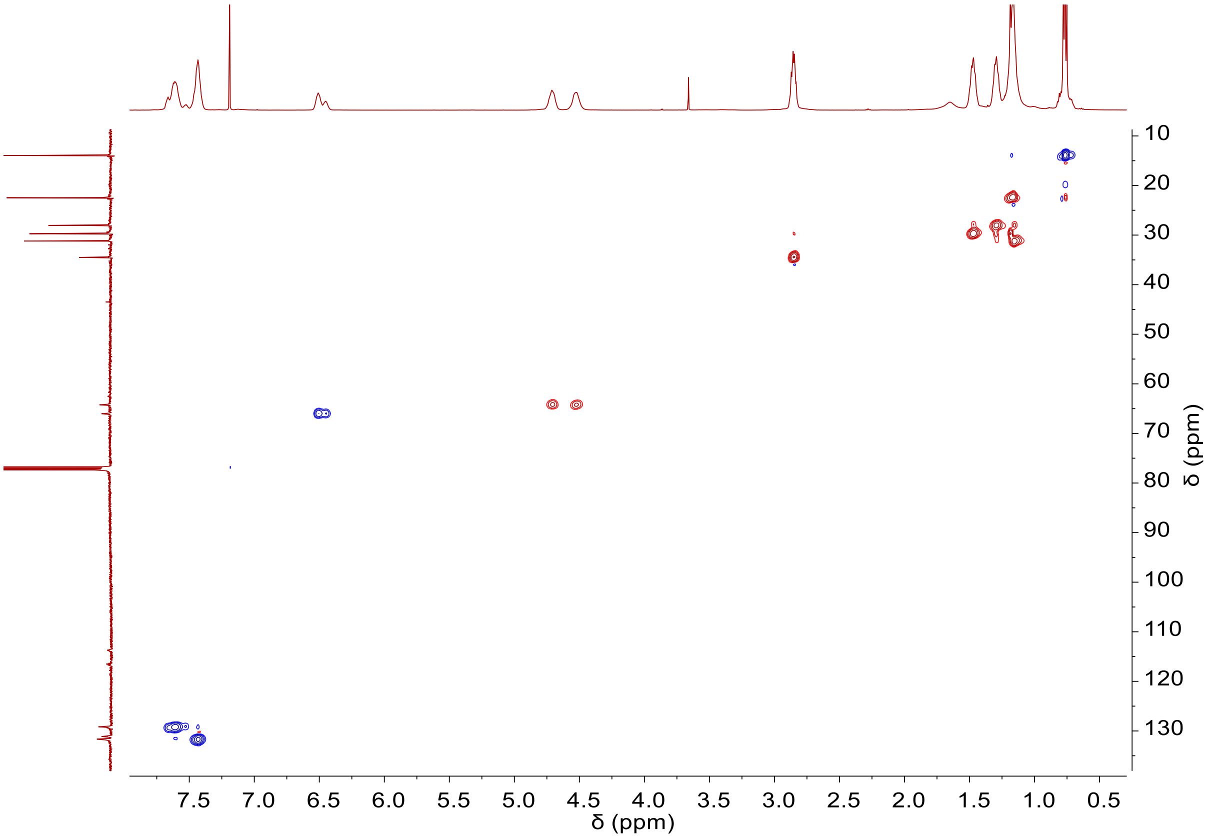

Figure S70 - ^{1}H-^{13}C HMBC NMR spectrum ($CDCl_3$) spectrum of ^{F}PO/PA copolymerisation after post-functionalisation with 1-hexanethiol.

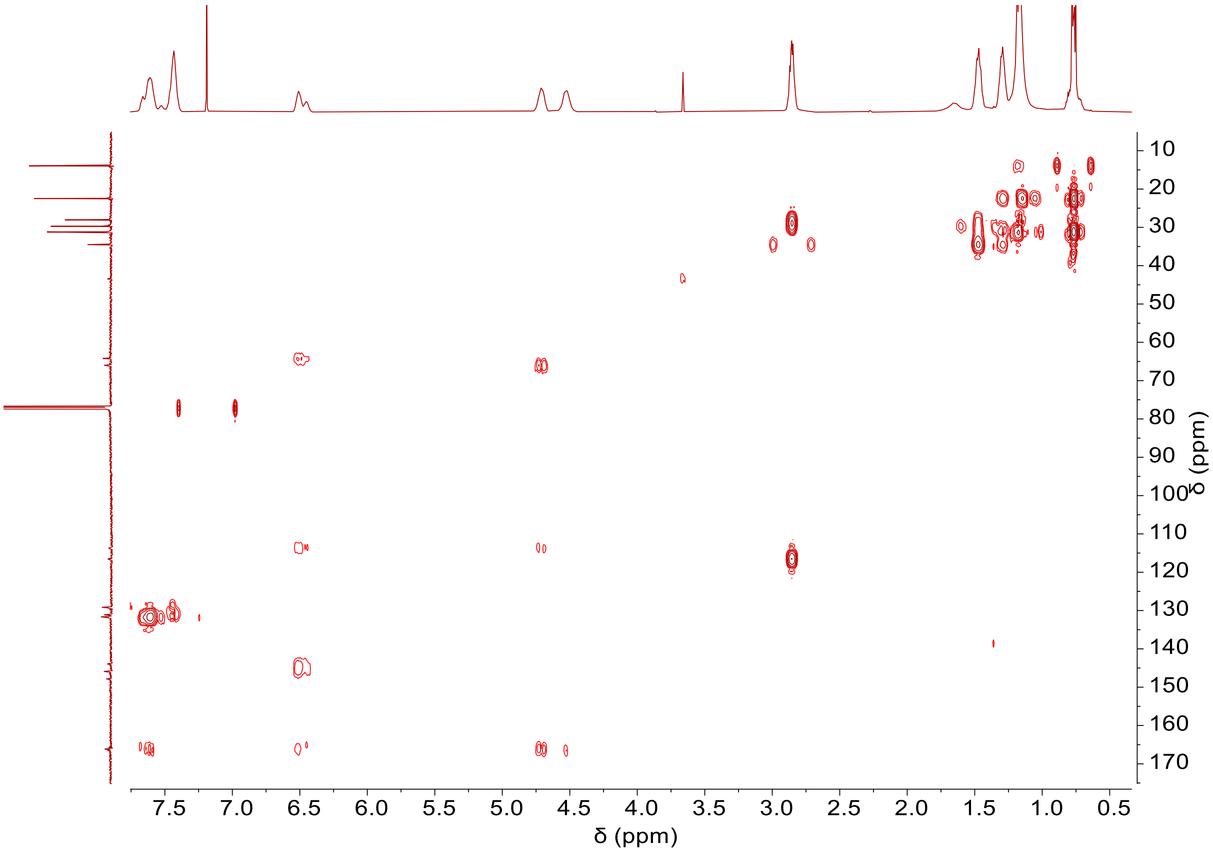

Figure S71 - ^{1}H-^{13}C HMQC NMR spectrum ($CDCl_3$) spectrum of ^{F}PO/PA copolymerisation after post-functionalisation with 1-hexanethiol.

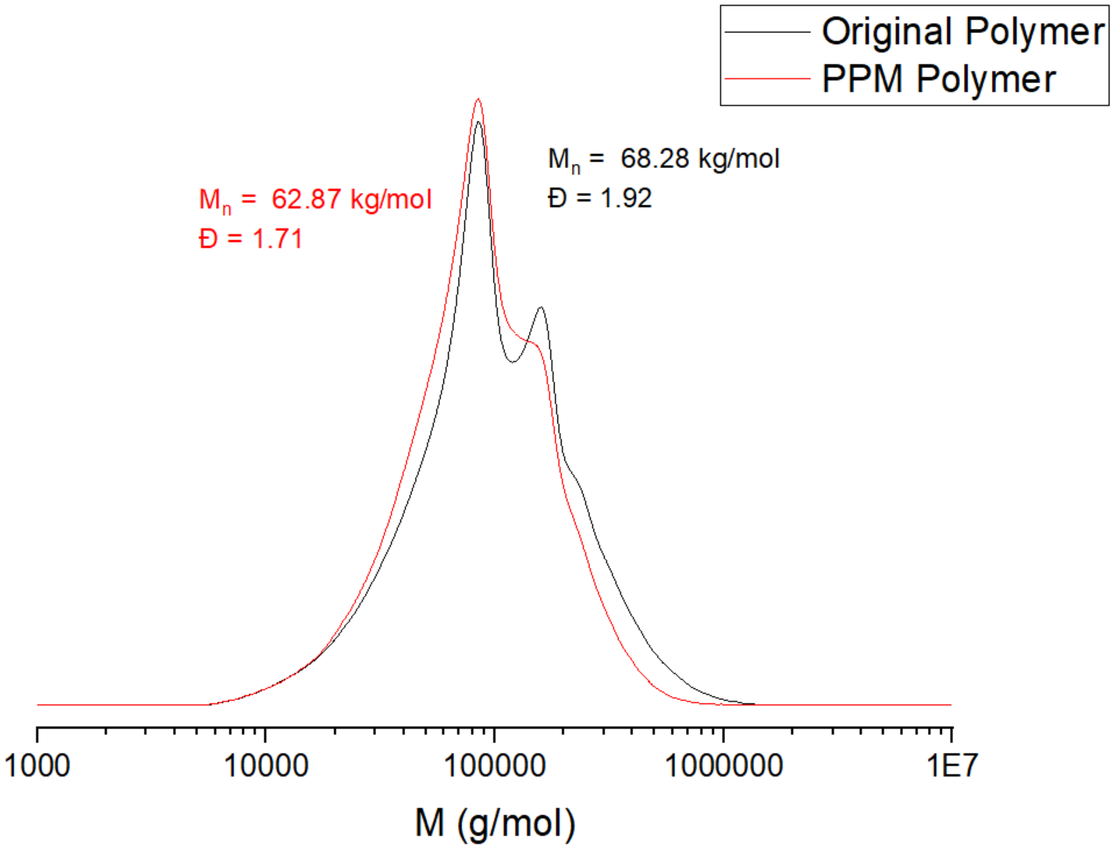

Figure S72 – GPC trace corresponding to ^{F}PO/PA copolymer before and after post-functionalisation with 1-hexanethiol.

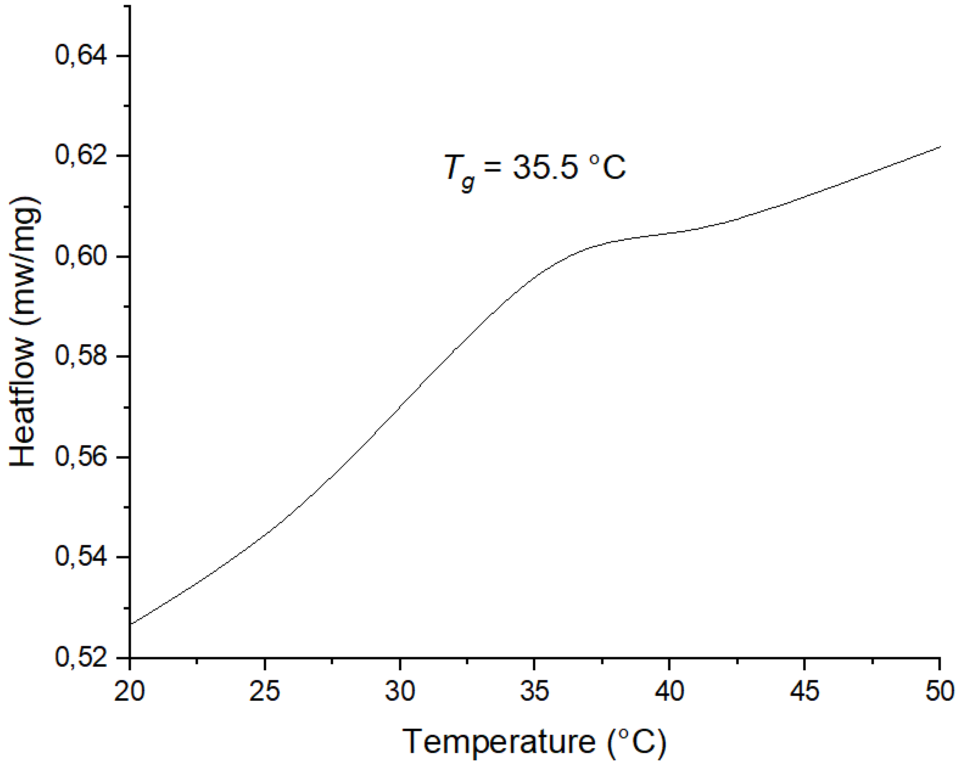

Figure S73 - DSC data from the second heating cycle corresponding to ^{F}SO/PA copolymer post-functionalised with hexane thiol.

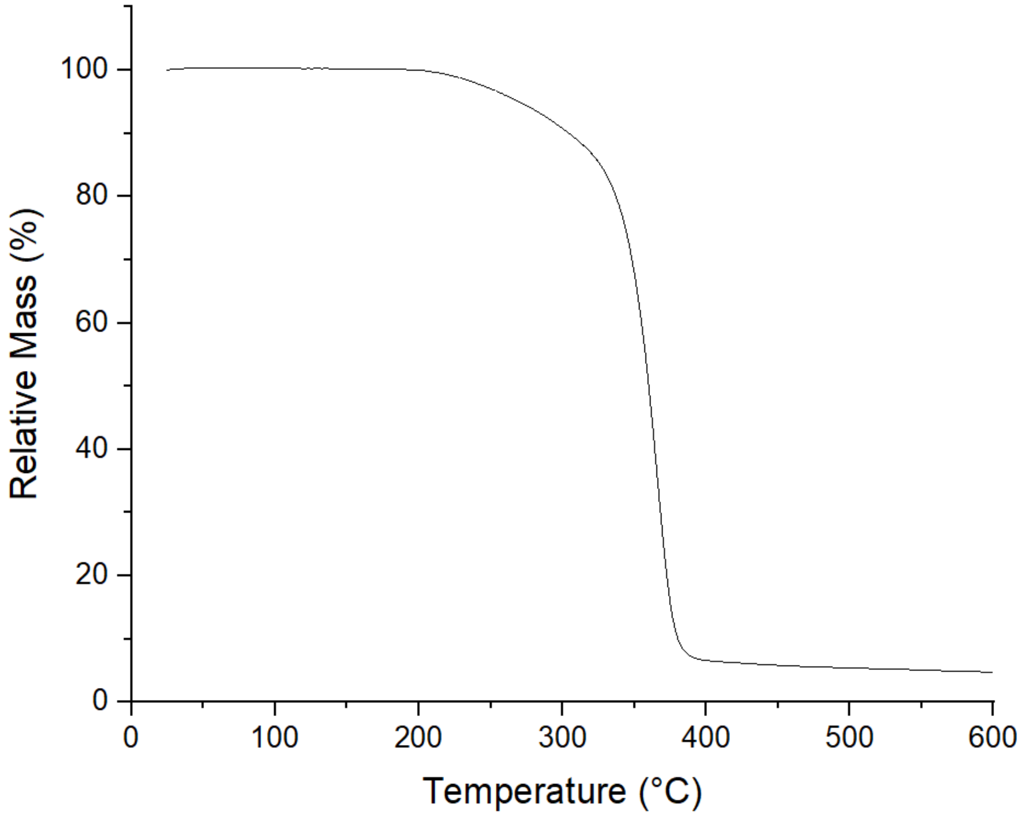

Figure S74 - TGA data of copolymer corresponding to ^{F}PO/PA copolymer post-functionalised with hexane thiol. $T_{d,5\%}$ = 269.8 ºC.

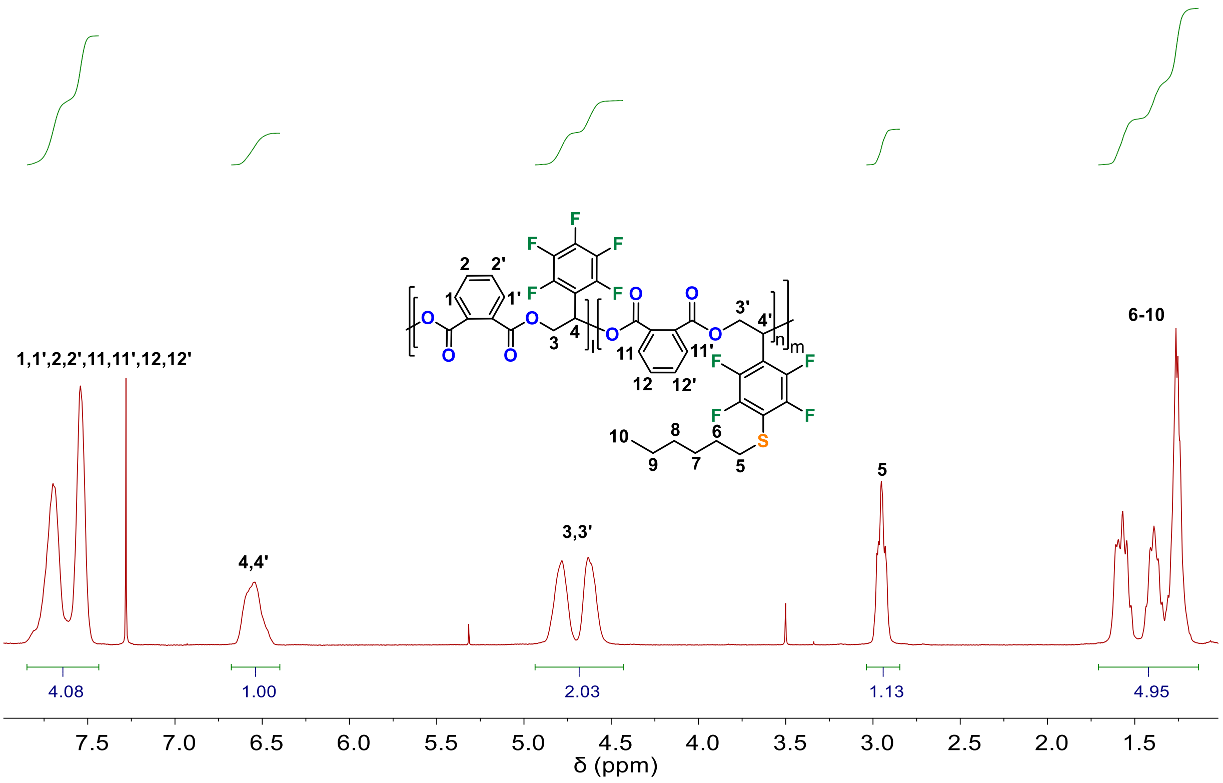

Figure S75 - ^{1}H NMR (500 MHz, $CDCl_3$) of ^{F}PO/PA copolymer after post-functionalisation with 0.5 equiv. 1-hexanethiol.

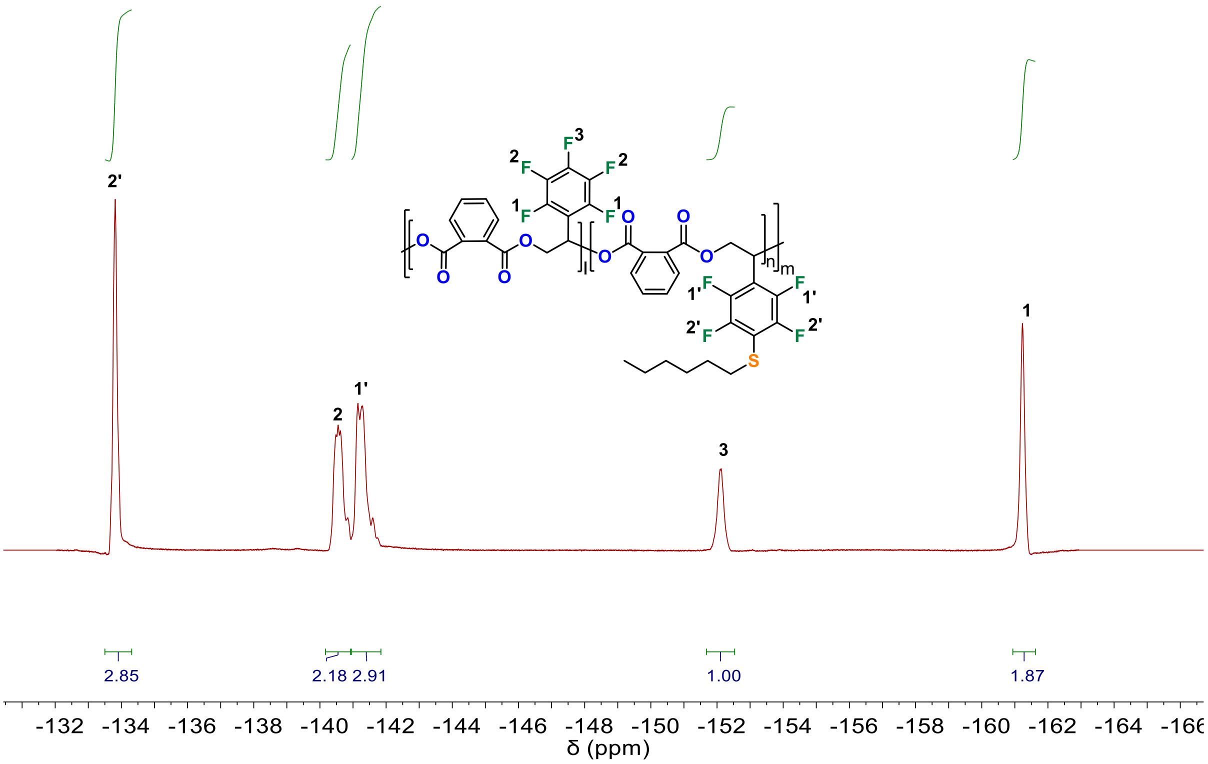

Figure S76 - ^{19}F NMR spectrum (376 MHz, $CDCl_3$) of ^{F}PO/PA copolymerisation after post-functionalisation with 0.5 equiv. 1-hexanethiol.

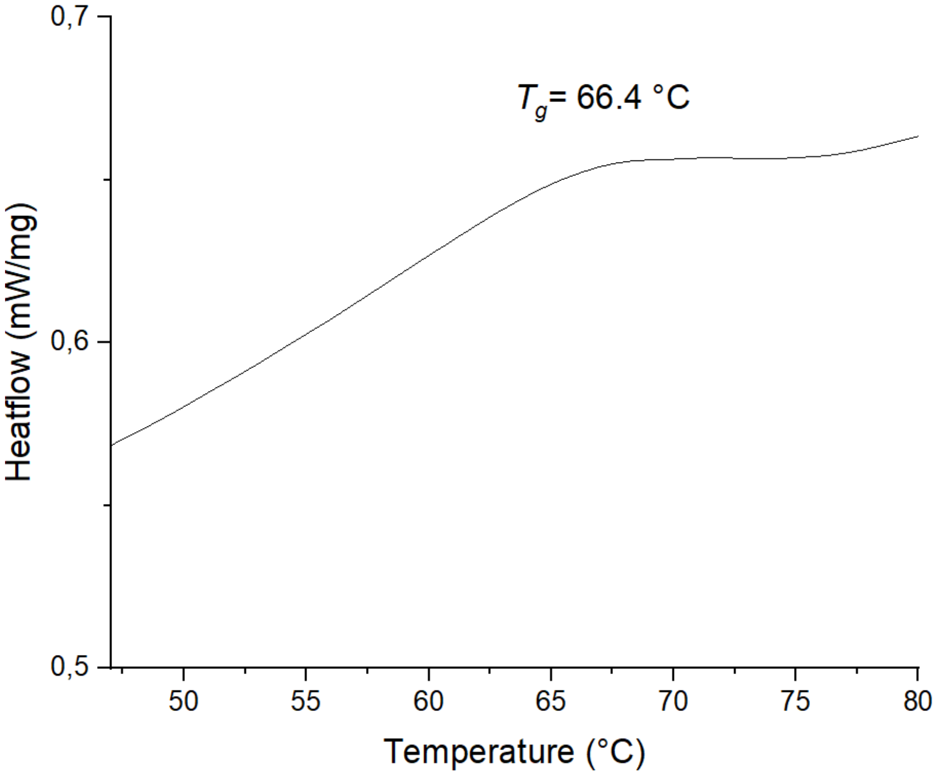

Figure S77 - DSC data from the second heating cycle corresponding to ^{F}SO/PA copolymer post-functionalised with 0.5 equiv. hexane thiol.

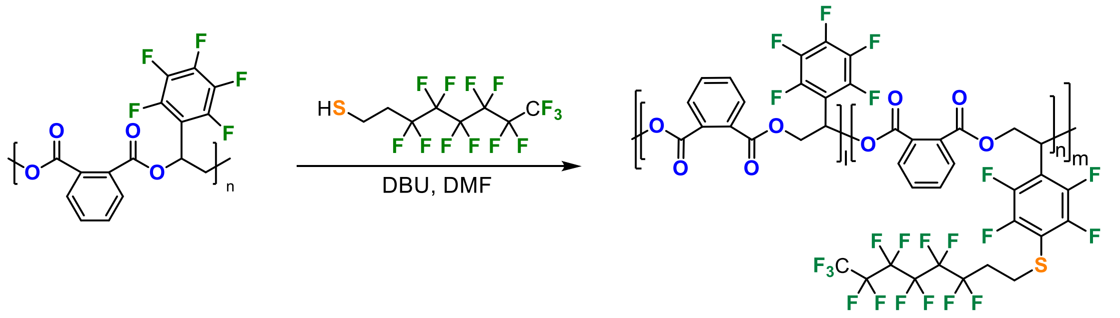

Figure S78 - Postfunctionalisation of ^{F}PO/PA copolymer with tridecafluor-1-octanthiol.

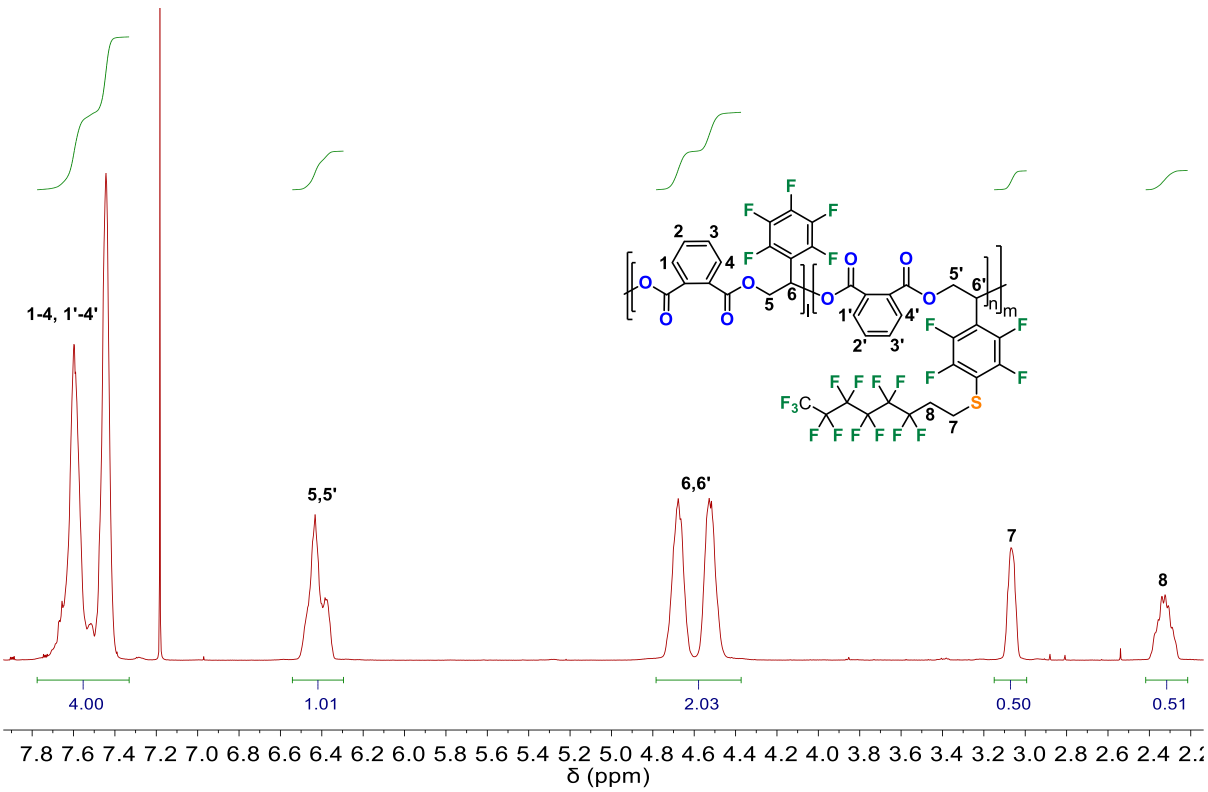

Figure S79 - ^{1}H NMR (500 MHz, $CDCl_3$) of ^{F}PO/PA copolymerisation after post-functionalisation with perfluor-1-octanthiol.

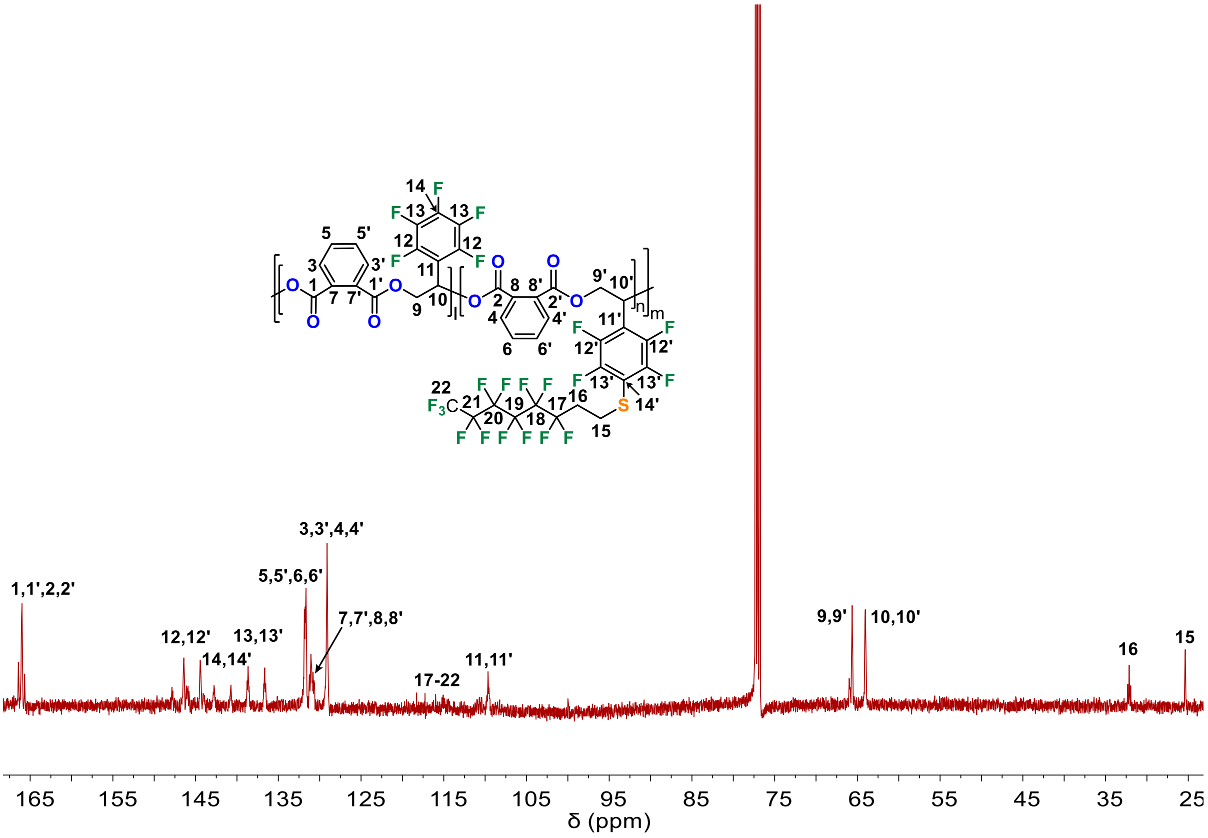

Figure S80 - ^{13}C NMR spectrum (126 MHz, $CDCl_3$) of ^{F}PO/PA copolymerisation after post-functionalisation with perfluor-1-octanthiol.

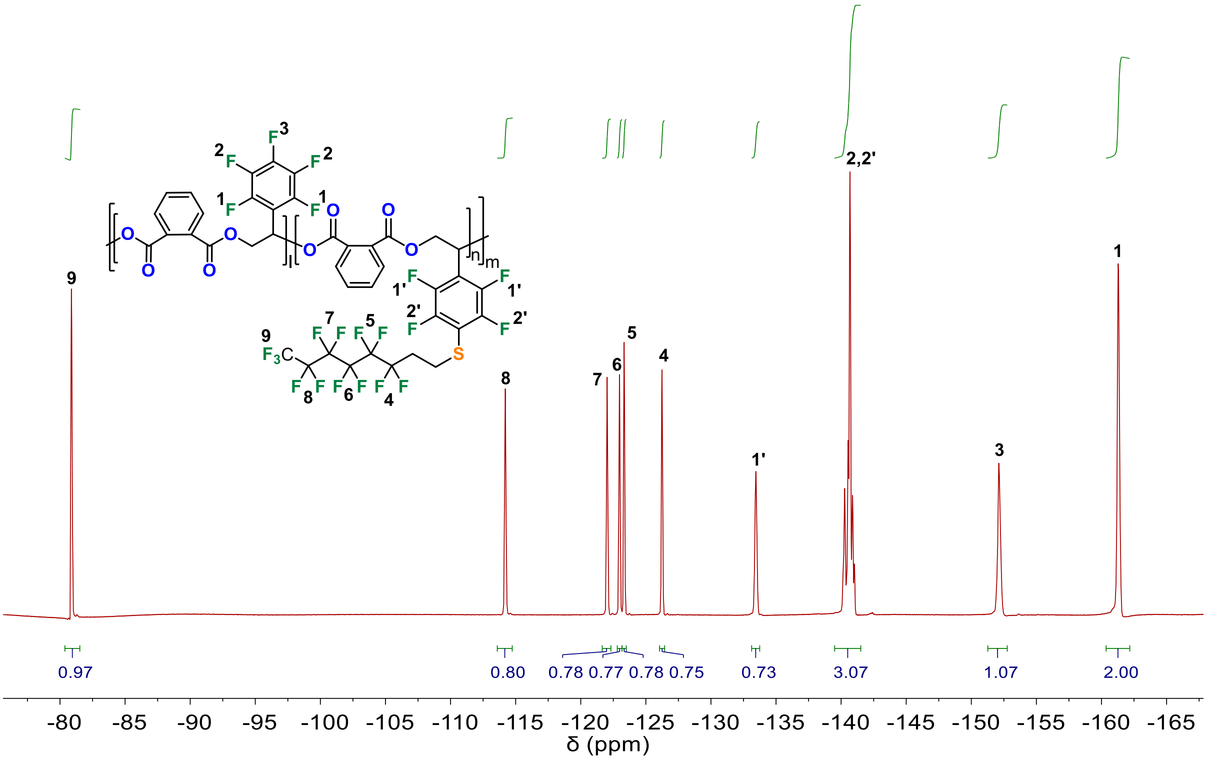

Figure S81 - ^{19}F NMR spectrum (376 MHz, $CDCl_3$) of ^{F}PO/PA copolymerisation after post-functionalisation with perfluor-1-octanthiol.

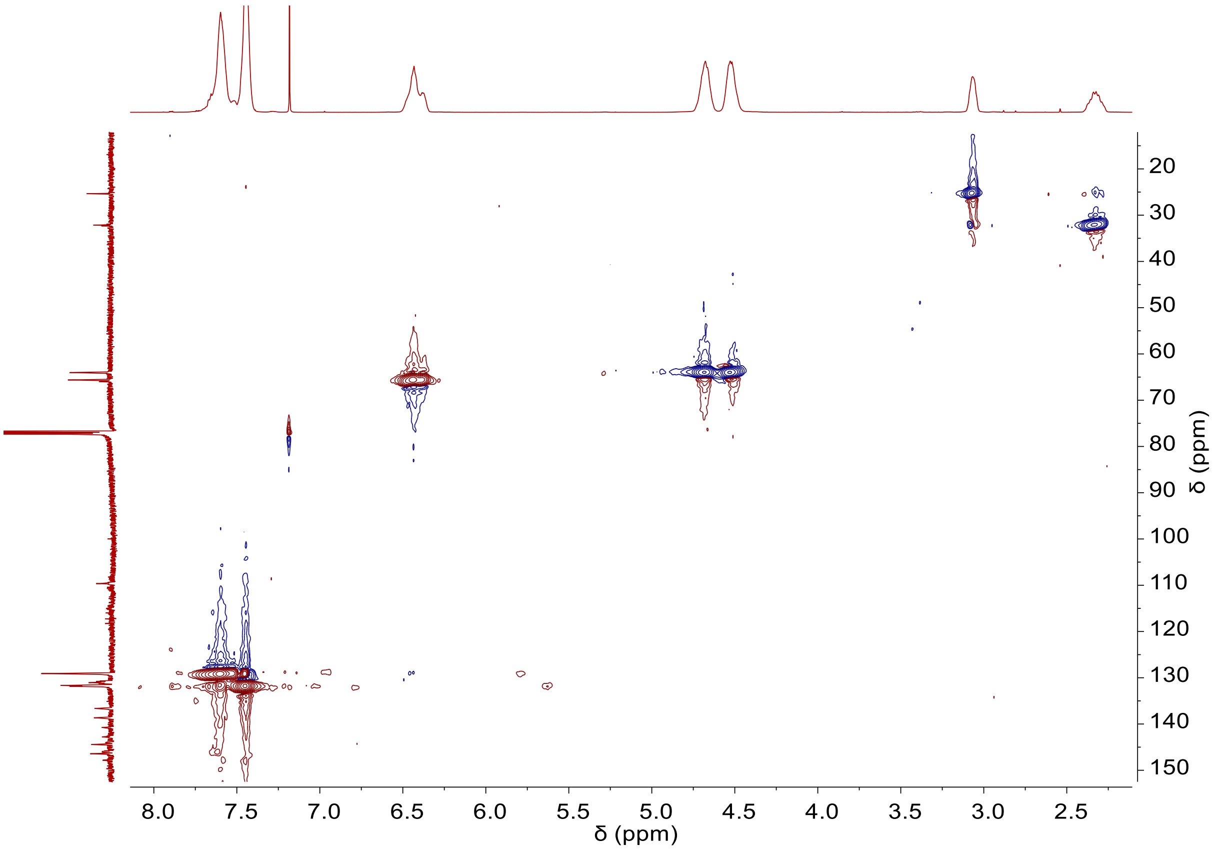

Figure S82 - ^{1}H-^{13}C HSQC NMR spectrum ($CDCl_3$) of ^{F}PO/PA copolymerisation after post-functionalisation with perfluor-1-octanthiol.

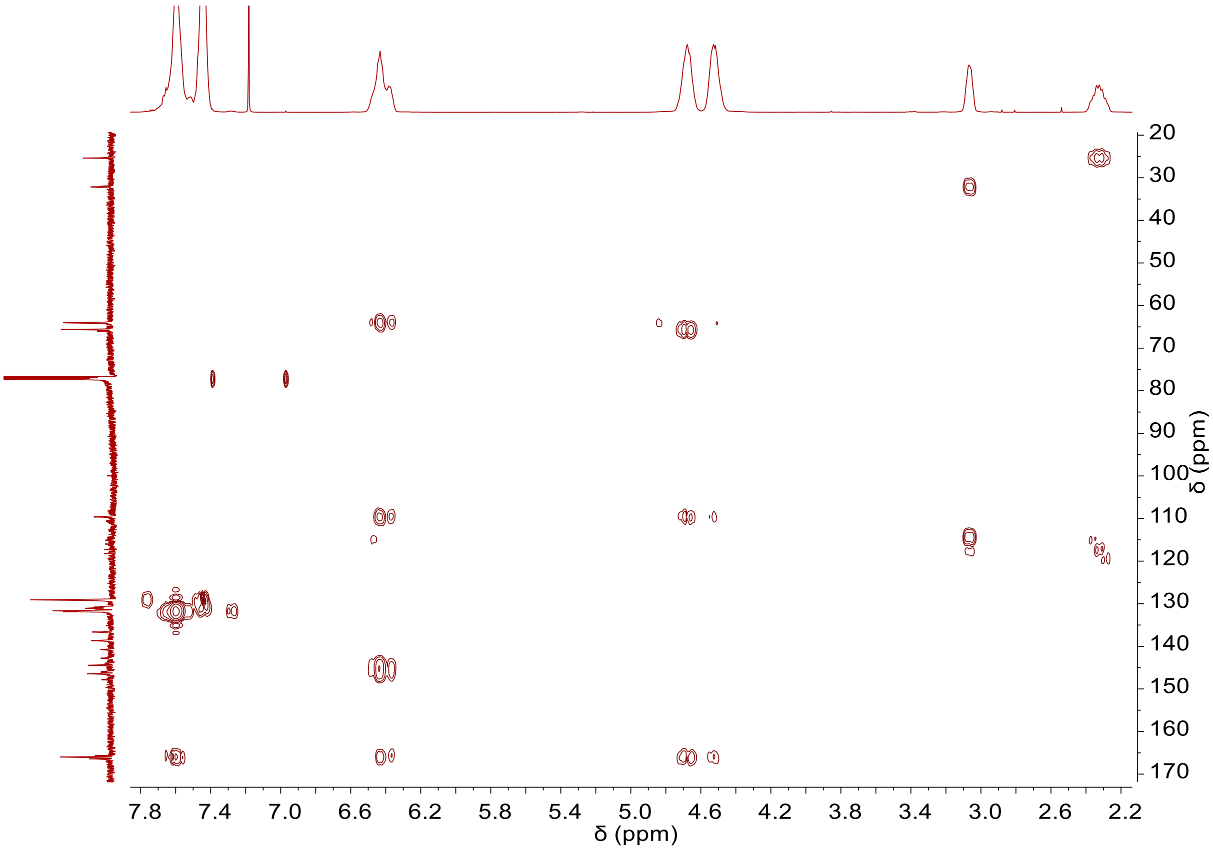

Figure S83 - ^{1}H-^{13}C HMQC NMR spectrum ($CDCl_3$) of ^{F}PO/PA copolymerisation after post-functionalisation with perfluor-1-octanthiol.

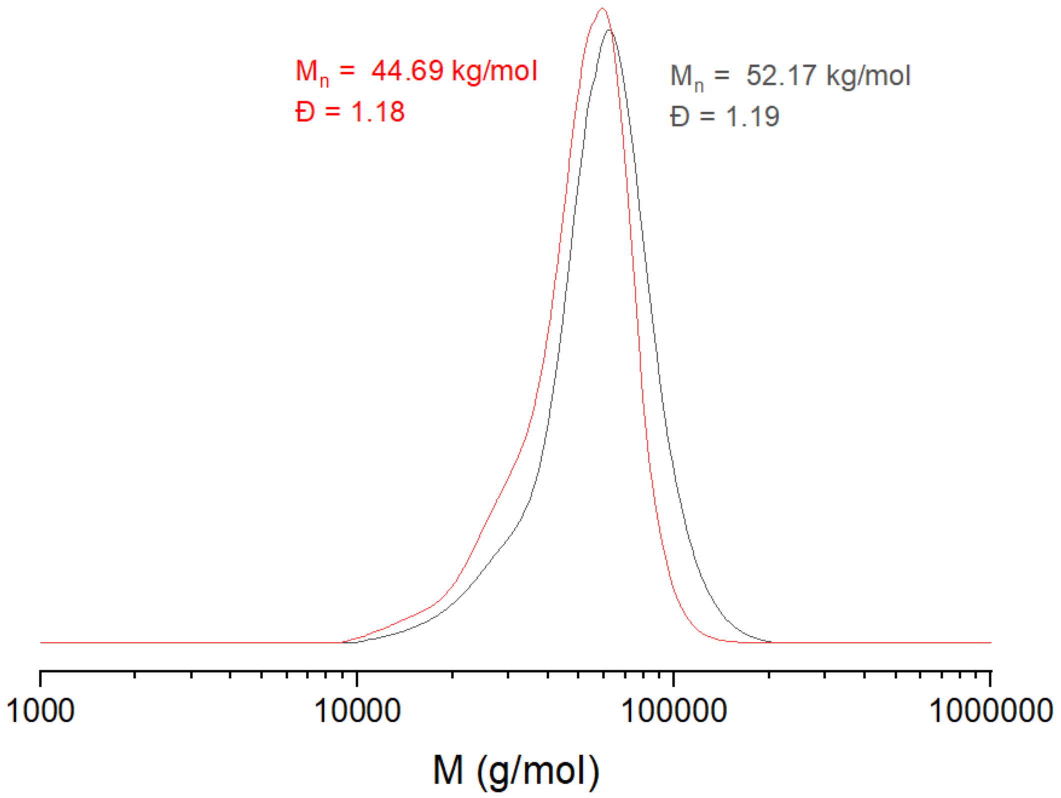

Figure S84 - GPC trace of ^{F}PO/PA copolymer after post-functionalisation with perfluor-1-octanthiol (red) vs original ^{F}PO/PA copolymer (black).

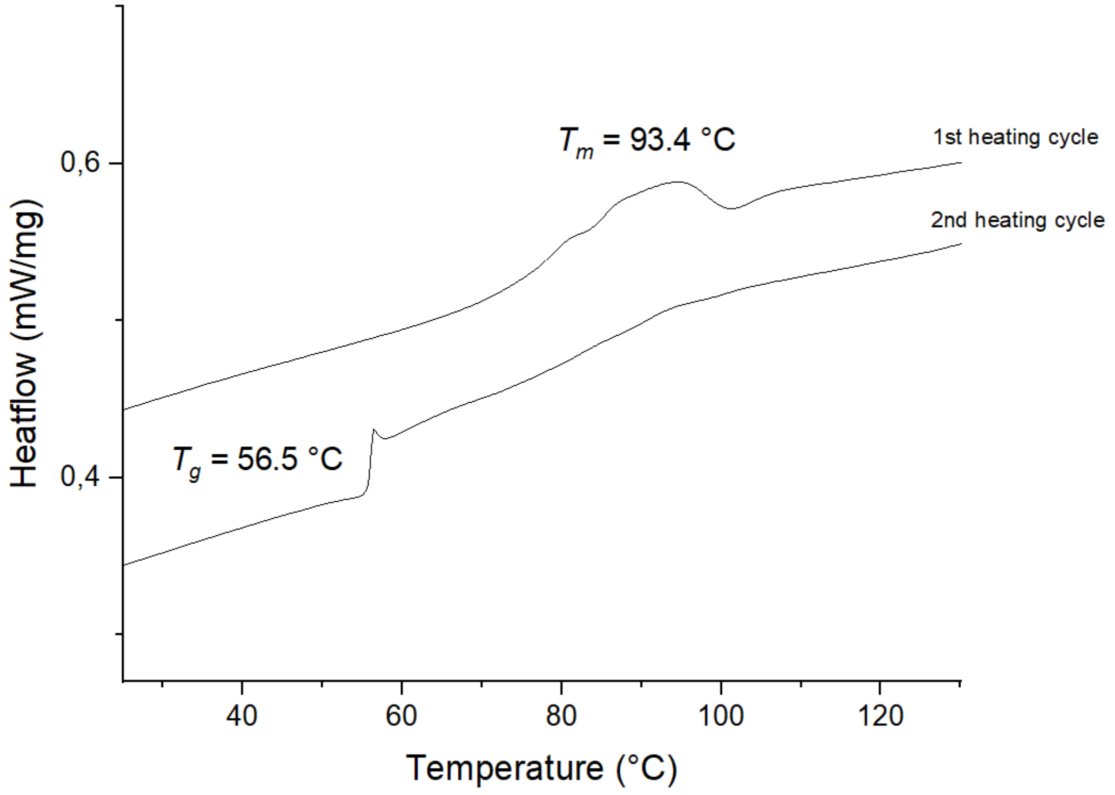

Figure S85 - DSC data from the first and second heating cycle corresponding to ^{F}PO/PA copolymer after post-functionalisation with perfluor-1-octanthiol.

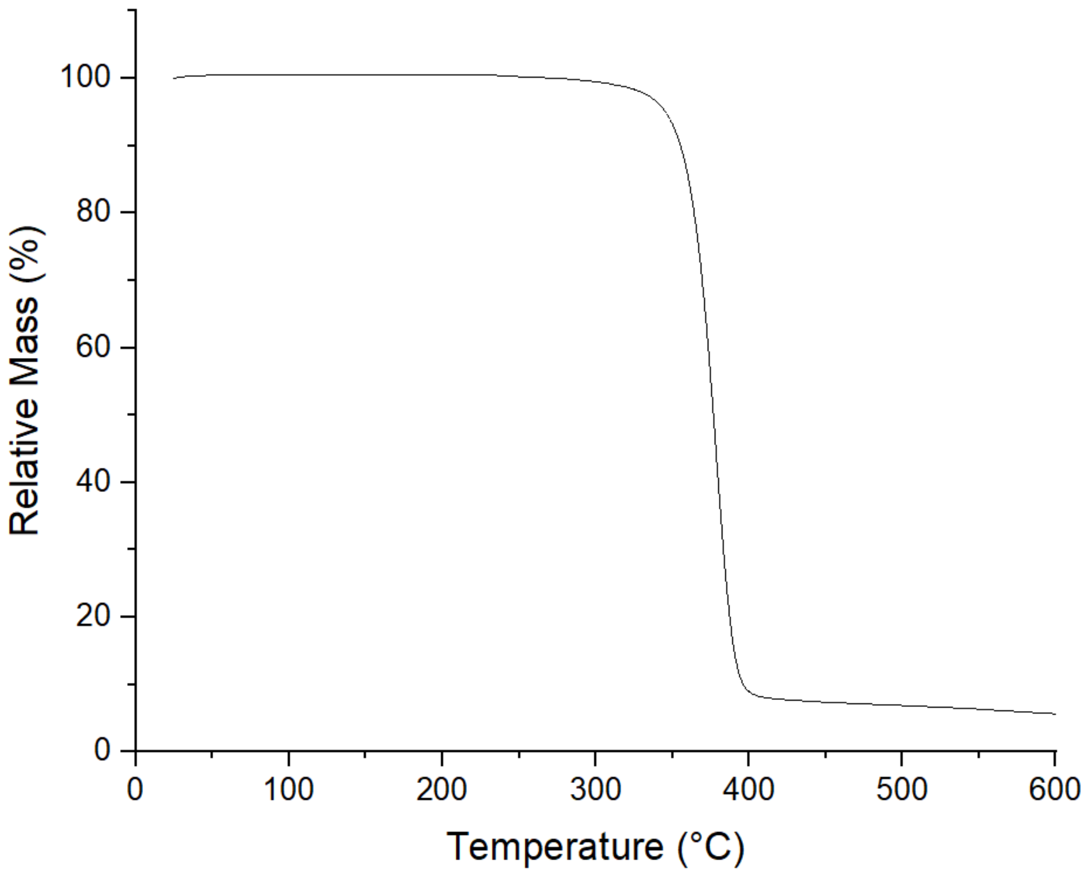

Figure S86 - TGA data of copolymer corresponding to ^{F}PO/PA copolymer post-functionalised with perfluor-1-octanthiol $T_{d,5\%}$ = 345.7 ºC.

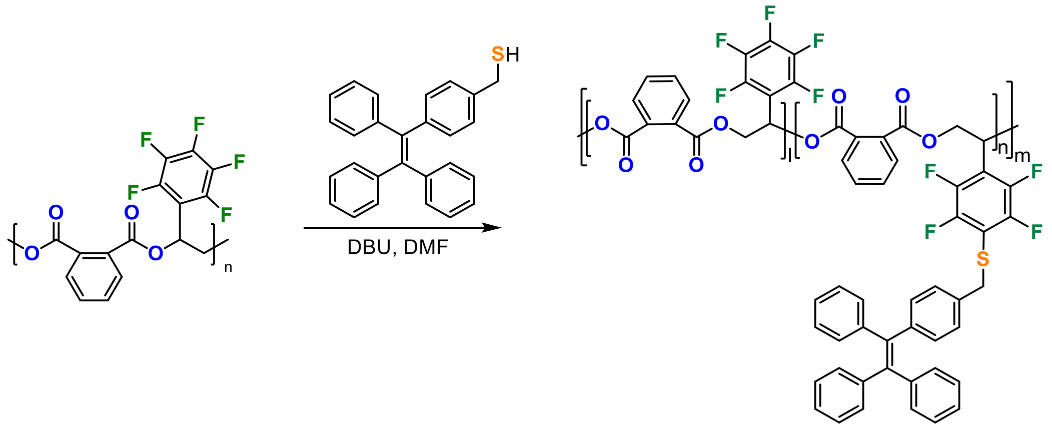

Figure S87 - Postfunctionalisation of ^{F}PO/PA copolymer with 4-(1,2,2-triphenylethenyl)benzenemethanethiol.

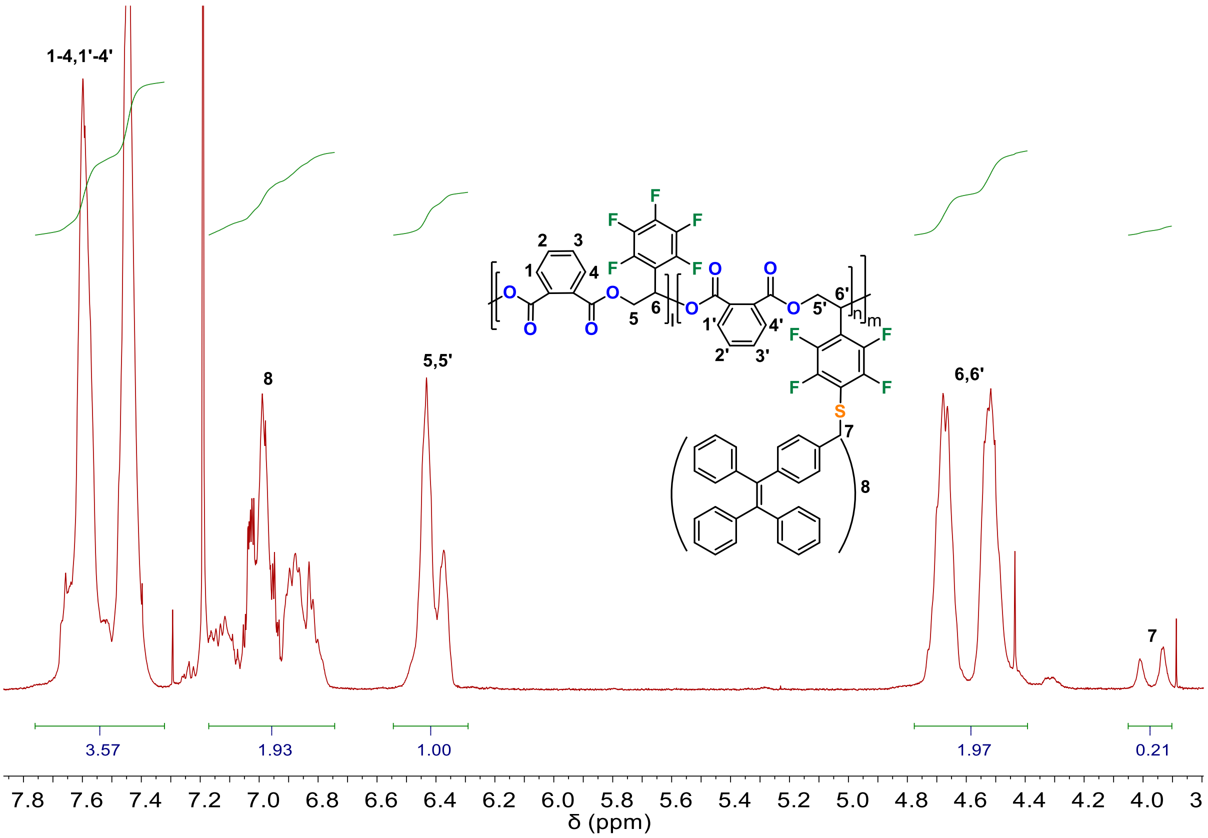

Figure S88 - ^{1}H NMR (500 MHz, $CDCl_3$) of post-functionalised ^{F}PO/PA copolymer with 4-(1,2,2-triphenylethenyl)benzenemethanethiol.

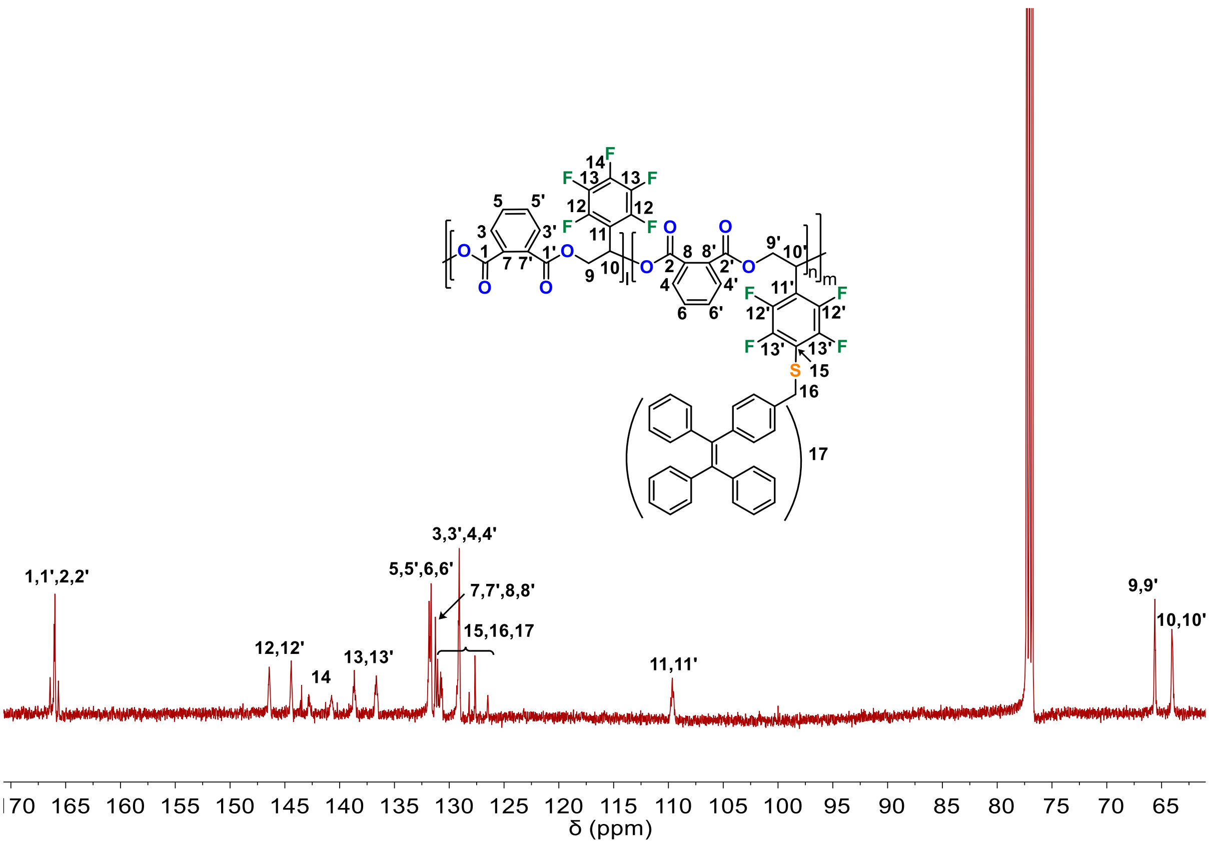

Figure S89 - ^{13}C NMR spectrum (126 MHz, $CDCl_3$) of post-functionalised ^{F}PO/PA copolymer with 4-(1,2,2-triphenylethenyl)benzenemethanethiol.

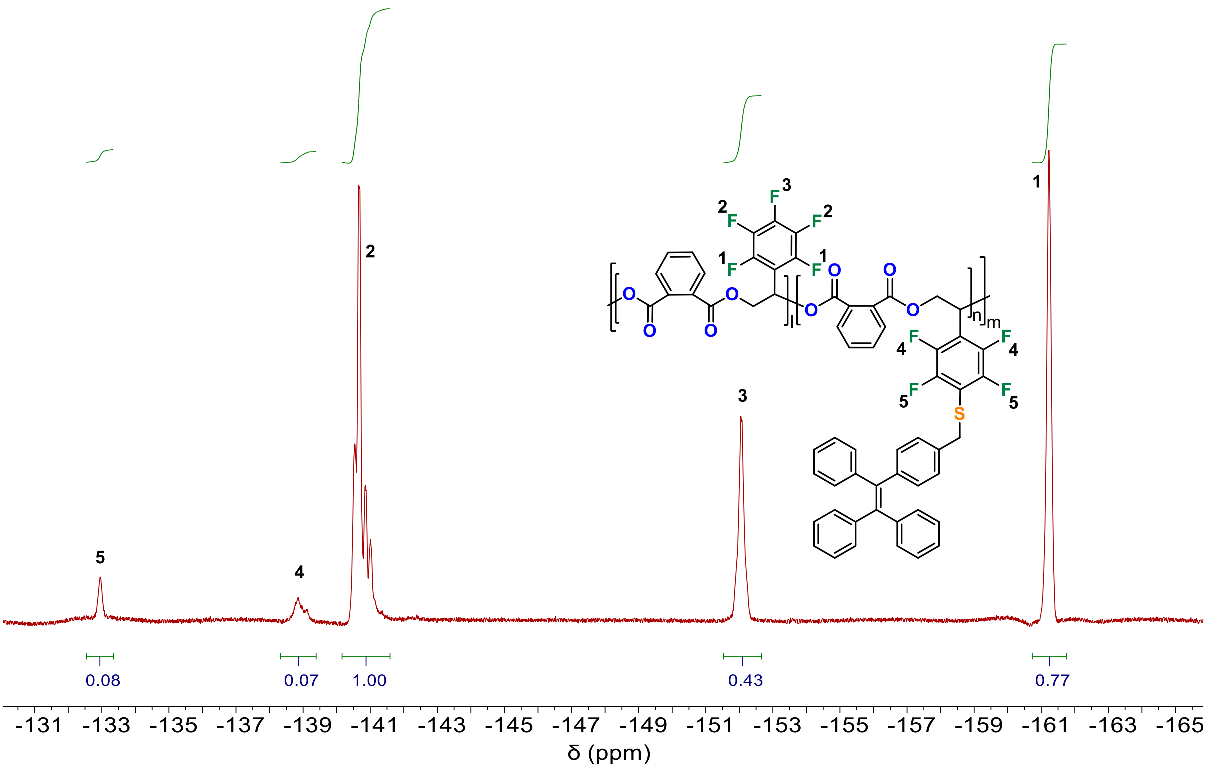

Figure S90 - ^{19}F NMR spectrum (376 MHz, $CDCl_3$) of post-functionalised ^{F}PO/PA copolymer with 4-(1,2,2-triphenylethenyl)benzenemethanethiol.

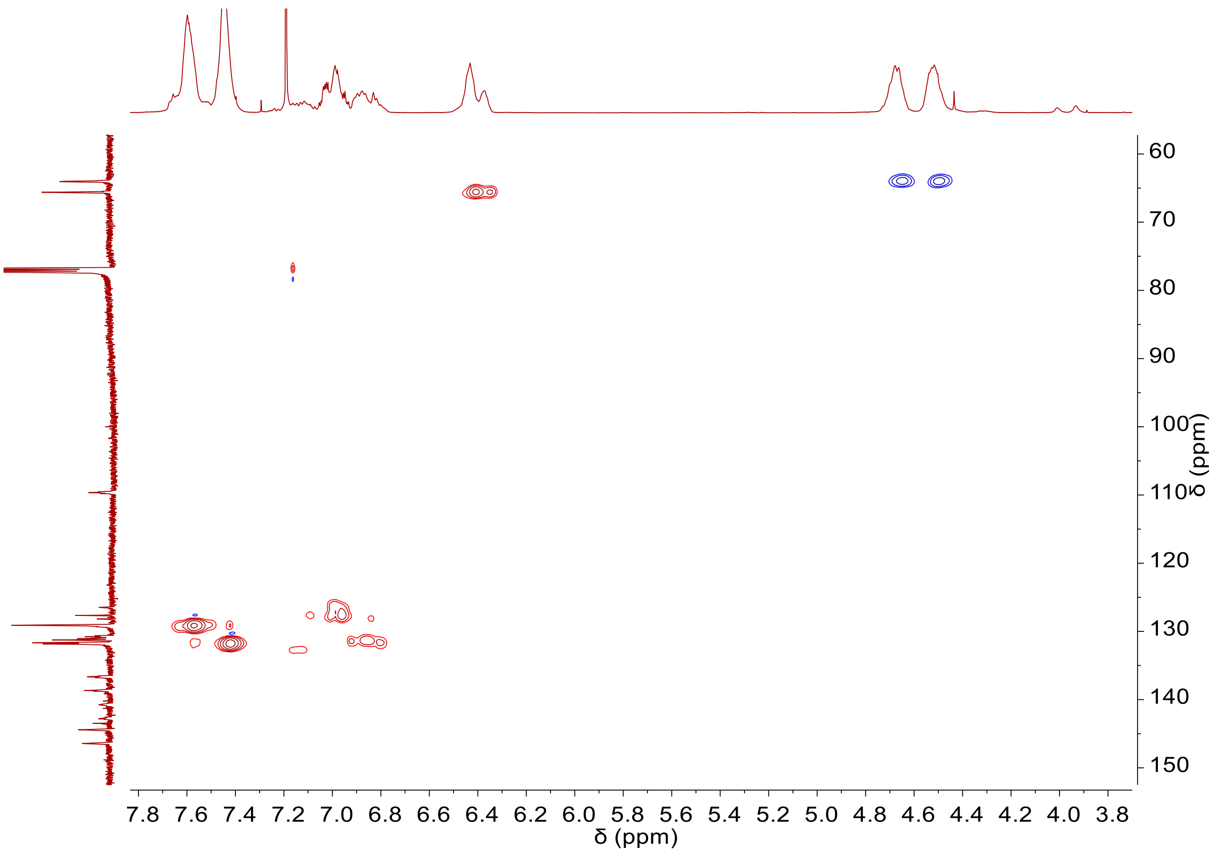

Figure S91 - ^{1}H-^{13}C HSQC NMR spectrum ($CDCl_3$) of [F]PO/PA copolymer after post-functionalisation with 4-(1,2,2-triphenylethenyl)benzenemethanethiol.

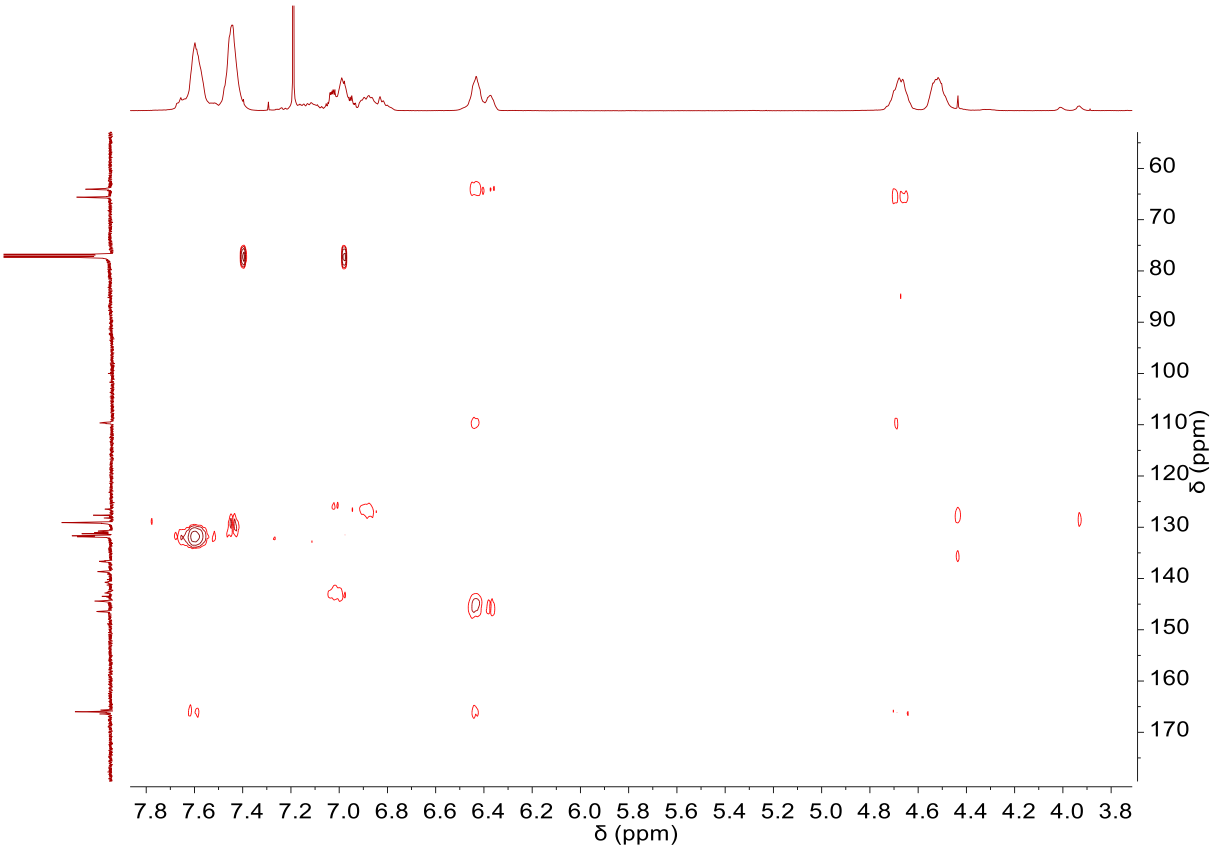

Figure S92 - ^{1}H-^{13}C HMQC NMR spectrum ($CDCl_3$) of [F]PO/PA copolymer after post-functionalisation with 4-(1,2,2-triphenylethenyl)benzenemethanethiol.

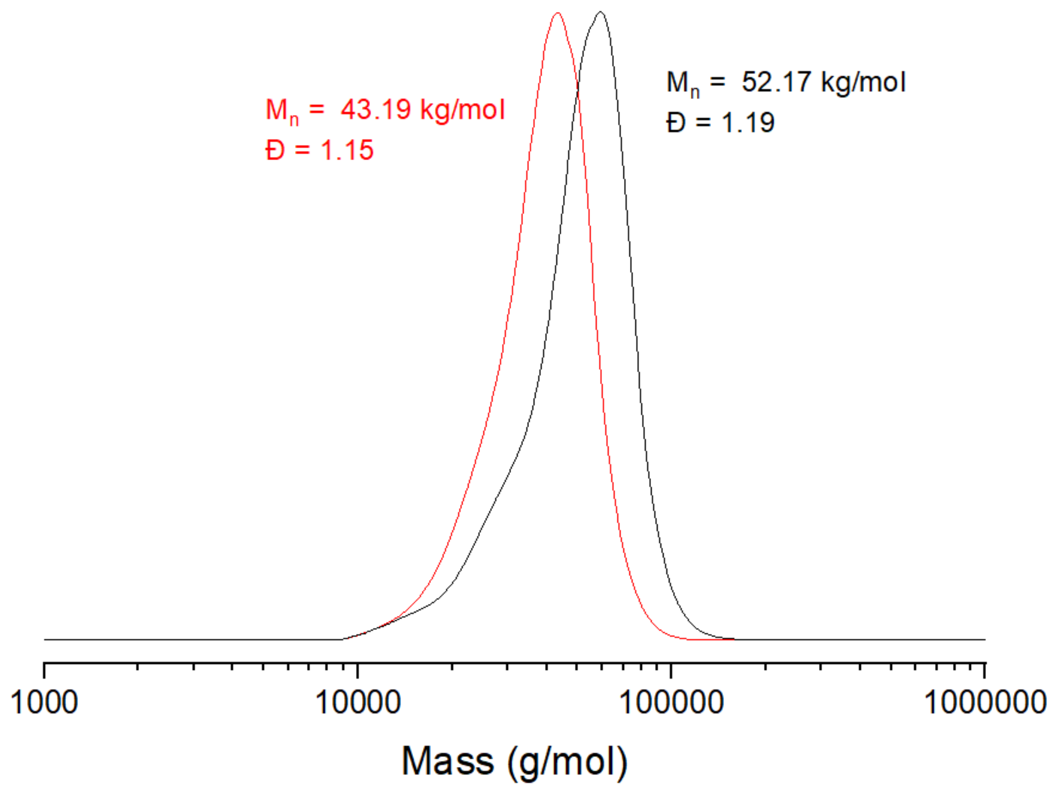

Figure S93 - GPC trace of ^{F}PO/PA copolymer after post-functionalisation with 4-(1,2,2-triphenylethenyl)benzenemethanethiol (red) vs original ^{F}PO/PA copolymer (black).

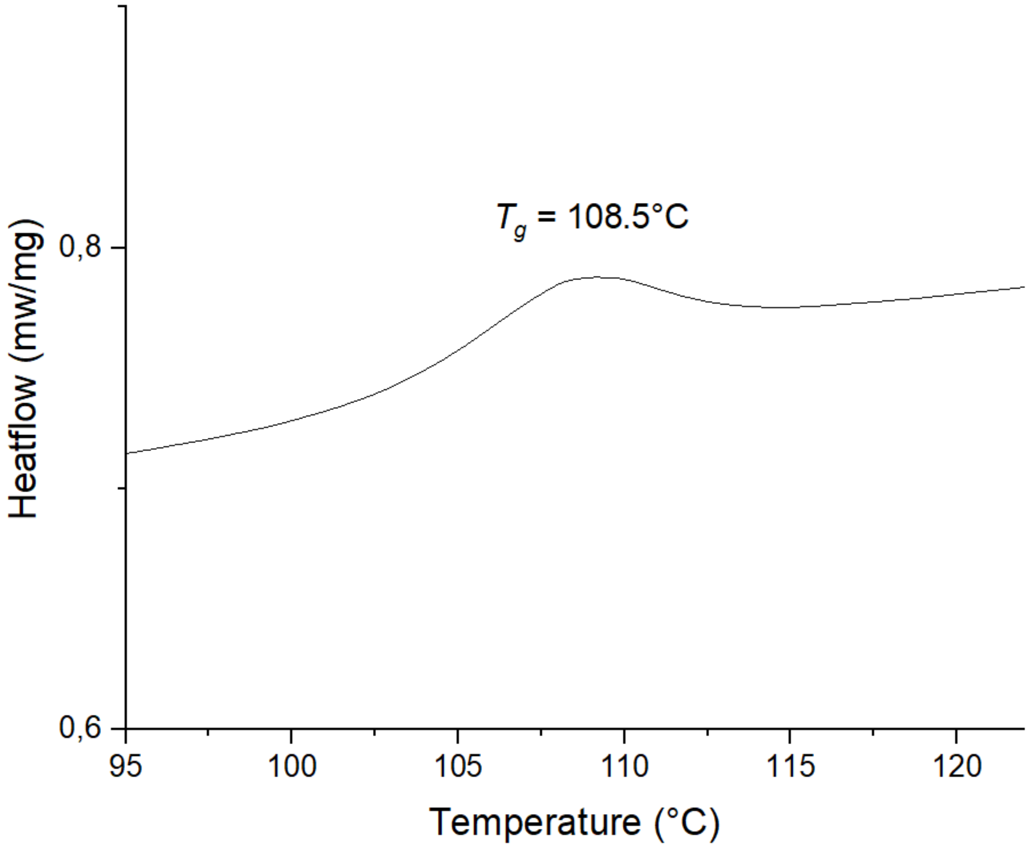

Figure S94 - DSC data from the second heating cycle corresponding to ^{F}SO/PA copolymer post-functionalised with 4-(1,2,2-triphenylethenyl)benzenemethanethiol.

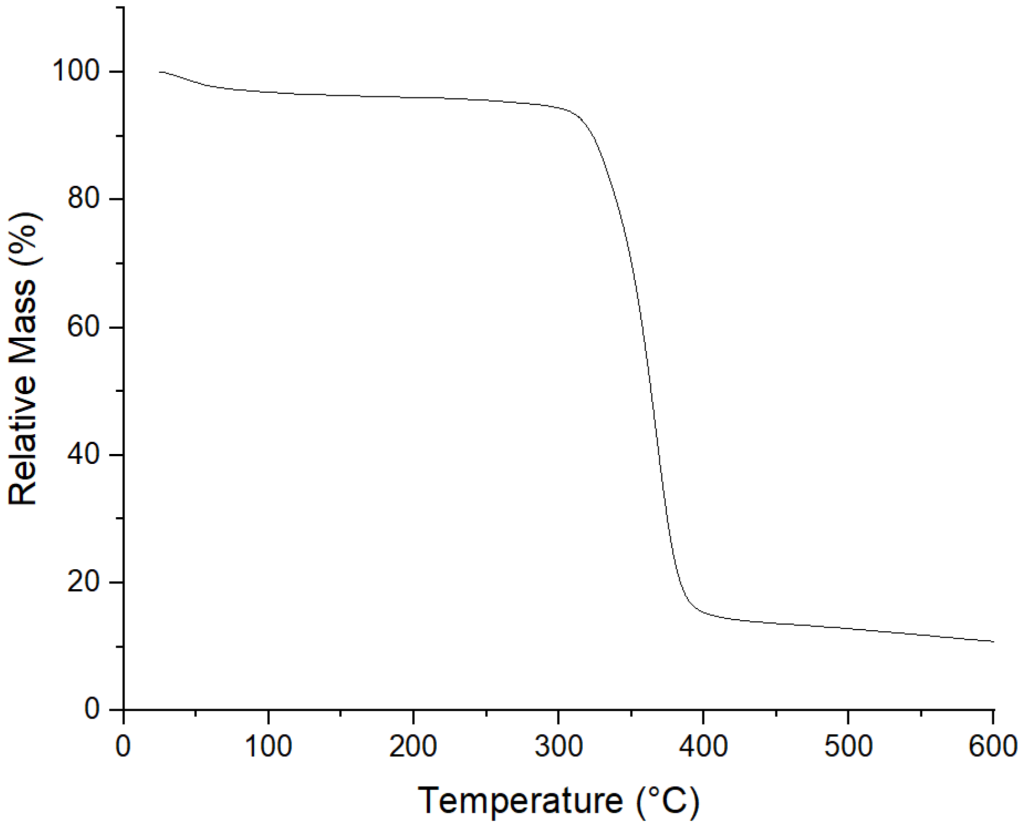

Figure S95 - TGA data of copolymer corresponding to ^{F}PO/PA copolymer post-functionalised with 4-(1,2,2-triphenylethenyl)benzenemethanethiol. $T_{d,5\%}$ = 301.5 ºC.

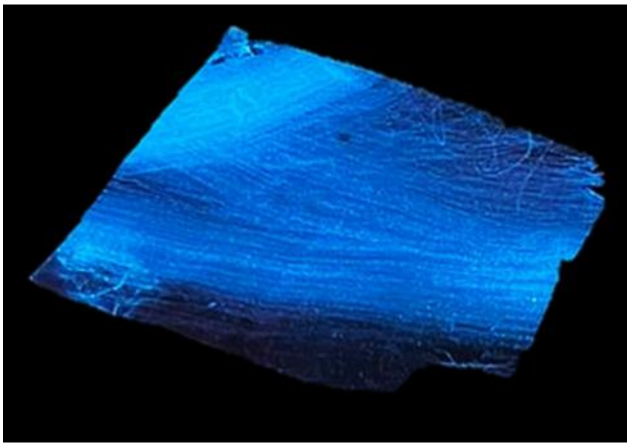

Figure S96 – Electro spun fibres after surface functionalisation with 4-(1,2,2-triphenylethenyl)benzenemethanethiol. under UV light (366 nm).

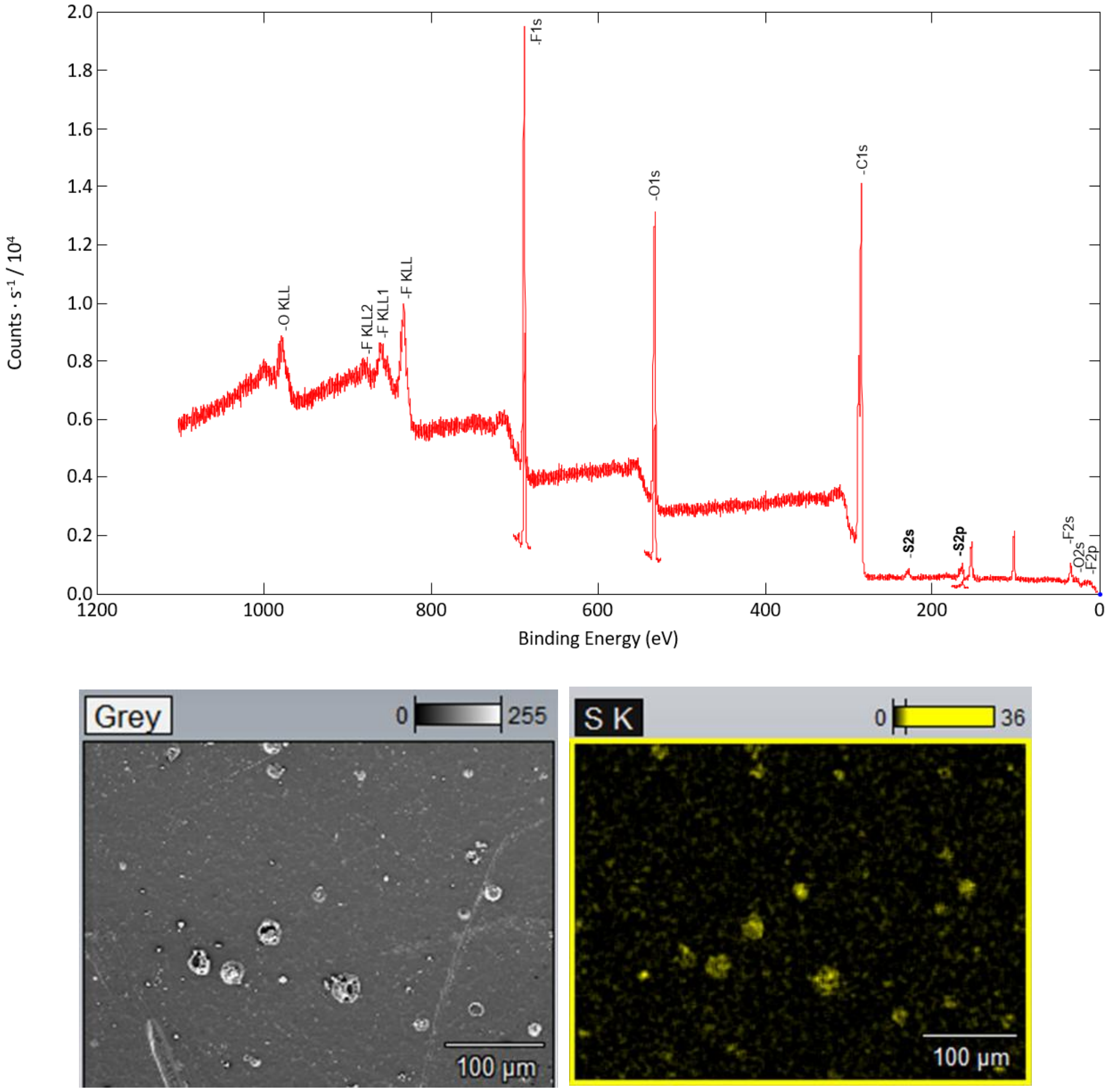

Figure S97 – XPS and SEM-EDX spectra ^{F}SO/PA copolymer surface functionalised with 4-(1,2,2-triphenylethenyl)benzenemethanethiol.

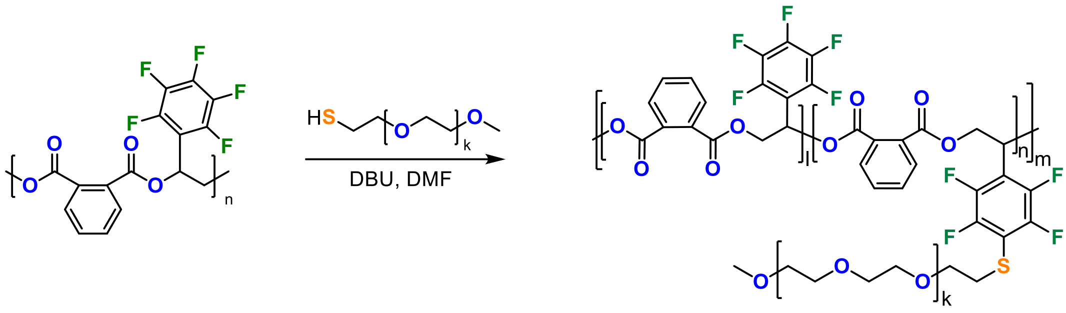

Figure S98 - Postfunctionalisation of ᶠPO/PA copolymer with mPEG-thiol.

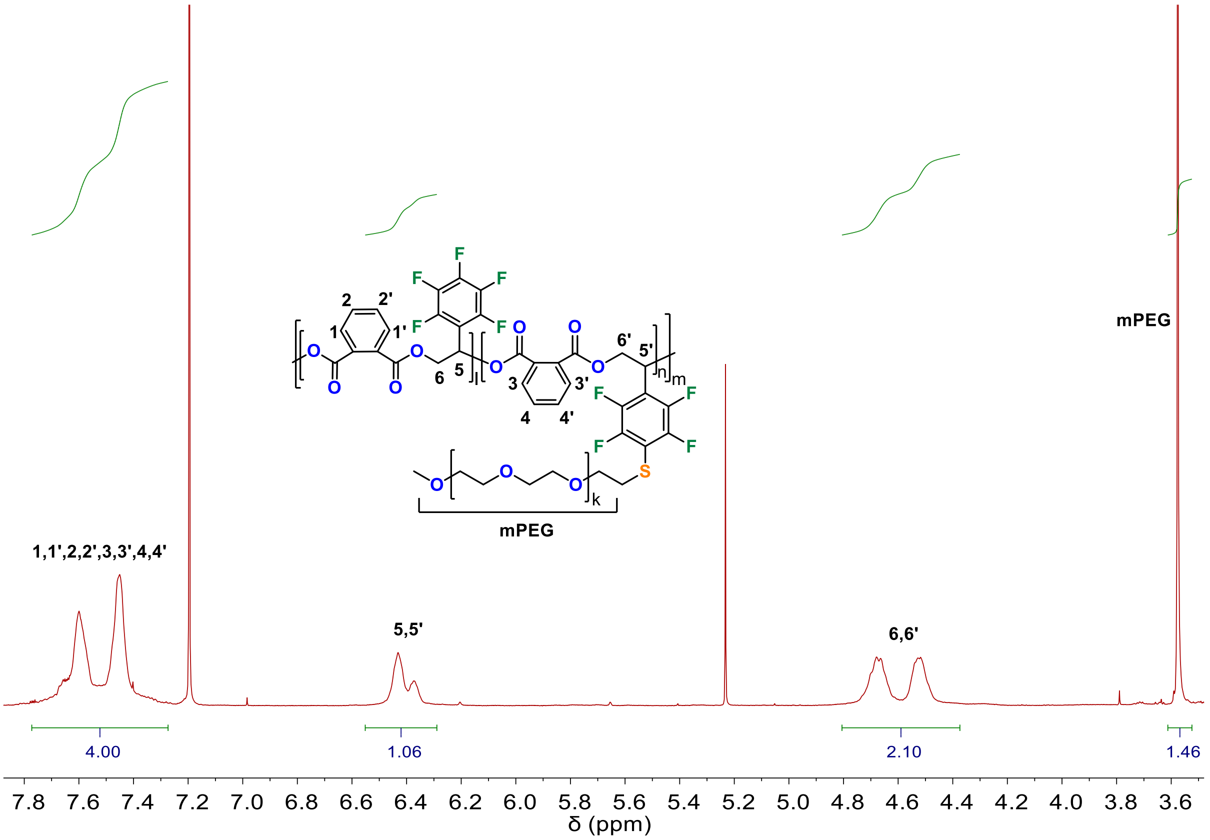

Figure S99 - ^{1}H NMR (500 MHz, $CDCl_3$) of $^{F}PO/PA$ copolymer after post-functionalisation with mPEG-thiol.

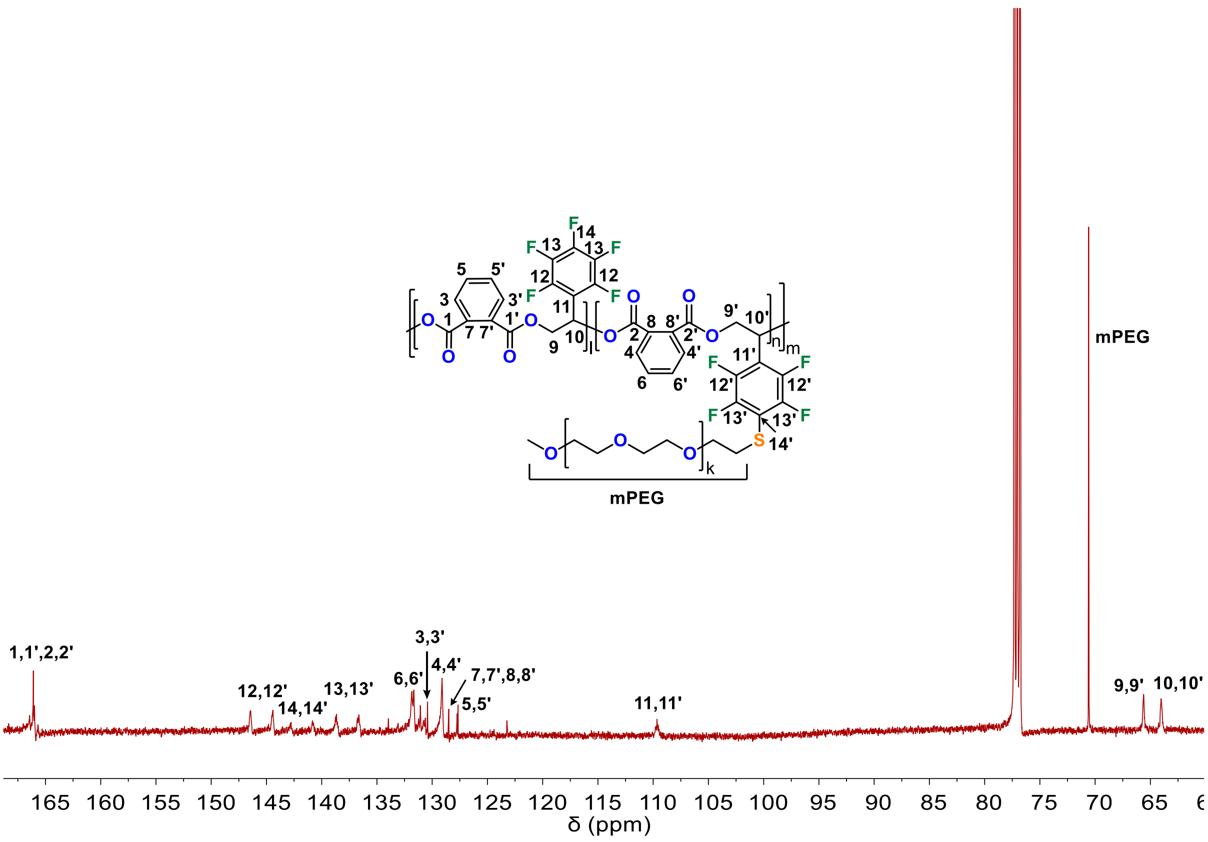

Figure S100 - ^{13}C NMR spectrum (126 MHz, $CDCl_3$) of ^{F}PO/PA copolymerisation after post-functionalisation with mPEG-thiol.

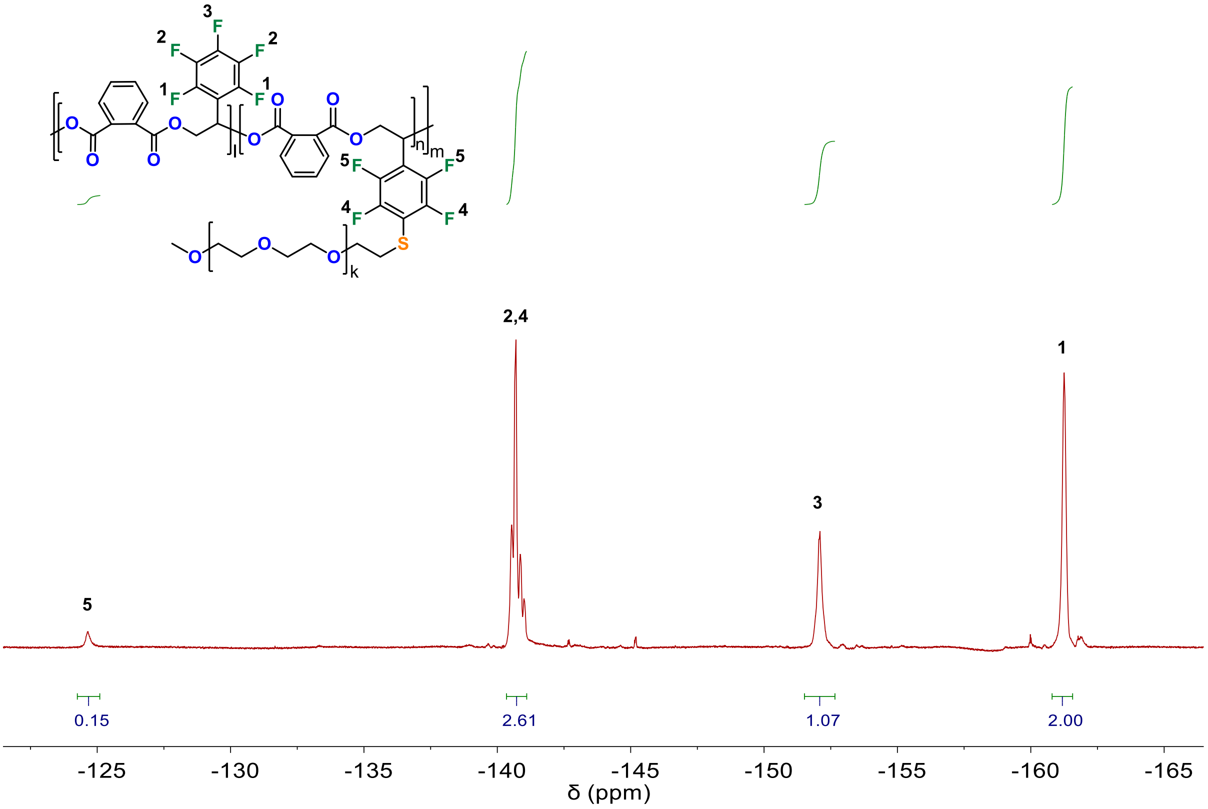

Figure S101 - ^{19}F NMR spectrum (376 MHz, $CDCl_3$) of ^{F}PO/PA copolymer after post-functionalisation with mPEG-thiol.

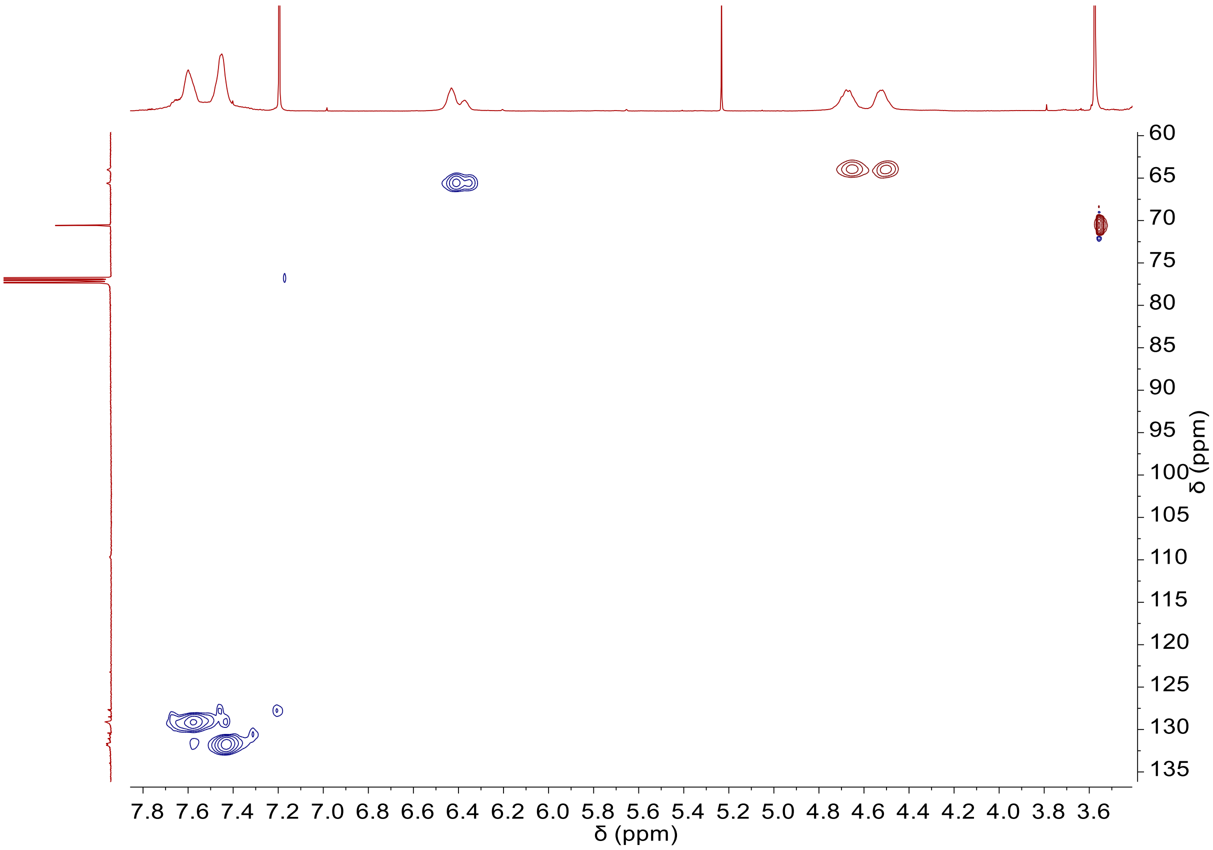

Figure S102 - ^{1}H-^{13}C HMBC NMR spectrum ($CDCl_3$) spectrum of ^{F}PO/PA copolymer after post-functionalisation with mPEG-thiol.

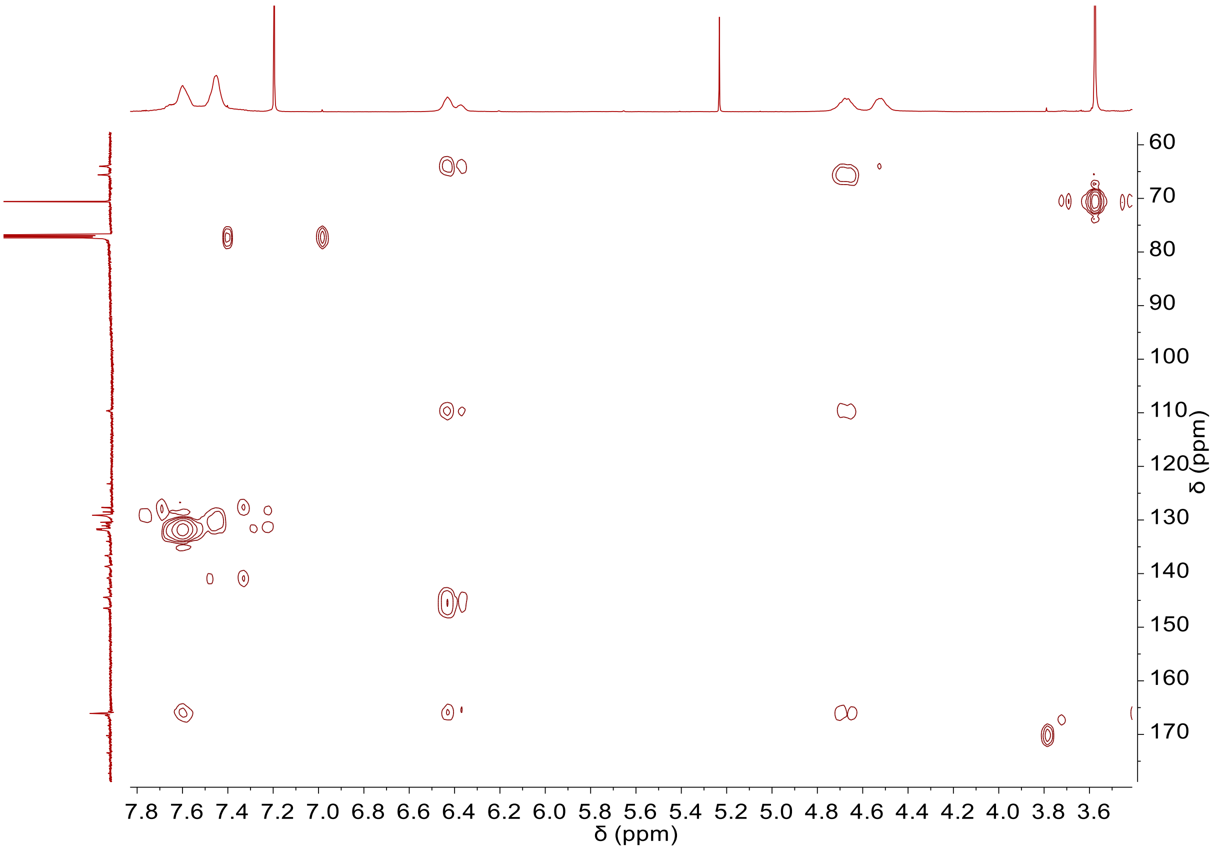

Figure S103 - ^{1}H-^{13}C HMQC NMR spectrum ($CDCl_3$) spectrum of ^{F}PO/PA copolymer after post-functionalisation with mPEG-thiol.

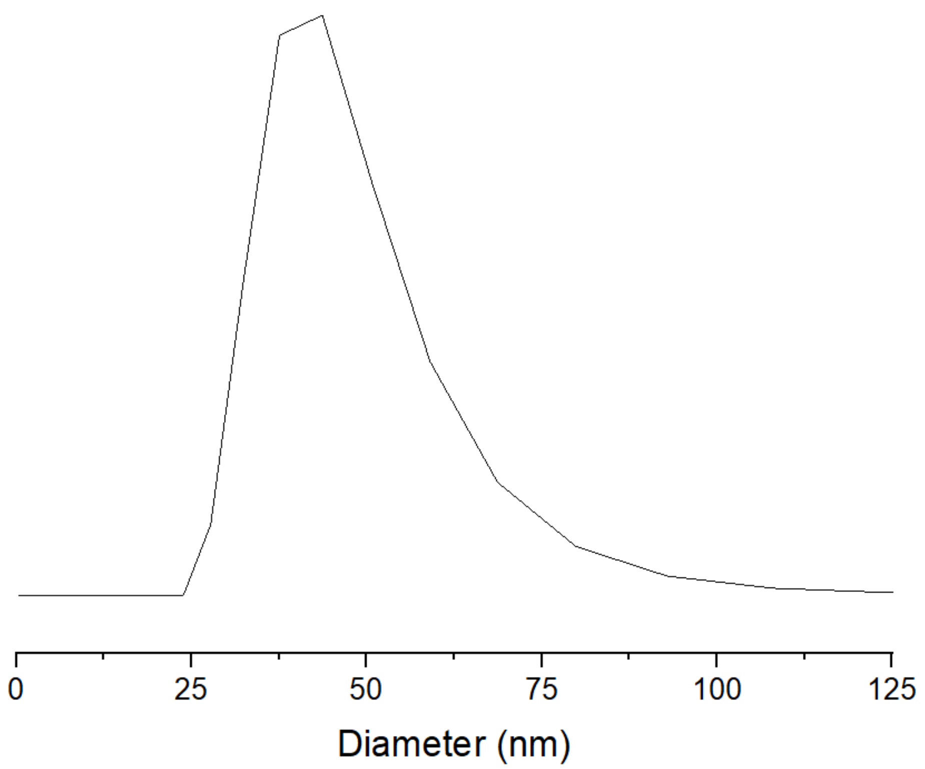

Figure S104 – DLS data from micelles formed by nanoprecipitation of the ^{F}PO/PA copolymer post-functionalised with mPEG-thiol.

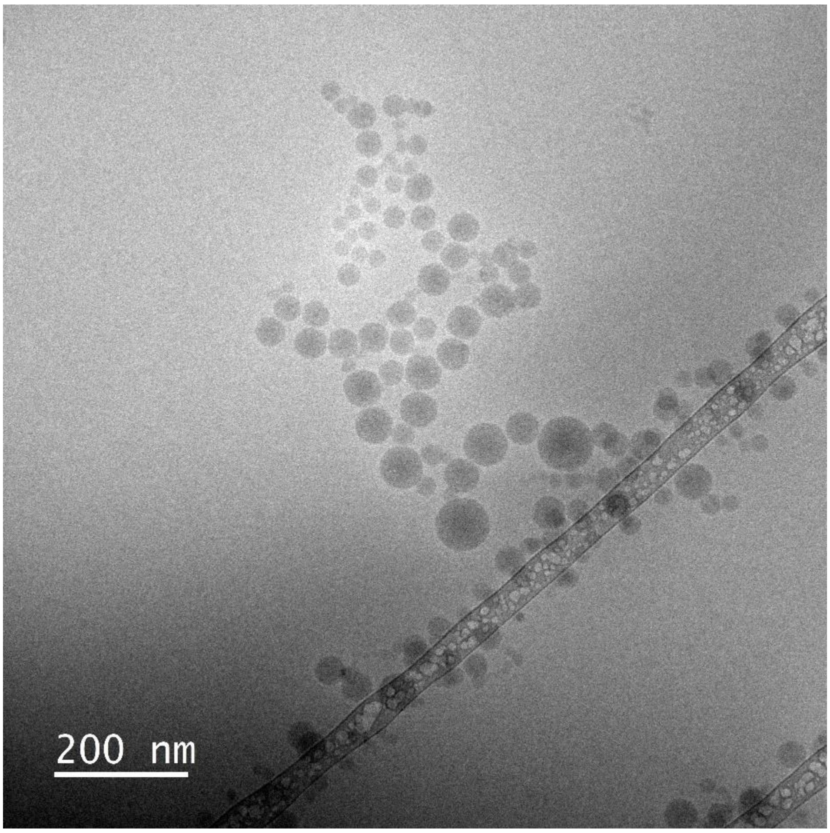

Figure S105 – Cryto-TEM image of micelles formed by nanoprecipitation of the ^{F}PO/PA copolymer post-functionalised with mPEG-thiol.

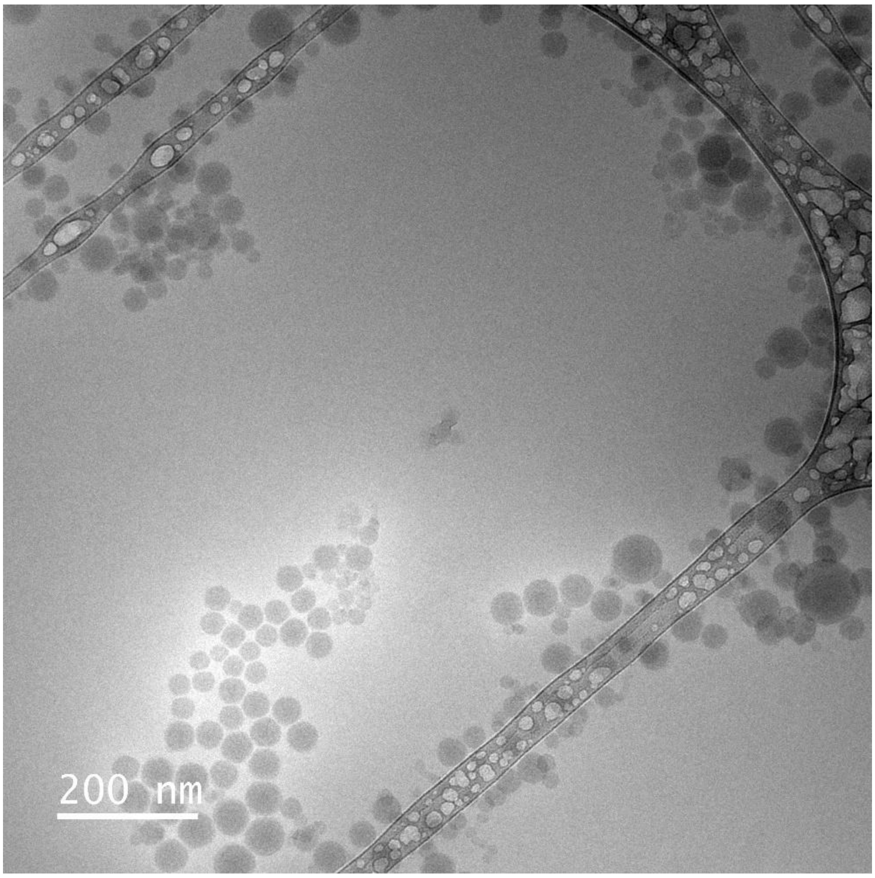

Figure S106 – Cryto-TEM image of micelles formed by nanoprecipitation of the ^{F}PO/PA copolymer post-functionalised with mPEG-thiol.

Section S10: Polymer degradation and fluoride recovery

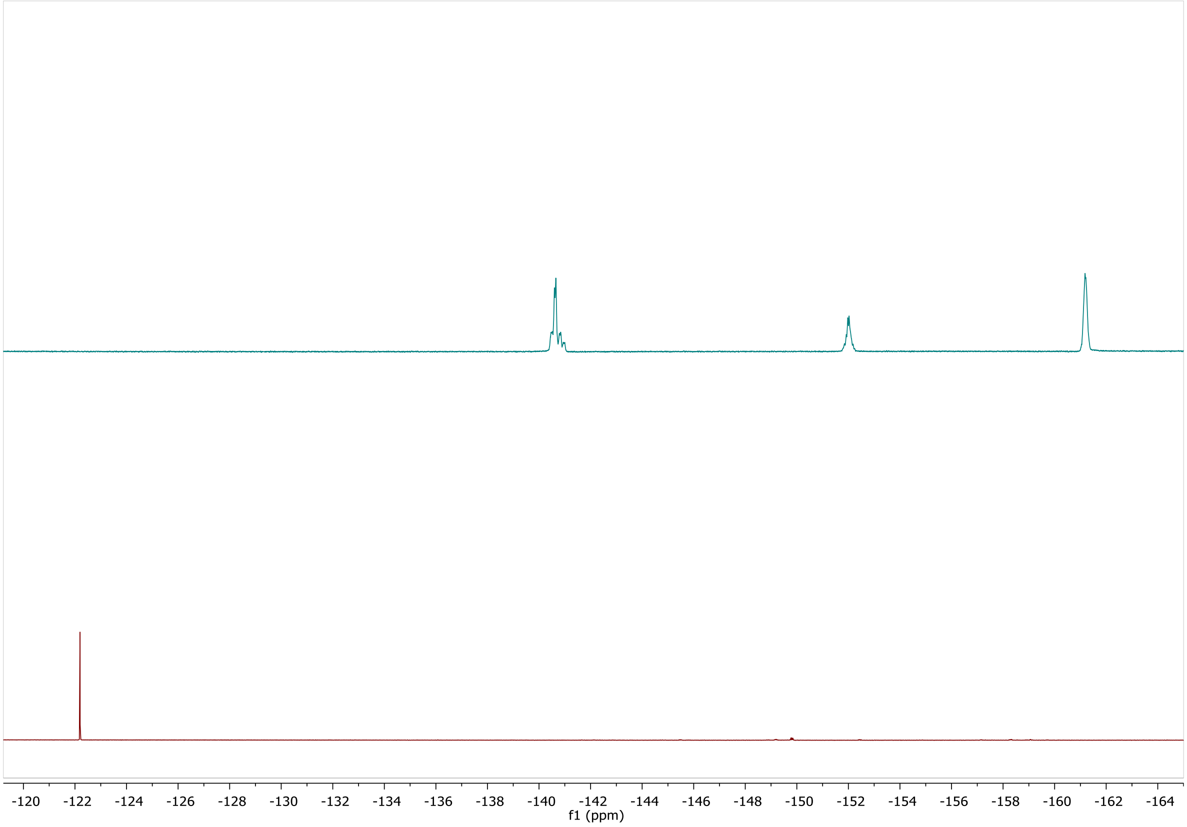

Figure S107 – ^{F}PO/PA copolymer before methanolysis (top) and after methanolysis with NaOMe (bottom).

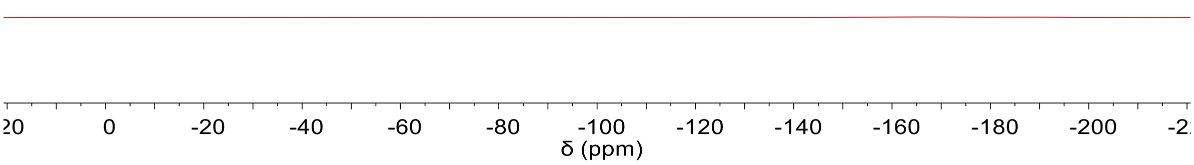

Figure S108 – ^{19}F NMR ($CDCl_3$) of methanolysis product.

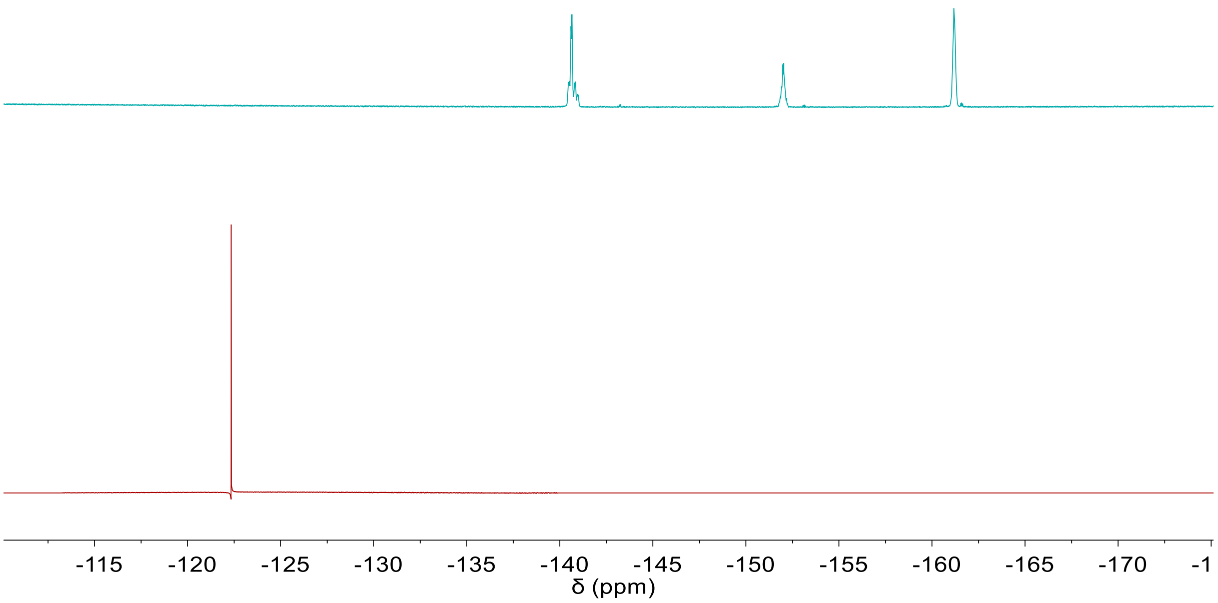

Figure S109 - ^{F}PO/PA copolymer fibres surface functionalised with perfluor-1-octanthiol before methanolysis (top) and after methanolysis with NaOMe (bottom).

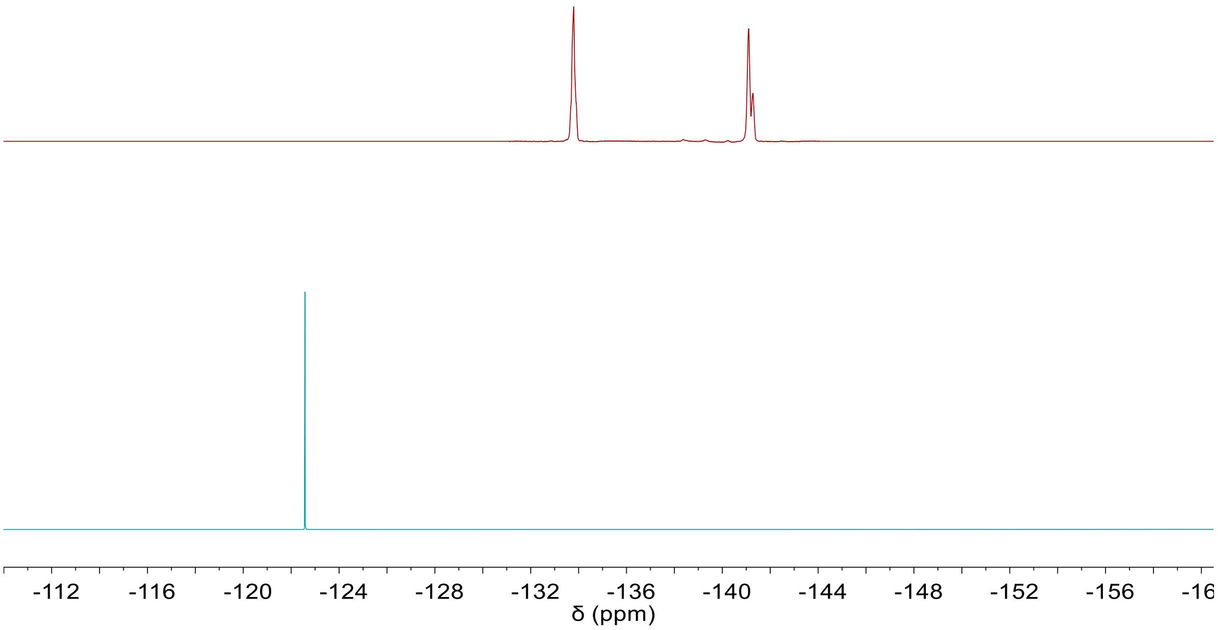

Figure S110 - ^{F}PO/PA copolymer post functionalised with hexanethiol before methanolysis (top) and after methanolysis with NaOMe (bottom).

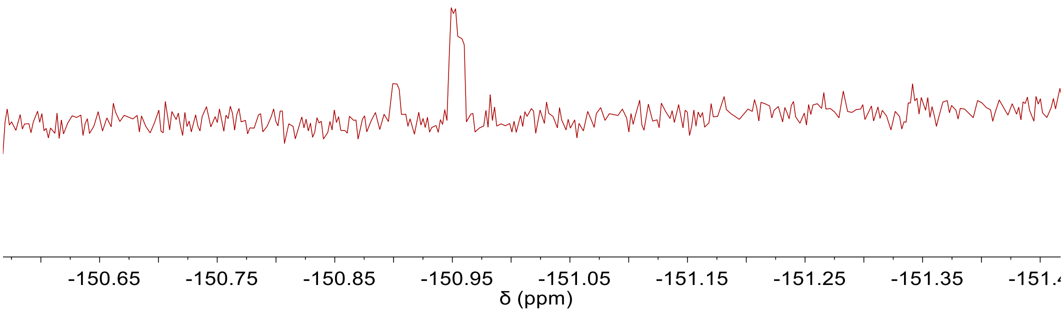

Figure S111 – ^{19}F NMR (D_2O) of methanolysis product after reaction with H_2SO_4, showing tetrafluoroborate signals.

Section S11: Computational Details

DFT Calculations of the Reaction Mechanism.

The alternating anionic copolymerization of styrene oxide and phthalic anhydride is investigated using DFT calculations. The mechanism of the copolymerization of propylene oxide and succinic anhydride using the same catalytic system was studied by Fieser et al.[8] That study provides a detailed examination of the initiation step and explores multiple reaction cycles, while the most probable cycle was further used to develop the model presented here. Furthermore, the substituent effects of using fluorinated styrene oxide are discussed which causes a rate accelerating effect observed by experiments.

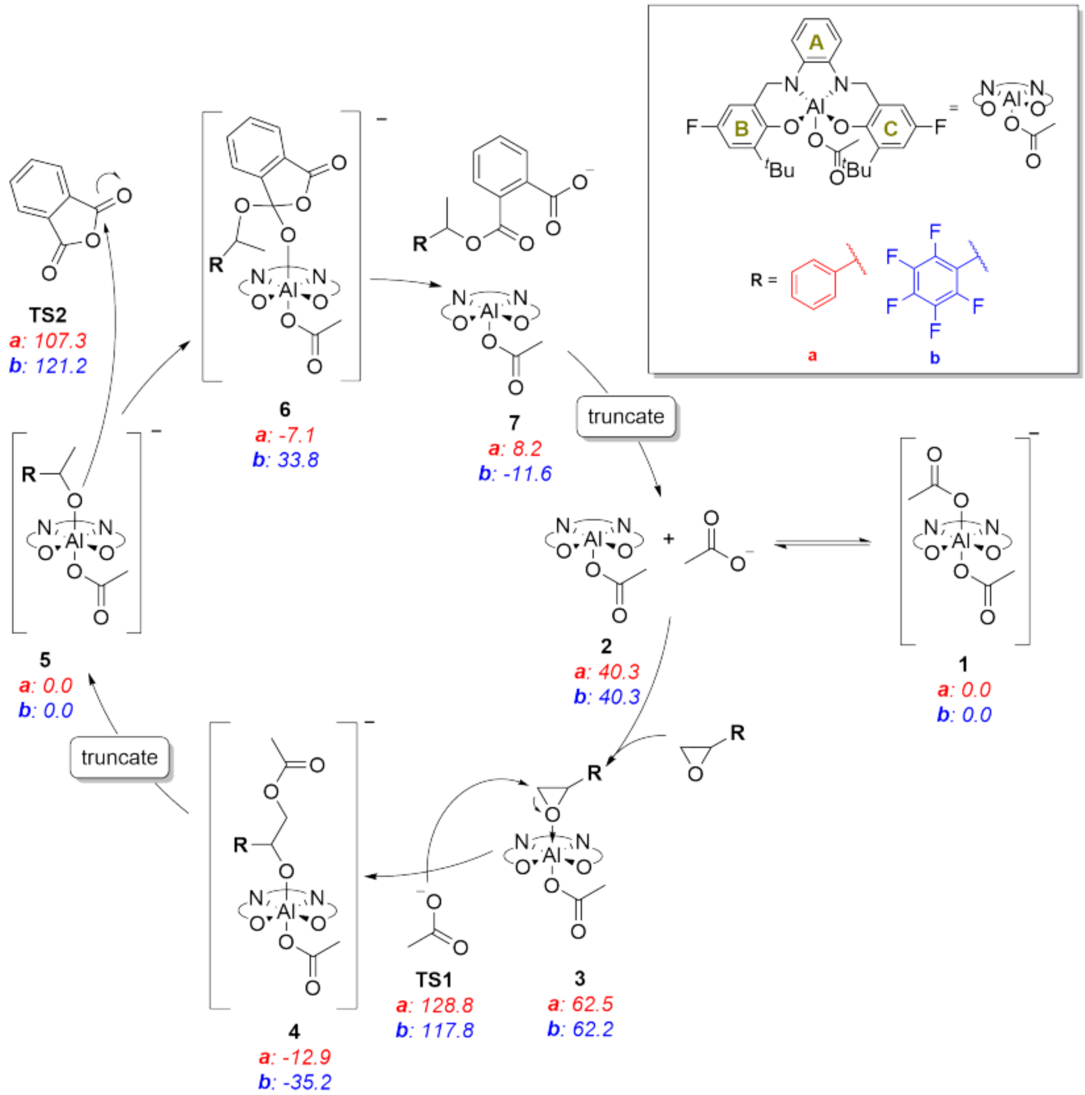

Figure S112 - Simplified reaction mechanism of the copolymerization of (fluorinated) styrene oxide and phthalic anhydride, with relative Gibbs free energies (kJ/mol) computed at 353 K using the wB97M-V/def2-TZVPP//PBEh-3c//COSMO-RS/TZVPD(styrene oxide) level of theory.

We begin by discussing the unfluorinated mechanism, referred to as path **a**. The reaction mechanism in Figure S104 is divided into two steps: a nucleophilic attack on styrene oxide which defines intermediates **1–4** and an electrophilic attack performed by phthalic anhydride, defining intermediates **5–7**. Starting with intermediate **1**, two acetate ligands bind to the aluminium catalyst. Both ligands bound to the catalyst represent the ends of a growing polymer

chain. Starting from this doubly bound state, the catalytic reaction proceeds with the addition of styrene oxide, initiated by elimination of one of the **1** acetate ligands. Ejection of the acetate anion leads to intermediate **2a** and causes an energy increase of 40.3 kJ/mol. The neutral styrene oxide binds to the catalyst's empty site in an endergonic addition reaction, forming intermediate **3a**. Upon binding, the unsubstituted carbon atom of the epoxy group becomes activated for a nucleophilic attack. The nucleophilic attack by the acetate molecule involves a total energy barrier of 128.8 kJ/mol making the formation of the transition state **TS1a** the rate determining step of the reaction. During this bond formation, the epoxy ring opens, leading to the exergonic formation of intermediate **4**. Phthalic anhydride reacts with intermediate **5**. Intermediate **5** is a truncated version of intermediate **4** in which the acetate is replaced by hydrogen. The energy of intermediate **5** is set to 0, for easier comparison of substituent effects later. Phthalic anhydride serves as the electrophile, attacking intermediate **5a** at the coordinated alkoxide oxygen. With an energy of 107.3 kJ/mol, the resulting barrier is lower than the barrier between intermediate **1** and **TS1a**. Because the newly formed ether group is weakly coordinating, a ligand rearrangement follows in which the negatively charged oxygen of phthalic anhydride stabilizes the charge by coordinating to the catalyst. Releasing the polymer chain forms intermediate **7**, which is truncated to restart the catalytic cycle.

<u>The π-Stacking in Fluorinated Styrene Oxide Stabilizes the Charge.</u>

Fluorinating styrene oxide slightly accelerates the reaction, as observed experimentally. We discuss the rate accelerating effect of fluorinating styrene oxide in this section. The fluorinated transition state **TS1b** is stabilized by 11.1 kJ/mol relative to **TS1a**, which aligns well with the experimentally observed rate acceleration. Because arenes tend to stack with fluorinated arenes, π-stacking was our initial hypothesis to explain the kinetic effects.[9]

Table S4 - The geometric data for molecules obtained through conformational sampling by the CREST/CENSO approach at the PBEh-3c/CPCM(ε = 2.25, styrene oxide) level of structure optimization. The centroid distance D_1 is measured between the aromatic centre of styrene oxide and the aromatic centre of the closest ring in the catalyst. The corresponding aromatic sites A, B or C of the catalyst are defined in Figure S104. The dihedral angle A1 is defined between the planes of the two involved aromatic rings. D_{rxn} indicates the distance along the acetate oxygen and the epoxy carbon, while D_{rxn2} is the bond length in the epoxy ring, which opens upon acetate attack. D_{Al-O} describes the bond length between the aluminium atom and the epoxy oxygen. Distances are given in Angstrom (Å) and angles in degrees (°).

Int.	D_1 (Å)	A_1 (°)	Site	D_{rxn}	D_{rxn2}	D_{Al-O}
**3a**	3.90	6.5	**B**	-	1.42	2.32
**3b**	3.67	18.4	**B**	-	1.42	2.39
**TS1a**	3.79	8.9	**B**	2.14	1.71	1.96
**TS1b**	3.58	6.0	**A**	2.17	1.69	1.97
**4a**	3.96	9.8	**B**	1.43	2.37	1.83
**4b**	3.50	4.4	**A**	1.43	2.36	1.84

Geometric parameters of the π-stacking interactions, including the centroid distances between the aromatic systems and the corresponding angles, are listed in *Table S4*. All structures exhibit typical πstacking distances of aromatic rings.[10] The low value of angle A1 indicates an almost parallel alignment of the aromatic systems. Whereas the unfluorinated ring of styrene oxide tends to stack with the mono-fluorinated aromatic rings of the catalyst (sites **B** and **C**),

the fluorinated ring of styrene oxide preferentially stacks with the unfluorinated site **A**. (The labelling of sites **A–C** is provided in *Figure 1*.) Notably, the centroid distance of the π-stacking systems decreases when going from styrene oxide to fluorinated styrene oxide.

The anionic π-component The neutral intermediates **3a** and **3b** are energetically similar, even though the coordination of the epoxy group was expected to weaken due to the fluorinated arene substituent. However, the slight weakening of the bond may be compensated by the π-stacking which exhibit moderate distances of 3.67 Å in the fluorinated case.

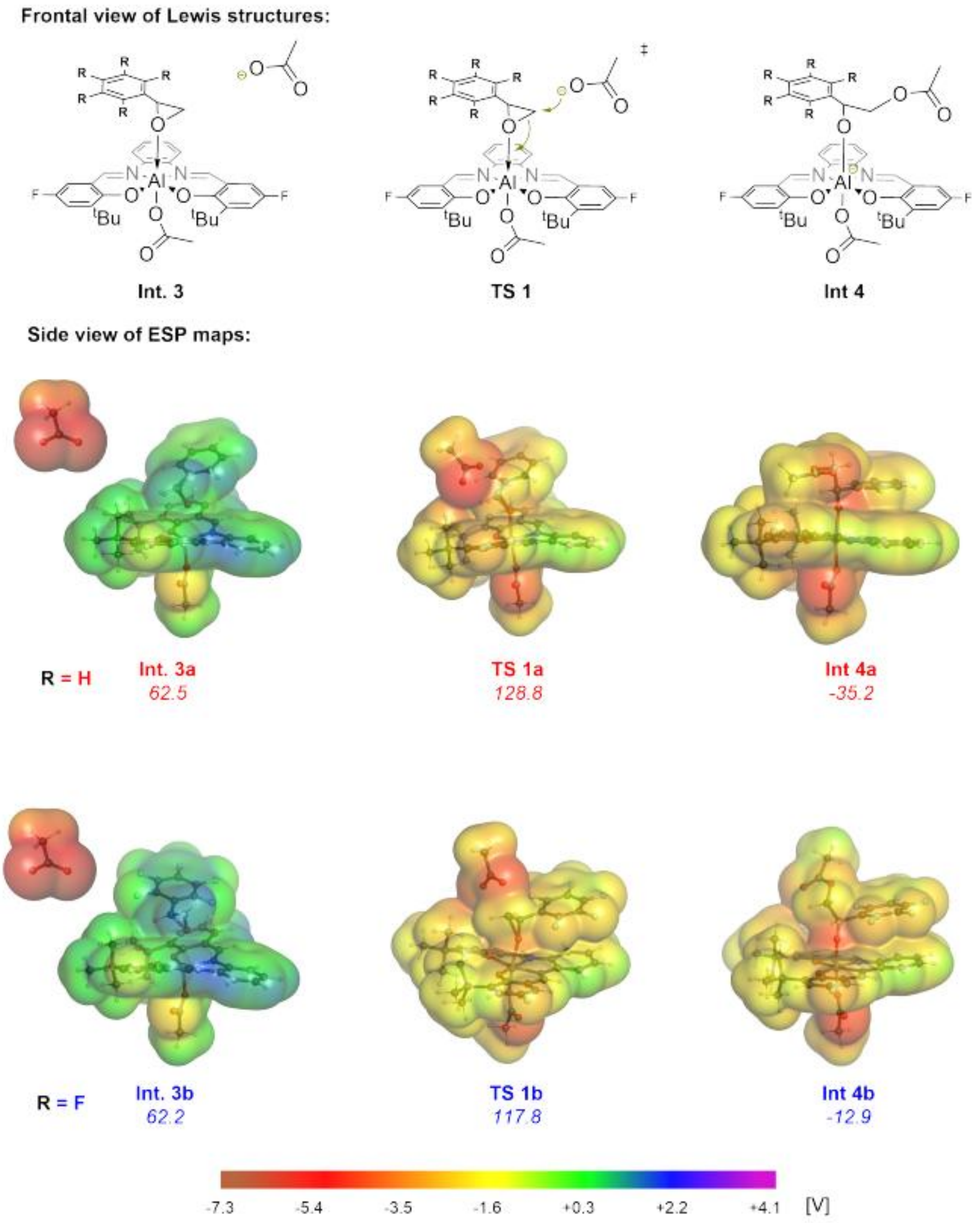

Figure S113 - The mechanism of the acetate attack on intermediate **3** using Lewis structures and the electrostatic potential mapped onto an iso-surface of the electron density at 0.0135 e/Å^3 (0.002 e/a$^3_0$). The iso-surface density and the electrostatic potential were calculated in gas-phase using wB97X-D4/def2- QZVP//PBEh-3c level of DFT.

The similar energies of intermediates **3a** and **3b** is lifted once the acetate anion attacks. Charge redistribution to styrene oxide and the aluminium atom ultimately results in full delocalization within the complex. This charge delocalization is further supported by Figure S105, which shows the electrostatic potential maps of both pathways. Upon charge redistribution, **TS1b**

and **TS4b** exhibit a stronger electrostatic interaction between the negatively charged fluorine atoms (orange) and slightly positively charged hydrogen atoms (green). As the reaction progresses, charge redistribution leads to an anionic-π component in the π-stacking interaction, which helps explain the changes in bond lengths and the energetic contributions driving the rate acceleration. When the reaction proceeds to intermediate **4**, the charge gets fully distributed to the catalyst. Hence, the anionic-π interaction gets stronger such that the fluorinated intermediate **4b** is 22.4 kJ/mol more stable than intermediate **4a**.

Transition state 2

As intermediate **4**, also the simplified intermediate **5** is stabilized by the anionic π-interaction. When phthalic anhydride performs an electrophilic attack on intermediate **5**, the anionic π-component decreases and the resulting energy difference of 13.9 kJ/mol favours **TS2a** over **TS2b**. The unfluorinated path a being favoured supports the significance of the anionic-π component. The stabilizing anionic–π component between fluorinated styrene oxide and the catalyst's π-system vanishes when the charge is redistributed to phthalic anhydride during the electrophilic attack. The loss of the charge stabilization increases the energy of **TS2b**. Furthermore, in contrast to path **a**, the fluorinated path **b** exhibits two barriers of similar magnitude (**TS1b**: 117.8 vs. **TS2b**: 121.2 kJ/mol).

Computational Details

Conformational Space Exploration The preliminary optimization was performed via conformational space exploration using the CREST/CENSO approach.[11,12] CREST 2.11 and xTB 2.11 with GFN2 parametrization were employed, along with the ALPB solvation model in THF.[13-15] The ensemble was refined via CENSO 1.2 interfacing TURBOMOLE 7.6.1.[16] Geometric structure optimization was carried out with the PBEh-3c composite level of theory, using COSMO (ε = 2.25, styrene oxide).[17,18] During the refinement in the CENSO program, the best conformer was selected based on Gibbs free energies of the ensemble, calculated from PBE0-D4/def2-TZVP single point electronic energies, thermal corrections at the xTB level, and COSMO-RS/BP_TZVP_COSMO_19 solvation enthalpies.[19-27]

Transition state optimization

Guess structures were generated by scanning the reaction coordinate and applying the nudge elastic band (NEB) method with a QM/xTB ONIOM approach.[28] These guesses originated from intermediates obtained during the conformer sampling. Transition state optimization yielded a confirmed imaginary mode that corresponds to the reaction coordinate.

Final Gibbs free energy values

The obtained structures were further processed by geometry optimizations and normal mode analyses at the PBEh-3c/CPCM (ε = 2.25, styrene oxide) level using ORCA 5.0.3.[18,29–34] These optimizations employed tight criteria for both geometric and electronic convergence, confirming that the final structures were true minima or valid transition states. Spurious imaginary modes lower than 30 cm^{-1} were treated as positive during the calculation of thermal corrections. Thermal corrections were calculated at 353 K and 105 Pa by applying the quasi rigid rotator harmonic oscillator (quasi-RRHO) approach with the frequencies above 100 cm^{-1} cut-off.[28] For single-point calculations, the ωB97M-V/def2-TZVPP functional was chosen, because it includes the VV109 non-local correlation term, which provides highly accurate descriptions of non-covalent interactions such as π-stacking.[29-30] Moreover, this functional is recommended for computing energy barriers in organic systems.[31] COSMO-RS solvation enthalpies were evaluated using the BP_TZVPD_FINE_19 parametrization with the COSMOtherm program, using one gas-phase single point calculation at the BP86/def2-TZVPD level, with a reference single point calculation done under ideal conductor approximation (COSMO, ε = ∞).[24-27] A

custom styrene oxide solvent entry was created for this purpose. We refer to the described methodology as the ωB97M-V/def2-TZVPP//PBEh-3c//COSMO-RS/TZVPD level of theory. Electrostatic potential maps were computed in gas-phase using wB97X-D4/def2-QZVP.[21,22,37]

The authors would like to thank the HPC Service of FUB-IT, Freie Universität Berlin, for computing time.[38]

17

E_el=-1010405.33, G_thr=273.36, G_solv=-26.35, PBEh-3c/CPCM, Styrene Oxide

O	2.47766	0.44786	-0.53406
C	2.54624	-0.04947	0.78579
C	1.58740	-0.58768	-0.18342
C	0.14129	-0.25869	-0.10607
C	-0.30274	1.05970	-0.06500
C	-1.65743	1.33721	0.03650
C	-2.58355	0.30456	0.09343
C	-2.14668	-1.01126	0.04218
C	-0.79178	-1.28945	-0.06099
H	0.40971	1.87236	-0.12535
H	-1.99210	2.36603	0.06600
H	-3.64017	0.52469	0.17051
H	-2.86146	-1.82304	0.07701
H	-0.45789	-2.31956	-0.10798
H	2.18776	0.61633	1.56712
H	3.43860	-0.61724	1.03731
H	1.81308	-1.55631	-0.62903

70

E_el=-5289657.70, G_thr=1298.81, G_solv=-92.59, PBEh-3c/CPCM, Int. 2

C	4.73801	-0.84444	-0.48214
C	3.33625	-0.73767	-0.39368
C	2.69631	0.51824	-0.25532
C	3.49888	1.69827	-0.22465
C	4.86935	1.54293	-0.29504
C	5.47524	0.29394	-0.41741
C	2.59088	-1.94379	-0.50420
N	1.31212	-2.05289	-0.36710
Al	0.06949	-0.55326	0.19719
O	0.18119	-0.89750	1.94916
C	-0.85166	-1.10008	2.72674
C	-0.49347	-1.19658	4.18936
O	1.40232	0.59509	-0.18845
C	2.87520	3.09035	-0.13770
C	2.03724	3.34972	-1.39840
F	6.81577	0.23872	-0.48182
C	0.65650	-3.28021	-0.50906
C	1.25425	-4.53623	-0.49530
C	0.47750	-5.67205	-0.63559
C	-0.90146	-5.56274	-0.76690
C	-1.50736	-4.31921	-0.75816
C	-0.73257	-3.16877	-0.64421
N	-1.21618	-1.85573	-0.59377
C	-2.43178	-1.57559	-0.94416
C	-3.08249	-0.32602	-0.79953
C	-2.41927	0.79998	-0.25127
C	-3.17166	1.98762	-0.00031
C	-4.50038	1.99084	-0.37195
C	-5.11806	0.88654	-0.96014
C	-4.44174	-0.27010	-1.17144
O	-1.15068	0.75405	-0.00517

C	-2.54215	3.21094	0.66461
C	-1.45224	3.79775	-0.24088
F	-6.41467	0.98801	-1.29801
C	-1.95968	2.83361	2.03554
C	-3.57493	4.31594	0.90748
C	3.94286	4.18737	-0.07465
C	2.02081	3.22818	1.13102
O	-1.99010	-1.19794	2.32216
H	3.17441	-2.83105	-0.74372
H	-3.04170	-2.36694	-1.37503
H	-4.93199	-1.13574	-1.59953
H	-5.11233	2.86678	-0.20841
H	5.52028	2.40513	-0.26082
H	5.21104	-1.81259	-0.59174
H	-2.58607	-4.26309	-0.81018
H	-1.51087	-6.45211	-0.85072
H	0.94638	-6.64632	-0.61917
H	2.32113	-4.64479	-0.35322
H	-4.00992	4.69337	-0.02005
H	-3.08570	5.15960	1.39590
H	-4.38654	3.99069	1.56134
H	-0.64917	3.09219	-0.42820
H	-1.01290	4.68108	0.22828
H	-1.86931	4.10551	-1.20177
H	-2.72859	2.40452	2.68007
H	-1.57310	3.72836	2.52814
H	-1.14441	2.11965	1.96439
H	2.62219	3.04268	2.02297
H	1.17272	2.55086	1.14584
H	1.62947	4.24525	1.20199
H	1.23853	2.62390	-1.52527
H	2.66673	3.32171	-2.28989
H	1.57987	4.34003	-1.34625

H	4.58259	4.09516	0.80536
H	3.45117	5.15908	-0.01442
H	4.57826	4.20581	-0.96217
H	-1.36569	-1.43354	4.79254
H	-0.07328	-0.24961	4.53216
H	0.27209	-1.95660	4.34804

7

E_el=-600030.43, G_thr=45.21, G_solv=-221.55, PBEh-3c/CPCM, Acetate

C	1.39164	0.00135	0.00727
H	1.75059	0.02817	-1.02671
C	-0.16083	-0.00086	0.01287
H	1.80657	-0.89773	0.46578
H	1.80215	0.87975	0.50820
O	-0.70206	-1.12215	-0.00366
O	-0.70335	1.12004	-0.00305

87

E_el=-6300132.49, G_thr=1647.97, G_solv=-103.03, PBEh-3c/CPCM, Int. 3a

C	4.53540	1.87373	0.87189
C	3.29400	1.39029	0.41589
C	2.96647	0.01207	0.49509
C	3.92516	-0.88730	1.05467
C	5.13291	-0.36664	1.47610
C	5.43027	0.99048	1.38510
C	2.41416	2.35939	-0.14378
N	1.25080	2.13321	-0.65123
C	0.48063	3.14412	-1.23429
C	-0.83962	2.77570	-1.53313
C	-1.68978	3.68591	-2.15357
C	-1.24471	4.96223	-2.44813
C	0.06114	5.33008	-2.14887
C	0.92330	4.42368	-1.55768
N	-1.17258	1.46008	-1.20085
Al	0.43663	0.30839	-0.81186
O	0.83532	0.05321	-2.56477
C	1.97766	0.20082	-3.16823
C	1.93440	-0.18407	-4.63136
O	1.81590	-0.42711	0.09825
C	3.62004	-2.37596	1.22297
C	4.83797	-3.15018	1.73824
F	6.63006	1.41429	1.82169
C	-2.39710	1.08530	-1.05495
C	-2.83882	-0.23860	-0.76731
C	-4.22863	-0.42267	-0.64915
C	-4.69969	-1.67607	-0.42356
C	-3.83962	-2.76765	-0.34158
C	-2.46805	-2.63701	-0.44476
C	-1.93530	-1.32294	-0.61950
O	-0.65646	-1.13062	-0.63309

C	-1.55904	-3.86481	-0.38123
C	-0.62918	-3.78052	0.83520
F	-6.02082	-1.89100	-0.28617
O	-0.31715	0.88683	1.29953
C	-0.99855	0.03930	2.21787
C	0.13722	0.86102	2.64335
C	-0.73186	-3.97895	-1.67093
C	-2.36525	-5.16023	-0.24630
C	3.21700	-3.02308	-0.11102
C	2.50715	-2.54043	2.26803
O	2.99486	0.60068	-2.63854
H	-3.18969	1.82577	-1.15405
H	2.78916	3.38109	-0.12374
H	4.77380	2.92788	0.79889
H	5.89214	-1.01125	1.89616
H	-4.28789	-3.74036	-0.19437
H	-4.90261	0.42105	-0.72931
H	1.94690	4.72128	-1.37516
H	0.41767	6.31984	-2.39952
H	-1.90944	5.66271	-2.93500
H	-2.69302	3.39783	-2.43861
H	5.68680	-3.08604	1.05467
H	4.57727	-4.20521	1.83242
H	5.16461	-2.81457	2.72432
H	2.28674	-2.63064	-0.50903
H	3.09203	-4.09927	0.02833
H	3.99506	-2.87472	-0.86183
H	2.83048	-2.15898	3.23877
H	2.25510	-3.59587	2.39240
H	1.59711	-2.01852	1.98252
H	-1.20269	-3.69685	1.76182
H	0.05171	-2.93792	0.76928
H	-0.02209	-4.68581	0.90660

H	-0.10893	-4.87532	-1.63181
H	-0.07559	-3.12611	-1.82323
H	-1.38333	-4.06739	-2.54223
H	-2.94901	-5.19560	0.67590
H	-1.67864	-6.00743	-0.22249
H	-3.04264	-5.31854	-1.08766
H	1.10130	0.38933	2.80828
H	-0.03626	1.77600	3.20170
H	1.68698	-1.24221	-4.73246
H	1.15321	0.37212	-5.15123
H	2.88887	0.00185	-5.11726
C	-2.41842	0.37947	2.47333
C	-3.31755	-0.64107	2.75452
C	-4.66191	-0.36170	2.95229
C	-5.11892	0.94360	2.86209
C	-4.22501	1.96769	2.57406
C	-2.88207	1.68924	2.37996
H	-2.97022	-1.66659	2.79729
H	-5.35339	-1.16693	3.16154
H	-6.16845	1.16367	3.00696
H	-4.57765	2.98805	2.49611
H	-2.20136	2.49488	2.13335
H	-0.80151	-1.02135	2.07809

94

E_el=-6900249.78, G_thr=1758.28, G_solv=-236.51, PBEh-3c/CPCM, TS 1a

C	4.58257	1.19976	-0.74914
C	3.21817	0.87164	-0.88696
C	2.72236	-0.39443	-0.46678
C	3.64228	-1.33313	0.10282
C	4.96646	-0.96360	0.20887
C	5.42574	0.28291	-0.21088
C	2.40168	1.86763	-1.49710
N	1.13268	1.79613	-1.70747
C	0.42072	2.79178	-2.37668
C	-0.97920	2.66135	-2.32821
C	-1.78173	3.57938	-3.00065
C	-1.21183	4.63486	-3.68994
C	0.17035	4.76699	-3.73641
C	0.98040	3.84611	-3.09581
N	-1.45010	1.55255	-1.62531
Al	0.01166	0.23558	-1.12447
O	-0.08508	-0.45274	-2.85574
C	0.82318	-0.67359	-3.74568
C	0.27790	-1.34983	-4.99263
O	1.47891	-0.70546	-0.58212
C	3.16702	-2.68862	0.62771
C	2.44677	-3.49455	-0.46536
F	6.73825	0.56159	-0.06849
C	-2.66988	1.45138	-1.22945
C	-3.25289	0.31551	-0.58911
C	-4.61201	0.43225	-0.24652
C	-5.24638	-0.64121	0.29077
C	-4.58863	-1.85500	0.46618
C	-3.25504	-2.02075	0.14885
C	-2.53218	-0.88599	-0.34266
O	-1.26521	-0.96796	-0.56038

C	-2.57732	-3.38189	0.31099
C	-1.45738	-3.31067	1.35602
F	-6.54525	-0.55493	0.64795
O	-0.05381	1.17113	0.60447
C	-0.51242	0.67635	1.84097
C	0.73744	1.35475	2.10868
C	-2.01894	-3.85735	-1.03875
C	-3.56581	-4.45368	0.78443
C	2.24167	-2.46512	1.83197
C	4.34049	-3.54720	1.11161
O	2.00274	-0.38498	-3.65142
H	-3.35263	2.28522	-1.39130
H	2.93719	2.76552	-1.80165
H	4.94644	2.16710	-1.07420
H	5.69417	-1.63984	0.63439
H	-5.16599	-2.67873	0.86246
H	-5.13712	1.36574	-0.40750
H	2.05359	3.94769	-3.18284
H	0.62094	5.57737	-4.29355
H	-1.84525	5.33949	-4.21195
H	-2.85732	3.46197	-3.01312
H	2.20273	-4.48890	-0.08415
H	3.08569	-3.62088	-1.34142
H	1.52175	-3.02644	-0.78758
H	1.34539	-1.91542	1.55712
H	2.74711	-1.90429	2.61882
H	1.92488	-3.42623	2.24479
H	4.87266	-3.09239	1.94912
H	5.05988	-3.74967	0.31519
H	3.96069	-4.50899	1.45957
H	-0.67409	-2.61351	1.07537
H	-0.99479	-4.29296	1.47658
H	-1.85240	-3.00989	2.32895

H	-1.55928	-4.84152	-0.92151
H	-1.26614	-3.18586	-1.44321
H	-2.81954	-3.95404	-1.77520
H	-3.04475	-5.40875	0.86687
H	-4.39068	-4.59283	0.08289
H	-3.98408	-4.22902	1.76763
H	0.75121	2.42427	2.24464
H	1.67303	0.82247	2.02931
H	-0.47483	-0.71664	-5.46546
H	1.06598	-1.55634	-5.71321
H	-0.21858	-2.28514	-4.73017
C	-1.82318	1.23016	2.29510
C	-2.19002	2.54092	2.00433
C	-3.42837	3.02586	2.39415
C	-4.31826	2.20562	3.07682
C	-3.95875	0.89908	3.36876
C	-2.71755	0.41660	2.97761
H	-1.51208	3.18199	1.45449
H	-3.70264	4.04741	2.16240
H	-5.28811	2.58299	3.37495
H	-4.64880	0.24974	3.89197
H	-2.44616	-0.60960	3.19481
H	-0.47026	-0.40887	1.94866
O	0.88924	1.21300	4.24109
C	1.94756	0.68719	4.68966
O	2.95962	0.39216	4.04427
C	1.91811	0.38904	6.19375
H	2.88742	0.05846	6.56355
H	1.60386	1.26892	6.75724
H	1.18537	-0.39395	6.40146

94

E_el=-6900432.29, G_thr=1776.20, G_solv=-213.67, PBEh-3c/CPCM, Int. 4a

C	4.70730	0.74615	-0.81150
C	3.31703	0.52895	-0.84234
C	2.77408	-0.76302	-0.58306
C	3.68628	-1.84836	-0.35015
C	5.03971	-1.58196	-0.34325
C	5.53767	-0.29943	-0.55947
C	2.51806	1.66134	-1.19037
N	1.23930	1.68797	-1.31434
C	0.53884	2.83485	-1.67770
C	-0.86324	2.76876	-1.54175
C	-1.62712	3.90073	-1.82559
C	-1.03298	5.06352	-2.28017
C	0.34300	5.11161	-2.46337
C	1.11819	4.00644	-2.16562
N	-1.37278	1.54385	-1.11145
Al	0.07019	0.16452	-0.68756
O	-0.24277	-0.40733	-2.47947
C	0.55344	-0.62354	-3.46282
C	-0.11434	-1.35337	-4.62306
O	1.51592	-0.97618	-0.54450
C	3.16155	-3.26600	-0.12214
C	2.36338	-3.73894	-1.34706
F	6.87767	-0.11469	-0.52270
C	-2.63253	1.30997	-1.01801
C	-3.24303	0.09055	-0.58756
C	-4.64935	0.08383	-0.57136
C	-5.30085	-1.03292	-0.15541
C	-4.60362	-2.17081	0.24185
C	-3.22492	-2.21860	0.24675
C	-2.49972	-1.04793	-0.16137
O	-1.22095	-1.04399	-0.12311

C	-2.48604	-3.48695	0.67652
C	-1.64671	-3.22053	1.93449
F	-6.65311	-1.06357	-0.12387
O	0.32368	0.92270	0.96948
C	-0.31953	0.70508	2.14968
C	0.50264	1.28900	3.30410
C	-1.58584	-3.99643	-0.45962
C	-3.45840	-4.62051	1.01995
C	2.28405	-3.32124	1.13766
C	4.30386	-4.26633	0.08259
O	1.73096	-0.30981	-3.53221
H	-3.34499	2.08942	-1.28525
H	3.08990	2.57268	-1.36366
H	5.10654	1.73678	-0.99468
H	5.75757	-2.37112	-0.16583
H	-5.18665	-3.02901	0.54685
H	-5.20023	0.96559	-0.87589
H	2.18632	4.06376	-2.32536
H	0.81291	6.01089	-2.83897
H	-1.64458	5.92903	-2.49800
H	-2.70038	3.88779	-1.69266
H	2.01974	-4.76517	-1.19259
H	2.98743	-3.72655	-2.24270
H	1.49256	-3.11791	-1.53516
H	1.40577	-2.68815	1.05506
H	2.84970	-3.00693	2.01788
H	1.94459	-4.34600	1.31051
H	4.90833	-4.03344	0.96202
H	4.96691	-4.32249	-0.78333
H	3.88549	-5.26293	0.23410
H	-0.88461	-2.46498	1.76640
H	-1.14320	-4.13818	2.24905
H	-2.28107	-2.89169	2.76095

H	-1.08623	-4.91863	-0.15180
H	-0.81875	-3.27815	-0.73348
H	-2.17772	-4.22103	-1.34949
H	-2.88951	-5.50609	1.30826
H	-4.08524	-4.90114	0.17084
H	-4.11125	-4.37068	1.85918
H	0.02057	1.08628	4.26380
H	0.61078	2.36966	3.19331
H	-1.00938	-0.82188	-4.95024
H	0.56100	-1.46577	-5.46892
H	-0.43933	-2.34456	-4.30019
C	-1.69970	1.34430	2.21924
C	-1.88050	2.66440	1.81221
C	-3.13510	3.25301	1.84769
C	-4.23545	2.52987	2.29478
C	-4.06617	1.21804	2.70979
C	-2.80607	0.63527	2.67117
H	-1.03255	3.22499	1.43720
H	-3.25667	4.27899	1.52102
H	-5.21758	2.98594	2.31435
H	-4.91818	0.63926	3.04345
H	-2.68581	-0.39958	2.97136
H	-0.45673	-0.36791	2.38052
O	1.77787	0.65437	3.34775
C	2.84800	1.28467	2.86842
O	2.89212	2.45284	2.58655
C	4.01156	0.33915	2.77297
H	4.91414	0.87248	2.48943
H	4.17484	-0.17697	3.71960
H	3.80538	-0.42243	2.02028

88

E_el=-6302019.06, G_thr=1669.90, G_solv=-214.75, PBEh-3c/CPCM, Int. 5a

C	2.59352	-0.31665	-0.45668
C	2.95661	-1.62081	-0.00743
C	4.28260	-2.08573	-0.11926
C	5.22275	-1.26988	-0.66164
C	4.90138	0.01344	-1.10066
C	3.62150	0.51937	-1.01550
C	2.02039	-2.53788	0.56679
N	0.77349	-2.31475	0.79000
Al	-0.09161	-0.47448	0.58984
O	-1.02797	-1.02166	-0.87903
C	-0.57746	-1.60414	-2.03127
C	-1.48240	-2.78282	-2.42157
F	6.50232	-1.68852	-0.78354
C	3.30958	1.94004	-1.48743
C	2.28188	1.91799	-2.62797
O	1.38797	0.10623	-0.38316
C	-0.06869	-3.26974	1.36079
C	-1.29586	-2.78586	1.85672
C	-2.17226	-3.68113	2.47147
C	-1.86238	-5.02529	2.56947
C	-0.67019	-5.50615	2.04334
C	0.21594	-4.63229	1.44063
N	-1.52989	-1.42304	1.66796
C	-2.63496	-0.85739	2.00546
C	-2.99556	0.50868	1.79794
C	-4.26878	0.88305	2.27123
C	-4.66980	2.17406	2.13917
C	-3.84291	3.13163	1.55658
C	-2.58703	2.82042	1.07908
C	-2.13702	1.46042	1.17576
O	-0.99142	1.13388	0.70716

C	-1.67844	3.90666	0.50269
C	-1.26659	3.58816	-0.94262
F	-5.88649	2.56267	2.58352
O	0.90665	-0.07229	2.17166
C	0.60185	0.36484	3.33932
O	-0.52297	0.55183	3.77626
C	1.82334	0.65476	4.20251
C	-2.37658	5.27055	0.47166
C	-0.43379	4.05540	1.39160
C	2.78528	2.78278	-0.31600
C	4.55848	2.64819	-2.02130
C	-0.51921	-0.62894	-3.19788
H	2.44746	-3.50263	0.84286
H	-3.40910	-1.44790	2.49350
H	-4.91236	0.14749	2.73940
H	-4.22117	4.14261	1.49442
H	5.70270	0.61108	-1.51288
H	4.54122	-3.07967	0.22646
H	-3.11428	-3.34096	2.87948
H	-2.55946	-5.70078	3.04717
H	-0.43457	-6.56076	2.09468
H	1.12883	-5.02610	1.01378
H	-1.69687	6.01179	0.04816
H	-3.27405	5.26037	-0.15077
H	-2.65499	5.62193	1.46735
H	0.22395	4.82775	0.98579
H	-0.71719	4.35082	2.40346
H	0.13629	3.13318	1.45793
H	-2.14676	3.48970	-1.58216
H	-0.66029	4.40568	-1.34102
H	-0.68643	2.67440	-1.02171
H	2.58281	3.80192	-0.65465
H	1.86479	2.38666	0.10187

H	3.52511	2.83915	0.48541
H	4.98786	2.13792	-2.88584
H	4.28559	3.65432	-2.34359
H	5.33669	2.75309	-1.26198
H	1.33645	1.47400	-2.33239
H	2.07987	2.93767	-2.96473
H	2.65881	1.35570	-3.48454
H	2.44618	1.41472	3.72688
H	2.44078	-0.23932	4.30259
H	1.54183	1.00245	5.19438
H	-1.14282	-3.27553	-3.33511
H	-2.50539	-2.43760	-2.58511
H	-1.49987	-3.52579	-1.62136
H	0.44437	-2.01817	-1.93314
C	0.26565	-0.90526	-4.31499
C	0.31557	-0.03208	-5.39168
C	-0.42472	1.14332	-5.36834
C	-1.21124	1.42760	-4.26061
C	-1.25790	0.54885	-3.18636
H	0.85904	-1.81360	-4.33481
H	0.94129	-0.26309	-6.24520
H	-0.38215	1.83259	-6.20236
H	-1.78543	2.34572	-4.22594
H	-1.85703	0.78651	-2.31787

15

E_el=-1399417.03, G_thr=177.90, G_solv=-37.42, PBEh-3c/CPCM, Phthalic Acid

H	-3.54074	1.22456	-0.00088
C	-2.59627	0.69735	-0.00024
C	-2.59628	-0.69733	-0.00025
H	-3.54075	-1.22452	-0.00094
C	-1.40843	-1.41952	0.00018
H	-1.40405	-2.50097	0.00007
C	-0.23662	-0.69022	0.00093
C	1.17008	-1.13190	0.00029
O	1.63792	-2.22264	-0.00069
H	-1.40402	2.50098	0.00008
C	-1.40841	1.41952	0.00016
C	-0.23662	0.69022	0.00091
C	1.17008	1.13188	0.00025
O	1.63794	2.22262	-0.00090
O	1.94904	-0.00001	-0.00013

88

E_el=-6302019.06, G_thr=1669.90, G_solv=-214.75, PBEh-3c/CPCM, Int. 5a

C	2.59352	-0.31665	-0.45668
C	2.95661	-1.62081	-0.00743
C	4.28260	-2.08573	-0.11926
C	5.22275	-1.26988	-0.66164
C	4.90138	0.01344	-1.10066
C	3.62150	0.51937	-1.01550
C	2.02039	-2.53788	0.56679
N	0.77349	-2.31475	0.79000
Al	-0.09161	-0.47448	0.58984
O	-1.02797	-1.02166	-0.87903
C	-0.57746	-1.60414	-2.03127
C	-1.48240	-2.78282	-2.42157
F	6.50232	-1.68852	-0.78354
C	3.30958	1.94004	-1.48743
C	2.28188	1.91799	-2.62797
O	1.38797	0.10623	-0.38316
C	-0.06869	-3.26974	1.36079
C	-1.29586	-2.78586	1.85672
C	-2.17226	-3.68113	2.47147
C	-1.86238	-5.02529	2.56947
C	-0.67019	-5.50615	2.04334
C	0.21594	-4.63229	1.44063
N	-1.52989	-1.42304	1.66796
C	-2.63496	-0.85739	2.00546
C	-2.99556	0.50868	1.79794
C	-4.26878	0.88305	2.27123
C	-4.66980	2.17406	2.13917
C	-3.84291	3.13163	1.55658
C	-2.58703	2.82042	1.07908
C	-2.13702	1.46042	1.17576
O	-0.99142	1.13388	0.70716

C	-1.67844	3.90666	0.50269
C	-1.26659	3.58816	-0.94262
F	-5.88649	2.56267	2.58352
O	0.90665	-0.07229	2.17166
C	0.60185	0.36484	3.33932
O	-0.52297	0.55183	3.77626
C	1.82334	0.65476	4.20251
C	-2.37658	5.27055	0.47166
C	-0.43379	4.05540	1.39160
C	2.78528	2.78278	-0.31600
C	4.55848	2.64819	-2.02130
C	-0.51921	-0.62894	-3.19788
H	2.44746	-3.50263	0.84286
H	-3.40910	-1.44790	2.49350
H	-4.91236	0.14749	2.73940
H	-4.22117	4.14261	1.49442
H	5.70270	0.61108	-1.51288
H	4.54122	-3.07967	0.22646
H	-3.11428	-3.34096	2.87948
H	-2.55946	-5.70078	3.04717
H	-0.43457	-6.56076	2.09468
H	1.12883	-5.02610	1.01378
H	-1.69687	6.01179	0.04816
H	-3.27405	5.26037	-0.15077
H	-2.65499	5.62193	1.46735
H	0.22395	4.82775	0.98579
H	-0.71719	4.35082	2.40346
H	0.13629	3.13318	1.45793
H	-2.14676	3.48970	-1.58216
H	-0.66029	4.40568	-1.34102
H	-0.68643	2.67440	-1.02171
H	2.58281	3.80192	-0.65465
H	1.86479	2.38666	0.10187

H	3.52511	2.83915	0.48541
H	4.98786	2.13792	-2.88584
H	4.28559	3.65432	-2.34359
H	5.33669	2.75309	-1.26198
H	1.33645	1.47400	-2.33239
H	2.07987	2.93767	-2.96473
H	2.65881	1.35570	-3.48454
H	2.44618	1.41472	3.72688
H	2.44078	-0.23932	4.30259
H	1.54183	1.00245	5.19438
H	-1.14282	-3.27553	-3.33511
H	-2.50539	-2.43760	-2.58511
H	-1.49987	-3.52579	-1.62136
H	0.44437	-2.01817	-1.93314
C	0.26565	-0.90526	-4.31499
C	0.31557	-0.03208	-5.39168
C	-0.42472	1.14332	-5.36834
C	-1.21124	1.42760	-4.26061
C	-1.25790	0.54885	-3.18636
H	0.85904	-1.81360	-4.33481
H	0.94129	-0.26309	-6.24520
H	-0.38215	1.83259	-6.20236
H	-1.78543	2.34572	-4.22594
H	-1.85703	0.78651	-2.31787

103

E_el=-7701427.43, G_thr=1935.08, G_solv=-240.79, PBEh-3c/CPCM, TS 2a

C	2.83282	-0.53459	-0.21934
C	3.01748	-1.91607	0.06287
C	4.24085	-2.55836	-0.20635
C	5.25851	-1.82927	-0.73265
C	5.12570	-0.46176	-0.95586
C	3.95418	0.21887	-0.69340
C	2.01764	-2.71870	0.68831
N	0.81421	-2.36128	0.98174
Al	0.12603	-0.48310	0.75833
O	-0.73734	-1.12359	-1.03720
C	0.10696	-1.83416	-1.93082
C	-0.21847	-3.32733	-2.01958
F	6.43945	-2.41325	-1.02477
C	3.89656	1.74068	-0.83360
C	2.76358	2.19658	-1.76244
O	1.68578	0.03164	-0.05883
C	-0.08793	-3.22791	1.60660
C	-1.28196	-2.63631	2.05249
C	-2.23213	-3.42104	2.70283
C	-2.01541	-4.77088	2.90126
C	-0.84609	-5.36162	2.43783
C	0.10709	-4.59632	1.79277
N	-1.41420	-1.27332	1.77919
C	-2.32444	-0.54463	2.32992
C	-2.50777	0.85865	2.15717
C	-3.58992	1.42755	2.86162
C	-3.81718	2.75880	2.74316
C	-2.98939	3.56818	1.96593
C	-1.91685	3.06621	1.26312
C	-1.67323	1.65376	1.32349
O	-0.72840	1.12383	0.62916

C	-1.00785	4.00182	0.46648
C	-0.97629	3.62585	-1.02144
F	-4.84912	3.33912	3.39137
O	0.76563	0.01526	2.47590
C	1.83562	-0.14655	3.16807
O	2.75627	-0.90475	2.90814
C	1.88785	0.73664	4.40412
C	-1.48434	5.45587	0.54259
C	0.40634	3.95539	1.06106
C	3.72244	2.35375	0.56387
C	5.19638	2.30545	-1.41790
C	0.28461	-1.15563	-3.27914
H	2.34631	-3.72692	0.93258
H	-3.03091	-1.00982	3.01466
H	-4.22715	0.80305	3.47613
H	-3.22282	4.62303	1.93239
H	5.99063	0.06182	-1.33764
H	4.36230	-3.61309	0.00884
H	-3.16544	-2.98963	3.03609
H	-2.76755	-5.36610	3.40154
H	-0.67938	-6.42191	2.57210
H	1.00197	-5.08351	1.43031
H	-0.79826	6.08395	-0.02767
H	-2.47872	5.58803	0.11110
H	-1.49876	5.83868	1.56507
H	1.07894	4.59425	0.48336
H	0.40567	4.31673	2.09158
H	0.81770	2.95074	1.05605
H	-1.98140	3.62210	-1.44310
H	-0.37919	4.35856	-1.56955
H	-0.54104	2.64695	-1.19375
H	3.65155	3.44156	0.48971
H	2.82317	1.99978	1.06167

H	4.57636	2.11541	1.20080
H	5.39244	1.92722	-2.42342
H	5.11109	3.39066	-1.48971
H	6.06624	2.09353	-0.79348
H	1.78119	2.00588	-1.34377
H	2.84560	3.27193	-1.93812
H	2.82009	1.69454	-2.72920
H	0.97824	0.63149	4.99694
H	1.94612	1.78477	4.10244
H	2.74923	0.50707	5.02760
H	-1.04108	-3.54435	-2.69587
H	-0.47413	-3.71320	-1.03302
H	0.65727	-3.88544	-2.35630
H	1.10824	-1.79421	-1.50473
C	0.61789	-1.88723	-4.41670
C	0.88136	-1.25836	-5.62681
C	0.82052	0.12265	-5.72577
C	0.49971	0.86306	-4.59556
C	0.24309	0.23243	-3.38892
H	0.66428	-2.96733	-4.37484
H	1.12931	-1.85583	-6.49554
H	1.01914	0.61602	-6.66873
H	0.44587	1.94336	-4.65134
H	-0.00843	0.83101	-2.52656
H	-4.82567	-4.12055	0.87037
C	-4.61352	-3.11083	0.54216
C	-5.43717	-2.06838	0.97105
H	-6.27660	-2.28366	1.61968
C	-5.19793	-0.76705	0.55348
H	-5.83647	0.05235	0.85726
C	-4.10953	-0.55517	-0.27400
C	-3.64794	0.69292	-0.91699
O	-4.14420	1.78548	-0.84130

H	-2.90908	-3.70048	-0.63334
C	-3.53218	-2.88188	-0.29821
C	-3.28029	-1.57775	-0.68047
C	-2.34946	-1.03815	-1.73227
O	-2.34365	-1.51503	-2.85173
O	-2.57728	0.41139	-1.65262

103

E_el=-7701561.50, G_thr=1936.69, G_solv=-222.78, PBEh-3c/CPCM, Int. 6a

C	-2.96506	-3.18312	1.49557
C	-3.01107	-1.80571	1.68545
C	-3.88614	-1.05377	0.91169
C	-4.70629	-1.65831	-0.02995
C	-4.65244	-3.03212	-0.21592
C	-3.77527	-3.79036	0.54857
C	-2.18778	-1.12276	2.75322
C	-2.85193	-1.27800	4.11484
O	-0.88039	-1.64314	2.89910
C	0.01344	-1.46482	1.84013
C	1.39241	-1.19841	2.41707
C	1.86408	-0.07478	3.06541
C	3.20543	-0.04978	3.42260
C	4.04940	-1.12754	3.15322
C	3.56152	-2.25933	2.52046
C	2.22471	-2.26200	2.16101
C	1.44057	-3.29952	1.46179
O	1.82478	-4.38286	1.09760
O	-0.41334	-0.64921	0.93375
O	0.21168	-2.84775	1.27945
Al	0.10244	0.28607	-0.60242
O	1.51876	1.13976	0.21402
C	2.78792	0.97967	0.12263
C	3.35399	-0.21492	-0.40473
C	4.74371	-0.42896	-0.37579
C	5.55020	0.53947	0.13242
C	5.03218	1.75377	0.57279
C	3.67742	2.01616	0.56107
C	2.56996	-1.26360	-0.98850
N	1.29448	-1.26496	-1.14078
C	0.59421	-2.33637	-1.70533

C	-0.76894	-2.10226	-1.94471
C	-1.57361	-3.13259	-2.41740
C	-1.02640	-4.36963	-2.70499
C	0.32807	-4.59343	-2.49780
C	1.12900	-3.58811	-1.98725
C	3.14494	3.40320	0.92564
C	4.27103	4.34690	1.36111
F	6.88806	0.35127	0.19113
N	-1.22321	-0.82096	-1.63764
C	-2.32765	-0.35092	-2.08755
C	-2.89892	0.92006	-1.74958
C	-2.33322	1.78350	-0.76872
C	-3.08113	2.94336	-0.37510
C	-4.27674	3.20379	-1.01453
C	-4.77840	2.36586	-2.00615
C	-4.12226	1.23134	-2.36885
O	-1.20443	1.53419	-0.21594
C	-2.56868	3.86175	0.73499
C	-2.40006	3.07306	2.04363
F	-5.95278	2.69116	-2.59153
O	0.52360	1.23963	-2.18271
C	0.77455	0.93769	-3.40288
O	0.73680	-0.17967	-3.89473
C	1.13698	2.14645	-4.25486
C	-3.54863	5.00339	1.02392
C	-1.23345	4.50165	0.33016
C	2.13622	3.35839	2.08221
C	2.49257	4.02140	-0.32159
H	3.14969	-2.11526	-1.34306
H	-2.91785	-0.94980	-2.78082
H	-4.53603	0.56760	-3.11907
H	-4.86296	4.07472	-0.75563
H	5.73603	2.49921	0.91678

H	5.15934	-1.35659	-0.75113
H	-2.63933	-2.98611	-2.52947
H	-1.66150	-5.16689	-3.06805
H	0.75791	-5.56485	-2.70247
H	2.16783	-3.80758	-1.78540
H	-1.34604	5.08660	-0.58503
H	-0.45669	3.76111	0.16606
H	-0.88974	5.17849	1.11681
H	-2.07779	3.74509	2.84296
H	-1.66181	2.28064	1.95321
H	-3.34823	2.62588	2.35150
H	-4.52572	4.64053	1.35007
H	-3.69648	5.65363	0.15914
H	-3.14949	5.62423	1.82776
H	1.68766	3.40698	-0.71722
H	3.23284	4.15549	-1.11322
H	2.07954	5.00472	-0.08168
H	5.01483	4.49974	0.57663
H	4.78646	3.99223	2.25662
H	3.84842	5.32512	1.59680
H	1.25073	2.77997	1.83838
H	1.81663	4.37433	2.32788
H	2.59312	2.93244	2.97796
H	0.30134	2.84819	-4.28498
H	1.97961	2.68163	-3.81418
H	1.39037	1.85932	-5.27327
H	-2.14329	-0.05604	2.50923
H	-2.93461	-2.32813	4.39900
H	-2.28516	-0.75527	4.88673
H	-3.85758	-0.85795	4.08865
H	4.20085	-3.10481	2.30150
H	5.09289	-1.07035	3.43372
H	3.61106	0.82799	3.90936

H	1.22112	0.77186	3.26230
H	-3.91473	0.02335	1.03137
H	-5.37470	-1.05006	-0.62664
H	-5.28493	-3.50906	-0.95453
H	-3.71483	-4.86124	0.40064
H	-2.27734	-3.78501	2.07446

70

E_el=-5289657.70, G_thr=1298.81, G_solv=-92.59, PBEh-3c/CPCM, Int. 2

C	4.73801	-0.84444	-0.48214
C	3.33625	-0.73767	-0.39368
C	2.69631	0.51824	-0.25532
C	3.49888	1.69827	-0.22465
C	4.86935	1.54293	-0.29504
C	5.47524	0.29394	-0.41741
C	2.59088	-1.94379	-0.50420
N	1.31212	-2.05289	-0.36710
Al	0.06949	-0.55326	0.19719
O	0.18119	-0.89750	1.94916
C	-0.85166	-1.10008	2.72674
C	-0.49347	-1.19658	4.18936
O	1.40232	0.59509	-0.18845
C	2.87520	3.09035	-0.13770
C	2.03724	3.34972	-1.39840
F	6.81577	0.23872	-0.48182
C	0.65650	-3.28021	-0.50906
C	1.25425	-4.53623	-0.49530
C	0.47750	-5.67205	-0.63559
C	-0.90146	-5.56274	-0.76690
C	-1.50736	-4.31921	-0.75816
C	-0.73257	-3.16877	-0.64421
N	-1.21618	-1.85573	-0.59377
C	-2.43178	-1.57559	-0.94416
C	-3.08249	-0.32602	-0.79953
C	-2.41927	0.79998	-0.25127
C	-3.17166	1.98762	-0.00031
C	-4.50038	1.99084	-0.37195
C	-5.11806	0.88654	-0.96014
C	-4.44174	-0.27010	-1.17144
O	-1.15068	0.75405	-0.00517

C	-2.54215	3.21094	0.66461
C	-1.45224	3.79775	-0.24088
F	-6.41467	0.98801	-1.29801
C	-1.95968	2.83361	2.03554
C	-3.57493	4.31594	0.90748
C	3.94286	4.18737	-0.07465
C	2.02081	3.22818	1.13102
O	-1.99010	-1.19794	2.32216
H	3.17441	-2.83105	-0.74372
H	-3.04170	-2.36694	-1.37503
H	-4.93199	-1.13574	-1.59953
H	-5.11233	2.86678	-0.20841
H	5.52028	2.40513	-0.26082
H	5.21104	-1.81259	-0.59174
H	-2.58607	-4.26309	-0.81018
H	-1.51087	-6.45211	-0.85072
H	0.94638	-6.64632	-0.61917
H	2.32113	-4.64479	-0.35322
H	-4.00992	4.69337	-0.02005
H	-3.08570	5.15960	1.39590
H	-4.38654	3.99069	1.56134
H	-0.64917	3.09219	-0.42820
H	-1.01290	4.68108	0.22828
H	-1.86931	4.10551	-1.20177
H	-2.72859	2.40452	2.68007
H	-1.57310	3.72836	2.52814
H	-1.14441	2.11965	1.96439
H	2.62219	3.04268	2.02297
H	1.17272	2.55086	1.14584
H	1.62947	4.24525	1.20199
H	1.23853	2.62390	-1.52527
H	2.66673	3.32171	-2.28989
H	1.57987	4.34003	-1.34625

H	4.58259	4.09516	0.80536
H	3.45117	5.15908	-0.01442
H	4.57826	4.20581	-0.96217
H	-1.36569	-1.43354	4.79254
H	-0.07328	-0.24961	4.53216
H	0.27209	-1.95660	4.34804

33

E_el=-2411719.32, G_thr=545.43, G_solv=-226.64, PBEh-3c/CPCM, Free Chain (H)

C	3.01280	-1.58020	0.73082
C	4.20040	-1.46751	0.02534
C	4.45407	-0.31037	-0.69857
C	3.52918	0.72343	-0.70625
C	2.34328	0.63815	0.01636
C	2.08737	-0.54150	0.72043
C	1.43008	1.86474	0.11606
O	1.07454	2.14047	1.27351
C	0.80191	-0.76133	1.43478
O	-0.22577	-0.37452	0.69088
C	-1.54101	-0.37616	1.23916
C	-1.72486	0.73746	2.26158
O	1.18851	2.45028	-0.95088
O	0.69767	-1.31415	2.50179
C	-2.48511	-0.19718	0.07756
H	4.91737	-2.27847	0.03547
H	5.37705	-0.21267	-1.25740
H	3.72793	1.62474	-1.27185
H	2.79541	-2.48494	1.28490
H	-1.73522	-1.34352	1.71503
H	-1.09852	0.58147	3.13792
H	-2.76656	0.75736	2.58472
H	-1.46915	1.70278	1.82881
C	-2.20065	0.69311	-0.95634
C	-3.11795	0.88269	-1.97969
C	-4.32515	0.19606	-1.98562
C	-4.61174	-0.69112	-0.95896
C	-3.69268	-0.88556	0.06312
H	-1.26328	1.23726	-0.97468
H	-2.88395	1.57429	-2.77889
H	-5.03572	0.34975	-2.78795

H	-5.54631	-1.23789	-0.95515
H	-3.91769	-1.58629	0.85949

17

E_el=-2313437.75, G_thr=145.10, G_solv=-23.96, PBEh-3c/CPCM, Fluorinated Styrene Oxide

O	-3.26972	-0.20157	0.34807
C	-3.12480	1.18722	0.55764
C	-2.36211	0.48927	-0.47322
C	-0.91674	0.19605	-0.26854
C	0.00133	1.22579	-0.15052
C	1.34943	0.98293	0.03453
C	1.79925	-0.32389	0.09465
C	0.90504	-1.37160	-0.03450
C	-0.43906	-1.10334	-0.22130
F	-0.41733	2.48639	-0.20993
F	2.20885	1.98608	0.15565
F	3.08713	-0.57208	0.27256
F	1.34354	-2.62220	0.01432
F	-1.27312	-2.12305	-0.36879
H	-3.96657	1.80818	0.26628
H	-2.63459	1.47950	1.48190
H	-2.66688	0.59800	-1.51273

87

E_el=-7603171.58, G_thr=1525.30, G_solv=-99.89, PBEh-3c/CPCM, Int. 3b

C	-4.92574	0.47306	-2.01258
C	-3.77549	0.50527	-1.19988
C	-3.16175	-0.68966	-0.74241
C	-3.76771	-1.93997	-1.07439
C	-4.89984	-1.91839	-1.86497
C	-5.46055	-0.73279	-2.33370
C	-3.31070	1.79649	-0.82443
N	-2.31529	2.05153	-0.04523
C	-1.99165	3.35384	0.34696
C	-0.73616	3.48175	0.95726
C	-0.31836	4.72070	1.43151
C	-1.12937	5.83087	1.27469
C	-2.37336	5.70570	0.66880
C	-2.80998	4.47192	0.21910
N	-0.01867	2.28792	1.07054
Al	-1.16222	0.65210	0.80612
O	-1.86994	0.69327	2.47069
C	-3.12582	0.59874	2.80231
C	-3.34326	0.48190	4.29434
O	-2.06433	-0.65575	-0.05645
C	-3.18632	-3.26894	-0.58971
C	-3.02700	-3.28120	0.93874
F	-6.55929	-0.80558	-3.10554
C	1.26006	2.27767	1.23025
C	2.06767	1.12042	1.42529
C	3.44512	1.34147	1.62209
C	4.24059	0.27386	1.89048
C	3.70848	-1.00749	2.02372
C	2.36502	-1.27294	1.84650
C	1.51669	-0.18690	1.46251
O	0.27670	-0.39610	1.16334

C	1.80447	-2.67180	2.10577
C	1.24420	-3.28220	0.81792
F	5.56226	0.43823	2.07065
O	-0.02214	0.87823	-1.28377
C	0.50161	-0.18536	-2.05034
C	-0.18653	0.93976	-2.69015
C	0.70914	-2.60240	3.18212
C	2.88336	-3.62649	2.62633
C	-1.83502	-3.51417	-1.27254
C	-4.09596	-4.44844	-0.95028
O	-4.04776	0.58883	2.01293
H	1.79634	3.22570	1.23473
H	-3.87641	2.62807	-1.24147
H	-5.38148	1.39384	-2.35579
H	-5.39278	-2.83946	-2.14217
H	4.39229	-1.80019	2.29344
H	3.85347	2.34335	1.57065
H	-3.80183	4.38998	-0.20453
H	-3.01821	6.56823	0.57023
H	-0.80207	6.79060	1.65055
H	0.62471	4.82350	1.95200
H	-3.97492	-3.05516	1.42976
H	-2.28697	-2.56952	1.29163
H	-2.71267	-4.27527	1.26410
H	-1.41026	-4.46534	-0.94456
H	-1.11492	-2.73829	-1.02975
H	-1.94726	-3.55567	-2.35768
H	-5.08879	-4.35528	-0.50588
H	-3.65469	-5.36941	-0.56721
H	-4.21209	-4.57730	-2.02811
H	2.03602	-3.42837	0.08175
H	0.46444	-2.66776	0.37930
H	0.81118	-4.26290	1.02733

H	-0.14688	-2.00760	2.87464
H	1.10186	-2.17933	4.10852
H	0.35058	-3.60959	3.40498
H	3.68812	-3.78236	1.90499
H	2.43415	-4.60202	2.81695
H	3.32403	-3.28488	3.56496
H	-1.19759	0.78390	-3.05442
H	0.37406	1.72168	-3.19083
H	-2.94238	-0.46873	4.65115
H	-2.80951	1.26831	4.82815
H	-4.39993	0.52936	4.54450
C	1.97357	-0.37428	-2.00550
C	2.49386	-1.64970	-2.17593
C	3.83788	-1.92692	-2.01935
C	4.71022	-0.90293	-1.70303
C	4.22943	0.38545	-1.56252
C	2.88002	0.63965	-1.72050
F	1.68149	-2.65669	-2.48550
F	4.28923	-3.16529	-2.16362
F	5.99947	-1.15156	-1.54599
F	5.06524	1.37455	-1.27975
F	2.47922	1.90037	-1.59708
H	-0.06425	-1.10734	-1.96727

94

E_el=-8203308.92, G_thr=1634.81, G_solv=-223.24, PBEh-3c/CPCM, TS 1b

C	-4.59053	1.76392	-1.25993
C	-3.48300	1.16948	-0.62368
C	-3.33611	-0.24342	-0.58244
C	-4.37361	-1.05670	-1.14093
C	-5.43676	-0.42482	-1.75305
C	-5.53369	0.96335	-1.81996
C	-2.53428	2.06985	-0.05519
N	-1.45164	1.75684	0.56927
C	-0.52639	2.71975	0.98352
C	0.68266	2.21368	1.49507
C	1.70828	3.09137	1.83986
C	1.53033	4.45781	1.72625
C	0.32159	4.96202	1.26238
C	-0.69427	4.10056	0.89018
N	0.76635	0.82229	1.57009
Al	-0.81292	-0.15169	0.76441
O	-1.42866	-0.36849	2.50877
C	-2.56285	-0.10620	3.06492
C	-2.62781	-0.54474	4.51778
O	-2.29695	-0.79682	-0.06557
C	-4.32294	-2.58145	-1.03578
C	-3.06981	-3.14755	-1.72014
F	-6.59686	1.50448	-2.45155
C	1.64880	0.21826	2.29180
C	1.80788	-1.19445	2.41546
C	2.79300	-1.63236	3.32506
C	3.00932	-2.96546	3.46103
C	2.30043	-3.89558	2.70182
C	1.33207	-3.52128	1.79427
C	1.04487	-2.12500	1.65454
O	0.12815	-1.73018	0.84175

C	0.57645	-4.56957	0.97754
C	-0.90819	-4.56855	1.37017
F	3.92746	-3.42295	4.33735
O	-0.00525	0.23867	-0.99210
C	1.35370	0.21010	-1.33871
C	0.62331	-0.41235	-2.41980
C	1.11343	-5.98111	1.23650
C	0.73043	-4.30186	-0.52775
C	-5.53202	-3.23417	-1.71396
C	-4.36193	-2.99780	0.44300
O	-3.50895	0.44098	2.52776
H	2.34471	0.81179	2.88412
H	-2.77489	3.12077	-0.20257
H	-4.68404	2.84264	-1.29837
H	-6.23575	-0.99995	-2.19987
H	2.53908	-4.93958	2.84866
H	3.36049	-0.91381	3.90413
H	-1.61737	4.52364	0.51867
H	0.17428	6.02997	1.17498
H	2.33697	5.12845	1.98985
H	2.66553	2.71195	2.17224
H	-2.14948	-2.82542	-1.24296
H	-3.03162	-2.84981	-2.77000
H	-3.09439	-4.23941	-1.68637
H	-6.47806	-2.92757	-1.26327
H	-5.46039	-4.31743	-1.60509
H	-5.57540	-3.01883	-2.78368
H	-3.52362	-2.60268	1.01011
H	-4.33352	-4.08714	0.52303
H	-5.28239	-2.65219	0.91707
H	-1.38262	-3.60958	1.18240
H	-1.03270	-4.80993	2.42777
H	-1.44645	-5.32188	0.78980

H	2.16664	-6.08026	0.96575
H	0.55444	-6.69152	0.62580
H	0.99470	-6.29283	2.27662
H	0.27828	-5.12139	-1.09146
H	1.77892	-4.23433	-0.81951
H	0.24351	-3.38223	-0.83679
H	0.15758	0.16938	-3.19677
H	0.43341	-1.47080	-2.36014
H	-3.60508	-0.34501	4.95140
H	-2.41125	-1.61055	4.60367
H	-1.86928	-0.02251	5.10345
C	1.99978	1.55705	-1.45484
C	3.31908	1.73337	-1.06308
C	3.92245	2.97849	-1.03186
C	3.19614	4.09627	-1.38927
C	1.88419	3.95519	-1.79647
C	1.30791	2.69929	-1.84029
F	4.04595	0.69099	-0.67235
F	5.18768	3.10878	-0.64104
F	3.75077	5.30143	-1.33770
F	1.18112	5.03063	-2.13920
F	0.05151	2.63397	-2.26482
H	1.96487	-0.48521	-0.76738
O	2.09532	-0.86487	-3.94305
C	2.95293	-1.72155	-3.58222
O	3.06645	-2.23233	-2.46309
C	3.91590	-2.16423	-4.69125
H	3.38291	-2.78751	-5.41311
H	4.30308	-1.30643	-5.24272
H	4.75115	-2.74177	-4.29793

94

E_el=-8203489.82, G_thr=1647.58, G_solv=-208.04, PBEh-3c/CPCM, Int. 4b

C	-4.81335	1.71948	-1.08374
C	-3.65422	1.13629	-0.53656
C	-3.49589	-0.27825	-0.49842
C	-4.53523	-1.09446	-1.06058
C	-5.64795	-0.47176	-1.58493
C	-5.78359	0.91496	-1.58917
C	-2.67257	2.04707	-0.04596
N	-1.52528	1.75052	0.45629
C	-0.59496	2.72075	0.82605
C	0.61832	2.23678	1.35675
C	1.61820	3.14078	1.71265
C	1.43166	4.50231	1.56183
C	0.23704	4.98055	1.04090
C	-0.76175	4.09755	0.67546
N	0.73338	0.84758	1.43628
Al	-0.87315	-0.16033	0.65212
O	-1.45556	-0.20620	2.45657
C	-2.53834	0.14564	3.04990
C	-2.54080	-0.19418	4.53525
O	-2.46111	-0.82943	0.01066
C	-4.40465	-2.61725	-1.07824
C	-3.16214	-3.03871	-1.87847
F	-6.90751	1.44962	-2.11825
C	1.70187	0.26841	2.05632
C	1.92165	-1.13755	2.16832
C	3.07683	-1.52866	2.87647
C	3.36479	-2.84976	2.98819
C	2.54792	-3.82168	2.40965
C	1.40754	-3.49654	1.70881
C	1.05674	-2.10807	1.58278
O	0.00117	-1.77286	0.94734

C	0.53321	-4.57923	1.07748
C	-0.86477	-4.56839	1.71343
F	4.46373	-3.25499	3.66204
O	-0.20481	-0.01036	-1.05998
C	1.07819	-0.11795	-1.48359
C	1.09178	-0.69792	-2.90412
C	1.12010	-5.97714	1.29773
C	0.42785	-4.36959	-0.44130
C	-5.61235	-3.28269	-1.74625
C	-4.32696	-3.16129	0.35595
O	-3.50065	0.69915	2.54303
H	2.45259	0.87753	2.56012
H	-2.95570	3.09424	-0.14354
H	-4.92655	2.79714	-1.09645
H	-6.45400	-1.05121	-2.01376
H	2.84577	-4.85431	2.52874
H	3.72477	-0.78060	3.31791
H	-1.67332	4.50016	0.25632
H	0.08692	6.04318	0.90514
H	2.22359	5.18651	1.83549
H	2.56842	2.79001	2.09252
H	-2.24210	-2.65204	-1.45009
H	-3.23163	-2.68745	-2.91031
H	-3.08890	-4.12894	-1.90357
H	-6.54626	-3.07593	-1.21928
H	-5.47295	-4.36510	-1.74223
H	-5.73199	-2.97654	-2.78780
H	-3.46836	-2.77388	0.89607
H	-4.24900	-4.25139	0.33735
H	-5.22716	-2.90025	0.91584
H	-1.37598	-3.62169	1.56414
H	-0.80419	-4.76029	2.78690
H	-1.48190	-5.35375	1.26972

H	2.10864	-6.08694	0.84681
H	0.46795	-6.71686	0.83063
H	1.19430	-6.23703	2.35597
H	-0.13321	-5.19495	-0.88690
H	1.41475	-4.34584	-0.90546
H	-0.08327	-3.44566	-0.69528
H	0.46964	-0.09379	-3.56423
H	0.69493	-1.71369	-2.88095
H	-3.45632	0.13712	5.02099
H	-2.43720	-1.27160	4.67595
H	-1.68741	0.26938	5.03304
C	1.80799	1.23246	-1.48542
C	3.10661	1.36143	-1.02329
C	3.75288	2.58081	-0.93320
C	3.09615	3.72693	-1.33191
C	1.80918	3.63594	-1.82204
C	1.18697	2.40221	-1.90218
F	3.78777	0.28442	-0.61770
F	4.99560	2.66018	-0.45581
F	3.69552	4.91046	-1.23622
F	1.17791	4.73814	-2.21951
F	-0.04079	2.38733	-2.40915
H	1.68366	-0.79865	-0.86384
O	2.40559	-0.66467	-3.47378
C	3.23312	-1.68592	-3.26743
O	2.93266	-2.70892	-2.71316
C	4.60497	-1.38093	-3.80401
H	5.18076	-2.29656	-3.90799
H	4.56168	-0.86211	-4.76008
H	5.12503	-0.72787	-3.10076

88

E_el=-7605097.46, G_thr=1544.57, G_solv=-203.41, PBEh-3c/CPCM, Int. 5b

C	-3.28039	0.29584	0.38561
C	-3.34493	-1.10214	0.65142
C	-4.49003	-1.67251	1.24126
C	-5.54261	-0.87324	1.55288
C	-5.51240	0.49616	1.29573
C	-4.41858	1.10592	0.71993
C	-2.28637	-2.00814	0.34473
N	-1.14631	-1.71565	-0.17511
Al	-0.56445	0.19720	-0.50935
O	-0.08087	0.25430	1.25920
C	1.14307	0.47427	1.80751
C	1.04213	1.18623	3.15945
F	-6.65281	-1.39576	2.12219
C	-4.41395	2.60751	0.43355
C	-5.71894	3.27382	0.88122
O	-2.24666	0.83970	-0.13083
C	-0.15547	-2.66992	-0.39819
C	1.08472	-2.17521	-0.85211
C	2.14046	-3.06264	-1.05623
C	1.98252	-4.41847	-0.83708
C	0.75622	-4.90901	-0.40947
C	-0.29917	-4.04265	-0.19505
N	1.15709	-0.79164	-1.03349
C	2.16100	-0.21922	-1.59982
C	2.31890	1.18015	-1.84430
C	3.51868	1.56766	-2.47441
C	3.73532	2.88329	-2.73012
C	2.79656	3.85429	-2.38191
C	1.60818	3.53169	-1.76370
C	1.34114	2.14920	-1.47426
O	0.25697	1.82173	-0.88605

C	0.59265	4.61022	-1.38825
C	0.38276	4.63666	0.13400
F	4.87769	3.28284	-3.33189
O	-0.95753	0.03575	-2.35985
C	-1.98631	-0.38654	-3.00191
O	-2.95013	-0.96957	-2.53306
C	-1.91726	-0.08832	-4.49505
C	1.07073	6.00569	-1.80151
C	-0.74333	4.36192	-2.10539
C	-3.27339	3.30272	1.19313
C	-4.27678	2.85396	-1.07664
C	1.91696	-0.84233	1.98822
H	-2.50286	-3.04334	0.60600
H	2.99290	-0.82773	-1.95505
H	4.25654	0.82204	-2.74649
H	3.03484	4.88262	-2.61701
H	-6.38684	1.07195	1.56640
H	-4.52719	-2.73734	1.43912
H	3.10966	-2.70268	-1.37476
H	2.81659	-5.08885	-0.99525
H	0.62184	-5.96847	-0.23687
H	-1.24220	-4.45449	0.13657
H	1.21915	6.09129	-2.88015
H	0.31503	6.74120	-1.52075
H	1.99994	6.29201	-1.30413
H	-1.45519	5.15014	-1.84729
H	-0.60832	4.37845	-3.18905
H	-1.18924	3.41030	-1.83213
H	1.32191	4.84980	0.64912
H	-0.32665	5.42544	0.39669
H	-0.00923	3.69704	0.51321
H	-3.38047	3.15456	2.26991
H	-2.29517	2.93674	0.89624

H	-3.30243	4.37858	1.00151
H	-3.35238	2.44785	-1.47661
H	-5.10872	2.39979	-1.61841
H	-4.29008	3.92763	-1.28155
H	-5.88801	3.17509	1.95575
H	-5.67120	4.34128	0.65889
H	-6.59107	2.87437	0.35930
H	-1.00368	-0.50074	-4.92648
H	-1.88036	0.99050	-4.65842
H	-2.77525	-0.49427	-5.02712
H	0.55470	2.15203	3.01824
H	2.02727	1.36365	3.59662
H	0.44769	0.61139	3.87072
H	1.78203	1.11448	1.17830
C	1.30368	-1.98897	2.47652
C	1.96077	-3.20480	2.54898
C	3.27732	-3.30120	2.14574
C	3.92556	-2.17717	1.67773
C	3.24326	-0.97533	1.61214
F	0.04432	-1.96898	2.89954
F	1.33333	-4.28599	3.00761
F	3.91160	-4.46979	2.19573
F	5.19407	-2.26341	1.27362
F	3.91738	0.07506	1.13182

103

E_el=-9004498.71, G_thr=1814.79, G_solv=-227.73, PBEh-3c/CPCM, TS 2b

C	-3.45386	0.42024	0.74438
C	-3.70475	-0.97822	0.68568
C	-5.01082	-1.49535	0.75308
C	-6.04966	-0.62751	0.84851
C	-5.84229	0.74632	0.78831
C	-4.58146	1.30475	0.69012
C	-2.66835	-1.95117	0.55278
N	-1.41146	-1.70863	0.42922
Al	-0.69125	0.14839	0.21205
O	0.15454	-0.03165	2.11456
C	1.54187	0.25393	2.18333
C	1.93567	1.39414	3.13563
F	-7.31511	-1.08470	0.95894
C	-4.44309	2.79534	0.36069
C	-5.78654	3.52806	0.45843
O	-2.24568	0.87424	0.83983
C	-0.46908	-2.72402	0.27262
C	0.77757	-2.30094	-0.20758
C	1.81603	-3.21399	-0.34617
C	1.61030	-4.54844	-0.04106
C	0.37017	-4.97300	0.41856
C	-0.66206	-4.06513	0.58419
N	0.87336	-0.93372	-0.46320
C	1.73658	-0.44728	-1.28188
C	1.91793	0.94229	-1.57150
C	2.91600	1.23863	-2.51946
C	3.17488	2.53872	-2.81193
C	2.48248	3.57080	-2.18549
C	1.49092	3.33510	-1.25311
C	1.17643	1.97571	-0.92949
O	0.25960	1.70362	-0.05956

C	0.72875	4.50444	-0.62796
C	0.79999	4.46909	0.90582
F	4.11840	2.85610	-3.72136
O	-1.36602	0.27893	-1.54575
C	-1.48079	-0.50377	-2.55866
O	-1.10545	-1.66428	-2.61099
C	-2.15541	0.15734	-3.74846
C	1.30102	5.85566	-1.06934
C	-0.73149	4.45723	-1.09219
C	-3.47769	3.55496	1.27533
C	-3.98094	2.88571	-1.10194
C	2.44582	-0.97000	2.36510
H	-3.01381	-2.98290	0.51545
H	2.39803	-1.12681	-1.81787
H	3.46500	0.43952	-3.00287
H	2.74703	4.58206	-2.46070
H	-6.71986	1.37708	0.79162
H	-5.17129	-2.56634	0.74587
H	2.79953	-2.88342	-0.65535
H	2.42621	-5.25201	-0.13707
H	0.21414	-6.01227	0.67470
H	-1.60606	-4.40868	0.98643
H	1.22641	6.00823	-2.14765
H	0.73169	6.65546	-0.59366
H	2.34532	5.98186	-0.77531
H	-1.28980	5.29237	-0.66222
H	-0.79853	4.53386	-2.17933
H	-1.22629	3.54124	-0.78763
H	1.83650	4.45720	1.24830
H	0.32547	5.36362	1.31486
H	0.29116	3.60763	1.32698
H	-3.82601	3.53970	2.30607
H	-2.46948	3.15704	1.25591

H	-3.42933	4.59823	0.95377
H	-3.03551	2.37533	-1.27176
H	-4.72365	2.43926	-1.76693
H	-3.85617	3.93104	-1.39520
H	-6.20642	3.47797	1.46519
H	-5.63225	4.58198	0.22247
H	-6.53009	3.15116	-0.24606
H	-2.20870	-0.51192	-4.60426
H	-1.61610	1.06141	-4.03509
H	-3.16655	0.46828	-3.47941
H	1.24367	2.22317	3.00747
H	2.92263	1.76741	2.86955
H	1.94211	1.10427	4.17967
H	1.81313	0.63376	1.20220
C	2.07391	-2.24952	2.75570
C	2.98793	-3.28257	2.89873
C	4.32115	-3.08284	2.62286
C	4.72825	-1.83913	2.18391
C	3.79734	-0.82708	2.05107
F	0.81564	-2.59855	2.95813
F	2.56857	-4.48690	3.27826
F	5.19749	-4.07368	2.75615
F	6.00807	-1.62607	1.88373
F	4.25552	0.33331	1.57138
H	-3.70580	-3.67516	3.69216
C	-3.46773	-2.61874	3.71288
C	-4.48696	-1.69461	3.93512
H	-5.50361	-2.03890	4.07143
C	-4.19574	-0.34238	3.99681
H	-4.96310	0.39320	4.19902
C	-2.87860	0.03901	3.80774
C	-2.28456	1.37990	3.96859
O	-2.84884	2.39878	4.27430

H	-1.37957	-2.96233	3.40262
C	-2.14994	-2.21857	3.53475
C	-1.86710	-0.86144	3.55249
C	-0.54038	-0.13454	3.65891
O	0.28523	-0.50177	4.48022
O	-0.97822	1.28576	3.78767

103

E_el=-9004588.57, G_thr=1810.77, G_solv=-221.27, PBEh-3c/CPCM, Int. 6b

C	-4.02799	-3.00323	0.43003
C	-3.14863	-1.90665	0.37938
C	-3.13717	-1.02365	-0.73853
C	-4.01532	-1.30957	-1.83683
C	-4.85671	-2.39958	-1.73700
C	-4.86681	-3.22485	-0.61598
C	-2.22125	-1.79723	1.46426
N	-1.32809	-0.88470	1.59855
C	-0.41887	-0.87763	2.65494
C	0.18009	0.36277	2.92311
C	1.15501	0.44745	3.91127
C	1.50585	-0.67043	4.64667
C	0.90273	-1.89367	4.38952
C	-0.04541	-1.99852	3.38862
N	-0.24581	1.41635	2.11425
C	0.00619	2.64453	2.38865
C	-0.35361	3.78053	1.59528
C	-1.02122	3.67519	0.34080
C	-1.36377	4.88976	-0.34528
C	-0.97496	6.09108	0.20999
C	-0.29013	6.15850	1.42063
C	0.01185	5.03488	2.12125
O	-1.31079	2.54067	-0.17965
Al	-1.35094	0.83242	0.51689
O	-2.96556	1.30443	1.38722
C	-3.40571	1.27727	2.59077
C	-4.79675	1.87835	2.73310
F	0.05896	7.37433	1.89559
C	-2.20034	4.85651	-1.62427
C	-2.38976	6.25704	-2.21683
O	-2.33853	-0.02101	-0.79814

C	-4.02027	-0.43145	-3.08950
C	-5.02849	-0.93213	-4.12889
F	-5.72372	-4.26953	-0.59415
C	-3.59696	4.31590	-1.28053
C	-1.54356	4.00056	-2.71800
C	-2.63912	-0.44265	-3.76255
C	-4.41375	1.00778	-2.73201
O	-2.82110	0.82769	3.56504
O	0.25462	0.45452	-0.36470
C	1.10421	-0.48315	-0.60931
O	1.78550	-0.16114	-1.79384
C	2.56355	-1.12273	-2.45979
C	2.95701	-0.53132	-3.80567
C	0.61130	-1.92227	-0.57558
C	-0.35903	-2.53858	-1.34443
C	-0.64562	-3.87120	-1.08248
C	0.03296	-4.57424	-0.08696
C	1.02690	-3.95751	0.65833
C	1.29485	-2.62678	0.39013
C	2.29345	-1.72542	1.00295
O	2.15540	-0.52361	0.45981
O	3.12959	-1.99891	1.82346
C	3.78666	-1.56255	-1.67253
H	-2.30253	-2.57595	2.22245
H	0.53353	2.88441	3.31170
H	0.53029	5.09634	3.07080
H	-1.20383	7.02562	-0.28319
H	-5.54016	-2.64351	-2.53853
H	-4.02518	-3.66371	1.28901
H	1.67088	1.37949	4.09946
H	2.27608	-0.59246	5.40211
H	1.19941	-2.77432	4.94292
H	-0.46558	-2.96813	3.15632

H	-3.55698	3.33532	-0.81305
H	-4.20431	4.23312	-2.18551
H	-4.11045	4.99393	-0.59539
H	-2.13124	4.06857	-3.63697
H	-1.47181	2.95342	-2.44276
H	-0.53788	4.36272	-2.94120
H	-2.91510	6.93237	-1.53857
H	-2.99263	6.18328	-3.12348
H	-1.44039	6.72027	-2.49470
H	-1.86542	-0.02514	-3.12309
H	-2.66854	0.15205	-4.67877
H	-2.35088	-1.45904	-4.04188
H	-4.45301	1.61962	-3.63693
H	-3.70490	1.46801	-2.05127
H	-5.40151	1.03749	-2.26763
H	-6.05394	-0.91681	-3.75392
H	-4.80515	-1.94398	-4.47414
H	-4.99610	-0.27910	-5.00251
H	-5.51012	1.32454	2.12020
H	-5.13638	1.86575	3.76664
H	-4.80281	2.90685	2.36868
H	1.96596	-2.01750	-2.65862
H	3.54829	0.37629	-3.69587
H	3.53928	-1.25492	-4.37599
H	2.06256	-0.28752	-4.37878
H	1.57350	-4.49130	1.42506
H	-0.21556	-5.61160	0.09619
H	-1.41713	-4.37067	-1.65425
H	-0.88518	-2.00252	-2.12171
C	4.06808	-2.90527	-1.47990
C	5.15445	-3.33503	-0.73865
C	5.99685	-2.40368	-0.16950
C	5.75118	-1.05528	-0.35200

C	4.66209	-0.65083	-1.09849
F	3.28066	-3.84132	-2.01037
F	5.38946	-4.63393	-0.57359
F	7.04539	-2.79852	0.54134
F	6.57048	-0.15966	0.18897
F	4.47242	0.65260	-1.25295

33

E_el=-3714781.33, G_thr=421.51, G_solv=-213.30, PBEh-3c/CPCM, Fluorinated Free Chain (F)

C	3.78958	-1.69040	-0.48058
C	5.10120	-1.31869	-0.22770
C	5.38072	-0.02249	0.18448
C	4.34993	0.89221	0.33477
C	3.03031	0.53376	0.08398
C	2.75916	-0.77017	-0.31874
C	1.90111	1.55777	0.18761
O	0.79907	1.14501	-0.23048
C	1.36638	-1.23718	-0.58994
O	0.75888	-1.58967	0.55639
C	-0.58846	-2.03007	0.46379
C	-0.91423	-2.80126	1.73142
O	2.19544	2.66817	0.64362
O	0.92390	-1.48420	-1.67929
C	-1.49591	-0.84367	0.23589
H	5.90032	-2.03831	-0.35366
H	6.40339	0.27441	0.38258
H	4.55262	1.90823	0.64706
H	3.56944	-2.70113	-0.80306
H	-0.69013	-2.71055	-0.38292
H	-0.79645	-2.19894	2.62960
H	-0.26402	-3.67230	1.81634
H	-1.94418	-3.15524	1.68857
C	-1.46088	0.26497	1.06802
C	-2.24402	1.37734	0.83268
C	-3.08773	1.40040	-0.25977
C	-3.14595	0.31142	-1.10666
C	-2.34929	-0.78897	-0.85279
F	-0.69036	0.27335	2.14566
F	-2.19844	2.42225	1.65149

F	-3.85320	2.46214	-0.49006
F	-3.96870	0.32727	-2.15514
F	-2.44396	-1.82383	-1.68905

Section S12: References

[1] N. J. Van Zee, M. J. Sanford, G. W. Coates, Journal of the American Chemical Society 2016, ^ 138, 2755-2761.

[2] B. A. Abel, C. A. Lidston, G. W. Coates, Journal of the American Chemical Society 2019, 141, 12760-12769.

[3] K. Sakakibara, K. Nakano, K. Nozaki, Macromolecules 2007, 40, 6136-6142.

[4] Y. Liu, J. Zhou, M. R. Wasielewski, H. Xing, C. A. Mirkin, Chemical Communications 2018, 54, 12041-12044.

[5] U. Boudriot, R. Dersch, B. Goetz, P. Griss, A. Greiner, J. Wendorff, 2004.

[6] M. Rist, A. Greiner, RSC Applied Polymers 2024, 2, 642-655.

[7] a) N. D. Harrold, Y. Li, M. H. Chisholm, Macromolecules 2013, 46, 692-698. b) C. Fornacon-Wood, M. R. Stühler, A. Millanvois, L. Steiner, C. Weimann, D. Silbernagl, H. Sturm, B. Paulus, A. J. Plajer, Chem. Commun. 2024, 60, 7479–7482.

[8] M. E. Fieser, M. J. Sanford, L. A. Mitchell, C. R. Dunbar, M. Mandal, N. J. Van Zee, D. M. Urness, C. J. Cramer, G. W. Coates, W. B. Tolman, Journal of the American Chemical Society 2017, 139, 15222-15231.

[9] C. Patrick, G. Prosser, Nature 1960, 187, 1021-1021.

[10] S. Tsuzuki, T. Uchimaru, M. Mikami, The Journal of Physical Chemistry A 2006, 110, 2027-2033.

[11] P. Pracht, F. Bohle, S. Grimme, Physical Chemistry Chemical Physics 2020, 22, 7169-7192;

[12] S. Grimme, F. Bohle, A. Hansen, P. Pracht, S. Spicher, M. Stahn, The Journal of Physical Chemistry A 2021, 125, 4039-4054.

[13] C. Bannwarth, E. Caldeweyher, S. Ehlert, A. Hansen, P. Pracht, J. Seibert, S. Spicher, S. Grimme,
WIREs Computational Molecular Science 2021, 11, e1493, DOI 10.1002/wcms.1493.

[14] C. Bannwarth, S. Ehlert, S. Grimme, J. Chem. Theory Comput. 2019, 15, 1652–1671, DOI 10.1021/acs.jctc.8b01176.

[15] S. Ehlert, M. Stahn, S. Spicher, S. Grimme, J. Chem. Theory Comput. 2021, 17, 4250–4261, DOI 10.1021/acs.jctc.1c00471.

[16] R. Ahlrichs, M. Bär, M. Häser, H. Horn, C. Kölmel, Chemical Physics Letters 1989, 162, 165–169, DOI 10.1016/0009-2614(89)85118-8.

[17] S. Grimme, J. G. Brandenburg, C. Bannwarth, A. Hansen, The Journal of Chemical Physics 2015,143, 054107, DOI 10.1063/1.4927476.

[18] J. Zha, T. Ding, J. Chen, R. Wang, G. Gao, F. Xia, J. Phys. Chem. A 2020, 124, 7991–7998, DOI 10.1021/acs.jpca.0c04662.

[19] J. P. Perdew, K. Burke, M. Ernzerhof, Physical Review Letters 1996, 77, 3865–3868, DOI 10.1103/physrevlett.77.3865.

[20] J. P. Perdew, M. Ernzerhof, K. Burke, The Journal of Chemical Physics 1996, 105, 9982–9985, DOI 10.1063/1.472933.

[21] F. Weigend, R. Ahlrichs, Phys. Chem. Chem. Phys. 2005, 7, 3297–3305, DOI 10.1039/B508541A.

[22] E. Caldeweyher, S. Ehlert, A. Hansen, H. Neugebauer, S. Spicher, C. Bannwarth, S. Grimme, The Journal of Chemical Physics 2019, 150, 154122, DOI 10.1063/1.5090222.
[23] S. Spicher, S. Grimme, J. Chem. Theory Comput. 2021, 17, 1701–1714, DOI 10.1021/acs.jctc.0c01306.
[24] A. Klamt, The Journal of Physical Chemistry 1995, 99, 2224–2235, DOI 10.1021/j100007a062.
[25] A. Klamt, V. Jonas, T. Bürger, J. C. Lohrenz, The Journal of Physical Chemistry A 1998, 102, 5074–5085, DOI 10.1021/jp980017s.
[26] A. Klamt, COSMO-RS: From Quantum Chemistry to Fluid Phase Thermodynamics and Drug Design, 2005.
[27] F. Eckert, A. Klamt, 2019.
[28] V. Ásgeirsson, B. O. Birgisson, R. Bjornsson, U. Becker, F. Neese, C. Riplinger, H. Jónsson, J. Chem. Theory Comput. 2021, 17, 4929–4945, DOI 10.1021/acs.jctc.1c00462.
[29] F. Neese, WIREs Computational Molecular Science 2022, 12, e1606, DOI 10.1002/wcms.1606.
[30] F. Neese, Journal of Computational Chemistry 2023, 44, 381–396, DOI 10.1002/jcc.26942.
[31] F. Neese, Journal of Computational Chemistry 2003, 24, 1740–1747, DOI 10.1002/jcc.10318.
[32] M. Cossi, N. Rega, G. Scalmani, V. Barone, J. Comput. Chem. 2003, 24, 669.
[33] S. Grimme, Chemistry–A European Journal 2012, 18, 9955–9964, DOI 10.1002/chem.201200497.
[34] N. Mardirossian, M. Head-Gordon, J Chem Phys 2016, 144, 214110, DOI 10.1063/1.4952647.
[35] P. R. Horn, Y. Mao, M. Head-Gordon, Phys. Chem. Chem. Phys. 2016, 18, 23067–23079, DOI 10.1039/C6CP03784D.
[36] M. Bursch, J.-M. Mewes, A. Hansen, S. Grimme, Angewandte Chemie International Edition 2022, 61, e202205735, DOI 10.1002/anie.202205735.
[37] J.-D. Chai, M. Head-Gordon, Phys. Chem. Chem. Phys. 2008, 10, 6615–6620, DOI 10.1039/B810189B.
[38] L. Bennett, B. Melchers, B. Proppe, 2020, DOI 10.17169/refubium-26754.
